# Exploring and validating observations of non‐local species in eDNA samples

**DOI:** 10.1002/ece3.10612

**Published:** 2023-10-14

**Authors:** Coen Westerduin, Marko Suokas, Tuukka Petäjä, Ulla Saarela, Seppo Vainio, Marko Mutanen

**Affiliations:** ^1^ Ecology and Genetics Research Unit, Faculty of Science University of Oulu Oulu Finland; ^2^ Department of Physics, Institute for Atmospheric and Earth System Research (INAR) University of Helsinki Helsinki Finland; ^3^ CRC, The Faculty of Medicine University of Oulu Oulu Finland; ^4^ Laboratory of Developmental Biology, Faculty of Biochemistry and Molecular Medicine University of Oulu Oulu Finland

**Keywords:** contamination, dietary studies, environmental DNA, false positives, Lepidoptera, metabarcoding

## Abstract

The development of DNA‐based methods in recent decades has opened the door to numerous new lines of research in the biological sciences. While the speed and accuracy of DNA methodologies are clearly beneficial, the sensitivity of these methods has the adverse effect of increased susceptibility to false positives resulting from contamination in field or lab. Here, we present findings from a metabarcoding study on the diet of and food availability for five insectivorous birds, in which multiple lepidopteran species not known to occur locally were discovered. After describing the pattern of occurrences of these non‐local species in the samples, we discuss various potential origins of these sequences. First, we assessed that the taxonomic assignments appeared reliable, and local occurrences of many of the species could be plausibly ruled out. Then, we looked into the possibilities of natural environmental contamination, judging it to be unlikely, albeit impossible to fully falsify. Finally, while dissimilar combinations of non‐local species' occurrences across the samples did not initially suggest lab contamination, we found overlap with taxa and sequences handled in the same lab, which was undoubtedly not coincidental. Even so, not all exact sequences were accounted for in these locally conducted studies, nor was it clear if these and other sequences could remain detectable years later. Although the full explanation for the observations of non‐local species remains inconclusive, these findings highlight the importance of critical examination of metabarcoding results, and showcase how species‐level taxonomic assignments utilizing comprehensive reference libraries may be a tool in detecting potential contamination events, and false positives in general.

## Graphical abstract

The sensitivity of DNA‐based methodologies provides great benefits while also carrying inherent risks of contamination, and subsequently false positives in the final datasets. Here, we present the observations of sequences assigned to presumed false positive non‐local species, investigate their likely origins, and discuss how best to resolve them.

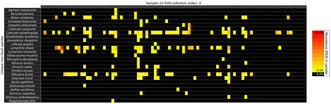

## 1 INTRODUCTION

The development of high‐throughput sequencing technologies and creation of public reference libraries has led to the common usage of DNA sequencing‐based methodologies in the biological sciences (Alberdi et al., 2019; Ratnasingham & Hebert, 2007; Taberlet et al., 2012; Wirta et al., 2015; Yu et al., 2012; Zaiko et al., 2022). These methods frequently utilize barcodes—standardized short, informative fragments of DNA for identification of taxa (Hebert et al., 2003)—to assess the contents of mixed environmental samples, known as metabarcoding. Metabarcoding has made it possible to study complex food webs (Kaunisto et al., 2017), host–parasitoid relationships (Peralta et al., 2015) and monitor species in ecosystems (Thomsen & Sigsgaard, 2019; Yu et al., 2017) with considerable sensitivity, accuracy and speed.

Despite the methods' increasingly wide use, questions remain regarding the trade‐off between false positive and false negative results in sample replication and data clean‐up (Mata et al., 2019; Zaiko et al., 2022). In addition, the benefits of using molecular operational taxonomic units (MOTUs) versus identification of taxa remains a point of contention (Blaxter et al., 2005; Curry et al., 2018). These two issues are partially connected, since false positives can be difficult to detect without taxonomic assignment of the sequences (Furlan et al., 2020). Potential sources of false positives are widespread. They range from contamination in the field or later in the lab to amplification and sequencing errors, including tag‐jumping and chimeric sequences (Drake et al., 2022). These variable origins require an equally diverse range of measures to resolve them (if they cannot be prevented), and some should arguably not even be classified using identical terminology. After all, DNA of some ‘erroneously present taxa’ (Drake et al., 2022) would have been accurately observed by the sensitive methodology, even if originating from a time or place beyond the study's interest (Darling et al., 2021). Problematically, reliably discriminating between false positives and genuine observations may require not just an extensive library of reference barcodes, but also knowledge on often complex and taxon‐dependent species delimitation in DNA barcodes (DeSalle et al., 2005; Naciri & Linder, 2015) and information on local species occurrences, the latter of which may defeat the purpose of certain metabarcoding studies. As with many variables that have prevented standardization in metabarcoding procedures, the specific obstacles and their optimal solutions may be strongly context‐dependent (Alberdi et al., 2019; Drake et al., 2022).

Finland is among the nations with the highest percentage of its fauna and flora represented in barcode reference libraries (Roslin et al., 2021). For example, out of >2600 species of Lepidoptera reported for the country, 92% is covered by at least one reference cytochrome c oxidase subunit I (COI) barcode from the national Finnish Barcode of Life initiative (FinBOL, finbol.org), and only a single species lacks any reference barcode in the complete Barcode of Life Data Systems (BOLD) database. In addition, local fauna and particularly lepidopterans have been mapped and monitored extensively. These factors made metabarcoding a promising method to study the diets of several local insectivorous birds: the crested tit (*Lophophanes cristatus*, Linnaeus 1758), willow tit (*Poecile montanus*, Conrad von Baldenstein 1827), blue tit (*Cyanistes caeruleus*, Linnaeus 1758), great tit (*Parus major*, Linnaeus 1758) and pied flycatcher (*Ficedula hypoleuca*, Pallas 1764). Numerous aspects of the biology of several of these species have a long tradition of research both locally (Vatka et al., 2014) and elsewhere across their range (e.g. Kvist et al., 2003; Lasters et al., 2021; van Oers et al., 2014; Visser et al., 2003). Even so, the ecologically fundamental aspect of these species' diets has remained largely unclear, owing to the difficulties of identifying their small and often soft‐bodied (larval) prey with sufficient taxonomic resolution and accuracy using traditional methods (Carew et al., 2013; Oehm et al., 2011). Not only was the context described herein thus expected to be ideal for metabarcoding, metabarcoding also appeared an ideal method for this study subject.

Here, we present the discovery of sequences matched to not locally occurring lepidopteran species in faecal samples from the birds, as well as from frass (arthropod faecal material sampled from the environment) collected in the same area. We look into the various pathways via which this DNA could have ended up in our samples, namely unknown local occurrences of the species, airborne transfer and subsequent contamination in the field, errors in amplification/sequencing or lab contamination. Finally, we discuss the ramifications of these findings for metabarcoding studies. With metabarcoding and other DNA‐based techniques becoming increasingly widely used and accessible, awareness of the potential for such observations is vital.

## 2 MATERIALS AND METHODS

Field work took place at sites near Oulu, Finland (approx. 65°05′ N 25°33′ E, with nests and frass collectors within a 4 km radius). These areas consist of a patchwork of younger and older stands of predominantly Scots pine (*Pinus sylvestris*, L.), Norway spruce (*Picea abies*, (L.) H.Karst) and birch (*Betula* spp., L.), interspaced with swamps and clear‐cut areas (Orell & Ojanen, 1983). In recent years, the area has contained ~350 nest boxes (used mainly by blue tits, great tits and pied flycatchers) and >1000 provided rotten tree stumps (in which crested tits and willow tits excavate nesting holes). Nests were typically visited weekly to monitor their progress, with chicks ringed at the ages of 13 (willow tits and blue tits) or 14 days (crested tits and great tits). Although not ringed, pied flycatchers were visited and briefly handled to collect faecal samples at the age of 13 days. When handled, nestlings will commonly defecate (King et al., 2008), allowing practical and non‐invasive collection of samples for our dietary study. Faecal sacs were collected into 5 mL ethanol‐filled tubes (pooled per nest), and deposited in a freezer at −20°C later that day until further analysis. Animal handling and ringing complied with Finnish laws, under licence 180 from the Finnish Ringing Centre. Frass was simultaneously collected to provide information on prey availability. It was obtained from three different types of collectors, hereafter referred to as ‘funnel’, ‘sheet’ or ‘tarpaulin’ samples. The first were plastic funnels (diameter 35 cm, with a coffee filter underneath) attached to birch trunks in four field locations. The second were cloth sheets (1 × 1 m) placed just above the ground with birch canopy overhead, in two locations. Contents from the funnels and sheets were collected weekly. The third were plastic tarpaulins (2 × 3 m) with a funnel and attached coffee filter in the centre, placed under mixed birch/pine/spruce canopy in two (2018) or four (2019 onwards) locations; these were typically collected twice‐weekly due to large yields. Different frass collection locations were 1.5–8 km distant from each other. Frass samples were placed in the freezer at −20°C on the day of collection unless wet, in which case closed collection envelopes/filters were first dried at room temperature before deposition into the freezer the next day.

In preparation for DNA extraction, samples were transferred to 2 mL Eppendorf tubes, removing plant material where possible. The first samples from 2017 (see below for details; ‘Original 2017’ in Table 1) were manually homogenized using a new, sterile plastic pestle for each sample. For all samples processed later, a metal bead was instead added to each tube, and they were shaken for 1 min at 30 Hz (bird samples) or 25 Hz (frass) in a TissueLyser (Qiagen). Beads were then removed from tubes with tweezers. Both methods were expected to be equally sterile. The change was motivated mainly by the substantial time savings offered by the second method (with manual homogenization taking ~2 min/sample, and the TissueLyser handling batches of up to 24 samples in just 1 min), in addition to using fewer disposable tools. A subset (250 mg or as much as possible) of the sample was transferred to another Eppendorf for later DNA extraction. Excess ethanol (for bird samples) was evaporated by placing open tubes in a flow cabinet for 24 h; samples were otherwise kept at −20°C. Tubes were opened slowly and open tubes were placed spread out in a rack to minimize cross‐contamination. Tools used to transfer material were cleaned in ethanol and flamed between uses. Metal beads were cleaned with water and soap, followed by ethanol and finally placed in an oven at 120°C for at least 24 h to sterilize them between uses. Sample preparation took place in a separate room from later DNA extraction, amplification and sequencing steps.

**TABLE 1:** Number of samples for each category (bird species or frass collector) analysed in the various processing batches/sequencing runs, listed in chronological order.

		Original 2017	Batch 2	Batch 3	Batch 4	Batch 5	Batch 6
Crested tit		9 (2017)	7 (2018)	4 (2019)	8 (2019)	1 (2017) 19 (2020)	–
Willow tit		24 (2017)	29 (2018)	17 (2019)	11 (2019)	4 (2017) 26 (2020)	5 (2017)
Blue tit		16 (2017)	27 (2018)	1 (2018) 17 (2019)	7 (2019)	20 (2020)	–
Great tit		24 (2017)	23 (2018)	17 (2019)	8 (2019)^a^	7 (2017) 4 (2018) 1 (2019) 24 (2020)^a^	20 (2017)
Pied flycatcher		–	25 (2018)	17 (2019)	11 (2019)	4 (2019)^a^ 22 (2020)^a^	–
Frass	Funnel	–	–	13 (2017)	–	–	–
	Sheet	22 (2017)	17 (2018)	12 (2019)	7 (2019)	–	–
	Tarpaulin	–	16 (2018)	2 (2018) 47 (2019)	24 (2019)^a^	–	–

*Note*: Numbers in brackets indicate the years in which the listed samples were collected.

^a^ These counts include the following number of technical replicate pairs: 1 [great tit, batch 4]; 3 [frass tarpaulin, batch 4]; 4 [great tit, batch 5]; 2 [pied flycatcher, batch 5 (2019)]; 2 [pied flycatcher, batch 5 (2020)].

Further steps in the pipeline largely followed the methods extensively detailed in a previous study (Rytkönen et al., 2019); the main points and any deviations from prior methods will briefly be described below. DNA extraction was performed using QiaAmp Fast DNA Stool Mini Kit (Qiagen), following the manufacturer's instructions with two exceptions. Firstly, incubation of the lysate in step 7 was done for 1 h (up from prescribed 10 min). Secondly, centrifugation during DNA elution in step 14 took place for 5 min (up from prescribed 1 min). The ZBJ‐ArtF1c/ZBJ‐ArtR2c (Zeale et al., 2011) primers targeting a subsection of the standardized COI region of mitochondrial DNA (mtDNA) were selected for DNA amplification. Despite shortcomings in the amplification of certain taxa, these primers perform well for the main group of interest here, Lepidoptera (Clarke et al., 2014; Elbrecht et al., 2019; Jusino et al., 2019; Piñol et al., 2015, 2018). A two‐stage polymerase chain reaction (PCR) protocol was followed, amplifying the relevant sequences with the aforementioned primers in the first step and adding unique sample‐specific tags and adapters in the second step. Two PCR reactions were performed for each sample in the first step, combining both afterwards to partially compensate for stochasticity. The PCR cycle consisted of: 98°C (2 min); 38 cycles [98°C (10 s), 58°C (30 s), 72°C (15 s)]; 72°C (5 min) after the last cycle. Amplified products were purified with AMPure XP (Agencourt). The second PCR reaction step adding tags as well as Ion Torrent‐specific adapters A & TrP1 used the first step's primers as linkers. The shorter PCR in the second step was: 98°C (90 s); eight cycles [98°C (10 s), 63°C (20 s), 72°C (20 s)]; 72°C (5 min) after the last cycle. New products were purified with AMPure XP, quantified with picogreen dsDNA (Thermofisher) and pooled in equimolar ratios, followed by further AMPure XP purification prior to sequencing. For full details, see our previous publication (Rytkönen et al., 2019). Amplicons were sequenced with Ion 316 v2 Chips on an Ion Torrent PGM (Thermofisher) at the Biocenter Oulu (University of Oulu). Initially, negative controls (consisting of the same chemicals but no target DNA) were employed only for DNA extraction and amplification, and discarded prior to sequencing when no DNA was found in them. Informed by the potential contamination described in this study and methodological insights from literature, we later (Table 1: Batch 4–6) retained the negative controls (at least two per extraction/sequencing batch) and included these in the sequencing runs.

Raw fastq files from sequencing were imported as single‐end reads and processed using Qiime 2 (Bolyen et al., 2019). Imported reads were demultiplexed to individual samples based on their 9 bp unique identifier sequence at the 5′ end of each read with the q2‐cutadapt plugin and trimmed for primer sequences from both ends (Martin, 2011). Amplicon variant tables and representative sequence files of each variant were created from processed reads using dada2 denoiser (q2‐dada2 plugin) with default parameters and a truncation length of 145 bp (Callahan et al., 2016).

ASVs (Amplicon Sequence Variants) were assigned taxonomic identities primarily using the Identification Engine in BOLD (boldsystems.org), first using the Species Level Barcode Records database, then the All Barcode Records data if no match was available in the former (typically for dipterans lacking detailed taxonomic information). As BOLD lacks explicit specified query coverage/overlap, at least one sequence from a public record of the best matching taxon was compared to the relevant query sequence. Due to the short target region (145 bp), we discarded matches based on <96% coverage (<140 bp overlap). Identities discarded this way often stood out as they typically had only a single reference record as a match and were phylogenetically distant from all other matches. The Full Length Record database was used when poor overlap between query sequence and suggested matches was suspected, and overlap could not be verified because of non‐public reference sequences. If a single species presented the best match, this would be the assigned identity; if multiple species were equally likely (typically congeneric species), the lowest shared taxonomic level would be used instead. If locally occurring species were listed at the same match % as species not known to occur locally, the former were favoured. A threshold of 97% match was used for normal identifications, but for the non‐local species presented here, we accepted only those with a stricter >99% match (a maximum of 1 bp difference between queried and reference sequences). If no matches were available above this threshold, ASVs received the ‘No match’ identity instead. ‘No match’ ASVs are included in the full data (Appendix S1), but are otherwise omitted from most analyses. Species distributions, and thus their local occurrences, were determined by expert opinion (M. Mutanen), complemented by data from the Finnish Biodiversity Info Facility (laji.fi) and the Global Biodiversity Information Facility (gbif.org). ‘Non‐local species’ are defined as species with known distribution ranges >200 km from the study area. Given typically limited mobility in the lepidopterans studied here and their widespread monitoring, this was considered sufficient to rule out local occurrences. Many species found outside this range would furthermore be constrained by the lack of host plants and climatic conditions, preventing local settlement in the focal boreal area. For the purposes of this paper, we only consider the not locally occurring Lepidoptera encountered, as other arthropod taxa are typically too limited in both known distribution ranges and abundance of reference barcodes for similar assessments.

Maps illustrating the species' occurrences were produced using GBIF's online tool https://api.gbif.org/v2/map/debug/ol/#. Links to the relevant datasets of coordinates are provided at the respective figures; GBIF data is licenced under CC BY‐NC 4.0, while base map data is © OpenStreetMap contributors and © OpenMapTiles.

Potential alternative taxonomic assignments were investigated via the Protax web application (laji.fi/en/theme/protax; Roslin et al., 2021), using the full‐length COI, probability threshold of 0.1 and 140 bp minimal overlap settings. This application is aimed at specifically identifying Finnish species (currently Insecta and Arachnida), using reference data from FinBOL. The resulting taxonomic placements based on Bayesian probabilistic statistics would be expected to present ‘unknown’ values or low probabilities for lower taxonomic levels for which reference sequences are absent, as would be the case for completely foreign species.

For quantitative analyses, we calculated Relative Read Abundances (RRA, Deagle et al., 2019), applied a filter removing any ASVs with an RRA <0.1% or with a ‘no match’ identity, then recalculated the RRA so that all values within a sample added up to 100% again. A handful of additional appearances of non‐local species occurred below the 0.1% threshold; these are left out of consideration here as they would be plausibly classified as artefacts and have little impact on any ecological conclusions drawn from the data due to their limited presence. Abundances of ASVs with the same taxonomic assignment were merged for some analyses, while abundances of individual ASVs are used where relevant (e.g. when discussing variant haplotypes).

Since high relative abundances for non‐local species could be the result of limited other material in the respective samples, and chimeras and the impact of PCR stochasticity may also be more prevalent in samples with low template DNA (Alberdi et al., 2018), we looked for correlations between these abundances and the total number of reads in the samples, and between the abundances and the number of different overall ASVs in R (R Core Team, 2020), using Spearman's rank correlation coefficient.

Alignment of sequences (ClustalW) and the creation of Neighbor‐Joining trees to investigate similarities were performed using MEGA‐X, version 10.1.8 (Kumar et al., 2018) on default settings.

This study mainly focusses on the results of a sequencing batch in which the non‐local species were most prevalent. These 95 samples (‘Original 2017’ in the overview in Table 1) were collected in the spring/summer of 2017, and analysed in the lab over December 2017–January 2018. The findings presented in this paper prompted us to, in subsequent years, analyse an additional 50 samples that had been collected in 2017. These were prepared for DNA extraction and processed in 2019 (Batch 3, Table 1) and 2021 (Batch 5 and 6, Table 1) alongside any later samples they were sequenced with. Complementary data is provided by another 474 samples collected in later years (2018–2020; see Table 1 for a full overview). Given the possibility of contamination playing a role in the findings presented here, it should be noted that the samples were typically prepared in the autumn after collection and sequenced within the next year, that is, the six sequencing runs presented here took place over the course of almost 5 years (early 2018–late 2021), typically with 6–12‐month intervals between each.

To investigate specific potential sources of contamination, we compared our findings to sequencing results from two unrelated studies for which samples were handled in the same lab around the same time as the original 2017 batch. The first were faecal samples for a dietary study (J. C. Senar, unpublished data) collected near Barcelona, Spain, from blue tits and great tits, which would have been processed using the same protocol described above. We performed DNA extractions and PCR of these samples in December–January 2018. The second concerned a phylogenetic study on Cochylina (Brown et al., 2020), for which the data are publicly available on BOLD at dx.doi.org/10.5883/DS‐COCHY. For these, DNA extractions took place in January 2018 and while we have no record of the exact PCR date, it must have occurred prior to sequencing, which occurred in April 2018. After looking for exact sequence matches, Neighbor‐Joining trees were created using all our ASVs assigned to non‐local species and these two datasets to alternatively find the nearest similar sequences (and in the case of the Barcelona data, subsequently identify these via BOLD).

## 3 RESULTS

The initial 95 samples from 2017 contained 952 unique ASVs to which 351 different identities were assigned: species‐level identities were possible for 303 of these, genus for 36, family for 11, order for 1 (full dataset available in Appendix S1). One sample (#363) was omitted from subsequent analyses as it contained a total of only 150 reads, compared to a mean of 28,145 (SE: 743) reads in the retained 94 samples. The 0.1% RRA filter removed ASVs with 14–78 reads from the samples. For reference, although no negative controls were unfortunately sequenced for this batch, the highest read count for any ASV in negative controls in later runs was 7. The 130 ASVs removed after receiving the ‘no match’ identity had a mean RRA of 1.26% (SE: 0.36%), excluding complete absences. Six hundred thirty ASVs assigned 283 unique identities remained after filtering.

### 3.1 Non‐local BOLD identities

Eighty four ASVs were identified as 25 different non‐local species (overview in Table 2); 34 of the 84 assignments were made based on 100% matches in BOLD (for full details on these, see Appendix S2). The majority of taxonomic assignments to these non‐local species was unambiguous, with multiple independent matches to reference sequences of solely the non‐local species, and often additional matches to the same species at a lower percentage (i.e. variant haplotypes) below these. In many cases, there was a >2% gap to the nearest alternative species compared to the best match, with the nearest locally occurring alternative frequently even further removed. In cases where taxonomic assignments were less straightforward, we attributed the ASV to a species for which overlap between queried and reference sequence could be checked (public data) and was sufficient (≥140 bp). Note that whenever seemingly equally likely matches were listed (*Dryobota labecula* (Esper 1788) for *Rileyiana fovea*; *Satyrium spini* (Denis & Schiffermüller 1775) for *Satyrium esculi*; *Aethes baloghi* (Sabourin & Metzler 2002) for *Aethes seriatana*; cases 18, 19 and 22, respectively in Appendix S2), the alternative identities also represented non‐local species.

**TABLE 2:** Overview of non‐local species encountered in initial 2017 samples. Species names listed here are those used throughout the main text, while the listed synonyms are restricted to those encountered in relevant data used in the study, that is, BOLD output and GBIF data sources.

#	Family	Species	Synonym (if any)	Authority
1	Geometridae	*Agriopis marginaria*		Fabricius 1776
2	Nolidae	*Bena bicolorana*		Fuessly 1775
3	Geometridae	*Biston stratarius*	*Biston strataria*	Hufnagel 1767
4	Geometridae	*Campaea honoraria*	*Gerinia honoraria*	Denis & Schiffermüller 1775
5	Erebidae	*Catephia alchymista*		Denis & Schiffermüller 1775
6	Erebidae	*Catocala conjuncta*	*Catocala coniuncta*	Esper 1787
7	Erebidae	*Catocala nymphagoga*		Esper 1787
8	Noctuidae	*Dryobotodes tenebrosa*		Esper 1789
9	Pieridae	*Gonepteryx cleopatra*		Linnaeus 1767
10	Erebidae	*Lithosia quadra*		Linnaeus 1758
11	Erebidae	*Lymantria dispar*		Linnaeus 1758
12	Erebidae	*Lymantria monacha*		Linnaeus 1758
13	Lasiocampidae	*Malacosoma neustria*		Linnaeus 1758
14	Geometridae	*Menophra abruptaria*		Thunberg 1792
15	Erebidae	*Minucia lunaris*		Denis & Schiffermüller 1775
16	Erebidae	*Ocneria rubea*		Denis & Schiffermüller 1775
17	Notodontidae	*Peridea anceps*		Goeze 1781
18	Noctuidae	*Rileyiana fovea*		Treitschke 1825
19	Lycaenidae	*Satyrium esculi*		Hübner 1804
20	Noctuidae	*Xestia agathina*		Duponchel 1827
21	Noctuidae	*Xylocampa areola*		Esper 1789
22	Tortricidae	*Aethes seriatana*		Zeller 1875
23	Tortricidae	*Henricus cognatus*	*Henricus cognata*	Walsingham 1914
24	Tortricidae	*Henricus umbrabasanus*		Kearfott 1908
25	Tortricidae	*Platphalonidia felix*		Walsingham 1895

### 3.2 Protax identities

Some species had such numerous reference material in BOLD that the full Top 100 only displayed identical matches to that species, possibly hiding alternative identities from view. The Protax taxonomic assignments, used to investigate the validity of these identities and explore similarities to local species, are displayed alongside their BOLD counterparts in Appendix S2. Of the 84 ASVs, 24 were assigned the same identities as per BOLD, predominantly with very high probabilities (>0.8 for 21, >0.95 for 16). These included all matches for *Lymantria dispar* and *L. monacha*, which had typically been lacking alternatives in BOLD. No likely matches were presented for lower taxonomic ranks for any of the remaining 60 ASVs, with no probabilities >0.5 at the genus level and none >0.25 for species. For 16, the nearest local alternatives from the BOLD results were also listed in the Protax output, albeit consistently at low probabilities. This was the case for most instances where *Catocala nymphagoga* was the best match in BOLD, and the locally occurring *C. fraxini* (Linnaeus 1758) and *C. sponsa* (Linnaeus 1767) were presented as slightly less likely matches. Overall, results from BOLD were supported for many non‐local but still Finnish taxa (ASVs assigned to *Agriopis marginaria*, *Bena bicolorana*, *Biston stratarius*, *Lithosia quadra*, *L. dispar*, *L. monacha*, *Malacosoma neustria* and one of two ASVs identified as *Minucia lunaris*), while no probable alternative identities were provided for any of the residual ASVs.

### 3.3 Abundances of non‐local species

Read counts for individual ASVs matched to non‐local species ranged from 19 to 6036 per sample (after 0.1% RRA filter). The abundances of individual ASVs matching non‐local species ranged from 0.10% to 22.75% per sample, and the total contribution of non‐local species in a sample ranged from 0.10% to 55.35% (excluding samples where non‐local species were absent).

### 3.4 Non‐local species across sample categories

**FIGURE 1:**
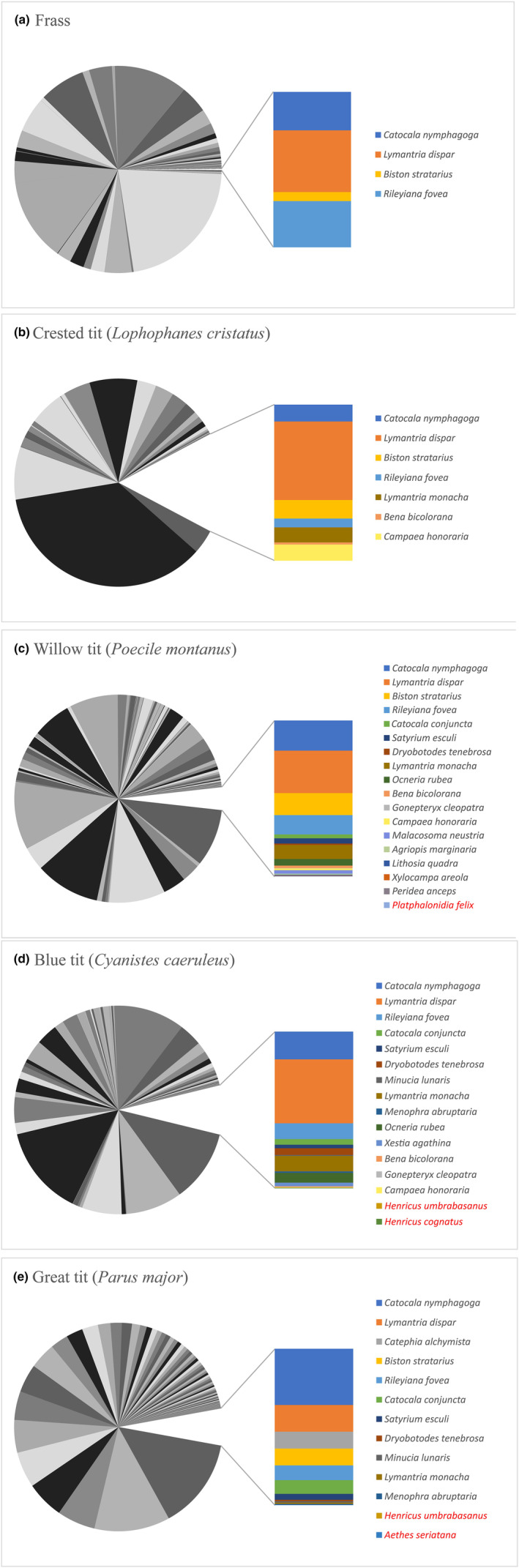
Average abundance (RRA) of arthropod species across sample categories: environmental arthropod frass (a) and diets of the four bird species (b–e). Local arthropod species are displayed as the incomplete circles in greyscale, with a cut‐out slice for the combined non‐local species. Individual non‐local species are shown in the separate bar graphs in colour, and only their identities are included in the legend. North American species are indicated in red text. For full details on abundances of individual local species, see Appendix S1. RRA, Relative Read Abundances.

The non‐local species were present in material from all four bird species sampled in 2017 as well as the frass (sheet) samples, as displayed in Figure 1a–e. The lowest number of non‐local species (4) and lowest overall abundance (0.23%) was found for the frass (Figure 1a). The highest number of different non‐local species (18) was present in samples from the willow tit (Figure 1c), while the crested tit samples showed the largest overall presence of these (15.50%), albeit based on a relatively low number (7) of different non‐local species (Figure 1b).

### 3.5 Geographic distributions of non‐local species

**FIGURE 2:**
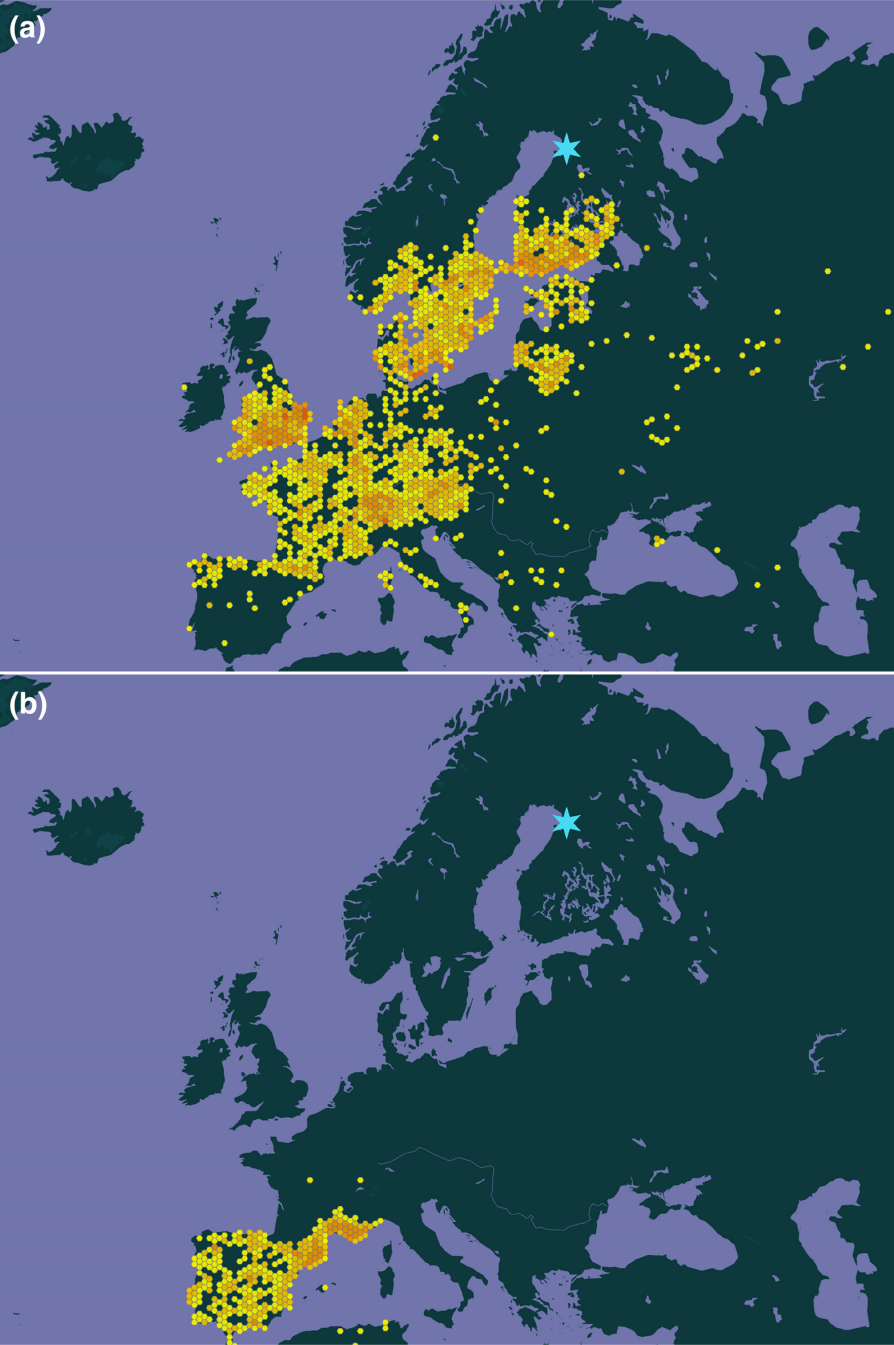
Maps indicating approximate distributions based on observational data for (a) *Lymantria monacha* and (b) *Satyrium esculi*. Occurrences are indicated by yellow‐orange markers (darker colours indicate higher numbers), while the location of the field area near Oulu is indicated by a light blue star. Data source: GBIF.org occurrence downloads https://doi.org/10.15468/dl.4nr6n8 and https://doi.org/10.15468/dl.y9d4yk (18 May 2022).

Of the non‐local species described here, 10 have occurrences or at least observations from southern Finland (approx. 200–500 km south of the study area), with most restricted to the southern coastal or archipelago region (*A. marginaria*, *B. bicolorana*, *M. lunaris*, *L. dispar*, *M. neustria*; *Peridea anceps, Xestia agathina*) and only three species more frequently seen further inland (*B. stratarius*, *L. quadria*, *L. monacha*). Eleven of the remaining species have European distributions, varying from widely ranging across the continent to more limited distributions typically concentrated in the west or south. Examples of non‐local Finnish and southwestern European distributions are illustrated by *L. monacha* and *S. esculi* in Figure 2a,b, respectively. It is worth noting that almost all of the European species may exist in sympatry in north‐eastern Spain, southern France and north‐western Italy. The last four species (*A. seriatana*, *Henricus cognatus*, *H. umbrabasanus*, *Platphalonidia felix*) are North American; all ASVs assigned to North American species were rare (occurring in one to two samples) and had low abundances (0.11%–0.26% per sample). Maps illustrating the approximate occurrences of all 25 species are provided in Appendix S3.

### 3.6 Spread of non‐local species across samples

**FIGURE 3:**
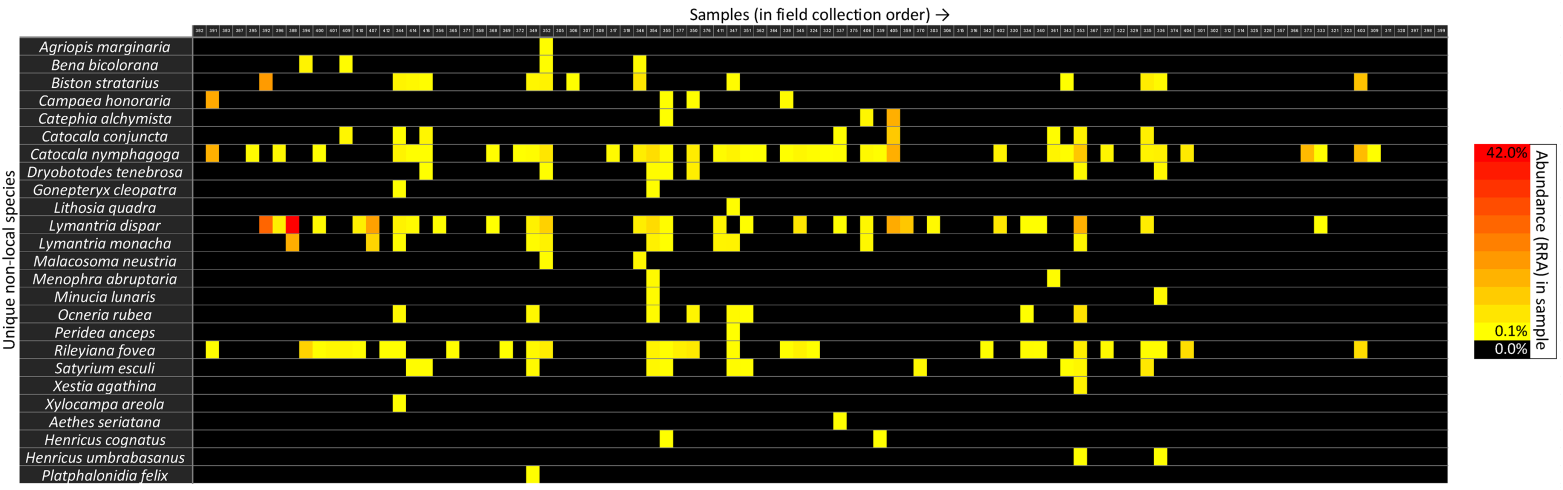
Distributions of non‐local species across the samples. Samples shown as columns, in order of field collection date, while different species are displayed as rows. Coloured cells indicate occurrence of the species, with the shade indicating the abundance (% RRA). Sample numbers in the top row correspond to those listed in Appendix S1. For ASV‐level information, see Appendix S4: Figure S4A. ASV, Amplicon Sequence Variants; RRA, Relative Read Abundances.

**FIGURE 4:**
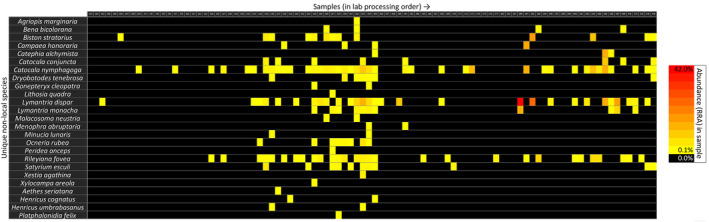
Distributions non‐local species across the samples. Samples shown as columns, in order of processing in the lab, while different species are displayed as rows. Coloured cells indicate occurrence of the species, with the shade indicating the abundance (% RRA). Sample numbers in the top row correspond to those listed in Appendix S1. For ASV‐level information, see Appendix S4: Figure S4B. ASV, Amplicon Sequence Variants; RRA, Relative Read Abundances.

**FIGURE 5:**
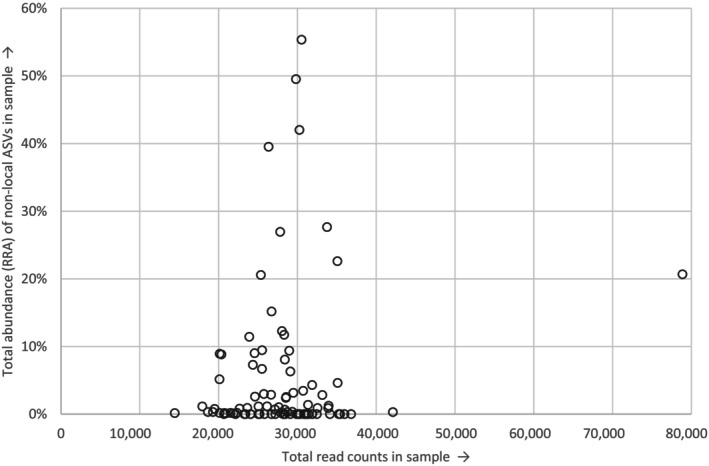
Relation between read counts and abundance of non‐local ASVs in samples. Presented read counts include ‘No match’ ASVs and ASVs filtered out by 0.1% RRA threshold, to explicitly include other potential chimeras or artefacts. ASV, Amplicon Sequence Variants; RRA, Relative Read Abundances.

**FIGURE 6:**
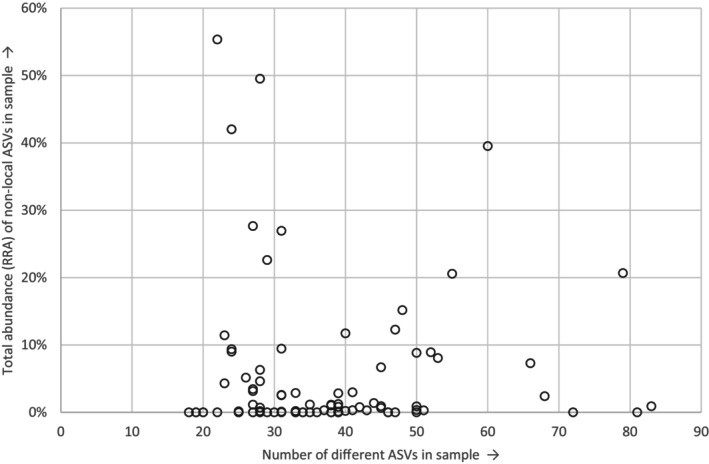
Relation between the number of variant ASVs and abundance of non‐local ASVs in samples. Presented read counts include ‘No match’ ASVs and ASVs filtered out by 0.1% RRA threshold, to explicitly include other potential chimeras or artefacts. ASV, Amplicon Sequence Variants; RRA, Relative Read Abundances.

The distributions of non‐local species over the samples in this batch are displayed in Figures 3 and 4 (for corresponding distributions of all ASVs, see Appendix S4). Of the 94 samples, 59 contained one or several non‐local species, with the most common ASV (7a, *L. dispar*) present in 35 samples. The various species and specific ASVs occurred in numerous different combinations and quantities across the samples, lacking any clear pattern. We placed samples in order of field collection date to look into the onset, peak and end of specific species' appearances (Figure 3). Many non‐local species occurred in samples collected across the period (14/5/2017–17/7/2017), with the peak abundances variably early, in the middle or near the end of this time window. We similarly explored potential lab contamination events by instead placing the samples in lab processing order (Figure 4). No clear pattern was found here, as the same ASVs commonly occurred both in early and later samples, with high abundances frequently at a considerable distance from other occurrences. Furthermore, we found non‐local species in samples collected across the study area (data not shown). Neither the total read counts (Figure 5) nor the number of different ASVs (Figure 6) in a sample appeared to have any explanatory power over the abundance of non‐local ASVs in the same sample. Both showed widely scattered distributions and low Spearman correlations at −.075 and .103 for total read counts and ASVs respectively.

### 3.7 Similarities to other ASVs

A phylogenetic tree constructed for the full set of 952 different ASVs (provided in its entirety in Appendix S5) revealed that, despite limited potential variation in the short 145 bp sequences, variant ASVs identified as the same non‐local species consistently clustered together and clusters displayed little similarity to ASVs from local species in the dataset. ASVs of different non‐local species had a scattered distribution, with no clear semblance to other non‐local species. While the non‐local ASVs or clusters generally appeared distinct from other species, a comparatively large number of ‘No match’ ASVs appeared among their clusters. The distribution of the various similar *Catocala* species showed a distinct branch for two ASVs assigned to *C. fraxini*, while a single *C. sponsa* match (which had an equally likely assignment to *C. nymphagoga*) was placed among the *C. nymphagoga* ASVs.

### 3.8 Barcelonan and North American data

Of the 84 ASVs assigned non‐local species identities, 22 fully matched those in the Barcelona data. We also specifically compared only those ASVs from our study that were identified based on 100% matches to the BOLD reference library, to prevent potential sequencing errors from inflating the apparent differences. Of the 22 ASVs found in both studies, 21 were those with 100% matches to reference data, leaving 13 of our ASVs with 100% matches with no equivalents in the Barcelona data. Among the ASVs with overlap in both studies were representatives of 19 of the 25 non‐local species, with only *P. anceps*, *Xylocampa areola* and the four North American species being absent. The phylogenetic tree featuring the full set of Barcelona ASVs and our ASVs for non‐local species (not shown) did reveal other sequences matched to *P. anceps* and *X. areola* in the Barcelona data. Of the four North American species from our data similarly compared to the locally handled Cochylina, one of the *H. cognatus* ASVs (with a 100% match to reference data) and the *P. felix* ASV matched completely to sequences from that study. The second *H. cognatus (*with a 99.31% match) and the *A. seriatana* sequence both deviated by 1 bp for the nearest equivalents, while the *H. umbrabasanus* ASV differed for three separate and distant base pairs. The closest matches for all ASVs were identical to the taxonomic assignments given by BOLD.

### 3.9 Further samples from 2017 and 2018–2020

No ASVs assigned to the same 25 or any other non‐local species were found in the 50 additional samples from 2017 that were evaluated in subsequent years. Across 474 samples analysed from later years, 22 samples contained a total of 25 different ASVs identified as non‐local species (sequences, identities and abundances for these are provided in Appendix S6). The affected samples include some originating from all five bird species (pied flycatchers having been added from 2018 onwards) but none from any frass collectors, with observations across the field area and time period (mid‐May to mid‐July). Thirteen non‐local species were found in these later samples, of which five were new. Ten of the 18 ASVs matched to previously encountered species were identical to data from the original 2017 batch; three of the eight new ASVs matched 100% to reference library sequences. A total of 17 out of 27 non‐local occurrences in later sequencing runs were found for the second batch, which exclusively contained material collected in 2018 (see Table 1). In contrast to the earlier findings, any ASVs for non‐local species had low occurrences (1–2 samples each, 13 samples in total) and abundances (<2% in 10 samples, maximum 11.75%). In the subsequent batch (#3), only a single sample contained a non‐local species (*L. dispar*) with an abundance of 0.45%. Three new and three previously seen non‐local species were found in 6 samples in the next batch (#4), with a new sequence for *C. nymphagoga*. The only new non‐local species without a European distribution (the Australian *Chloroclystis metallospora*; Turner 1904) was found here, although it had a wide range of alternative matches in BOLD at slightly lower percentages, none of which represented Finnish taxa. Abundances of non‐local species were predominantly low at <1% for five samples and 11.44% in the last. The latter was one of a pair of replicates for which subsets had been taken from the same original sample, but neither of the two non‐local species found in this sample were recorded for the replicate—limited similarities were found for the taxa in these two subsamples in general. Another pair of replicates for which only one contained a non‐local species (a new *L. monacha* haplotype) was found in the last large sequencing batch (#5). Here, both replicates were otherwise largely in agreement, but differences existed for ASVs with abundances <1%, and the *L. monacha* ASV had an abundance of 0.46% in the one sample. The same ASV was found at 0.61% in a different sample in the same batch. Of the in total 15 new ASVs for non‐local species found in later years, none fully matched material from Barcelona, although eight were identified as species that were previously found from there. For the new non‐local species, only *Stauropus fagi* (Linnaeus 1758), observed in the second batch, was detected in these data, although the exact sequences differed. None of the aforementioned non‐local species, nor any others, were found in any of the negative controls that were sequenced.

## 4 DISCUSSION

In our first large batch of metabarcoding results for a dietary study, we encountered a substantial group of ASVs that were matched to species which were not known to occur near the study area. Some of these ASVs occurred frequently, and several were at times highly abundant. This was not repeated in additional samples from the same and other years that were subsequently analysed. However, several of the same ASVs, new ASVs matching the same species or other ASVs matching novel non‐local species were found in later analyses, albeit less commonly. These data led us to question the validity of both pre‐existing knowledge and our own findings, given their clear mutual exclusivity. Below, we explore the potential origins of these species, their DNA or at least sequences matching their DNA, in our dataset.

### 4.1 Accuracy of taxonomic assignments

The first question our findings raised was whether the identities had been correctly assigned. The vast majority of ASVs presented here were assigned species based on multiple matches with high similarities (99%–100%) to reference sequences in BOLD and an absence of probable matches to more locally occurring species. Since complete coverage of all potential haplotypes of a species in the reference library would not be expected, matches lower than 100% are often considered acceptable, with a threshold >98% commonly used in metabarcoding studies (Esnaola et al., 2018; King et al., 2015; Vesterinen et al., 2016). We increased this to >99% here for the non‐local species, specifically validated overlap between queried and reference sequences, and looked for more likely (i.e. more local) identities with similar barcodes as well. The alternative Protax taxonomic assignments validated several cases which had previously been less clear, while never disagreeing with the prior findings. In short, taxonomic assignments were unambiguous for almost all of these ASVs (with potential caveats for the *C. nymphagoga* matches, those with equal likelihoods for *R. fovea*/*D. labecula* and the *C. metallospora* match in a later batch). Without prior knowledge on the distribution ranges of the relevant species, the observations of these species in our data would have appeared perfectly reliable. Assuming the taxonomic assignments of the specimens used for the reference barcodes were correct—highly likely, since many of our identifications were based on multiple independent reference sequences—the detection of DNA belonging to these non‐local species in our samples does appear to be genuine. This then leads to the next question: how did it get here?

### 4.2 Overlooked local occurrences

The most straightforward explanation would be that the Lepidoptera in question did in fact occur locally after all. Although this is impossible to fully disprove, it is unlikely for a number of reasons. Firstly, the field area has a fairly isolated location, near the western coast in northern Finland. Southern species would have had to cross the Gulf of Bothnia from Sweden, or traverse much of Finland, to reach this area, and have done so without observation along the route. For some species, the study area is half the European continent removed from their nearest known occurrences, while the North American species are naturally found even more distantly. That said, many lepidopterans are shifting northwards with ongoing climate change (Hällfors et al., 2021). Natural dispersal distances, limited by adult flight capabilities and ballooning by larvae, can be highly variable. For example, while European *L. dispar* females are poor fliers (McCormick et al., 2017), recent findings suggest larval dispersal in the same species may greatly exceed distances found in past experiments (Martemyanov et al., 2019). Still, even the nearest non‐local species would have needed to exceed the highest estimates of ballooning ranges (and many times the maximum distances recorded from direct observations) to cover the necessary distances in one or a few generations. Secondly, the study area would be unsuitable for several of these species even if their local presence had remained overlooked. For instance, *B. bicolorana* and *P. anceps* larvae feed on oak leaves, presently restricting their natural distribution to the south of Finland. For species not constrained by food plants like *L. dispar* or *L. monacha*, whose larvae can feed on locally abundant birch or coniferous trees respectively, lethally low winter temperatures currently limit expansion into higher latitudes (Fält‐Nardmann, Ruohomäki, et al., 2018). Thirdly, even if the species occurred locally, not all are suitable prey for all bird species in whose samples they were encountered. While parids are capable of dealing with the irritating hairs present on several of the caterpillars of non‐local species encountered in our data (Barbaro & Battisti, 2011), the pied flycatchers have no such ability, to the best of our knowledge, and their samples nonetheless contained DNA of both *L. dispar* and *L. monacha*. These samples, from late June to early July, likely pre‐date the adult flight period of these lepidopterans (late July–August), when they might be more suitable prey for the flycatchers. (It is unclear whether the flycatchers would consume the pupae of these species and whether the *Lymantria* species would be in their pupal stage quite yet at the time of the first observations). Finally, local settlement of the non‐local species would likely have resulted in continued observations in later years, which was not the case for many.

### 4.3 Local DNA without species' occurrences

Even without local presence of the non‐local Lepidoptera, it might have been possible for their DNA to be physically present. A potential pathway would be via *Wolbachia*, a common endosymbiont in Lepidoptera capable of promoting interspecific mitochondrial introgression even across distinct orders (Ahmed et al., 2016; Bennett et al., 2012; Smith et al., 2012). However, the latter appears rare and of limited impact on COI barcoding accuracy (Smith et al., 2012). Furthermore, *Wolbachia* may be entirely absent from some encountered species (Martemyanov et al., 2014; Pavlushin et al., 2017). A situation in which mtDNA from non‐local lepidopterans is endemic in local species furthermore poorly fits with the sharp decline in observations of this DNA after the first year, where a continued presence would be expected.

Alternatively, the potential for longer‐distance airborne contaminants in field sites has been hypothesized for eDNA studies in the past (Furlan et al., 2020). Atmospheric transport is known to carry diverse biotic and abiotic aerosol particles from across and beyond Europe to Finland, with southernly flows common according to air mass backtrajectories from May to July 2017 (results not shown). Vertebrate DNA from somewhat distant subjects has recently been successfully sampled directly from the air (Clare et al., 2022), and the same could plausibly be done for shed cells or hairs from arthropods. Observations there were limited to distances of a few hundred metres, however, not hundreds of kilometres. Environmental factors such as ultraviolet radiation, pH and bacterial activity are expected to result in rapid degradation of genetic material (Alberdi et al., 2019; Oehm et al., 2011), suggesting numerous transmission events would be required for our observations across several months. In addition, although DNA amplification exhibits biases that can strongly influence relative quantities of certain sequences (Piñol et al., 2015), the non‐local species would have to be substantially overamplified compared to all local sources of DNA to achieve abundances as high as those seen in our data, unless genuine high quantities of genetic material of these non‐local species were present in the environment. Additionally, presence of the relevant DNA in the air would logically mainly result in observations of this material in the environmental frass samples, yet non‐local species were rarely and sparsely (original 2017) or never (subsequent batches) found in these. The slightly longer storage at room temperature of some frass samples is unlikely to have caused sufficient degradation of DNA to explain this difference, given the stability of DNA in these (Rytkönen et al., 2019).

More directional transmission could theoretically have taken place via migratory birds passing through southern Europe on seasonal migration, like cuckoos (*Cuculus canorus*, Linnaeus 1758) and pied flycatchers, which arrive to our field areas in late April–early May (personal observations and laji.fi observational data for North Ostrobothnia). While the flycatchers are not known to consume the hairy caterpillars, cuckoos do consume these in large quantities (Barbaro & Battisti, 2011), but the latter very rarely parasitise nests of the focal cavity‐breeding birds due to insufficient nest entrance sizes (Grim et al., 2014; Rutila et al., 2002). As for abiotic transference above, it is furthermore unclear how either flycatchers or cuckoos would transfer any major amounts of DNA to the local area and into the samples.

### 4.4 Field contamination

It is worth noting that practical limitations precluded particularly sterile sample collection in the field. This potentially resulted in some frass from 1 week remaining in collectors until subsequent weeks, or transfer of small amounts of material across bird samples collected on the same day, with the latter sometimes collected from bird bags, scales or researchers' hands or clothing. The distribution of non‐local occurrences across the samples indicated no clear clusters arising from widespread accidental transmission, however. Irregularity across the season or years would be more in line with other biotic and abiotic factors that could cause field contamination—changing wind directions, fluctuating arthropod population sizes and outbreaks downwind or the stochastic odds of small fragments ending up in the samples.

### 4.5 Lab‐based contamination

The sensitivity of metabarcoding makes contamination in the lab an ever‐present risk. Technical replicates are theoretically beneficial in establishing accuracy of findings, but in practice not necessarily cost‐effective nor straightforward to analyse, since genuine rare taxa and contaminants are not easily separated (Alberdi et al., 2019; Ficetola et al., 2015). Our study contained a handful of replicates to explore variation between subsamples. Typically, findings for both subsamples were broadly similar for abundant taxa, while rare taxa were often only found in one of the replicates; this is in line with generally high risks of false negatives in metabarcoding studies (Ficetola et al., 2015). Non‐local species were twice found in one of a pair of replicates. For the only case where the non‐local species were abundant (>5% RRA) in these, both subsamples were dissimilar in other ways too, potentially indicating that the sample was poorly mixed rather than contaminated. Multiple subsamples could only be taken from samples with sufficient material, and precisely those risk being inadequately homogenized due to their increased volume. Besides these, we opted for biological over technical replicates since our original research intent was documenting the diets of generalist birds. In this context, ensuring detection of rare taxa was not a primary concern, nor was sample collection prohibitive from either financial or animal welfare perspectives.

With few technical replicates and no non‐local species encountered in negative controls, we attempted to trace back potential sources of the non‐local species indirectly. The distribution of non‐local occurrences across the samples did not indicate any clear point of contamination, suggesting either another cause or that the contamination was widespread. We cannot rule out that some cross‐contamination occurred when sample tubes were open when drying, but this would not account for the initial introduction of the non‐local species, nor the occurrences at high abundances. The many variable combinations in which non‐local species were present makes it unlikely that, for example, a single reused tool was the cause, even considering methodological stochasticity. Although there were limits to how well we could reconstruct events several years down the line, two other analyses conducted locally around the same time appeared relevant. While the North American Cochylini studied for a phylogenetic study (Brown et al., 2020) were processed by different people in different parts of the lab, a different source for these four species appears unlikely. The low abundances of these ASVs are also in line with contamination, even though the exact pathway and the absence of precisely matching sequences in several cases raises further questions. This is reinforced by the findings regarding the European species and their potential origin in the Barcelona samples. Considering that the ASVs identified as *R. fovea* may have been *D. labecula*, all species found in the initial 2017 batch could be found both in that region and the relevant data. Many of the exact sequences were different here too, however, something that will be discussed further below. Abundances of the European non‐local species were substantial in some cases, which would likely have required contact with PCR products or a strong primer bias towards these species. Since abundances for the same ASVs were not consistently high, the latter seems unlikely. The absence of non‐local species in later samples from the same year (2017) does hint at a lab‐based origin for the sequences. On the other hand, we have the absence of many specific sequences from the Barcelona data, the continued presence of some species over many years, and the emergence of wholly new variant sequences in later years. To the best of our knowledge, no other studies using these or similar species were conducted in this lab during this time besides those mentioned above.

### 4.6 Sources of variation

Errors in amplification or sequencing are further potential origins for the sequences, or at least sequence variants. It seems highly unlikely that chimeras would create this many different ASVs with (near‐)perfect matches to existing species. The non‐local species were distinct from each other and had no particular similarity to local species either, making a shared chimeric origin unlikely. Their frequent semblance to unidentified ASVs (which would include chimeras) is more easily explained by the fact that sequences required a >99% match to be identified as non‐local species, meaning that similar ASVs might have fallen below this threshold. Errors in sequencing could be plausible explanations for some variant ‘haplotypes’ we found, especially since our analyses used ASVs instead of MOTUs. For instance, visual inspection of the aligned sequences showed that a section often having seven repeated T‐bases was shortened to six T‐bases in several sequences, an erroneous call known to occur for homopolymers by the Ion Torrent PGM. While this barely impacted taxonomic assignments (at worst, it may have created a poorer match and possibly leave an ASV unidentified), it would have resulted in the apparent existence of a different haplotype. This specific issue was only seen six times, however, and some ASVs with <100% matches are plausibly genuine, simply lacking a reference barcode of the exact same haplotype. Since ASVs with 100% matches are likely to be free of these or other errors, it is puzzling how 100%‐matching ASVs lacking equivalents in the Barcelona data were found in the original 2017 dataset, and that more were encountered in the following years. A further potential origin of apparent haplotypic variation are nuclear mitochondrial DNA transpositions (NUMTs) (Leite, 2012; Song et al., 2008). NUMTs of a few hundred bp in length are especially common, although comparatively rare in Lepidoptera (Hebert et al., 2023). NUMTs can display strong similarities to mtDNA (including the COI region) and be co‐amplified and mistaken for mtDNA as a result—differential mutations in either the COI or NUMT occurring after transference to the nucleus could create the appearance of similar but slightly differing barcodes (Moulton et al., 2010). While our sequences could theoretically include NUMTs, the identifications are unlikely to be distorted, as NUMTs are rarely similar beyond closely related species and the full‐length reference barcodes are far less likely to include NUMTs (Hebert et al., 2023).

### 4.7 Consequences and implications of unexpected findings

Upon first noticing the non‐local species in the data from 2017, our initial reaction was to effectively run through the steps described above, albeit more superficially: we validated identities, explored the affected samples, attempted to rule out local occurrences and imagined alternative origins of the DNA sequences. In doing so, we strove to keep a balance between narrowing down options by ruling out pathways, while keeping an open mind for unknown factors. Lacking a satisfying answer, we hoped a combination of additional samples from 2017 and further material from later years would eventually resolve matters. The absence of continued high‐abundance occurrences of the non‐local species in later years pointed towards a one‐off (a)biotic transmission of DNA or contamination, while the absence in separately analysed additional samples from 2017 favours the latter explanation. Our methodologies have largely remained unaltered so as to allow fair comparisons over the years—besides the decision to sequence negative controls; a change explicitly made to better detect potential contamination events.

If frequent and abundant observations of non‐local species had continued, measures to rule out or validate the aforementioned explanations would have been next on the agenda. Pheromone traps were considered as a non‐DNA‐based control to either solidify or falsify our assumption that the species were non‐local and the observations genuine false positives (Darling et al., 2021). Even in the comparatively well‐studied Lepidoptera focussed on here, we cannot always rule out theoretical occurrences based purely on incomplete data regarding exact host plants, temperature limits or dispersal abilities. The latter at least should not be relied on too heavily, considering frequently aided dispersal via human actions (Gippet et al., 2019). The unexpected settlement of species like the infamous defoliator *L. dispar* (Fält‐Nardmann, Klemola, et al., 2018) would have been considerable news, though forests at high latitudes are expected to be outside its habitable range for the time being (Fält‐Nardmann, Ruohomäki, et al., 2018).

The initial detection of the non‐local species prompted us to consider if dietary metabarcoding would be a possible method in monitoring potential invasive species, though this might seem fanciful based on the potential susceptibility to contamination described here. Now, our findings might even raise the question on the validity of eDNA research, given the manifold sources of potential false positives impacting the results. In the years since we settled upon our particular protocol, many studies have suggested methods to improve accuracy of metabarcoding research, but also to adjust expectations. After all, as Darling et al. (2021) highlight, we should perhaps not decry our sensitive methodology for detecting trace amounts of DNA from times or areas that happen to be outside of our particular research interests while simultaneously desiring that same low detection threshold.

Specific bioinformatic pathways play a large role in the balance between false negatives and false positives, though optimal settings are context‐dependent and may require validation using non‐metabarcoding data sources (Drake et al., 2022; Marques et al., 2020). Stricter denoising and MOTU clustering can at least remove apparent haplotypic variation, though certain studies might benefit from this extra level of detail (Corse et al., 2019; Elbrecht et al., 2018). Indeed, the distribution of exact ASVs across samples and years also appears relevant to understanding the origins of the sequences of our non‐local species. If high reliability of specific observations is needed, the use of multiple molecular markers can be a solution, although there are some caveats to this. Perhaps most importantly, it will do little if reference data for other regions is lacking, as will be the case for anything besides COI for many taxa, including our focal arthropods (Galan et al., 2018). At present, studies employing multiple primer sets often do so to gain additional taxonomic coverage, rather than seeking overlap to validate findings, while explicit overlap is needed for validation (Corse et al., 2019; De Barba et al., 2014). It is also worth noting that combining datasets with dissimilar taxonomic coverage and detail is inherently fraught with practical obstacles (da Silva et al., 2019). Although generally used to test for the sensitivity of a protocol for detecting taxa of interest (Alberdi et al., 2019), mock communities can also play an important role in setting thresholds for filtering data (Drake et al., 2022). Studies like ours, on diverse and a‐priori unknown species communities, will however struggle to take into account even a fraction of the relevant species, of which each might have divergent amplification biases (Braukmann et al., 2019; Clarke et al., 2014; Piñol et al., 2015). Ficetola et al. (2015) showed in experiments and models how multiple replicates and negative controls could help detect and exclude false positive observations. Nevertheless, few ecological studies (including ours) use (m)any technical replicates, and it is easy to see why given the associated costs and workload. A simple recommendation easily applied to almost any context, however, is the plentiful inclusion of negative controls. More cost‐efficient than duplicating regular samples, these will give the most straightforward insight into both what might be contaminating, and how much. Their data can be combined with mock communities to finetune both overall and sequence‐specific abundance filters to remove false positives (Drake et al., 2022). If added at different moments throughout the pipeline, as performed by Galan et al. (2018), negative controls can even allow for the tracing of contamination to particular steps, perhaps allowing future prevention or reduction.

## 5 CONCLUSIONS

In summary, our findings suggest that certain sequences belonging to non‐local species originated from lab contamination, but the picture for the whole situation is partially still muddled—and due to the difficulties of investigating these matters retroactively, will likely remain so. Despite this, we believe this already provides worthwhile information for future metabarcoding studies. Previous research has handled observations of non‐local species in highly variable ways. Among pragmatic solutions are reassigning non‐local taxa to higher taxonomic levels until a locally occurring taxon is reached (De Barba et al., 2014), including non‐local taxa but accepting that lower‐level taxonomic assignments may be inaccurate (Verkuil et al., 2022) or only assigning identities at higher taxonomic levels to sequences with <99% matches to reference records (Evens et al., 2020). Based on our findings, in some way ignoring the detailed taxonomic information that metabarcoding can offer risks overlooking problematic sequences from contamination or other sources. At the same time, incomplete reference databases will necessitate some flexibility, as species lacking reference records will otherwise remain unidentified, resulting in potentially numerous false negatives. Creating a reference library of local food sources alongside a dietary study may help (see e.g. McClenaghan et al., 2019), but it is understandable that this is not always practical or affordable, nor likely complete. Even with an exhaustive reference database for local species, external factors may introduce unexpected taxa to field sites, observations of which can be difficult to either verify or rule out (see also Furlan et al., 2020; Marques et al., 2020).

Our findings demonstrate that reliable species‐level identifications can be obtained for taxa well‐represented in public reference libraries. They furthermore highlight the resulting possibilities of connecting this information to ecological data on these species, including but certainly not limited to detection of false positive observations. Although previous work has demonstrated that, for instance, reliable diversity metrics may be obtainable purely using MOTUs (e.g. Marques et al., 2020), losing out on these additional possibilities would be a major disadvantage and potential risk. Only the combination of an extensive reference database, species‐level taxonomic assignments and detailed pre‐existing knowledge on local fauna allowed us to detect the problematic species we found in the first place.

Based on our observations, we would caution against complete reliance on metabarcoding data in contexts where species identities are relevant but reference databases relatively incomplete. However, when solid reference data are available, detailed taxonomic assignments can be considered among the various defences against false positives; worthwhile even if species‐level information is not otherwise of interest for the study. DNA‐based methods can provide many advantages over traditional methods of identification: in certain contexts, they may not just offer the best, but in fact the only way to practically investigate specific questions. Metabarcoding is, however, not a method devoid of its own shortcomings. Some strengths, like its sensitivity, can even prove a disadvantage if faced with contamination. The ease with which metabarcoding data can be obtained may hide the necessity to critically examine these results. As the present work demonstrates, manifold factors may influence environmental DNA and metabarcoding studies, and we urge researchers to remain aware of any known issues and wary of those that are as of yet still unknown.

## AUTHOR CONTRIBUTIONS

**Coen Westerduin:** Conceptualization (lead); data curation (equal); formal analysis (lead); funding acquisition (equal); investigation (lead); methodology (lead); visualization (lead); writing – original draft (lead); writing – review and editing (lead). **Marko Suokas:** Data curation (lead); investigation (equal); methodology (equal); writing – original draft (equal). **Tuukka Petäjä:** Conceptualization (supporting); investigation (supporting); writing – original draft (equal). **Ulla Saarela:** Investigation (supporting); methodology (supporting); writing – original draft (supporting). **Seppo Vainio:** Conceptualization (equal); investigation (supporting); writing – original draft (equal). **Marko Mutanen:** Conceptualization (lead); formal analysis (equal); funding acquisition (equal); investigation (equal); methodology (equal); supervision (lead); writing – original draft (equal).

## Supporting information


Appendix S1

Appendix S2

Appendix S3

Appendix S4

Appendix S5

Appendix S6

## Data Availability

Detailed information on the relevant samples, sequences and assigned identities is provided in the Appendices [Link], [Link], [Link], [Link], [Link], [Link]. Complete reads for the focal samples are furthermore uploaded to NCBI's Sequence Read Archive (SRA), accession numbers SAMN31544596–SAMN31544690.
