# Supplementary material for: Exploring and validating observations of non‐local species in eDNA samples
Source: Ecol Evol. 2023 Oct 14;13(10):e10612. doi: 10.1002/ece3.10612 (PMC10576249; doi:10.1002/ece3.10612)
Supplement: Supplementary file 2 — Appendix S2 [file ECE3-13-e10612-s003.docx]

**Exploring and validating observations of non-local species in eDNA samples**

Coen Westerduin, Marko Suokas, Tuukka Petäjä, Ulla Saarela, Seppo Vainio, Marko Mutanen

**Supporting Information 2.** Full BOLD and FinPROTAX output for the 84 ASVs assigned non-local species.

For each ASV matched to a non-local species, the sequence and identifying code (1–25, with additional letters, if any, denoting variants) are given at the top, with the collated BOLD output and FinPROTAX outputs below. Debatable taxonomic assignments are explained at each species’ first mention (see cases 7, 18, 18, 22, and 25).

For the BOLD output, the full Top 100 matches are listed (from the Full Length Record Barcode database), with the number of identical matches (i.e. same species at same Match %) indicated in the first column (#). The best match is indicated in **bold**; if there are several equally likely matches (sometimes due to an alternative spelling/synonym of the name), all are highlighted this way. The nearest alternative identity that could realistically be found in our study area (if any are present in the Top 100) is underlined. To save space and aid comparison to the FinPROTAX data, the phylum is omitted (it is Arthropoda in all cases). Note that the full Top 100 is copied verbatim, meaning it will include some matches based on poor overlap, or records that are otherwise problematic. Typically, records with matching percentages of 99.31, 98.61, or 97.92 are trustworthy (representing 1, 2, or 3 bp difference and full overlap over the 145 bp queried sequence).

For the FinPROTAX output, subfamilies and tribes are left out, for better comparability to the BOLD data. Information matching the best match from the BOLD output is again presented in **bold** text, at all taxonomic levels. Taxonomic information matching the nearest alternative from the BOLD output (if any) is again underlined. When the best and nearest local match have overlapping data, the respective information is both **bold and underlined**.

For example, ASV 3d has a 100% match to one species in BOLD: *Biston stratarius*, alternatively known as *Biston strataria*. The former has 34 matches in the reference database, the latter 1; both are indicated in bold text. The same species has additional matches at 99.31, 98.61, and 97.92%. Below those, at 96.53%, is the first match to a species present in the study area: *Biston betularia* (synonym: *Biston betularius*), which is thus underlined. In the FinPROTAX table, all taxonomic levels from Insecta to the genus *Biston* are both bold and underlined (shared between the best and nearest local match), while the sole listed species is merely in bold, since it is the same taxonomic assignment given as the best match from the BOLD data.

1. *Agriopis marginaria*, Fabricius 1776

>1

AATATTAGGAACTTCTTTAAGATTATTAATCCGAGCAGAATTAGGTAATCCAGGGTCTTTAATTGGAGATGATCAAATTTATAACACTATTGTAACTGCCCATGCTTTTATTATAATTTTTTTTATAGTTATACCTATTATAATT

BOLD output

| # | Class | Order | Family | Genus | Species | Match % |
| --- | --- | --- | --- | --- | --- | --- |
| 38 | **Insecta** | **Lepidoptera** | **Geometridae** | ***Agriopis*** | ***marginaria*** | **100** |
| 29 | Insecta | Lepidoptera | Geometridae | *Agriopis* | *marginaria* | 99.31 |
| 4 | Insecta | Lepidoptera | Geometridae | *Agriopis* | *marginaria* | 98.61 |
| 1 | Insecta | Lepidoptera | Erebidae | *Eudoliche* | *sp.* | 97.22 |
| 1 | Insecta | Lepidoptera | Coleophoridae | *Coleophora* | *cornivorella* | 96.83 |
| 1 | Insecta | Lepidoptera | Lycaenidae | *Phengaris* | *nausithous* | 96.59 |
| 1 | Insecta | Lepidoptera | Noctuidae | *Spodoptera* | *frugiperda* | 96.55 |
| 1 | Insecta | Lepidoptera | Nymphalidae | *Bicyclus* | *rhacotis* | 96.55 |
| 1 | Insecta | Lepidoptera | Geometridae | *Anisoperas* | *tessellata* | 96.53 |
| 1 | Insecta | Lepidoptera | Sphingidae | *Platysphinx* | *phyllis* | 96.53 |
| 1 | Insecta | Lepidoptera | Geometridae | *Spartopteryx* | *kindermannaria* | 96.53 |
| 2 | Insecta | Lepidoptera | Geometridae | *Darisodes* | *oritropha* | 96.53 |
| 4 | Insecta | Lepidoptera | Geometridae | *Lycia* | *ypsilon* | 96.53 |
| 1 | Insecta | Lepidoptera | Tortricidae | *Ophiorrhabda* | *scaristis* | 96.53 |
| 4 | Insecta | Lepidoptera | Geometridae | *Chorodna* | *creataria* | 96.53 |
| 1 | Insecta | Lepidoptera | Euteliidae | *Eutelia* | *catephioidesGB01* | 96.53 |
| 1 | Insecta | Lepidoptera | Erebidae | *Eudoliche* | *sp.* | 96.53 |
| 4 | Insecta | Lepidoptera | Geometridae | *Exangerona* | *prattiaria* | 96.53 |
| 1 | Insecta | Lepidoptera | Geometridae | *Gongropteryx* | *moscata* | 96.53 |
| 3 | Insecta | Lepidoptera | Geometridae | *Arichanna* | *perimelaina* | 96.53 |

FinPROTAX output

|  | Class | Order | Family | Genus | Species | Probability |
| --- | --- | --- | --- | --- | --- | --- |
|  | **Insecta** |  |  |  |  | **0.999997** |
|  | **Insecta** | **Lepidoptera** |  |  |  | **0.996841** |
|  | **Insecta** | **Lepidoptera** | **Geometridae** |  |  | **0.996020** |
|  | **Insecta** | **Lepidoptera** | **Geometridae** | ***Agriopis*** |  | **0.866695** |
|  | **Insecta** | **Lepidoptera** | **Geometridae** | ***Agriopis*** | ***marginaria*** | **0.860811** |

2. *Bena bicolorana*, Fuessly 1775

>2a

AATAGTAGGAACTTCTTTAAGTCTTTTAATTCGAGCTGAATTAGGTAATCCAGGATCTTTAATTGGAGATGATCAAATTTATAATACTATTGTTACTGCTCATGCTTTTATTATAATTTTTTTTATAGTAATACCAATCATAATT

BOLD output

| # | Class | Order | Family | Genus | Species | Match % |
| --- | --- | --- | --- | --- | --- | --- |
| 1 | **Insecta** | **Lepidoptera** | **Nolidae** | ***Bena*** | ***bicolorana*** | **99.31** |
| 40 | Insecta | Lepidoptera | Nolidae | *Bena* | *bicolorana* | 98.61 |
| 1 | Insecta | Lepidoptera | Nolidae | *Blenina* | *chrysochlora* | 97.92 |
| 2 | Insecta | Lepidoptera | Nolidae | *Bena* | *bicolorana* | 97.92 |
| 1 | Insecta | Lepidoptera | Geometridae | *Chorodna* | *ugandaria* | 97.3 |
| 1 | Insecta | Lepidoptera | Erebidae | *Barsine* | *striata* | 97.22 |
| 4 | Insecta | Lepidoptera | Erebidae | *Callisthenia* | *variegata* | 97.22 |
| 4 | Insecta | Lepidoptera | Erebidae | *Miltochrista* | *fuscozonata* | 97.22 |
| 9 | Insecta | Lepidoptera | Erebidae | *Miltochrista* | *striata* | 97.22 |
| 5 | Insecta | Lepidoptera | Mimallonidae | *Psychocampa* | *manalca* | 97.22 |
| 3 | Insecta | Lepidoptera | Mimallonidae | *Cicinnus* | *sp.* | 97.22 |
| 11 | Insecta | Lepidoptera | Cosmopterigidae | *Macrobathra* | *aphristis* | 97.22 |
| 4 | Insecta | Lepidoptera | Cosmopterigidae | *Macrobathra* | *euryleuca* | 97.22 |
| 14 | Insecta | Lepidoptera | Noctuidae | *Feralia* | *major* | 97.22 |

FinPROTAX output

|  | Class | Order | Family | Genus | Species | Probability |
| --- | --- | --- | --- | --- | --- | --- |
|  | **Insecta** |  |  |  |  | **0.999996** |
|  | **Insecta** | **Lepidoptera** |  |  |  | **0.985702** |
|  | **Insecta** | **Lepidoptera** | **Nolidae** |  |  | **0.824110** |
|  | **Insecta** | **Lepidoptera** | **Nolidae** | ***Bena*** |  | **0.815912** |
|  | **Insecta** | **Lepidoptera** | **Nolidae** | ***Bena*** | ***bicolorana*** | **0.807889** |

>2b

AATAGTAGGAACTTCTTTAAGTCTTTTAATTCGAGCTGAATTAGGTAATCCAGGATCTTTAATTGGAGATGATCAAATTTATAATACTATTGTTACTGCTCATGCTTTTATTATAATTTTTTTTATAGTAATACCTATTATAATT

BOLD output

| # | Class | Order | Family | Genus | Species | Match % |
| --- | --- | --- | --- | --- | --- | --- |
| 40 | **Insecta** | **Lepidoptera** | **Nolidae** | ***Bena*** | ***bicolorana*** | **100** |
| 3 | Insecta | Lepidoptera | Nolidae | *Bena* | *bicolorana* | 99.31 |
| 1 | Insecta | Lepidoptera | Nolidae | *Blenina* | *lichenosa* | 98.61 |
| 1 | Insecta | Lepidoptera | Choreutidae | *Tortyra* | *cf. cuprinella* | 98.1 |
| 1 | Insecta | Lepidoptera | Hesperiidae | *Meza* | *mabea* | 98.1 |
| 1 | Insecta | Lepidoptera | Hesperiidae | *Aides* | *duma* | 98.1 |
| 13 | Insecta | Lepidoptera | Erebidae | *Rejectaria* | *funebris* | 97.92 |
| 12 | Insecta | Lepidoptera | Nolidae | *Acatapaustus* | *mesoleuca* | 97.92 |
| 4 | Insecta | Lepidoptera | Tineidae | *Micrerethista* | *entripta* | 97.92 |
| 1 | Insecta | Lepidoptera | Nolidae | *Nola* | *rwanda* | 97.92 |
| 1 | Insecta | Lepidoptera | Nolidae | *Meganola* | *melanographa* | 97.92 |
| 7 | Insecta | Lepidoptera | Erebidae | *Hormoschista* | *latipalpis* | 97.92 |
| 3 | Insecta | Lepidoptera | Gelechiidae | *Acompsia* | *tripunctella* | 97.92 |
| 1 | Insecta | Lepidoptera | Gelechiidae | *Acompsia* | *antirrhinella* | 97.92 |
| 1 | Insecta | Lepidoptera | Gelechiidae | *Acompsia* | *pyrenaella* | 97.92 |
| 1 | Insecta | Lepidoptera | Oecophoridae | *Cosmaresta* | *anarrecta* | 97.92 |
| 2 | Insecta | Lepidoptera | Oecophoridae | *Cosmaresta* | *niphias* | 97.92 |
| 1 | Insecta | Lepidoptera | Erebidae | *Ensipia* | *palpitatalis* | 97.92 |
| 1 | Insecta | Lepidoptera | Nolidae | *Blenina* | *chrysochlora* | 97.92 |
| 4 | Insecta | Lepidoptera | Nolidae | *Blenina* | *lichenosa* | 97.92 |
| 1 | Insecta | Lepidoptera | Xyloryctidae | *Lichenaula* | *sp. ANIC264* | 97.92 |

FinPROTAX output

|  | Class | Order | Family | Genus | Species | Probability |
| --- | --- | --- | --- | --- | --- | --- |
|  | **Insecta** |  |  |  |  | **0.999995** |
|  | **Insecta** | **Lepidoptera** |  |  |  | **0.987566** |
|  | Insecta | Lepidoptera | Noctuidae |  |  | 0.143102 |
|  | **Insecta** | **Lepidoptera** | **Nolidae** |  |  | **0.658215** |
|  | **Insecta** | **Lepidoptera** | **Nolidae** | ***Bena*** |  | **0.650470** |
|  | **Insecta** | **Lepidoptera** | **Nolidae** | ***Bena*** | ***bicolorana*** | **0.649712** |
|  | Insecta | Lepidoptera | Tortricidae |  |  | 0.118565 |

3. *Biston stratarius* (*Biston strataria*), Hufnagel 1767

>3a

AATAGTAGGAACATCTTTAAGTTTATTAATTCGAGCAGAATTAGGTAATCCTGGATCTTTAATTGGAGACGATCAAATTTATAACACTATTGTAACAGCTCATGCTTTTATTATAATCTTTTTCATAGTGATGCCAATTATAATT

BOLD output

| # | Class | Order | Family | Genus | Species | Match % |
| --- | --- | --- | --- | --- | --- | --- |
| 34 | **Insecta** | **Lepidoptera** | **Geometridae** | ***Biston*** | ***strataria*** | **99.31** |
| 1 | **Insecta** | **Lepidoptera** | **Geometridae** | ***Biston*** | ***stratarius*** | **99.31** |
| 12 | Insecta | Lepidoptera | Geometridae | *Biston* | *strataria* | 98.61 |
| 1 | Insecta | Lepidoptera | Geometridae | *Biston* | *stratarius* | 98.61 |
| 2 | Insecta | Lepidoptera | Geometridae | *Biston* | *strataria* | 97.92 |
| 1 | Insecta | Lepidoptera | Gelechiidae | *Aristotelia* | *iospora* | 97.32 |
| 5 | Insecta | Lepidoptera | Geometridae | *Biston* | *strataria* | 97.22 |
| 9 | Insecta | Lepidoptera | Gelechiidae | *Anarsia* | *spartiella* | 96.53 |
| 1 | Insecta | Lepidoptera | Scythrididae | *Scythris* | *trifurcella* | 95.83 |
| 2 | Insecta | Lepidoptera | Gelechiidae | *Anarsia* | *eleagnella* | 95.83 |
| 3 | Insecta | Lepidoptera | Geometridae | *Biston* | *rosenbaueri* | 95.83 |
| 1 | Insecta | Lepidoptera | Gelechiidae | *Chionodes* | *thyotes* | 95.83 |
| 1 | Insecta | Lepidoptera | Gelechiidae | *GelechiidaeGEN* | *sp. AAL8373* | 95.83 |
| 1 | Insecta | Lepidoptera | Scythrididae | *Neoscythris* | *JFL029* | 95.83 |
| 1 | Insecta | Lepidoptera | Geometridae | *Biston* | *betularia* | 95.83 |
| 1 | Insecta | Lepidoptera | Geometridae | *Hypomecis* | *intrusilinea* | 95.83 |
| 2 | Insecta | Lepidoptera | Gelechiidae | *Anarsia* | *spartiella* | 95.83 |
| 1 | Insecta | Lepidoptera | Gelechiidae | *Anarsia* | *sp. ANIC6* | 95.83 |
| 9 | Insecta | Lepidoptera | Gelechiidae | *Anarsia* | *bilbainella* | 95.83 |
| 1 | Insecta | Lepidoptera | Gelechiidae | *Anarsia* | *innoxiella* | 95.83 |
| 1 | Insecta | Lepidoptera | Geometridae | *Cyclophora* | *hyponoea* | 95.54 |
| 1 | Insecta | Lepidoptera | Euteliidae | *Paectes* | *roseovincta* | 95.14 |
| 4 | Insecta | Lepidoptera | Scythrididae | *Areniscythris* | *JFL291* | 95.14 |
| 1 | Insecta | Lepidoptera | Gelechiidae | *Anarsia* | *sp. 1* | 95.14 |
| 2 | Insecta | Lepidoptera | Noctuidae | *Helicoverpa* | *armigera* | 95.14 |
| 2 | Insecta | Lepidoptera | Gelechiidae | *Anarsia* | *guiera* | 95.14 |

FinPROTAX output

|  | Class | Order | Family | Genus | Species | Probability |
| --- | --- | --- | --- | --- | --- | --- |
|  | **Insecta** |  |  |  |  | **0.999997** |
|  | **Insecta** | **Lepidoptera** |  |  |  | **0.998918** |
|  | **Insecta** | **Lepidoptera** | **Geometridae** |  |  | **0.998124** |
|  | **Insecta** | **Lepidoptera** | **Geometridae** | ***Biston*** |  | **0.967347** |
|  | **Insecta** | **Lepidoptera** | **Geometridae** | ***Biston*** | ***stratarius*** | **0.960781** |

>3b

AATAGTGGGAACATCTTTAAGTTTATTAATTCGAGCAGAATTAGGTAATCCTGGATCTTTAATTGGAGACGATCAAATTTATAACACTATTGTAACAGCTCATGCTTTTATTATAATTTTTTTCATAGTGATGCCAATTATAATT

BOLD output

| # | Class | Order | Family | Genus | Species | Match % |
| --- | --- | --- | --- | --- | --- | --- |
| 34 | **Insecta** | **Lepidoptera** | **Geometridae** | ***Biston*** | ***strataria*** | **99.31** |
| 1 | **Insecta** | **Lepidoptera** | **Geometridae** | ***Biston*** | ***stratarius*** | **99.31** |
| 12 | Insecta | Lepidoptera | Geometridae | *Biston* | *strataria* | 98.61 |
| 1 | Insecta | Lepidoptera | Geometridae | *Biston* | *stratarius* | 98.61 |
| 2 | Insecta | Lepidoptera | Geometridae | *Biston* | *strataria* | 97.92 |
| 5 | Insecta | Lepidoptera | Geometridae | *Biston* | *strataria* | 97.22 |
| 1 | Insecta | Lepidoptera | Geometridae | *Hypomecis* | *intrusilinea* | 96.67 |
| 1 | Insecta | Lepidoptera | Geometridae | *Biston* | *betularia* | 96.53 |
| 9 | Insecta | Lepidoptera | Gelechiidae | *Anarsia* | *spartiella* | 96.53 |
| 1 | Insecta | Lepidoptera | Gelechiidae | *Aristotelia* | *iospora* | 96.43 |
| 11 | Insecta | Lepidoptera | Geometridae | *Biston* | *robustum* | 95.83 |
| 1 | Insecta | Lepidoptera | Scythrididae | *Scythris* | *trifurcella* | 95.83 |
| 2 | Insecta | Lepidoptera | Gelechiidae | *Anarsia* | *eleagnella* | 95.83 |
| 1 | Insecta | Lepidoptera | Geometridae | *Zeuctoboarmia* | *AH01* | 95.83 |
| 1 | Insecta | Lepidoptera | Gelechiidae | *gelBioLep01* | *BioLep2239* | 95.83 |
| 1 | Insecta | Lepidoptera | Geometridae | *Pero* | *hoedularia* | 95.83 |
| 1 | Insecta | Lepidoptera | Crambidae | *Eudonia* | *luteusalis* | 95.83 |
| 1 | Insecta | Lepidoptera | Gelechiidae | *Chionodes* | *thyotes* | 95.83 |
| 1 | Insecta | Lepidoptera | Gelechiidae | *GelechiidaeGEN* | *sp. AAL8373* | 95.83 |
| 1 | Insecta | Lepidoptera | Crambidae | *Argyria* | *BioLep453* | 95.83 |
| 1 | Insecta | Lepidoptera | Scythrididae | *Neoscythris* | *JFL029* | 95.83 |
| 11 | Insecta | Lepidoptera | Geometridae | *Biston* | *betularia* | 95.83 |

FinPROTAX output

|  | Class | Order | Family | Genus | Species | Probability |
| --- | --- | --- | --- | --- | --- | --- |
|  | **Insecta** |  |  |  |  | **0.999996** |
|  | **Insecta** | **Lepidoptera** |  |  |  | **0.998687** |
|  | **Insecta** | **Lepidoptera** | **Geometridae** |  |  | **0.997860** |
|  | **Insecta** | **Lepidoptera** | **Geometridae** | ***Biston*** |  | **0.947979** |
|  | **Insecta** | **Lepidoptera** | **Geometridae** | ***Biston*** | ***stratarius*** | **0.941544** |

>3c

AATAGTAGGAACATCTTTAAGTTTATTAATTCGAGCAGAATTAGGTAATCCTGGGTCTTTAATTGGAGACGATCAAATTTATAACACTATTGTAACAGCTCATGCTTTTATTATAATTTTTTTCATAGTGATGCCAATTATAATT

BOLD output

| # | Class | Order | Family | Genus | Species | Match % |
| --- | --- | --- | --- | --- | --- | --- |
| 34 | **Insecta** | **Lepidoptera** | **Geometridae** | ***Biston*** | ***strataria*** | **99.31** |
| 1 | **Insecta** | **Lepidoptera** | **Geometridae** | ***Biston*** | ***stratarius*** | **99.31** |
| 12 | Insecta | Lepidoptera | Geometridae | *Biston* | *strataria* | 98.61 |
| 1 | Insecta | Lepidoptera | Geometridae | *Biston* | *stratarius* | 98.61 |
| 2 | Insecta | Lepidoptera | Geometridae | *Biston* | *strataria* | 97.92 |
| 5 | Insecta | Lepidoptera | Geometridae | *Biston* | *strataria* | 97.22 |
| 9 | Insecta | Lepidoptera | Gelechiidae | *Anarsia* | *spartiella* | 96.53 |
| 1 | Insecta | Lepidoptera | Gelechiidae | *Aristotelia* | *iospora* | 96.43 |
| 1 | Insecta | Lepidoptera | Scythrididae | *Scythris* | *trifurcella* | 95.83 |
| 2 | Insecta | Lepidoptera | Gelechiidae | *Anarsia* | *eleagnella* | 95.83 |
| 1 | Insecta | Lepidoptera | Gelechiidae | *Chionodes* | *thyotes* | 95.83 |
| 1 | Insecta | Lepidoptera | Gelechiidae | *GelechiidaeGEN* | *sp. AAL8373* | 95.83 |
| 1 | Insecta | Lepidoptera | Scythrididae | *Neoscythris* | *JFL029* | 95.83 |
| 1 | Insecta | Lepidoptera | Geometridae | *Biston* | *betularia* | 95.83 |
| 1 | Insecta | Lepidoptera | Geometridae | *Hypomecis* | *intrusilinea* | 95.83 |
| 2 | Insecta | Lepidoptera | Gelechiidae | *Anarsia* | *spartiella* | 95.83 |
| 1 | Insecta | Lepidoptera | Gelechiidae | *Anarsia* | *sp. ANIC6* | 95.83 |
| 1 | Insecta | Lepidoptera | Gelechiidae | *gelBioLep01* | *BioLep472* | 95.83 |
| 9 | Insecta | Lepidoptera | Gelechiidae | *Anarsia* | *bilbainella* | 95.83 |
| 1 | Insecta | Lepidoptera | Gelechiidae | *Anarsia* | *innoxiella* | 95.83 |
| 1 | Insecta | Lepidoptera | Euteliidae | *Paectes* | *roseovincta* | 95.14 |
| 4 | Insecta | Lepidoptera | Scythrididae | *Areniscythris* | *JFL291* | 95.14 |
| 1 | Insecta | Lepidoptera | Gelechiidae | *Anarsia* | *sp. 1* | 95.14 |
| 2 | Insecta | Lepidoptera | Noctuidae | *Helicoverpa* | *armigera* | 95.14 |
| 2 | Insecta | Lepidoptera | Gelechiidae | *Anarsia* | *guiera* | 95.14 |
| 1 | Insecta | Lepidoptera | Geometridae | *Visiana* | *hyperctenista* | 95.14 |
| 1 | Insecta | Lepidoptera | Geometridae | *Trimetopia* | *aetheraria* | 95.14 |
| 1 | Insecta | Lepidoptera | Geometridae | *Biston* | *robustum* | 95.14 |

FinPROTAX output

|  | Class | Order | Family | Genus | Species | Probability |
| --- | --- | --- | --- | --- | --- | --- |
|  | **Insecta** |  |  |  |  | **0.999996** |
|  | **Insecta** | **Lepidoptera** |  |  |  | **0.998916** |
|  | **Insecta** | **Lepidoptera** | **Geometridae** |  |  | **0.997928** |
|  | **Insecta** | **Lepidoptera** | **Geometridae** | ***Biston*** |  | **0.956416** |
|  | **Insecta** | **Lepidoptera** | **Geometridae** | ***Biston*** | ***stratarius*** | **0.949924** |

>3d

AATAGTAGGAACATCTTTAAGTTTATTAATTCGAGCAGAATTAGGTAATCCTGGATCTTTAATTGGAGACGATCAAATTTATAACACTATTGTAACAGCTCATGCTTTTATTATAATTTTTTTCATAGTGATGCCAATTATAATT

BOLD output

| # | Class | Order | Family | Genus | Species | Match % |
| --- | --- | --- | --- | --- | --- | --- |
| 34 | **Insecta** | **Lepidoptera** | **Geometridae** | ***Biston*** | ***strataria*** | **100** |
| 1 | **Insecta** | **Lepidoptera** | **Geometridae** | ***Biston*** | ***stratarius*** | **100** |
| 12 | Insecta | Lepidoptera | Geometridae | *Biston* | *strataria* | 99.31 |
| 1 | Insecta | Lepidoptera | Geometridae | *Biston* | *stratarius* | 99.31 |
| 2 | Insecta | Lepidoptera | Geometridae | *Biston* | *strataria* | 98.61 |
| 5 | Insecta | Lepidoptera | Geometridae | *Biston* | *strataria* | 97.92 |
| 1 | Insecta | Lepidoptera | Gelechiidae | *Aristotelia* | *iospora* | 97.32 |
| 9 | Insecta | Lepidoptera | Gelechiidae | *Anarsia* | *spartiella* | 97.22 |
| 1 | Insecta | Lepidoptera | Geometridae | *Hypomecis* | *intrusilinea* | 96.67 |
| 1 | Insecta | Lepidoptera | Scythrididae | *Scythris* | *trifurcella* | 96.53 |
| 2 | Insecta | Lepidoptera | Gelechiidae | *Anarsia* | *eleagnella* | 96.53 |
| 1 | Insecta | Lepidoptera | Gelechiidae | *Chionodes* | *thyotes* | 96.53 |
| 1 | Insecta | Lepidoptera | Gelechiidae | *GelechiidaeGEN* | *sp. AAL8373* | 96.53 |
| 1 | Insecta | Lepidoptera | Scythrididae | *Neoscythris* | *JFL029* | 96.53 |
| 1 | Insecta | Lepidoptera | Geometridae | *Biston* | *betularia* | 96.53 |
| 2 | Insecta | Lepidoptera | Gelechiidae | *Anarsia* | *spartiella* | 96.53 |
| 1 | Insecta | Lepidoptera | Gelechiidae | *Anarsia* | *sp. ANIC6* | 96.53 |
| 9 | Insecta | Lepidoptera | Gelechiidae | *Anarsia* | *bilbainella* | 96.53 |
| 1 | Insecta | Lepidoptera | Gelechiidae | *Anarsia* | *innoxiella* | 96.53 |
| 1 | Insecta | Lepidoptera | Euteliidae | *Paectes* | *roseovincta* | 95.83 |
| 4 | Insecta | Lepidoptera | Scythrididae | *Areniscythris* | *JFL291* | 95.83 |
| 1 | Insecta | Lepidoptera | Gelechiidae | *Anarsia* | *sp. 1* | 95.83 |
| 2 | Insecta | Lepidoptera | Noctuidae | *Helicoverpa* | *armigera* | 95.83 |
| 2 | Insecta | Lepidoptera | Gelechiidae | *Anarsia* | *guiera* | 95.83 |
| 1 | Insecta | Lepidoptera | Geometridae | *Visiana* | *hyperctenista* | 95.83 |
| 3 | Insecta | Lepidoptera | Geometridae | *Biston* | *robustum* | 95.83 |

FinPROTAX output

|  | Class | Order | Family | Genus | Species | Probability |
| --- | --- | --- | --- | --- | --- | --- |
|  | **Insecta** |  |  |  |  | **0.999998** |
|  | **Insecta** | **Lepidoptera** |  |  |  | **0.998919** |
|  | **Insecta** | **Lepidoptera** | **Geometridae** |  |  | **0.998125** |
|  | **Insecta** | **Lepidoptera** | **Geometridae** | ***Biston*** |  | **0.967416** |
|  | **Insecta** | **Lepidoptera** | **Geometridae** | ***Biston*** | ***stratarius*** | **0.966289** |

4. *Campaea honoraria* (*Gerinia honoraria*), Denis & Schiffermüller 1775

>4a

AATAGTAGGAACTTCATTAAGTTTATTAATTCGAGCTGAATTAGGAAACCCAGGATCTCTAATTGGAGATGACCAAATTTATAATACAATTGTAACTGCCCATGCTTTTATTATAATCTTTTTCATAGTAATACCAATTATAATT

BOLD output

| # | Class | Order | Family | Genus | Species | Match % |
| --- | --- | --- | --- | --- | --- | --- |
| 17 | **Insecta** | **Lepidoptera** | **Geometridae** | ***Gerinia*** | ***honoraria*** | **100** |
| 5 | Insecta | Lepidoptera | Geometridae | *Gerinia* | *honoraria* | 99.31 |
| 1 | Insecta | Lepidoptera | Palaephatidae | *Ptyssoptera* | *sp. ANIC2* | 96.53 |
| 4 | Insecta | Lepidoptera | Tortricidae | *Gypsonoma* | *scolopiae* | 96.53 |
| 16 | Insecta | Lepidoptera | Erebidae | *Mocis* | *mayeri* | 95.83 |
| 1 | Insecta | Lepidoptera | Noctuidae | *Acontia* | *marmoralis* | 95.83 |
| 1 | Insecta | Lepidoptera | Palaephatidae | *Ptyssoptera* | *sp. ANIC2* | 95.83 |
| 2 | Insecta | Lepidoptera | Tortricidae | *Gypsonoma* | *scolopiae* | 95.83 |
| 1 | Insecta | Lepidoptera | Notodontidae | *Furcula* | *occidentalis* | 95.83 |
| 3 | Insecta | Lepidoptera | Notodontidae | *Furcula* | *furcula* | 95.83 |
| 1 | Insecta | Lepidoptera | Erebidae | *Hypotacha* | *watusi* | 95.83 |
| 1 | Insecta | Lepidoptera | Notodontidae | *Neocerura* | *liturata* | 95.83 |
| 1 | Insecta | Lepidoptera | Coleophoridae | *Coleophora* | *cornivorella* | 95.24 |
| 3 | Insecta | Lepidoptera | Noctuidae | *Acontia* | *elaeoa* | 95.14 |
| 1 | Insecta | Lepidoptera | Bombycidae | *Quentalia* | *ephonia* | 95.14 |
| 37 | Insecta | Lepidoptera | Notodontidae | *Furcula* | *furcula* | 95.14 |
| 2 | Insecta | Lepidoptera | Tineidae | *Edosa* | *xystidophora* | 95.14 |
| 2 | Insecta | Lepidoptera | Tineidae | *Edosa* | *sp. 7* | 95.14 |
| 1 | Insecta | Lepidoptera | Noctuidae | *Acontia* | *marmoralis* | 95.14 |

FinPROTAX output

|  | Class | Order | Family | Genus | Species | Probability |
| --- | --- | --- | --- | --- | --- | --- |
|  | **Insecta** |  |  |  |  | **0.999985** |
|  | Insecta | Diptera |  |  |  | 0.115020 |
|  | **Insecta** | **Lepidoptera** |  |  |  | **0.787084** |
|  | Insecta | Lepidoptera | Noctuidae |  |  | 0.112737 |
|  | Insecta | Lepidoptera | Notodontidae |  |  | 0.343374 |
|  | Insecta | Lepidoptera | Notodontidae | *Furcula* |  | 0.300126 |
|  | Insecta | Lepidoptera | Notodontidae | *Furcula* | unk | 0.298855 |

>4b

AATAGTAGGAACTTCATTAAGTTTATTAATTCGAGCTGAATTAGGAAACCCAGGATCTCTAATTGGAGATGACCAAATTTATAATACAATTGTAACTGCCCACGCTTTTATTATAATCTTTTTCATAGTAATACCAATTATAATT

BOLD output

| # | Class | Order | Family | Genus | Species | Match % |
| --- | --- | --- | --- | --- | --- | --- |
| 17 | **Insecta** | **Lepidoptera** | **Geometridae** | ***Gerinia*** | ***honoraria*** | **99.31** |
| 5 | Insecta | Lepidoptera | Geometridae | *Gerinia* | *honoraria* | 98.61 |
| 1 | Insecta | Lepidoptera | Erebidae | *Hypotacha* | *watusi* | 96.53 |
| 1 | Insecta | Lepidoptera | Palaephatidae | *Ptyssoptera* | *sp. ANIC2* | 95.83 |
| 4 | Insecta | Lepidoptera | Tortricidae | *Gypsonoma* | *scolopiae* | 95.83 |
| 9 | Insecta | Lepidoptera | Notodontidae | *Furcula* | *occidentalis* | 95.83 |
| 16 | Insecta | Lepidoptera | Erebidae | *Mocis* | *mayeri* | 95.14 |
| 1 | Insecta | Lepidoptera | Noctuidae | *Acontia* | *marmoralis* | 95.14 |
| 1 | Insecta | Lepidoptera | Palaephatidae | *Ptyssoptera* | *sp. ANIC2* | 95.14 |
| 2 | Insecta | Lepidoptera | Tortricidae | *Gypsonoma* | *scolopiae* | 95.14 |
| 1 | Insecta | Lepidoptera | Notodontidae | *Furcula* | *occidentalis* | 95.14 |
| 3 | Insecta | Lepidoptera | Geometridae | *Eurranthis* | *plummistaria* | 95.14 |
| 3 | Insecta | Lepidoptera | Notodontidae | *Furcula* | *furcula* | 95.14 |
| 4 | Insecta | Lepidoptera | Erebidae | *Audea* | *watusi* | 95.14 |
| 3 | Insecta | Lepidoptera | Geometridae | *Alcis* | *irrufata* | 95.14 |
| 1 | Insecta | Lepidoptera | Notodontidae | *Neocerura* | *liturata* | 95.14 |
| 1 | Insecta | Lepidoptera | Geometridae | *Metallolophia* | *inanularia* | 94.93 |
| 3 | Insecta | Lepidoptera | Noctuidae | *Acontia* | *elaeoa* | 94.44 |
| 2 | Insecta | Lepidoptera | Bombycidae | *Quentalia* | *ephonia* | 94.44 |
| 22 | Insecta | Lepidoptera | Notodontidae | *Furcula* | *furcula* | 94.44 |

FinPROTAX output

|  | Class | Order | Family | Genus | Species | Probability |
| --- | --- | --- | --- | --- | --- | --- |
|  | **Insecta** |  |  |  |  | **0.999978** |
|  | Insecta | Diptera |  |  |  | 0.115012 |
|  | **Insecta** | **Lepidoptera** |  |  |  | **0.787030** |
|  | Insecta | Lepidoptera | Notodontidae |  |  | 0.266239 |
|  | Insecta | Lepidoptera | Notodontidae | *Furcula* |  | 0.232493 |
|  | Insecta | Lepidoptera | Notodontidae | *Furcula* | unk | 0.232324 |
|  | Insecta | Lepidoptera | Tortricidae |  |  | 0.243616 |

>4c

GAATAGTAGGAACTTCATTAAGTTTATTAATTCGAGCTGAATTAGGAAACCCAGGATCTCTAATTGGAGATGACCAAATTTATAATAAAATTGTAACTGCCCATGCTTTTATTATAATCTTTTCATAGTAATACCAATTATAATT

BOLD output

| # | Class | Order | Family | Genus | Species | Match % |
| --- | --- | --- | --- | --- | --- | --- |
| 17 | **Insecta** | **Lepidoptera** | **Geometridae** | ***Gerinia*** | ***honoraria*** | **99.3** |
| 5 | Insecta | Lepidoptera | Geometridae | *Gerinia* | *honoraria* | 98.6 |
| 1 | Insecta | Lepidoptera | Palaephatidae | *Ptyssoptera* | *sp. ANIC2* | 95.8 |
| 4 | Insecta | Lepidoptera | Tortricidae | *Gypsonoma* | *scolopiae* | 95.8 |
| 16 | Insecta | Lepidoptera | Erebidae | *Mocis* | *mayeri* | 95.1 |
| 1 | Insecta | Lepidoptera | Noctuidae | *Acontia* | *marmoralis* | 95.1 |
| 1 | Insecta | Lepidoptera | Palaephatidae | *Ptyssoptera* | *sp. ANIC2* | 95.1 |
| 2 | Insecta | Lepidoptera | Tortricidae | *Gypsonoma* | *scolopiae* | 95.1 |
| 1 | Insecta | Lepidoptera | Notodontidae | *Furcula* | *occidentalis* | 95.1 |
| 3 | Insecta | Lepidoptera | Notodontidae | *Furcula* | *furcula* | 95.1 |
| 1 | Insecta | Lepidoptera | Erebidae | *Hypotacha* | *watusi* | 95.1 |
| 1 | Insecta | Lepidoptera | Notodontidae | *Neocerura* | *liturata* | 95.1 |
| 3 | Insecta | Lepidoptera | Noctuidae | *Acontia* | *elaeoa* | 94.41 |
| 1 | Insecta | Lepidoptera | Bombycidae | *Quentalia* | *ephonia* | 94.41 |
| 37 | Insecta | Lepidoptera | Notodontidae | *Furcula* | *furcula* | 94.41 |
| 2 | Insecta | Lepidoptera | Tineidae | *Edosa* | *xystidophora* | 94.41 |
| 2 | Insecta | Lepidoptera | Tineidae | *Edosa* | *sp. 7* | 94.41 |
| 2 | Insecta | Lepidoptera | Noctuidae | *Acontia* | *marmoralis* | 94.41 |

FinPROTAX output

|  | Class | Order | Family | Genus | Species | Probability |
| --- | --- | --- | --- | --- | --- | --- |
|  | **Insecta** |  |  |  |  | **0.999982** |
|  | Insecta | Coleoptera |  |  |  | 0.153288 |
|  | Insecta | Diptera |  |  |  | 0.182035 |
|  | **Insecta** | **Lepidoptera** |  |  |  | **0.663860** |
|  | Insecta | Lepidoptera | Notodontidae |  |  | 0.289187 |
|  | Insecta | Lepidoptera | Notodontidae | *Furcula* |  | 0.254414 |
|  | Insecta | Lepidoptera | Notodontidae | *Furcula* | unk | 0.254211 |

5. *Catephia alchymista*, Denis & Schiffermüller 1775

>5

AATAGTAGGAACATCTCTAAGTCTTTTAATTCGTGCTGAATTAGGAAATCCTGGTTCATTAATTGGAGATGATCAAATTTATAATACTATTGTTACTGCCCATGCCTTTATTATAATTTTTTTTATAGTTATACCTATTATAATT

BOLD output

| # | Class | Order | Family | Genus | Species | Match % |
| --- | --- | --- | --- | --- | --- | --- |
| 16 | **Insecta** | **Lepidoptera** | **Erebidae** | ***Catephia*** | ***alchymista*** | **100** |
| 6 | Insecta | Lepidoptera | Erebidae | *Catephia* | *alchymista* | 99.31 |
| 1 | Insecta | Lepidoptera | Geometridae | *Racotis* | *boarmiaria* | 98.81 |
| 1 | Insecta | Lepidoptera | Lycaenidae | *Polyommatus* | *dizinensis* | 97.53 |
| 1 | Insecta | Lepidoptera | Noctuidae | *Pseudobryomima* | *sp. KLKDNA0340* | 97.53 |
| 1 | Insecta | Lepidoptera | Crambidae | *Chrysoteuchia* | *culmella* | 96.7 |
| 1 | Insecta | Lepidoptera | Papilionidae | *Pachliopta* | *aristolochiae* | 96.55 |
| 1 | Insecta | Lepidoptera | Nymphalidae | *Oeneis* | *sculda* | 96.55 |
| 1 | Insecta | Lepidoptera | Nymphalidae | *Heliconius* | *ethilla* | 96.3 |
| 3 | Insecta | Lepidoptera | Nymphalidae | *Heliconius* | *melpomene* | 96.3 |
| 1 | Insecta | Lepidoptera | Blastobasidae | *Blastobasis* | *sprotundalis* | 96.3 |
| 1 | Insecta | Lepidoptera | Oecophoridae | *Deuterogonia* | *pudorina* | 96.3 |
| 1 | Insecta | Lepidoptera | Nymphalidae | *Pierella* | *lena* | 96.3 |
| 1 | Insecta | Lepidoptera | Nymphalidae | *Heliconius* | *ethilla x Heliconius melpomene* | 96.25 |
| 1 | Insecta | Lepidoptera | Nymphalidae | *Melinaea* | *menophilus* | 96.25 |
| 1 | Insecta | Lepidoptera | Nymphalidae | *Heliconius* | *ethilla* | 96.2 |
| 1 | Insecta | Lepidoptera | Hesperiidae | *Ocybadistes* | *walkeri* | 96.19 |
| 1 | Insecta | Lepidoptera | Hesperiidae | *Carrhenes* | *sp. UK48* | 96.19 |
| 1 | Insecta | Lepidoptera | Nymphalidae | *Anthanassa* | *drusilla* | 96.15 |
| 1 | Insecta | Lepidoptera | Crambidae | *Bocchoris* | *cf. inspersalis* | 95.96 |
| 3 | Insecta | Lepidoptera | Crambidae | *Bocchoris* | *inspersalis* | 95.83 |
| 4 | Insecta | Lepidoptera | Erebidae | *Crypsiprora* | *EF01* | 95.83 |
| 2 | Insecta | Lepidoptera | Crambidae | *Hednota* | *cyclosema* | 95.83 |
| 1 | Insecta | Lepidoptera | Noctuidae | *Stictoptera* | *conturbata* | 95.83 |
| 1 | Insecta | Lepidoptera | Autostichidae | *Anaptilora* | *homoclera* | 95.83 |
| 2 | Insecta | Lepidoptera | Nolidae | *Nola* | *afrotaeniata* | 95.83 |
| 1 | Insecta | Lepidoptera | Gelechiidae | *Pseudotelphusa* | *quercinigracella* | 95.83 |
| 2 | Insecta | Lepidoptera | Gelechiidae | *Gelechia* | *mediterranea* | 95.83 |
| 7 | Insecta | Lepidoptera | Nolidae | *Nola* | *politzari* | 95.83 |
| 1 | Insecta | Lepidoptera | Depressariidae | *Ethmia* | *baliostola* | 95.83 |
| 2 | Insecta | Lepidoptera | Erebidae | *Arugisa* | *latiorella* | 95.83 |
| 1 | Insecta | Lepidoptera | Oecophoridae | *Epicurica* | *sp. ANIC2* | 95.83 |
| 2 | Insecta | Lepidoptera | Oecophoridae | *Euchaetis* | *sp. ANIC29* | 95.83 |
| 1 | Insecta | Lepidoptera | Autostichidae | *Anaptilora* | *ephelotis* | 95.83 |
| 3 | Insecta | Lepidoptera | Erebidae | *Phobolosia* | *Poole01* | 95.83 |
| 1 | Insecta | Coleoptera | Cerambycidae | *Tetraopes* | *texanus* | 95.7 |
| 15 | Insecta | Lepidoptera | Nymphalidae | *Heliconius* | *melpomene* | 95.7 |
| 4 | Insecta | Lepidoptera | Nymphalidae | *Heliconius* | *numata* | 95.7 |
| 5 | Insecta | Lepidoptera | Nymphalidae | *Heliconius* | *timareta* | 95.7 |

FinPROTAX output

|  | Class | Order | Family | Genus | Species | Probability |
| --- | --- | --- | --- | --- | --- | --- |
|  | **Insecta** |  |  |  |  | **0.999947** |
|  | **Insecta** | **Lepidoptera** |  |  |  | **0.911566** |
|  | Insecta | Lepidoptera | Coleophoridae |  |  | 0.181929 |
|  | Insecta | Lepidoptera | Coleophoridae | *Coleophora* |  | 0.181921 |
|  | Insecta | Lepidoptera | Coleophoridae | *Coleophora* | unk | 0.181853 |
|  | Insecta | Lepidoptera | Gelechiidae |  |  | 0.108630 |
|  | Insecta | Lepidoptera | Noctuidae |  |  | 0.313101 |

6. *Catocala conjuncta* (*Catocala coniuncta*), Esper 1787

>6a

AGTGGTAGGAACCTCATTAAGATTATTAATTCGAGCTGAATTAGGTAATCCAGGATCTTTAATTGGCGATGATCAAATTTACAATACTATTGTAACAGCTCATGCTTTTATTATAATTTTTTTTATAGTTATGCCAATTATAATT

BOLD output

| # | Class | Order | Family | Genus | Species | Match % |
| --- | --- | --- | --- | --- | --- | --- |
| 7 | **Insecta** | **Lepidoptera** | **Erebidae** | ***Catocala*** | ***conjuncta*** | **100** |
| 1 | **Insecta** | **Lepidoptera** | **Erebidae** | ***Catocala*** | ***coniuncta*** | **100** |
| 4 | Insecta | Lepidoptera | Erebidae | *Catocala* | *conjuncta* | 98.58 |
| 1 | Insecta | Lepidoptera | Hesperiidae | *Justinia* | *sp. UK57* | 98.1 |
| 1 | Insecta | Lepidoptera | Nymphalidae | *Oleria* | *gunilla* | 97.8 |
| 1 | Insecta | Lepidoptera | Erebidae | *Catocala* | *yuanna* | 97.16 |
| 14 | Insecta | Lepidoptera | Crambidae | *Notarcha* | *quaternalis* | 97.16 |
| 1 | Insecta | Lepidoptera | Noctuidae | *Spodoptera* | *frugiperda sp. 1* | 96.88 |
| 1 | Insecta | Lepidoptera | Erebidae | *Calyptra* | *lata* | 96.83 |
| 1 | Insecta | Lepidoptera | Noctuidae | *Helicoverpa* | *zea* | 96.77 |
| 8 | Insecta | Lepidoptera | Nymphalidae | *Oleria* | *gunilla* | 96.77 |
| 3 | Insecta | Lepidoptera | Nymphalidae | *Pseudoscada* | *florula* | 96.77 |
| 1 | Insecta | Lepidoptera | Nymphalidae | *Oleria* | *gunilla* | 96.67 |
| 3 | Insecta | Lepidoptera | Erebidae | *Catocala* | *crataegi sp. 2-RJB* | 96.45 |
| 26 | Insecta | Lepidoptera | Noctuidae | *Hecatera* | *bicolorata* | 96.45 |
| 1 | Insecta | Lepidoptera | Erebidae | *Catocala* | *promissa* | 96.45 |
| 3 | Insecta | Lepidoptera | Erebidae | *Leucanopsis* | *sp. 3* | 96.45 |
| 1 | Insecta | Lepidoptera | Geometridae | *Macaria* | *artesiaria* | 96.45 |
| 2 | Insecta | Lepidoptera | Erebidae | *Catocala* | *eutychea* | 96.45 |
| 3 | Insecta | Lepidoptera | Limacodidae | *Acharia* | *horrida* | 96.45 |
| 1 | Insecta | Lepidoptera | Erebidae | *Eucereon* | *amadis* | 96.45 |
| 1 | Insecta | Lepidoptera | Erebidae | *Melese* | *incertus* | 96.45 |
| 9 | Insecta | Lepidoptera | Erebidae | *Trichromia* | *atta* | 96.45 |
| 1 | Insecta | Lepidoptera | Crambidae | *Pantographa* | *limata* | 96.45 |
| 1 | Insecta | Lepidoptera | Erebidae | *Catocala* | *largeteaui* | 96.45 |
| 4 | Insecta | Lepidoptera | Erebidae | *Catocala* | *andromedae* | 96.45 |

FinPROTAX output

|  | Class | Order | Family | Genus | Species | Probability |
| --- | --- | --- | --- | --- | --- | --- |
|  | **Insecta** |  |  |  |  | **0.999976** |
|  | **Insecta** | **Lepidoptera** |  |  |  | **0.937018** |
|  | Insecta | Lepidoptera | Crambidae |  |  | 0.155881 |
|  | Insecta | Lepidoptera | Crambidae | unk |  | 0.117531 |
|  | Insecta | Lepidoptera | Noctuidae |  |  | 0.524894 |
|  | Insecta | Lepidoptera | Tortricidae |  |  | 0.146607 |

>6b

AATGGTAGGAACCTCATTAAGATTATTAATTCGAGCTGAATTAGGTAATCCGGGATCTTTAATTGGCGATGATCAAATTTACAATACTATTGTAACAGCTCATGCTTTTATTATAATTTTTTTTATAGTTATACCAATTATAATT

BOLD output

| # | Class | Order | Family | Genus | Species | Match % |
| --- | --- | --- | --- | --- | --- | --- |
| 2 | **Insecta** | **Lepidoptera** | **Erebidae** | ***Catocala*** | ***conjuncta*** | **100** |
| 1 | Insecta | Lepidoptera | Nymphalidae | *Oleria* | *gunilla* | 98.9 |
| 9 | Insecta | Lepidoptera | Erebidae | *Catocala* | *conjuncta* | 98.61 |
| 1 | Insecta | Lepidoptera | Erebidae | *Catocala* | *coniuncta* | 98.61 |
| 1 | Insecta | Lepidoptera | Hesperiidae | *Justinia* | *sp. UK57* | 98.1 |
| 1 | Insecta | Lepidoptera | Hesperiidae | *Pompeius* | *pompeius* | 98.1 |
| 1 | Insecta | Lepidoptera | Noctuidae | *Helicoverpa* | *zea* | 97.85 |
| 8 | Insecta | Lepidoptera | Nymphalidae | *Oleria* | *gunilla* | 97.85 |
| 3 | Insecta | Lepidoptera | Nymphalidae | *Pseudoscada* | *florula* | 97.85 |
| 1 | Insecta | Lepidoptera | Nymphalidae | *Oleria* | *gunilla* | 97.78 |
| 1 | Insecta | Lepidoptera | Nymphalidae | *Paralasa* | *styx* | 97.44 |
| 1 | Insecta | Lepidoptera | Papilionidae | *Papilio* | *lorquinianus ssp CBGPFLC_00061* | 97.44 |
| 1 | Insecta | Lepidoptera | Hesperiidae | *Cabirus* | *procas* | 97.14 |
| 1 | Insecta | Lepidoptera | Noctuidae | *Helicoverpa* | *gelotopoeon* | 96.77 |
| 4 | Insecta | Lepidoptera | Noctuidae | *Spodoptera* | *dolichos* | 96.77 |
| 1 | Insecta | Lepidoptera | Nymphalidae | *Brevioleria* | *aelia* | 96.77 |
| 2 | Insecta | Lepidoptera | Nymphalidae | *Brevioleria* | *arzalia* | 96.77 |
| 1 | Insecta | Lepidoptera | Nymphalidae | *Napeogenes* | *sylphis* | 96.77 |
| 2 | Insecta | Lepidoptera | Noctuidae | *Spodoptera* | *pulchella* | 96.77 |
| 2 | Insecta | Lepidoptera | Nymphalidae | *Oleria* | *onega* | 96.77 |
| 1 | Insecta | Lepidoptera | Nymphalidae | *Brevioleria* | *arzalia* | 96.67 |
| 1 | Insecta | Lepidoptera | Nymphalidae | *Hyposcada* | *kena* | 96.67 |
| 1 | Insecta | Lepidoptera | Nymphalidae | *Napeogenes* | *sylphis* | 96.67 |
| 1 | Insecta | Lepidoptera | Nymphalidae | *Napeogenes* | *pharo* | 96.63 |
| 1 | Insecta | Lepidoptera | Nymphalidae | *Brevioleria* | *arzalia* | 96.55 |
| 1 | Insecta | Lepidoptera | Noctuidae | *Spodoptera* | *frugiperda* | 96.55 |
| 3 | Insecta | Lepidoptera | Erebidae | *Catocala* | *crataegi sp. 2-RJB* | 96.53 |
| 3 | Insecta | Lepidoptera | Erebidae | *Leucanopsis* | *sp. 3* | 96.53 |
| 1 | Insecta | Lepidoptera | Erebidae | *Catocala* | *yuanna* | 96.53 |
| 5 | Insecta | Lepidoptera | Erebidae | *Leucanopsis* | *martona* | 96.53 |
| 1 | Insecta | Lepidoptera | Erebidae | *Spilosoma* | *lubricipeda* | 96.53 |
| 1 | Insecta | Lepidoptera | Erebidae | *Catocala* | *chelidonia* | 96.53 |
| 17 | Insecta | Lepidoptera | Erebidae | *Catocala* | *aestivalia* | 96.53 |
| 1 | Insecta | Lepidoptera | Erebidae | *Leucanopsis* | *valentina* | 96.53 |
| 1 | Insecta | Lepidoptera | Erebidae | *Catocala* | *crataegi RJB* | 96.53 |
| 5 | Insecta | Lepidoptera | Erebidae | *Leucanopsis* | *aurantiaca* | 96.53 |
| 12 | Insecta | Lepidoptera | Erebidae | *Leucanopsis* | *stipulata* | 96.53 |

FinPROTAX output

|  | Class | Order | Family | Genus | Species | Probability |
| --- | --- | --- | --- | --- | --- | --- |
|  | **Insecta** |  |  |  |  | **0.999982** |
|  | **Insecta** | **Lepidoptera** |  |  |  | **0.902457** |
|  | Insecta | Lepidoptera | Crambidae |  |  | 0.102169 |
|  | Insecta | Lepidoptera | Gelechiidae |  |  | 0.155817 |
|  | Insecta | Lepidoptera | Noctuidae |  |  | 0.344029 |

>6c

AATGGTAGGAACCTCATTAAGATTATTAATTCGAGCTGAATTAGGTAATCCAGGATCTTTAATTGGCGATGATCAAATTTACAATACTATTGTAACAGCTCATGCTTTTATTATAATTTTTTTTATAGTTATGCCAATTATAATT

BOLD output

| # | Class | Order | Family | Genus | Species | Match % |
| --- | --- | --- | --- | --- | --- | --- |
| 7 | **Insecta** | **Lepidoptera** | **Erebidae** | ***Catocala*** | ***conjuncta*** | **100** |
| 1 | Insecta | Lepidoptera | Erebidae | *Catocala* | *coniuncta* | 100 |
| 4 | Insecta | Lepidoptera | Erebidae | *Catocala* | *conjuncta* | 98.61 |
| 1 | Insecta | Lepidoptera | Hesperiidae | *Justinia* | *sp. UK57* | 98.1 |
| 1 | Insecta | Lepidoptera | Nymphalidae | *Oleria* | *gunilla* | 97.8 |
| 1 | Insecta | Lepidoptera | Noctuidae | *Spodoptera* | *frugiperda sp. 1* | 96.88 |
| 1 | Insecta | Lepidoptera | Noctuidae | *Helicoverpa* | *zea* | 96.77 |
| 8 | Insecta | Lepidoptera | Nymphalidae | *Oleria* | *gunilla* | 96.77 |
| 3 | Insecta | Lepidoptera | Nymphalidae | *Pseudoscada* | *florula* | 96.77 |
| 1 | Insecta | Lepidoptera | Nymphalidae | *Oleria* | *gunilla* | 96.67 |
| 1 | Insecta | Lepidoptera | Erebidae | *Catocala* | *yuanna* | 96.53 |
| 14 | Insecta | Lepidoptera | Crambidae | *Notarcha* | *quaternalis* | 96.53 |
| 1 | Insecta | Lepidoptera | Geometridae | *Chorodna* | *ugandaria* | 96.4 |
| 1 | Insecta | Lepidoptera | Hesperiidae | *Pompeius* | *pompeius* | 96.19 |
| 1 | Insecta | Lepidoptera | Hesperiidae | *Cabirus* | *procas* | 96.19 |
| 1 | Insecta | Lepidoptera | Nymphalidae | *Paralasa* | *styx* | 96.15 |
| 1 | Insecta | Lepidoptera | Papilionidae | *Papilio* | *lorquinianus ssp CBGPFLC_00061* | 96.15 |
| 1 | Insecta | Lepidoptera | Geometridae | *Chloroclydon* | *rinodaria* | 96.03 |
| 3 | Insecta | Lepidoptera | Erebidae | *Catocala* | *crataegi sp. 2-RJB* | 95.83 |
| 26 | Insecta | Lepidoptera | Noctuidae | *Hecatera* | *bicolorata* | 95.83 |
| 1 | Insecta | Lepidoptera | Erebidae | *Catocala* | *promissa* | 95.83 |
| 1 | Insecta | Lepidoptera | Geometridae | *Alsophila* | *japonensis* | 95.83 |
| 9 | Insecta | Lepidoptera | Noctuidae | *Spodoptera* | *frugiperda sp. 1* | 95.83 |
| 7 | Insecta | Lepidoptera | Noctuidae | *Spodoptera* | *frugiperda sp. 2* | 95.83 |
| 3 | Insecta | Lepidoptera | Erebidae | *Leucanopsis* | *sp. 3* | 95.83 |
| 1 | Insecta | Lepidoptera | Geometridae | *Macaria* | *artesiaria* | 95.83 |

FinPROTAX output

|  | Class | Order | Family | Genus | Species | Probability |
| --- | --- | --- | --- | --- | --- | --- |
|  | **Insecta** |  |  |  |  | **0.999982** |
|  | **Insecta** | **Lepidoptera** |  |  |  | **0.935354** |
|  | Insecta | Lepidoptera | Crambidae |  |  | 0.155193 |
|  | Insecta | Lepidoptera | Crambidae | unk |  | 0.115953 |
|  | Insecta | Lepidoptera | Noctuidae |  |  | 0.522577 |
|  | Insecta | Lepidoptera | Tortricidae |  |  | 0.147066 |

7. *Catocala nymphagoga*, Esper 1787

Matches to the non-local *Catocala nymphagoga* consistently give matches at a 1 bp difference to the congeneric as well as more locally occurring species *Catocala sponsa* (Linnaeus 1767) and *Catocala fraxini* (Linnaeus 1758). It should be noted, however, that *C. sponsa* and *C. fraxini* were rarely found as good matches for any sequences elsewhere in this batch (*C. fraxini* was assigned to one ASV based on a 98.61% match, and once at 97.92%; one ASV with an abundance <0.1% was identified as *C. sponsa* at 99.21%, with equally likely matches to *C. nymphagoga* listed for this one). As such, the observations of *C. nymphagoga* appeared sufficiently realistic.

>7a

AATAGTAGGAACTTCATTAAGATTATTAATTCGAGCTGAATTAGGTAACCCCGGATCTTTAATTGGAGATGATCAAATTTATAATACTATTGTTACAGCTCACGCTTTTATTATAATTTTTTTTATAGTTATACCAATCATAATT

BOLD output

| # | Class | Order | Family | Genus | Species | Match % |
| --- | --- | --- | --- | --- | --- | --- |
| 36 | **Insecta** | **Lepidoptera** | **Erebidae** | ***Catocala*** | ***nymphagoga*** | **99.31** |
| 1 | **Insecta** | **Lepidoptera** | **Erebidae** | ***Catocala*** | ***nymphogaga*** | **99.31** |
| 1 | Insecta | Lepidoptera | Erebidae | *Catocala* | *nymphagoga* | 99.3 |
| 1 | Insecta | Lepidoptera | Crambidae | *Microcramboides* | *meretricellus* | 98.82 |
| 1 | Insecta | Lepidoptera | Erebidae | *Catocala* | *sponsa* | 98.61 |
| 2 | Insecta | Lepidoptera | Erebidae | *Catocala* | *nymphagoga* | 98.61 |
| 6 | Insecta | Lepidoptera | Erebidae | *Catocala* | *herodias* | 98.61 |
| 15 | Insecta | Lepidoptera | Erebidae | *Catocala* | *concumbens* | 98.61 |
| 1 | Insecta | Lepidoptera | Erebidae | *Leucanopsis* | *Espinoza02* | 98.61 |
| 4 | Insecta | Lepidoptera | Erebidae | *Catocala* | *fraxini* | 98.61 |
| 17 | Insecta | Lepidoptera | Erebidae | *Catocala* | *sponsa* | 97.92 |
| 1 | Insecta | Lepidoptera | Erebidae | *Leucanopsis* | *tabernilla* | 97.92 |
| 14 | Insecta | Lepidoptera | Noctuidae | *Homorthodes* | *communis* | 97.92 |

FinPROTAX output

|  | Class | Order | Family | Genus | Species | Probability |
| --- | --- | --- | --- | --- | --- | --- |
|  | **Insecta** |  |  |  |  | **0.999994** |
|  | **Insecta** | **Lepidoptera** |  |  |  | **0.977047** |
|  | **Insecta** | **Lepidoptera** | **Erebidae** |  |  | **0.556470** |
|  | Insecta | Lepidoptera | Erebidae | *Arctia* |  | 0.105607 |
|  | Insecta | Lepidoptera | Erebidae | *Arctia* | unk | 0.104487 |
|  | **Insecta** | **Lepidoptera** | **Erebidae** | ***Catocala*** |  | **0.382648** |
|  | Insecta | Lepidoptera | Erebidae | *Catocala* | *fraxini* | 0.142945 |
|  | Insecta | Lepidoptera | Erebidae | *Catocala* | *sponsa* | 0.142945 |
|  | Insecta | Lepidoptera | Noctuidae |  |  | 0.181535 |
|  | Insecta | Lepidoptera | Tortricidae |  |  | 0.150408 |

>7b

GAATAGTAGGAACTTCATTAAGATTATTAATTCGAGCTGAATTAGGTAACCCCGGATCCTTAATTGGAGATGATCAAATTTATAATACTATTGTTACAGCTCATGCTTTTATTATAATTTTTTTATAGTTATACCAATCATAATT

BOLD output

| # | Class | Order | Family | Genus | Species | Match % |
| --- | --- | --- | --- | --- | --- | --- |
| 37 | **Insecta** | **Lepidoptera** | **Erebidae** | ***Catocala*** | ***nymphagoga*** | **99.3** |
| 1 | **Insecta** | **Lepidoptera** | **Erebidae** | ***Catocala*** | ***nymphogaga*** | **99.3** |
| 1 | Insecta | Lepidoptera | Nymphalidae | *Heliconius* | *elevatus* | 98.75 |
| 1 | Insecta | Lepidoptera | Nymphalidae | *Heliconius* | *hecale* | 98.75 |
| 1 | Insecta | Lepidoptera | Erebidae | *Catocala* | *sponsa* | 98.6 |
| 2 | Insecta | Lepidoptera | Erebidae | *Praeamastus* | *cymothoe* | 98.6 |
| 2 | Insecta | Lepidoptera | Erebidae | *Catocala* | *nymphagoga* | 98.6 |
| 6 | Insecta | Lepidoptera | Erebidae | *Catocala* | *herodias* | 98.6 |
| 15 | Insecta | Lepidoptera | Erebidae | *Catocala* | *concumbens* | 98.6 |
| 4 | Insecta | Lepidoptera | Erebidae | *Catocala* | *fraxini* | 98.6 |
| 17 | Insecta | Lepidoptera | Erebidae | *Catocala* | *sponsa* | 97.9 |
| 1 | Insecta | Lepidoptera | Geometridae | *Melanolophia* | *bugnathos* | 97.9 |
| 6 | Insecta | Lepidoptera | Erebidae | *Ammalo* | *helops* | 97.9 |
| 1 | Insecta | Lepidoptera | Erebidae | *Catocala* | *benjamini* | 97.9 |
| 1 | Insecta | Lepidoptera | Erebidae | *Catocala* | *johnsoniana* | 97.9 |
| 1 | Insecta | Lepidoptera | Geometridae | *Melanolophia* | *imperfectaria* | 97.9 |
| 1 | Insecta | Lepidoptera | Geometridae | *Melanolophia* | *mallea* | 97.9 |
| 1 | Insecta | Lepidoptera | Erebidae | *Praeamastus* | *cymothoe* | 97.9 |
| 1 | Insecta | Lepidoptera | Erebidae | *Eublemma* | *loxotoma* | 97.9 |

FinPROTAX output

|  | Class | Order | Family | Genus | Species | Probability |
| --- | --- | --- | --- | --- | --- | --- |
|  | **Insecta** |  |  |  |  | **0.999994** |
|  | **Insecta** | **Lepidoptera** |  |  |  | **0.984177** |
|  | **Insecta** | **Lepidoptera** | **Erebidae** |  |  | **0.652456** |
|  | Insecta | Lepidoptera | Erebidae | *Arctia* |  | 0.106548 |
|  | Insecta | Lepidoptera | Erebidae | *Arctia* | unk | 0.105333 |
|  | **Insecta** | **Lepidoptera** | **Erebidae** | ***Catocala*** |  | **0.423704** |
|  | Insecta | Lepidoptera | Erebidae | *Catocala* | *fraxini* | 0.159086 |
|  | Insecta | Lepidoptera | Erebidae | *Catocala* | *sponsa* | 0.159086 |
|  | Insecta | Lepidoptera | Noctuidae |  |  | 0.216439 |

>7c

AATAGTAGGAACTTCATTAGGATTATTAATTCGAGCTGAATTAGGTAACCCCGGATCTTCAATTGGAGATGATCAAATTTATAATACTATTGTTACAGCTCATGCTTTTATTATAATTTTTTTTATAGTTATACCAATCATAATT

BOLD output

| # | Class | Order | Family | Genus | Species | Match % |
| --- | --- | --- | --- | --- | --- | --- |
| 37 | **Insecta** | **Lepidoptera** | **Erebidae** | ***Catocala*** | ***nymphagoga*** | **99.29** |
| 1 | **Insecta** | **Lepidoptera** | **Erebidae** | ***Catocala*** | ***nymphogaga*** | **99.29** |
| 1 | Insecta | Lepidoptera | Nymphalidae | *Heliconius* | *elevatus* | 98.77 |
| 1 | Insecta | Lepidoptera | Nymphalidae | *Heliconius* | *hecale* | 98.77 |
| 1 | Insecta | Lepidoptera | Erebidae | *Catocala* | *sponsa* | 98.58 |
| 2 | Insecta | Lepidoptera | Erebidae | *Catocala* | *nymphagoga* | 98.58 |
| 6 | Insecta | Lepidoptera | Erebidae | *Catocala* | *herodias* | 98.58 |
| 15 | Insecta | Lepidoptera | Erebidae | *Catocala* | *concumbens* | 98.58 |
| 4 | Insecta | Lepidoptera | Erebidae | *Catocala* | *fraxini* | 98.58 |
| 1 | Insecta | Lepidoptera | Riodinidae | *Stalachtis* | *euterpe* | 97.89 |
| 17 | Insecta | Lepidoptera | Erebidae | *Catocala* | *sponsa* | 97.87 |
| 1 | Insecta | Lepidoptera | Erebidae | *Catocala* | *benjamini* | 97.87 |
| 1 | Insecta | Lepidoptera | Erebidae | *Catocala* | *johnsoniana* | 97.87 |
| 1 | Insecta | Lepidoptera | Geometridae | *Melanolophia* | *imperfectaria* | 97.87 |
| 1 | Insecta | Lepidoptera | Geometridae | *Melanolophia* | *mallea* | 97.87 |
| 1 | Insecta | Lepidoptera | Erebidae | *Axiocteta* | *oenoplex PS1* | 97.87 |
| 2 | Insecta | Lepidoptera | Erebidae | *Praeamastus* | *cymothoe* | 97.87 |
| 1 | Insecta | Lepidoptera | Erebidae | *Renodes* | *Poole05* | 97.87 |
| 1 | Insecta | Lepidoptera | Erebidae | *Leucanopsis* | *aenone* | 97.87 |
| 5 | Insecta | Lepidoptera | Crambidae | *Authaeretis* | *sp. AAL6248* | 97.87 |

FinPROTAX output

|  | Class | Order | Family | Genus | Species | Probability |
| --- | --- | --- | --- | --- | --- | --- |
|  | **Insecta** |  |  |  |  | **0.999994** |
|  | **Insecta** | **Lepidoptera** |  |  |  | **0.984765** |
|  | **Insecta** | **Lepidoptera** | **Erebidae** |  |  | **0.652452** |
|  | Insecta | Lepidoptera | Erebidae | *Arctia* |  | 0.123226 |
|  | Insecta | Lepidoptera | Erebidae | *Arctia* | unk | 0.123001 |
|  | **Insecta** | **Lepidoptera** | **Erebidae** | ***Catocala*** |  | **0.448701** |
|  | **Insecta** | **Lepidoptera** | **Erebidae** | ***Catocala*** | **unk** | **0.248006** |
|  | Insecta | Lepidoptera | Noctuidae |  |  | 0.212847 |

>7d

AATAGTAGGAACTTCATTAAGATTATTAATTCGAGCTGAATTAGGTAACCCCGGATCTTTAATTGGAGATGATCGAATTTATAATACTATTGTTACAGCTCATGCTTTTATTATAATTTTTTTTATAGTTATACCAATCATAATT

BOLD output

| # | Class | Order | Family | Genus | Species | Match % |
| --- | --- | --- | --- | --- | --- | --- |
| 36 | **Insecta** | **Lepidoptera** | **Erebidae** | ***Catocala*** | ***nymphagoga*** | **99.31** |
| 1 | **Insecta** | **Lepidoptera** | **Erebidae** | ***Catocala*** | ***nymphogaga*** | **99.31** |
| 1 | Insecta | Lepidoptera | Erebidae | *Catocala* | *nymphagoga* | 99.3 |
| 1 | Insecta | Lepidoptera | Erebidae | *Catocala* | *sponsa* | 98.61 |
| 2 | Insecta | Lepidoptera | Erebidae | *Catocala* | *nymphagoga* | 98.61 |
| 6 | Insecta | Lepidoptera | Erebidae | *Catocala* | *herodias* | 98.61 |
| 15 | Insecta | Lepidoptera | Erebidae | *Catocala* | *concumbens* | 98.61 |
| 4 | Insecta | Lepidoptera | Erebidae | *Catocala* | *fraxini* | 98.61 |
| 17 | Insecta | Lepidoptera | Erebidae | *Catocala* | *sponsa* | 97.92 |
| 1 | Insecta | Lepidoptera | Erebidae | *Catocala* | *benjamini* | 97.92 |
| 1 | Insecta | Lepidoptera | Erebidae | *Catocala* | *johnsoniana* | 97.92 |
| 1 | Insecta | Lepidoptera | Geometridae | *Melanolophia* | *imperfectaria* | 97.92 |
| 1 | Insecta | Lepidoptera | Geometridae | *Melanolophia* | *mallea* | 97.92 |
| 2 | Insecta | Lepidoptera | Erebidae | *Praeamastus* | *cymothoe* | 97.92 |
| 5 | Insecta | Lepidoptera | Crambidae | *Authaeretis* | *sp. AAL6248* | 97.92 |
| 1 | Insecta | Lepidoptera | Erebidae | *Catocala* | *mesopotamica* | 97.92 |
| 1 | Insecta | Lepidoptera | Geometridae | *Pero* | *AM01Br* | 97.92 |
| 1 | Insecta | Lepidoptera | Erebidae | *Catocala* | *sp.* | 97.92 |
| 1 | Insecta | Lepidoptera | Erebidae | *Catocala* | *herodias* | 97.92 |
| 2 | Insecta | Lepidoptera | Erebidae | *Catocala* | *butleri* | 97.92 |

FinPROTAX output

|  | Class | Order | Family | Genus | Species | Probability |
| --- | --- | --- | --- | --- | --- | --- |
|  | **Insecta** |  |  |  |  | **0.999994** |
|  | **Insecta** | **Lepidoptera** |  |  |  | **0.984768** |
|  | **Insecta** | **Lepidoptera** | **Erebidae** |  |  | **0.652454** |
|  | Insecta | Lepidoptera | Erebidae | *Arctia* |  | 0.123843 |
|  | Insecta | Lepidoptera | Erebidae | *Arctia* | unk | 0.122529 |
|  | **Insecta** | **Lepidoptera** | **Erebidae** | ***Catocala*** |  | **0.448721** |
|  | Insecta | Lepidoptera | Erebidae | *Catocala* | *fraxini* | 0.167628 |
|  | Insecta | Lepidoptera | Erebidae | *Catocala* | *sponsa* | 0.167628 |
|  | Insecta | Lepidoptera | Noctuidae |  |  | 0.212847 |

>7e

AATAGTAGGAACTTCACTAAGATTATTAATTCGAGCTGAATTAGGTAACCCCGGATCTTTAATTGGAGATGATCAAATTTATAATACTATTGTTACAGCTCATGCTTTTATTATAATTTTTTTTATAGTTATACCAATCATAATT

BOLD output

| # | Class | Order | Family | Genus | Species | Match % |
| --- | --- | --- | --- | --- | --- | --- |
| 36 | **Insecta** | **Lepidoptera** | **Erebidae** | ***Catocala*** | ***nymphagoga*** | **99.31** |
| 1 | **Insecta** | **Lepidoptera** | **Erebidae** | ***Catocala*** | ***nymphogaga*** | **99.31** |
| 1 | Insecta | Lepidoptera | Erebidae | *Catocala* | *nymphagoga* | 99.3 |
| 1 | Insecta | Lepidoptera | Erebidae | *Ulotrichopus* | *recchiai* | 99.1 |
| 1 | Insecta | Lepidoptera | Riodinidae | *Stalachtis* | *euterpe* | 98.95 |
| 1 | Insecta | Lepidoptera | Noctuidae | *Helicoverpa* | *gelotopoeon* | 98.92 |
| 1 | Insecta | Lepidoptera | Nymphalidae | *Brevioleria* | *arzalia* | 98.92 |
| 1 | Insecta | Lepidoptera | Nymphalidae | *Heliconius* | *elevatus* | 98.77 |
| 1 | Insecta | Lepidoptera | Nymphalidae | *Heliconius* | *hecale* | 98.77 |
| 1 | Insecta | Lepidoptera | Erebidae | *Catocala* | *sponsa* | 98.61 |
| 2 | Insecta | Lepidoptera | Erebidae | *Catocala* | *nymphagoga* | 98.61 |
| 6 | Insecta | Lepidoptera | Erebidae | *Catocala* | *herodias* | 98.61 |
| 15 | Insecta | Lepidoptera | Erebidae | *Catocala* | *concumbens* | 98.61 |
| 4 | Insecta | Lepidoptera | Erebidae | *Catocala* | *fraxini* | 98.61 |
| 1 | Insecta | Lepidoptera | Erebidae | *Catocala* | *okurai* | 98.21 |
| 27 | Insecta | Lepidoptera | Noctuidae | *Helicoverpa* | *armigera* | 97.92 |

FinPROTAX output

|  | Class | Order | Family | Genus | Species | Probability |
| --- | --- | --- | --- | --- | --- | --- |
|  | **Insecta** |  |  |  |  | **0.999992** |
|  | **Insecta** | **Lepidoptera** |  |  |  | **0.976821** |
|  | **Insecta** | **Lepidoptera** | **Erebidae** |  |  | **0.538207** |
|  | **Insecta** | **Lepidoptera** | **Erebidae** | ***Catocala*** |  | **0.364132** |
|  | Insecta | Lepidoptera | Noctuidae |  |  | 0.175577 |
|  | Insecta | Lepidoptera | Tortricidae |  |  | 0.145471 |

>7f

AATAGTAGGAACTTCATTAAGATTATTAATTCGAGCTGAATTAGGTAACCCCGGATCTTTAATTGGAGATGATCAAATTTATAATACTATTGTAACAGCTCATGCTTTTATTATAATTTTTTTTATAGTTATACCAATCATAATT

BOLD output

| # | Class | Order | Family | Genus | Species | Match % |
| --- | --- | --- | --- | --- | --- | --- |
| 37 | **Insecta** | **Lepidoptera** | **Erebidae** | ***Catocala*** | ***nymphagoga*** | **99.31** |
| 1 | **Insecta** | **Lepidoptera** | **Erebidae** | ***Catocala*** | ***nymphogaga*** | **99.31** |
| 1 | Insecta | Lepidoptera | Erebidae | *Catocala* | *nymphagoga* | 99.3 |
| 1 | Insecta | Lepidoptera | Geometridae | *Neonemoria* | *thalassinata* | 99.11 |
| 3 | Insecta | Lepidoptera | Nymphalidae | *Pseudoscada* | *florula* | 98.92 |
| 3 | Insecta | Lepidoptera | Erebidae | *Catocala* | *crataegi sp. 2-RJB* | 98.61 |
| 1 | Insecta | Lepidoptera | Erebidae | *Catocala* | *sponsa* | 98.61 |
| 2 | Insecta | Lepidoptera | Noctuidae | *Schinia* | *ligeae* | 98.61 |
| 1 | Insecta | Lepidoptera | Erebidae | *Catocala* | *crataegi RJB* | 98.61 |
| 1 | Insecta | Lepidoptera | Erebidae | *Catocala* | *nymphagoga* | 98.61 |
| 1 | Insecta | Lepidoptera | Erebidae | *Eublemma* | *gratissima* | 98.61 |
| 6 | Insecta | Lepidoptera | Erebidae | *Catocala* | *herodias* | 98.61 |
| 2 | Insecta | Lepidoptera | Noctuidae | *Schinia* | *varix* | 98.61 |
| 15 | Insecta | Lepidoptera | Erebidae | *Catocala* | *concumbens* | 98.61 |
| 7 | Insecta | Lepidoptera | Erebidae | *Catocala* | *crataegi* | 98.61 |
| 4 | Insecta | Lepidoptera | Erebidae | *Catocala* | *fraxini* | 98.61 |
| 5 | Insecta | Lepidoptera | Erebidae | *Eucereon* | *consorta* | 98.61 |
| 9 | Insecta | Lepidoptera | Erebidae | *Catocala* | *sponsa* | 97.92 |

FinPROTAX output

|  | Class | Order | Family | Genus | Species | Probability |
| --- | --- | --- | --- | --- | --- | --- |
|  | **Insecta** |  |  |  |  | **0.999989** |
|  | **Insecta** | **Lepidoptera** |  |  |  | **0.950118** |
|  | **Insecta** | **Lepidoptera** | **Erebidae** |  |  | **0.355085** |
|  | Insecta | Lepidoptera | Erebidae | *Arctia* |  | 0.151348 |
|  | Insecta | Lepidoptera | Erebidae | *Arctia* | unk | 0.110639 |
|  | **Insecta** | **Lepidoptera** | **Erebidae** | ***Catocala*** |  | **0.137796** |
|  | Insecta | Lepidoptera | Noctuidae |  |  | 0.389649 |

>7g

AATAGTAGGAACTTCATTAAGATTATTAATTCGAGCTGAATTAGGTAACCCCGGATCTTTAATTGGAGATGATCAAATTTATAATACTATTTTTACAGCTCATGCTTTTATTATAATTTTTTTTATAGTTATACCAATCATAATT

BOLD output

| # | Class | Order | Family | Genus | Species | Match % |
| --- | --- | --- | --- | --- | --- | --- |
| 36 | **Insecta** | **Lepidoptera** | **Erebidae** | ***Catocala*** | ***nymphagoga*** | **99.31** |
| 1 | **Insecta** | **Lepidoptera** | **Erebidae** | ***Catocala*** | ***nymphogaga*** | **99.31** |
| 1 | Insecta | Lepidoptera | Erebidae | *Catocala* | *nymphagoga* | 99.3 |
| 1 | Insecta | Lepidoptera | Erebidae | *Catocala* | *sponsa* | 98.61 |
| 2 | Insecta | Lepidoptera | Erebidae | *Catocala* | *nymphagoga* | 98.61 |
| 6 | Insecta | Lepidoptera | Erebidae | *Catocala* | *herodias* | 98.61 |
| 15 | Insecta | Lepidoptera | Erebidae | *Catocala* | *concumbens* | 98.61 |
| 4 | Insecta | Lepidoptera | Erebidae | *Catocala* | *fraxini* | 98.61 |
| 17 | Insecta | Lepidoptera | Erebidae | *Catocala* | *sponsa* | 97.92 |
| 1 | Insecta | Lepidoptera | Erebidae | *Catocala* | *benjamini* | 97.92 |
| 1 | Insecta | Lepidoptera | Erebidae | *Catocala* | *johnsoniana* | 97.92 |
| 1 | Insecta | Lepidoptera | Geometridae | *Melanolophia* | *imperfectaria* | 97.92 |
| 1 | Insecta | Lepidoptera | Geometridae | *Melanolophia* | *mallea* | 97.92 |
| 2 | Insecta | Lepidoptera | Erebidae | *Praeamastus* | *cymothoe* | 97.92 |
| 5 | Insecta | Lepidoptera | Crambidae | *Authaeretis* | *sp. AAL6248* | 97.92 |
| 1 | Insecta | Lepidoptera | Erebidae | *Catocala* | *mesopotamica* | 97.92 |
| 1 | Insecta | Lepidoptera | Geometridae | *Pero* | *AM01Br* | 97.92 |
| 1 | Insecta | Lepidoptera | Erebidae | *Catocala* | *sp.* | 97.92 |
| 1 | Insecta | Lepidoptera | Erebidae | *Catocala* | *herodias* | 97.92 |
| 2 | Insecta | Lepidoptera | Erebidae | *Catocala* | *butleri* | 97.92 |

FinPROTAX output

|  | Class | Order | Family | Genus | Species | Probability |
| --- | --- | --- | --- | --- | --- | --- |
|  | **Insecta** |  |  |  |  | **0.999994** |
|  | **Insecta** | **Lepidoptera** |  |  |  | **0.984768** |
|  | **Insecta** | **Lepidoptera** | **Erebidae** |  |  | **0.652454** |
|  | Insecta | Lepidoptera | Erebidae | *Arctia* |  | 0.123843 |
|  | Insecta | Lepidoptera | Erebidae | *Arctia* | unk | 0.122529 |
|  | **Insecta** | **Lepidoptera** | **Erebidae** | ***Catocala*** |  | **0.448721** |
|  | Insecta | Lepidoptera | Erebidae | *Catocala* | *fraxini* | 0.167628 |
|  | Insecta | Lepidoptera | Erebidae | *Catocala* | *sponsa* | 0.167628 |
|  | Insecta | Lepidoptera | Noctuidae |  |  | 0.212847 |

>7h

AATAGTAGGAACTTCATTAAGATTATTAATTCGAGCTGAATTAGGTAACCCCGGATCTTTAATTGGAGATGATCAAATTTATAATACTATTGTTACAGCTCATGCTTTTATTATAATTTTTTTTATAGTTATGCCAATCATAATT

BOLD output

| # | Class | Order | Family | Genus | Species | Match % |
| --- | --- | --- | --- | --- | --- | --- |
| 36 | **Insecta** | **Lepidoptera** | **Erebidae** | ***Catocala*** | ***nymphagoga*** | **99.31** |
| 1 | **Insecta** | **Lepidoptera** | **Erebidae** | ***Catocala*** | ***nymphogaga*** | **99.31** |
| 1 | Insecta | Lepidoptera | Erebidae | *Catocala* | *nymphagoga* | 99.3 |
| 1 | Insecta | Lepidoptera | Riodinidae | *Stalachtis* | *euterpe* | 98.95 |
| 1 | Insecta | Lepidoptera | Erebidae | *Catocala* | *sponsa* | 98.61 |
| 2 | Insecta | Lepidoptera | Erebidae | *Catocala* | *aholibah* | 98.61 |
| 2 | Insecta | Lepidoptera | Erebidae | *Catocala* | *nymphagoga* | 98.61 |
| 6 | Insecta | Lepidoptera | Erebidae | *Catocala* | *herodias* | 98.61 |
| 15 | Insecta | Lepidoptera | Erebidae | *Catocala* | *concumbens* | 98.61 |
| 4 | Insecta | Lepidoptera | Erebidae | *Catocala* | *fraxini* | 98.61 |
| 1 | Insecta | Lepidoptera | Geometridae | *Neonemoria* | *thalassinata* | 98.21 |
| 1 | Insecta | Lepidoptera | Erebidae | *Catocala* | *dotata* | 98.21 |
| 1 | Insecta | Lepidoptera | Erebidae | *Ulotrichopus* | *recchiai* | 98.2 |
| 2 | Insecta | Lepidoptera | Erebidae | *Catocala* | *viviannae* | 97.92 |
| 17 | Insecta | Lepidoptera | Erebidae | *Catocala* | *sponsa* | 97.92 |
| 1 | Insecta | Lepidoptera | Erebidae | *Catocala* | *ohshimai* | 97.92 |
| 8 | Insecta | Lepidoptera | Erebidae | *Catocala* | *dilecta* | 97.92 |

FinPROTAX output

|  | Class | Order | Family | Genus | Species | Probability |
| --- | --- | --- | --- | --- | --- | --- |
|  | **Insecta** |  |  |  |  | **0.999991** |
|  | **Insecta** | **Lepidoptera** |  |  |  | **0.980086** |
|  | **Insecta** | **Lepidoptera** | **Erebidae** |  |  | **0.557576** |
|  | Insecta | Lepidoptera | Erebidae | *Arctia* |  | 0.105834 |
|  | Insecta | Lepidoptera | Erebidae | *Arctia* | unk | 0.104711 |
|  | **Insecta** | **Lepidoptera** | **Erebidae** | ***Catocala*** |  | **0.383470** |
|  | Insecta | Lepidoptera | Erebidae | *Catocala* | *fraxini* | 0.143252 |
|  | Insecta | Lepidoptera | Erebidae | *Catocala* | *sponsa* | 0.143252 |
|  | Insecta | Lepidoptera | Noctuidae |  |  | 0.181896 |
|  | Insecta | Lepidoptera | Tortricidae |  |  | 0.150707 |

>7i

AATAGTAGGAACTTCATTAAGATTATTAATTCGAGCTGAATTAGGTAACCCCGGATCTTTAATTGGAGATGATCAAATTTATAATACTATTGTTACAGCTCATGCTTTTATTATAATTTTTTTTATAGCTATACCAATCATAATT

BOLD output

| # | Class | Order | Family | Genus | Species | Match % |
| --- | --- | --- | --- | --- | --- | --- |
| 36 | **Insecta** | **Lepidoptera** | **Erebidae** | ***Catocala*** | ***nymphagoga*** | **99.31** |
| 1 | **Insecta** | **Lepidoptera** | **Erebidae** | ***Catocala*** | ***nymphogaga*** | **99.31** |
| 1 | Insecta | Lepidoptera | Erebidae | *Catocala* | *nymphagoga* | 99.3 |
| 1 | Insecta | Lepidoptera | Erebidae | *Catocala* | *sponsa* | 98.61 |
| 2 | Insecta | Lepidoptera | Erebidae | *Catocala* | *nymphagoga* | 98.61 |
| 6 | Insecta | Lepidoptera | Erebidae | *Catocala* | *herodias* | 98.61 |
| 15 | Insecta | Lepidoptera | Erebidae | *Catocala* | *concumbens* | 98.61 |
| 4 | Insecta | Lepidoptera | Erebidae | *Catocala* | *fraxini* | 98.61 |
| 1 | Insecta | Lepidoptera | Geometridae | *Neonemoria* | *thalassinata* | 98.21 |
| 1 | Insecta | Lepidoptera | Erebidae | *Catocala* | *dotata* | 98.21 |
| 1 | Insecta | Lepidoptera | Erebidae | *Ulotrichopus* | *recchiai* | 98.2 |
| 17 | Insecta | Lepidoptera | Erebidae | *Catocala* | *sponsa* | 97.92 |
| 1 | Insecta | Lepidoptera | Erebidae | *Catocala* | *benjamini* | 97.92 |
| 1 | Insecta | Lepidoptera | Erebidae | *Catocala* | *johnsoniana* | 97.92 |
| 1 | Insecta | Lepidoptera | Geometridae | *Melanolophia* | *imperfectaria* | 97.92 |
| 1 | Insecta | Lepidoptera | Geometridae | *Melanolophia* | *mallea* | 97.92 |
| 2 | Insecta | Lepidoptera | Erebidae | *Praeamastus* | *cymothoe* | 97.92 |
| 5 | Insecta | Lepidoptera | Crambidae | *Authaeretis* | *sp. AAL6248* | 97.92 |
| 1 | Insecta | Lepidoptera | Erebidae | *Catocala* | *mesopotamica* | 97.92 |
| 1 | Insecta | Lepidoptera | Geometridae | *Pero* | *AM01Br* | 97.92 |
| 1 | Insecta | Lepidoptera | Erebidae | *Catocala* | *sp.* | 97.92 |

FinPROTAX output

|  | Class | Order | Family | Genus | Species | Probability |
| --- | --- | --- | --- | --- | --- | --- |
|  | **Insecta** |  |  |  |  | **0.999994** |
|  | **Insecta** | **Lepidoptera** |  |  |  | **0.984768** |
|  | **Insecta** | **Lepidoptera** | **Erebidae** |  |  | **0.652454** |
|  | Insecta | Lepidoptera | Erebidae | *Arctia* |  | 0.123843 |
|  | Insecta | Lepidoptera | Erebidae | *Arctia* | unk | 0.122529 |
|  | **Insecta** | **Lepidoptera** | **Erebidae** | ***Catocala*** |  | **0.448721** |
|  | Insecta | Lepidoptera | Erebidae | *Catocala* | *fraxini* | 0.167628 |
|  | Insecta | Lepidoptera | Erebidae | *Catocala* | *sponsa* | 0.167628 |
|  | Insecta | Lepidoptera | Noctuidae |  |  | 0.212847 |

>7j

AATAGTAGGAACTTCATTAAGATTATTAATTCGAGCTGAATTAGGTAACCCCGGATCTTTAATTGGAGATGATCAAATTTATAATACTATTGTTACAGCTCATGCTTTTATTATAATTTTTTTTATAGTTATACCAATCATAATT

BOLD output

| # | Class | Order | Family | Genus | Species | Match % |
| --- | --- | --- | --- | --- | --- | --- |
| 37 | **Insecta** | **Lepidoptera** | **Erebidae** | ***Catocala*** | ***nymphagoga*** | **100** |
| 1 | **Insecta** | **Lepidoptera** | **Erebidae** | ***Catocala*** | ***nymphogaga*** | **100** |
| 1 | Insecta | Lepidoptera | Erebidae | *Catocala* | *sponsa* | 99.31 |
| 2 | Insecta | Lepidoptera | Erebidae | *Catocala* | *nymphagoga* | 99.31 |
| 6 | Insecta | Lepidoptera | Erebidae | *Catocala* | *herodias* | 99.31 |
| 15 | Insecta | Lepidoptera | Erebidae | *Catocala* | *concumbens* | 99.31 |
| 4 | Insecta | Lepidoptera | Erebidae | *Catocala* | *fraxini* | 99.31 |
| 1 | Insecta | Lepidoptera | Riodinidae | *Stalachtis* | *euterpe* | 98.95 |
| 1 | Insecta | Lepidoptera | Noctuidae | *Helicoverpa* | *gelotopoeon* | 98.92 |
| 1 | Insecta | Lepidoptera | Nymphalidae | *Brevioleria* | *arzalia* | 98.92 |
| 1 | Insecta | Lepidoptera | Nymphalidae | *Heliconius* | *elevatus* | 98.77 |
| 1 | Insecta | Lepidoptera | Nymphalidae | *Heliconius* | *hecale* | 98.77 |
| 17 | Insecta | Lepidoptera | Erebidae | *Catocala* | *sponsa* | 98.61 |
| 1 | Insecta | Lepidoptera | Erebidae | *Catocala* | *benjamini* | 98.61 |
| 1 | Insecta | Lepidoptera | Erebidae | *Catocala* | *johnsoniana* | 98.61 |
| 1 | Insecta | Lepidoptera | Geometridae | *Melanolophia* | *imperfectaria* | 98.61 |
| 1 | Insecta | Lepidoptera | Geometridae | *Melanolophia* | *mallea* | 98.61 |
| 2 | Insecta | Lepidoptera | Erebidae | *Praeamastus* | *cymothoe* | 98.61 |
| 5 | Insecta | Lepidoptera | Crambidae | *Authaeretis* | *sp. AAL6248* | 98.61 |
| 1 | Insecta | Lepidoptera | Erebidae | *Catocala* | *mesopotamica* | 98.61 |

FinPROTAX output

|  | Class | Order | Family | Genus | Species | Probability |
| --- | --- | --- | --- | --- | --- | --- |
|  | **Insecta** |  |  |  |  | **0.999995** |
|  | **Insecta** | **Lepidoptera** |  |  |  | **0.984769** |
|  | **Insecta** | **Lepidoptera** | **Erebidae** |  |  | **0.652455** |
|  | Insecta | Lepidoptera | Erebidae | *Arctia* |  | 0.124197 |
|  | Insecta | Lepidoptera | Erebidae | *Arctia* | unk | 0.116858 |
|  | **Insecta** | **Lepidoptera** | **Erebidae** | ***Catocala*** |  | **0.448733** |
|  | Insecta | Lepidoptera | Erebidae | *Catocala* | *fraxini* | 0.195935 |
|  | Insecta | Lepidoptera | Erebidae | *Catocala* | *sponsa* | 0.195935 |
|  | Insecta | Lepidoptera | Noctuidae |  |  | 0.212848 |

>7k

AATAGTAGGAACTTCATTAAGATTATTAATTCGAGCTGAATTAGGTAACCCCGGATCTTTAATTGGAGATGATCAAATTTATAGTACTATTGTTACAGCTCATGCTTTTATTATAATTTTTTTTATAGTTATACCAATCATAATT

BOLD output

| # | Class | Order | Family | Genus | Species | Match % |
| --- | --- | --- | --- | --- | --- | --- |
| 36 | **Insecta** | **Lepidoptera** | **Erebidae** | ***Catocala*** | ***nymphagoga*** | **99.31** |
| 1 | **Insecta** | **Lepidoptera** | **Erebidae** | ***Catocala*** | ***nymphogaga*** | **99.31** |
| 1 | Insecta | Lepidoptera | Erebidae | *Catocala* | *nymphagoga* | 99.3 |
| 1 | Insecta | Lepidoptera | Erebidae | *Catocala* | *sponsa* | 98.61 |
| 2 | Insecta | Lepidoptera | Erebidae | *Catocala* | *nymphagoga* | 98.61 |
| 6 | Insecta | Lepidoptera | Erebidae | *Catocala* | *herodias* | 98.61 |
| 15 | Insecta | Lepidoptera | Erebidae | *Catocala* | *concumbens* | 98.61 |
| 4 | Insecta | Lepidoptera | Erebidae | *Catocala* | *fraxini* | 98.61 |
| 17 | Insecta | Lepidoptera | Erebidae | *Catocala* | *sponsa* | 97.92 |
| 1 | Insecta | Lepidoptera | Erebidae | *Catocala* | *benjamini* | 97.92 |
| 1 | Insecta | Lepidoptera | Erebidae | *Catocala* | *johnsoniana* | 97.92 |
| 1 | Insecta | Lepidoptera | Geometridae | *Melanolophia* | *imperfectaria* | 97.92 |
| 1 | Insecta | Lepidoptera | Geometridae | *Melanolophia* | *mallea* | 97.92 |
| 2 | Insecta | Lepidoptera | Erebidae | *Praeamastus* | *cymothoe* | 97.92 |
| 5 | Insecta | Lepidoptera | Crambidae | *Authaeretis* | *sp. AAL6248* | 97.92 |
| 1 | Insecta | Lepidoptera | Erebidae | *Catocala* | *mesopotamica* | 97.92 |
| 1 | Insecta | Lepidoptera | Geometridae | *Pero* | *AM01Br* | 97.92 |
| 1 | Insecta | Lepidoptera | Erebidae | *Catocala* | *sp.* | 97.92 |
| 1 | Insecta | Lepidoptera | Erebidae | *Catocala* | *herodias* | 97.92 |
| 2 | Insecta | Lepidoptera | Erebidae | *Catocala* | *butleri* | 97.92 |

FinPROTAX output

|  | Class | Order | Family | Genus | Species | Probability |
| --- | --- | --- | --- | --- | --- | --- |
|  | **Insecta** |  |  |  |  | **0.999994** |
|  | **Insecta** | **Lepidoptera** |  |  |  | **0.984768** |
|  | **Insecta** | **Lepidoptera** | **Erebidae** |  |  | **0.652454** |
|  | Insecta | Lepidoptera | Erebidae | *Arctia* |  | 0.123843 |
|  | Insecta | Lepidoptera | Erebidae | *Arctia* | unk | 0.122529 |
|  | **Insecta** | **Lepidoptera** | **Erebidae** | ***Catocala*** |  | **0.448721** |
|  | Insecta | Lepidoptera | Erebidae | *Catocala* | *fraxini* | 0.167628 |
|  | Insecta | Lepidoptera | Erebidae | *Catocala* | *sponsa* | 0.167628 |
|  | Insecta | Lepidoptera | Noctuidae |  |  | 0.212847 |

>7l

AATAGTAGGAACTTCATTAAGATTATTAATTCGAGCTGAATTAGGTAACCCCGGATCTTTAATTGGAGATGATCAAATTTATAATACTATTGTTACAGCTCATGCTTTTATTATAATTCTTTTTATAGTTATACCAATCATAATT

BOLD output

| # | Class | Order | Family | Genus | Species | Match % |
| --- | --- | --- | --- | --- | --- | --- |
| 36 | **Insecta** | **Lepidoptera** | **Erebidae** | ***Catocala*** | ***nymphagoga*** | **99.31** |
| 1 | **Insecta** | **Lepidoptera** | **Erebidae** | ***Catocala*** | ***nymphogaga*** | **99.31** |
| 1 | Insecta | Lepidoptera | Erebidae | *Catocala* | *nymphagoga* | 99.3 |
| 1 | Insecta | Lepidoptera | Erebidae | *Catocala* | *sponsa* | 98.61 |
| 2 | Insecta | Lepidoptera | Erebidae | *Catocala* | *nymphagoga* | 98.61 |
| 6 | Insecta | Lepidoptera | Erebidae | *Catocala* | *herodias* | 98.61 |
| 15 | Insecta | Lepidoptera | Erebidae | *Catocala* | *concumbens* | 98.61 |
| 4 | Insecta | Lepidoptera | Erebidae | *Catocala* | *fraxini* | 98.61 |
| 1 | Insecta | Lepidoptera | Geometridae | *Neonemoria* | *thalassinata* | 98.21 |
| 1 | Insecta | Lepidoptera | Erebidae | *Catocala* | *dotata* | 98.21 |
| 1 | Insecta | Lepidoptera | Erebidae | *Ulotrichopus* | *recchiai* | 98.2 |
| 17 | Insecta | Lepidoptera | Erebidae | *Catocala* | *sponsa* | 97.92 |
| 1 | Insecta | Lepidoptera | Erebidae | *Catocala* | *benjamini* | 97.92 |
| 1 | Insecta | Lepidoptera | Erebidae | *Catocala* | *johnsoniana* | 97.92 |
| 1 | Insecta | Lepidoptera | Geometridae | *Melanolophia* | *imperfectaria* | 97.92 |
| 1 | Insecta | Lepidoptera | Geometridae | *Melanolophia* | *mallea* | 97.92 |
| 2 | Insecta | Lepidoptera | Erebidae | *Praeamastus* | *cymothoe* | 97.92 |
| 5 | Insecta | Lepidoptera | Crambidae | *Authaeretis* | *sp. AAL6248* | 97.92 |
| 1 | Insecta | Lepidoptera | Erebidae | *Catocala* | *mesopotamica* | 97.92 |
| 1 | Insecta | Lepidoptera | Geometridae | *Pero* | *AM01Br* | 97.92 |
| 1 | Insecta | Lepidoptera | Erebidae | *Catocala* | *sp.* | 97.92 |

FinPROTAX output

|  | Class | Order | Family | Genus | Species | Probability |
| --- | --- | --- | --- | --- | --- | --- |
|  | **Insecta** |  |  |  |  | **0.999994** |
|  | **Insecta** | **Lepidoptera** |  |  |  | **0.984768** |
|  | **Insecta** | **Lepidoptera** | **Erebidae** |  |  | **0.652454** |
|  | Insecta | Lepidoptera | Erebidae | *Arctia* |  | 0.123843 |
|  | Insecta | Lepidoptera | Erebidae | *Arctia* | unk | 0.122529 |
|  | **Insecta** | **Lepidoptera** | **Erebidae** | ***Catocala*** |  | **0.448721** |
|  | Insecta | Lepidoptera | Erebidae | *Catocala* | *fraxini* | 0.167628 |
|  | Insecta | Lepidoptera | Erebidae | *Catocala* | *sponsa* | 0.167628 |
|  | Insecta | Lepidoptera | Noctuidae |  |  | 0.212847 |

>7m

AATAGTAGGAACTTCATTAAGATTATTAATTCGAGCTGAATTAGGTAACCCCGGATCTTTAATTGGAGATGATCAAATTTATAATACTACTGTTACAGCTCATGCTTTTATTATAATTTTTTTTATAGTTATACCAATCATAATT

BOLD output

| # | Class | Order | Family | Genus | Species | Match % |
| --- | --- | --- | --- | --- | --- | --- |
| 36 | **Insecta** | **Lepidoptera** | **Erebidae** | ***Catocala*** | ***nymphagoga*** | **99.31** |
| 1 | **Insecta** | **Lepidoptera** | **Erebidae** | ***Catocala*** | ***nymphogaga*** | **99.31** |
| 1 | Insecta | Lepidoptera | Erebidae | *Catocala* | *nymphagoga* | 99.3 |
| 1 | Insecta | Lepidoptera | Erebidae | *Catocala* | *sponsa* | 98.61 |
| 2 | Insecta | Lepidoptera | Erebidae | *Catocala* | *nymphagoga* | 98.61 |
| 6 | Insecta | Lepidoptera | Erebidae | *Catocala* | *herodias* | 98.61 |
| 15 | Insecta | Lepidoptera | Erebidae | *Catocala* | *concumbens* | 98.61 |
| 4 | Insecta | Lepidoptera | Erebidae | *Catocala* | *fraxini* | 98.61 |
| 17 | Insecta | Lepidoptera | Erebidae | *Catocala* | *sponsa* | 97.92 |
| 1 | Insecta | Lepidoptera | Erebidae | *Catocala* | *benjamini* | 97.92 |
| 1 | Insecta | Lepidoptera | Erebidae | *Catocala* | *johnsoniana* | 97.92 |
| 1 | Insecta | Lepidoptera | Geometridae | *Melanolophia* | *imperfectaria* | 97.92 |
| 1 | Insecta | Lepidoptera | Geometridae | *Melanolophia* | *mallea* | 97.92 |
| 2 | Insecta | Lepidoptera | Erebidae | *Praeamastus* | *cymothoe* | 97.92 |
| 5 | Insecta | Lepidoptera | Crambidae | *Authaeretis* | *sp. AAL6248* | 97.92 |
| 1 | Insecta | Lepidoptera | Erebidae | *Catocala* | *mesopotamica* | 97.92 |
| 1 | Insecta | Lepidoptera | Geometridae | *Pero* | *AM01Br* | 97.92 |
| 1 | Insecta | Lepidoptera | Erebidae | *Catocala* | *sp.* | 97.92 |
| 1 | Insecta | Lepidoptera | Erebidae | *Catocala* | *herodias* | 97.92 |
| 2 | Insecta | Lepidoptera | Erebidae | *Catocala* | *butleri* | 97.92 |

FinPROTAX output

|  | Class | Order | Family | Genus | Species | Probability |
| --- | --- | --- | --- | --- | --- | --- |
|  | **Insecta** |  |  |  |  | **0.999994** |
|  | **Insecta** | **Lepidoptera** |  |  |  | **0.984768** |
|  | **Insecta** | **Lepidoptera** | **Erebidae** |  |  | **0.652454** |
|  | Insecta | Lepidoptera | Erebidae | *Arctia* |  | 0.123843 |
|  | Insecta | Lepidoptera | Erebidae | *Arctia* | unk | 0.122529 |
|  | **Insecta** | **Lepidoptera** | **Erebidae** | ***Catocala*** |  | **0.448721** |
|  | Insecta | Lepidoptera | Erebidae | *Catocala* | *fraxini* | 0.167628 |
|  | Insecta | Lepidoptera | Erebidae | *Catocala* | *sponsa* | 0.167628 |
|  | Insecta | Lepidoptera | Noctuidae |  |  | 0.212847 |

>7n

AATAGTAGGAACTTCATTAAGATTATTAATTCGAGCCGAATTAGGTAACCCCGGATCTTTAATTGGAGATGATCAAATTTATAATACTATTGTTACAGCTCATGCTTTTATTATAATTTTTTTTATAGTTATACCAATCATAATT

BOLD output

| # | Class | Order | Family | Genus | Species | Match % |
| --- | --- | --- | --- | --- | --- | --- |
| 36 | **Insecta** | **Lepidoptera** | **Erebidae** | ***Catocala*** | ***nymphagoga*** | **99.31** |
| 1 | **Insecta** | **Lepidoptera** | **Erebidae** | ***Catocala*** | ***nymphogaga*** | **99.31** |
| 1 | Insecta | Lepidoptera | Erebidae | *Catocala* | *nymphagoga* | 99.3 |
| 1 | Insecta | Lepidoptera | Riodinidae | *Stalachtis* | *euterpe* | 98.95 |
| 1 | Insecta | Lepidoptera | Noctuidae | *Helicoverpa* | *gelotopoeon* | 98.92 |
| 1 | Insecta | Lepidoptera | Nymphalidae | *Brevioleria* | *arzalia* | 98.92 |
| 1 | Insecta | Lepidoptera | Nymphalidae | *Heliconius* | *elevatus* | 98.77 |
| 1 | Insecta | Lepidoptera | Nymphalidae | *Heliconius* | *hecale* | 98.77 |
| 1 | Insecta | Lepidoptera | Erebidae | *Catocala* | *sponsa* | 98.61 |
| 3 | Insecta | Lepidoptera | Erebidae | *Catocala* | *amatrix* | 98.61 |
| 2 | Insecta | Lepidoptera | Erebidae | *Catocala* | *nymphagoga* | 98.61 |
| 6 | Insecta | Lepidoptera | Erebidae | *Catocala* | *herodias* | 98.61 |
| 15 | Insecta | Lepidoptera | Erebidae | *Catocala* | *concumbens* | 98.61 |
| 4 | Insecta | Lepidoptera | Erebidae | *Catocala* | *fraxini* | 98.61 |
| 26 | Insecta | Lepidoptera | Noctuidae | *Helicoverpa* | *armigera* | 97.92 |

FinPROTAX output

|  | Class | Order | Family | Genus | Species | Probability |
| --- | --- | --- | --- | --- | --- | --- |
|  | **Insecta** |  |  |  |  | **0.999994** |
|  | **Insecta** | **Lepidoptera** |  |  |  | **0.979879** |
|  | **Insecta** | **Lepidoptera** | **Erebidae** |  |  | **0.572332** |
|  | **Insecta** | **Lepidoptera** | **Erebidae** | ***Catocala*** |  | **0.384033** |
|  | Insecta | Lepidoptera | Erebidae | *Catocala* | *fraxini* | 0.143456 |
|  | Insecta | Lepidoptera | Erebidae | *Catocala* | *sponsa* | 0.143456 |
|  | Insecta | Lepidoptera | Noctuidae |  |  | 0.186709 |

>7o

AATAATAGGAACTTCATTAAGATTATTAATTCGAGCTGAATTAGGTAACCCCGGATCTTTAATTGGAGATGATCAAATTTATAATACTATTGTTACAGCTCATGCTTTTATTATAATTTTTTTTATAGTTATACCAATCATAATT

BOLD output

| # | Class | Order | Family | Genus | Species | Match % |
| --- | --- | --- | --- | --- | --- | --- |
| 1 | **Insecta** | **Lepidoptera** | **Erebidae** | ***Catocala*** | ***nymphagoga*** | **100** |
| 36 | Insecta | Lepidoptera | Erebidae | *Catocala* | *nymphagoga* | 99.31 |
| 1 | Insecta | Lepidoptera | Erebidae | *Catocala* | *nymphogaga* | 99.31 |
| 1 | Insecta | Lepidoptera | Erebidae | *Catocala* | *nymphagoga* | 99.3 |
| 1 | Insecta | Lepidoptera | Riodinidae | *Stalachtis* | *euterpe* | 98.95 |
| 1 | Insecta | Lepidoptera | Noctuidae | *Helicoverpa* | *gelotopoeon* | 98.92 |
| 1 | Insecta | Lepidoptera | Nymphalidae | *Brevioleria* | *arzalia* | 98.92 |
| 1 | Insecta | Lepidoptera | Nymphalidae | *Heliconius* | *elevatus* | 98.77 |
| 1 | Insecta | Lepidoptera | Nymphalidae | *Heliconius* | *hecale* | 98.77 |
| 1 | Insecta | Lepidoptera | Erebidae | *Catocala* | *sponsa* | 98.61 |
| 1 | Insecta | Lepidoptera | Erebidae | *Catocala* | *nymphagoga* | 98.61 |
| 6 | Insecta | Lepidoptera | Erebidae | *Catocala* | *herodias* | 98.61 |
| 15 | Insecta | Lepidoptera | Erebidae | *Catocala* | *concumbens* | 98.61 |
| 4 | Insecta | Lepidoptera | Erebidae | *Catocala* | *fraxini* | 98.61 |
| 29 | Insecta | Lepidoptera | Noctuidae | *Helicoverpa* | *armigera* | 97.92 |

FinPROTAX output

|  | Class | Order | Family | Genus | Species | Probability |
| --- | --- | --- | --- | --- | --- | --- |
|  | **Insecta** |  |  |  |  | **0.999993** |
|  | **Insecta** | **Lepidoptera** |  |  |  | **0.984523** |
|  | **Insecta** | **Lepidoptera** | **Erebidae** |  |  | **0.534568** |
|  | **Insecta** | **Lepidoptera** | **Erebidae** | ***Catocala*** |  | **0.361522** |
|  | Insecta | Lepidoptera | Erebidae | *Catocala* | *fraxini* | 0.135053 |
|  | Insecta | Lepidoptera | Erebidae | *Catocala* | *sponsa* | 0.135053 |
|  | Insecta | Lepidoptera | Geometridae |  |  | 0.139374 |
|  | Insecta | Lepidoptera | Noctuidae |  |  | 0.174390 |

>7p

AATAGTAGGAACTTCATTAAGATTATTAATTCGAGCTGAATTAGGTAACCCCGGATCTTTAATTGGAGATGATCAAATTTATAATACTATTGTTACAGCTCATGCTTTTATTATAATTTTTTTTATAGTTACACCAATCATAATT

BOLD output

| # | Class | Order | Family | Genus | Species | Match % |
| --- | --- | --- | --- | --- | --- | --- |
| 36 | **Insecta** | **Lepidoptera** | **Erebidae** | ***Catocala*** | ***nymphagoga*** | **99.31** |
| 1 | **Insecta** | **Lepidoptera** | **Erebidae** | ***Catocala*** | ***nymphogaga*** | **99.31** |
| 1 | Insecta | Lepidoptera | Erebidae | *Catocala* | *nymphagoga* | 99.3 |
| 1 | Insecta | Lepidoptera | Erebidae | *Catocala* | *sponsa* | 98.61 |
| 2 | Insecta | Lepidoptera | Erebidae | *Catocala* | *nymphagoga* | 98.61 |
| 6 | Insecta | Lepidoptera | Erebidae | *Catocala* | *herodias* | 98.61 |
| 15 | Insecta | Lepidoptera | Erebidae | *Catocala* | *concumbens* | 98.61 |
| 4 | Insecta | Lepidoptera | Erebidae | *Catocala* | *fraxini* | 98.61 |
| 1 | Insecta | Lepidoptera | Geometridae | *Neonemoria* | *thalassinata* | 98.21 |
| 1 | Insecta | Lepidoptera | Erebidae | *Catocala* | *dotata* | 98.21 |
| 1 | Insecta | Lepidoptera | Erebidae | *Ulotrichopus* | *recchiai* | 98.2 |
| 17 | Insecta | Lepidoptera | Erebidae | *Catocala* | *sponsa* | 97.92 |
| 1 | Insecta | Lepidoptera | Erebidae | *Catocala* | *benjamini* | 97.92 |
| 1 | Insecta | Lepidoptera | Erebidae | *Catocala* | *johnsoniana* | 97.92 |
| 1 | Insecta | Lepidoptera | Geometridae | *Melanolophia* | *imperfectaria* | 97.92 |
| 1 | Insecta | Lepidoptera | Geometridae | *Melanolophia* | *mallea* | 97.92 |
| 2 | Insecta | Lepidoptera | Erebidae | *Praeamastus* | *cymothoe* | 97.92 |
| 5 | Insecta | Lepidoptera | Crambidae | *Authaeretis* | *sp. AAL6248* | 97.92 |
| 1 | Insecta | Lepidoptera | Erebidae | *Catocala* | *mesopotamica* | 97.92 |
| 1 | Insecta | Lepidoptera | Geometridae | *Pero* | *AM01Br* | 97.92 |
| 1 | Insecta | Lepidoptera | Erebidae | *Catocala* | *sp.* | 97.92 |

FinPROTAX output

|  | Class | Order | Family | Genus | Species | Probability |
| --- | --- | --- | --- | --- | --- | --- |
|  | **Insecta** |  |  |  |  | **0.999994** |
|  | **Insecta** | **Lepidoptera** |  |  |  | **0.984768** |
|  | **Insecta** | **Lepidoptera** | **Erebidae** |  |  | **0.652454** |
|  | Insecta | Lepidoptera | Erebidae | *Arctia* |  | 0.123843 |
|  | Insecta | Lepidoptera | Erebidae | *Arctia* | unk | 0.122529 |
|  | **Insecta** | **Lepidoptera** | **Erebidae** | ***Catocala*** |  | **0.448721** |
|  | Insecta | Lepidoptera | Erebidae | *Catocala* | *fraxini* | 0.167628 |
|  | Insecta | Lepidoptera | Erebidae | *Catocala* | *sponsa* | 0.167628 |
|  | Insecta | Lepidoptera | Noctuidae |  |  | 0.212847 |

8. *Dryobotodes tenebrosa*, Esper 1789

>8a

TATAGTAGGTACATCACTAAGATTATTAATTCGTGCTGAATTAGGAAACCCTGGATCCTTAATTGGCGATGATCAAATTTATAATACTATTGTTACAGCTCATGCTTTTATTATAATTTTTTTTATAGTTATACCTATTATAATT

BOLD output

| # | Class | Order | Family | Genus | Species | Match % |
| --- | --- | --- | --- | --- | --- | --- |
| 8 | **Insecta** | **Lepidoptera** | **Noctuidae** | ***Dryobotodes*** | ***tenebrosa*** | **99.31** |
| 1 | Insecta | Lepidoptera | Nymphalidae | *Oeneis* | *sculda* | 98.85 |
| 1 | Insecta | Lepidoptera | Nymphalidae | *Heliconius* | *ethilla* | 98.77 |
| 3 | Insecta | Lepidoptera | Nymphalidae | *Heliconius* | *melpomene* | 98.77 |
| 1 | Insecta | Lepidoptera | Nymphalidae | *Heliconius* | *ethilla x Heliconius melpomene* | 98.75 |
| 1 | Insecta | Lepidoptera | Nymphalidae | *Melinaea* | *menophilus* | 98.75 |
| 1 | Insecta | Lepidoptera | Nymphalidae | *Heliconius* | *ethilla* | 98.73 |
| 1 | Insecta | Lepidoptera | Nymphalidae | *Paralasa* | *styx* | 98.72 |
| 1 | Insecta | Lepidoptera | Noctuidae | *Dryobotodes* | *tenebrosa* | 98.61 |
| 15 | Insecta | Lepidoptera | Nymphalidae | *Heliconius* | *melpomene* | 97.85 |
| 8 | Insecta | Lepidoptera | Nymphalidae | *Heliconius* | *numata* | 97.85 |
| 11 | Insecta | Lepidoptera | Nymphalidae | *Heliconius* | *timareta* | 97.85 |
| 1 | Insecta | Lepidoptera | Nymphalidae | *Calisto* | *pulchella* | 97.7 |
| 1 | Insecta | Lepidoptera | Papilionidae | *Pachliopta* | *aristolochiae* | 97.7 |
| 1 | Insecta | Lepidoptera | Nymphalidae | *Heliconius* | *numata* | 97.7 |
| 1 | Insecta | Lepidoptera | Noctuidae | *Spodoptera* | *frugiperda* | 97.7 |
| 1 | Insecta | Lepidoptera | Nymphalidae | *Bicyclus* | *rhacotis* | 97.7 |
| 1 | Insecta | Lepidoptera | Nymphalidae | *Melinaea* | *marsaeus* | 97.62 |
| 1 | Insecta | Lepidoptera | Nymphalidae | *Heliconius* | *elevatus* | 97.53 |
| 1 | Insecta | Lepidoptera | Nymphalidae | *Heliconius* | *ethilla* | 97.53 |
| 1 | Insecta | Lepidoptera | Nymphalidae | *Heliconius* | *hecale* | 97.53 |
| 1 | Insecta | Lepidoptera | Noctuidae | *Pseudobryomima* | *sp. KLKDNA0340* | 97.53 |
| 1 | Insecta | Lepidoptera | Nymphalidae | *Hyposcada* | *anchiala* | 97.47 |
| 1 | Insecta | Lepidoptera | Papilionidae | *Papilio* | *lorquinianus ssp CBGPFLC_00061* | 97.44 |
| 1 | Insecta | Lepidoptera | Nymphalidae | *Anthanassa* | *drusilla* | 97.44 |
| 5 | Insecta | Lepidoptera | Noctuidae | *Xylena* | *vetusta* | 97.22 |
| 1 | Insecta | Lepidoptera | Noctuidae | *Conistra* | *rubiginosa* | 97.22 |
| 2 | Insecta | Lepidoptera | Noctuidae | *Hyalobole* | *conistroides* | 97.22 |
| 2 | Insecta | Lepidoptera | Noctuidae | *Lithophane* | *contra* | 97.22 |
| 1 | Insecta | Lepidoptera | Noctuidae | *Sympistis* | *griseicollis* | 97.22 |
| 1 | Insecta | Lepidoptera | Limacodidae | *Parasa* | *vitilena* | 97.22 |
| 5 | Insecta | Lepidoptera | Noctuidae | *Jodia* | *croceago* | 97.22 |
| 3 | Insecta | Lepidoptera | Oecophoridae | *Promalactis* | *bitaenia* | 97.22 |
| 7 | Insecta | Lepidoptera | Noctuidae | *Xylena* | *formosa* | 97.22 |
| 1 | Insecta | Lepidoptera | Erebidae | *Simplicia* | *moorei* | 97.22 |
| 1 | Insecta | Lepidoptera | Noctuidae | *Saragossa* | *porosa* | 97.22 |
| 2 | Insecta | Lepidoptera | Gelechiidae | *Ardozyga* | *micropa* | 97.22 |
| 3 | Insecta | Lepidoptera | Noctuidae | *Conistra* | *gallica* | 97.22 |
| 1 | Insecta | Lepidoptera | Noctuidae | *Homoglaea* | *dives* | 97.22 |

FinPROTAX output

|  | Class | Order | Family | Genus | Species | Probability |
| --- | --- | --- | --- | --- | --- | --- |
|  | **Insecta** |  |  |  |  | **0.999960** |
|  | **Insecta** | **Lepidoptera** |  |  |  | **0.987128** |
|  | **Insecta** | **Lepidoptera** | **Noctuidae** |  |  | **0.793961** |
|  | Insecta | Lepidoptera | Tortricidae |  |  | 0.171163 |

>8b

TATGGTAGGTACATCACTAAGATTATTAATTCGTGCTGAATTAGGAAACCCTGGATCTTTAATTGGCGATGATCAAATTTATAATACTATTGTTACAGCTCATGCTTTTATTATAATTTTTTTTATAGTTATACCTATTATAATT

BOLD output

| # | Class | Order | Family | Genus | Species | Match % |
| --- | --- | --- | --- | --- | --- | --- |
| 8 | **Insecta** | **Lepidoptera** | **Noctuidae** | ***Dryobotodes*** | ***tenebrosa*** | **99.31** |
| 1 | Insecta | Lepidoptera | Nymphalidae | *Oeneis* | *sculda* | 98.85 |
| 1 | Insecta | Lepidoptera | Nymphalidae | *Heliconius* | *ethilla* | 98.77 |
| 3 | Insecta | Lepidoptera | Nymphalidae | *Heliconius* | *melpomene* | 98.77 |
| 1 | Insecta | Lepidoptera | Nymphalidae | *Heliconius* | *ethilla x Heliconius melpomene* | 98.75 |
| 1 | Insecta | Lepidoptera | Nymphalidae | *Melinaea* | *menophilus* | 98.75 |
| 1 | Insecta | Lepidoptera | Nymphalidae | *Heliconius* | *ethilla* | 98.73 |
| 1 | Insecta | Lepidoptera | Nymphalidae | *Paralasa* | *styx* | 98.72 |
| 1 | Insecta | Lepidoptera | Noctuidae | *Dryobotodes* | *tenebrosa* | 98.61 |
| 1 | Insecta | Lepidoptera | Erebidae | *Simplicia* | *moorei* | 98.15 |
| 1 | Insecta | Lepidoptera | Hesperiidae | *Quasimellana* | *inconspicua* | 98.1 |
| 38 | Insecta | Lepidoptera | Noctuidae | *Helicoverpa* | *armigera* | 97.92 |
| 1 | Insecta | Lepidoptera | Noctuidae | *Helicoverpa* | *gelotopoeon* | 97.85 |
| 4 | Insecta | Lepidoptera | Noctuidae | *Spodoptera* | *dolichos* | 97.85 |
| 15 | Insecta | Lepidoptera | Nymphalidae | *Heliconius* | *melpomene* | 97.85 |
| 8 | Insecta | Lepidoptera | Nymphalidae | *Heliconius* | *numata* | 97.85 |
| 2 | Insecta | Lepidoptera | Noctuidae | *Spodoptera* | *pulchella* | 97.85 |
| 11 | Insecta | Lepidoptera | Nymphalidae | *Heliconius* | *timareta* | 97.85 |
| 1 | Insecta | Lepidoptera | Nymphalidae | *Brevioleria* | *arzalia* | 97.85 |

FinPROTAX output

|  | Class | Order | Family | Genus | Species | Probability |
| --- | --- | --- | --- | --- | --- | --- |
|  | **Insecta** |  |  |  |  | **0.999950** |
|  | **Insecta** | **Lepidoptera** |  |  |  | **0.977616** |
|  | **Insecta** | **Lepidoptera** | **Noctuidae** |  |  | **0.509640** |
|  | Insecta | Lepidoptera | Tortricidae |  |  | 0.422254 |
|  | Insecta | Lepidoptera | Tortricidae | *Lozotaenia* |  | 0.106284 |

>8c

TATAGTAGGTACATCACTAAGATTATTAATTCGTGCTGAATTAGGAAACCCTGGATCTTTAATTGGCGATGATCAAATTTATAATACTATTGTTACAGCTCATGCTTTTATTATAATTTTTTTTATAGTTATACCTATTATAATT

BOLD output

| # | Class | Order | Family | Genus | Species | Match % |
| --- | --- | --- | --- | --- | --- | --- |
| 8 | **Insecta** | **Lepidoptera** | **Noctuidae** | ***Dryobotodes*** | ***tenebrosa*** | **100** |
| 1 | Insecta | Lepidoptera | Noctuidae | *Dryobotodes* | *tenebrosa* | 99.31 |
| 1 | Insecta | Lepidoptera | Nymphalidae | *Oeneis* | *sculda* | 98.85 |
| 1 | Insecta | Lepidoptera | Nymphalidae | *Heliconius* | *ethilla* | 98.77 |
| 3 | Insecta | Lepidoptera | Nymphalidae | *Heliconius* | *melpomene* | 98.77 |
| 1 | Insecta | Lepidoptera | Nymphalidae | *Heliconius* | *ethilla x Heliconius melpomene* | 98.75 |
| 1 | Insecta | Lepidoptera | Nymphalidae | *Melinaea* | *menophilus* | 98.75 |
| 1 | Insecta | Lepidoptera | Nymphalidae | *Heliconius* | *ethilla* | 98.73 |
| 1 | Insecta | Lepidoptera | Nymphalidae | *Paralasa* | *styx* | 98.72 |
| 1 | Insecta | Lepidoptera | Erebidae | *Simplicia* | *moorei* | 98.15 |
| 1 | Insecta | Lepidoptera | Hesperiidae | *Quasimellana* | *inconspicua* | 98.1 |
| 1 | Insecta | Lepidoptera | Noctuidae | *Conistra* | *rubiginosa* | 97.92 |
| 38 | Insecta | Lepidoptera | Noctuidae | *Helicoverpa* | *armigera* | 97.92 |
| 2 | Insecta | Lepidoptera | Noctuidae | *Hyalobole* | *conistroides* | 97.92 |
| 5 | Insecta | Lepidoptera | Noctuidae | *Jodia* | *croceago* | 97.92 |
| 3 | Insecta | Lepidoptera | Oecophoridae | *Promalactis* | *bitaenia* | 97.92 |
| 7 | Insecta | Lepidoptera | Noctuidae | *Xylena* | *formosa* | 97.92 |
| 2 | Insecta | Lepidoptera | Gelechiidae | *Ardozyga* | *micropa* | 97.92 |
| 3 | Insecta | Lepidoptera | Noctuidae | *Conistra* | *gallica* | 97.92 |
| 8 | Insecta | Lepidoptera | Noctuidae | *Homoglaea* | *dives* | 97.92 |
| 1 | Insecta | Lepidoptera | Noctuidae | *Helicoverpa* | *gelotopoeon* | 97.85 |
| 4 | Insecta | Lepidoptera | Noctuidae | *Spodoptera* | *dolichos* | 97.85 |
| 6 | Insecta | Lepidoptera | Nymphalidae | *Heliconius* | *melpomene* | 97.85 |

FinPROTAX output

|  | Class | Order | Family | Genus | Species | Probability |
| --- | --- | --- | --- | --- | --- | --- |
|  | **Insecta** |  |  |  |  | **0.999966** |
|  | **Insecta** | **Lepidoptera** |  |  |  | **0.977683** |
|  | **Insecta** | **Lepidoptera** | **Noctuidae** |  |  | **0.509675** |
|  | Insecta | Lepidoptera | Tortricidae |  |  | 0.422283 |
|  | Insecta | Lepidoptera | Tortricidae | *Lozotaenia* |  | 0.139642 |

9. *Gonepteryx cleopatra*, Linnaeus 1767

>9

AATAGTAGGAACATCTTTAAGTTTATTAATTCGAACAGAATTAGGTAATCCAGGTTCATTAATTGGAGATGACCAAATTTATAATACTATTGTTACAGCTCATGCTTTTATTATAATTTTTTTTATAGTTATACCTATTATAATT

BOLD output

| # | Class | Order | Family | Genus | Species | Match % |
| --- | --- | --- | --- | --- | --- | --- |
| 100 | **Insecta** | **Lepidoptera** | **Pieridae** | ***Gonepteryx*** | ***cleopatra*** | **100** |

FinPROTAX output

|  | Class | Order | Family | Genus | Species | Probability |
| --- | --- | --- | --- | --- | --- | --- |
|  | **Insecta** |  |  |  |  | **0.999977** |
|  | **Insecta** | **Lepidoptera** |  |  |  | **0.948135** |
|  | Insecta | Lepidoptera | Crambidae |  |  | 0.185741 |
|  | Insecta | Lepidoptera | Crambidae | unk |  | 0.154656 |
|  | Insecta | Lepidoptera | Depressariidae |  |  | 0.260962 |
|  | Insecta | Lepidoptera | Depressariidae | *Depressaria* |  | 0.118681 |
|  | Insecta | Lepidoptera | Depressariidae | *Depressaria* | unk | 0.117135 |
|  | Insecta | Lepidoptera | Tortricidae |  |  | 0.134833 |

10. *Lithosia quadra*, Linnaeus 1758

>10

AATAGTAGGAACTTCATTAAGATTATTAATTCGAGCAGAATTAGGAAATCCAGGATCCTTAATTGGAGATGATCAAATTTATAATACTATTGTAACTGCTCATGCTTTTATTATAATTTTTTTTATGGTTATACCTATTATAATT

BOLD output

| # | Class | Order | Family | Genus | Species | Match % |
| --- | --- | --- | --- | --- | --- | --- |
| 42 | **Insecta** | **Lepidoptera** | **Erebidae** | ***Lithosia*** | ***quadra*** | **100** |
| 52 | Insecta | Lepidoptera | Erebidae | *Lithosia* | *quadra* | 99.31 |
| 1 | Insecta | Lepidoptera | Erebidae | *Lithosia* | *yuennanensis* | 99.31 |
| 1 | Insecta | Lepidoptera | Nepticulidae | *Stigmella* | *arbusculae* | 98.47 |
| 1 | Insecta | Lepidoptera | Tortricidae | *Thrincophora* | *lignigerana* | 97.92 |
| 3 | Insecta | Lepidoptera | Erebidae | *Apistosia* | *judas* | 97.92 |

FinPROTAX output

|  | Class | Order | Family | Genus | Species | Probability |
| --- | --- | --- | --- | --- | --- | --- |
|  | **Insecta** |  |  |  |  | **0.999993** |
|  | **Insecta** | **Lepidoptera** |  |  |  | **0.977943** |
|  | **Insecta** | **Lepidoptera** | **Erebidae** |  |  | **0.800852** |
|  | **Insecta** | **Lepidoptera** | **Erebidae** | ***Lithosia*** |  | **0.614752** |
|  | **Insecta** | **Lepidoptera** | **Erebidae** | ***Lithosia*** | ***quadra*** | **0.610579** |

11. *Lymantria dispar*, Linnaeus 1758

>11a

AATAGTGGGAACATCTCTAAGTTTACTAATTCGAGCTGAATTAGGGAATCCTGGATCATTAATCGGAGATGATCAAATTTATAATACTATTGTTACAGCTCATGCATTTATCATAATTTTTTTTATAGTTATACCAATTATAATT

BOLD output

| # | Class | Order | Family | Genus | Species | Match % |
| --- | --- | --- | --- | --- | --- | --- |
| 100 | **Insecta** | **Lepidoptera** | **Erebidae** | ***Lymantria*** | ***dispar*** | **99.31** |

FinPROTAX output

|  | Class | Order | Family | Genus | Species | Probability |
| --- | --- | --- | --- | --- | --- | --- |
|  | **Insecta** |  |  |  |  | **0.999987** |
|  | **Insecta** | **Lepidoptera** |  |  |  | **0.999277** |
|  | **Insecta** | **Lepidoptera** | **Erebidae** |  |  | **0.998341** |
|  | **Insecta** | **Lepidoptera** | **Erebidae** | ***Lymantria*** |  | **0.992190** |
|  | **Insecta** | **Lepidoptera** | **Erebidae** | ***Lymantria*** | ***dispar*** | **0.985455** |

>11b

AATAGTAGGAACATCTCTAAGTTTACTAATTCGAGCTGAATTAGGGAATCCTGGATCATTAATCGGAAATGATCAAATTTATAATACTATTGTTACAGCTCATGCATTTATCATAATTTTTTTTATAGTTATACCAATTATAATT

BOLD output

| # | Class | Order | Family | Genus | Species | Match % |
| --- | --- | --- | --- | --- | --- | --- |
| 100 | **Insecta** | **Lepidoptera** | **Erebidae** | ***Lymantria*** | ***dispar*** | **100** |

FinPROTAX output

|  | Class | Order | Family | Genus | Species | Probability |
| --- | --- | --- | --- | --- | --- | --- |
|  | **Insecta** |  |  |  |  | **0.999984** |
|  | **Insecta** | **Lepidoptera** |  |  |  | **0.999678** |
|  | **Insecta** | **Lepidoptera** | **Erebidae** |  |  | **0.999200** |
|  | **Insecta** | **Lepidoptera** | **Erebidae** | ***Lymantria*** |  | **0.990166** |
|  | **Insecta** | **Lepidoptera** | **Erebidae** | ***Lymantria*** | ***dispar*** | **0.983445** |

>11c

GAATAGTGGGAACATCTCTAAGTTTACTAATTCGAGCTGAATTAGGGAATCCTGGATCATTAATCGGAAATGATCAAATTTATAATACTATTGTTACAGCTCATGCATTTATCATAATTTTTTTATAGTTATACCAATTATAATT

BOLD output

| # | Class | Order | Family | Genus | Species | Match % |
| --- | --- | --- | --- | --- | --- | --- |
| 100 | **Insecta** | **Lepidoptera** | **Erebidae** | ***Lymantria*** | ***dispar*** | **100** |

FinPROTAX output

|  | Class | Order | Family | Genus | Species | Probability |
| --- | --- | --- | --- | --- | --- | --- |
|  | **Insecta** |  |  |  |  | **0.999993** |
|  | **Insecta** | **Lepidoptera** |  |  |  | **0.999829** |
|  | **Insecta** | **Lepidoptera** | **Erebidae** |  |  | **0.999754** |
|  | **Insecta** | **Lepidoptera** | **Erebidae** | ***Lymantria*** |  | **0.994017** |
|  | **Insecta** | **Lepidoptera** | **Erebidae** | ***Lymantria*** | ***dispar*** | **0.992859** |

>11d

AATAGTGGGAACATCTCTAAGTTTACTAATTCGAGCTGAATTAGGGAATCCTGGATCATTAATCGGAAATGATCAAATTTATAATACTATTGTTACAGCTCATGCATTTATCATAATTTTTTTTATAGTTATACCAATTATAATT

BOLD output

| # | Class | Order | Family | Genus | Species | Match % |
| --- | --- | --- | --- | --- | --- | --- |
| 100 | **Insecta** | **Lepidoptera** | **Erebidae** | ***Lymantria*** | ***dispar*** | **100** |

FinPROTAX output

|  | Class | Order | Family | Genus | Species | Probability |
| --- | --- | --- | --- | --- | --- | --- |
|  | **Insecta** |  |  |  |  | **0.999991** |
|  | **Insecta** | **Lepidoptera** |  |  |  | **0.999862** |
|  | **Insecta** | **Lepidoptera** | **Erebidae** |  |  | **0.999793** |
|  | **Insecta** | **Lepidoptera** | **Erebidae** | ***Lymantria*** |  | **0.994772** |
|  | **Insecta** | **Lepidoptera** | **Erebidae** | ***Lymantria*** | ***dispar*** | **0.993613** |

>11e

AATAGTGGGAACATCTCTAAGTTTACTAATTCGAGCTGAATTAGGAAATCCTGGATCATTAATCGGAAATGATCGAATTTATAATACTATTGTTACAGCTCATGCATTTATCATAATTTTTTTTATAGTTATACCAATTATAATT

BOLD output

| # | Class | Order | Family | Genus | Species | Match % |
| --- | --- | --- | --- | --- | --- | --- |
| 60 | **Insecta** | **Lepidoptera** | **Erebidae** | ***Lymantria*** | ***dispar*** | **99.31** |
| 1 | Insecta | Lepidoptera | Erebidae | *Lymantria* | *sp. AN-2017* | 99.31 |
| 2 | Insecta | Lepidoptera | Erebidae | *Lymantria* | *dispar* | 98.61 |
| 37 | Insecta | Lepidoptera | Erebidae | *Lymantria* | *umbrosa* | 98.61 |

FinPROTAX output

|  | Class | Order | Family | Genus | Species | Probability |
| --- | --- | --- | --- | --- | --- | --- |
|  | **Insecta** |  |  |  |  | **0.999988** |
|  | **Insecta** | **Lepidoptera** |  |  |  | **0.999586** |
|  | **Insecta** | **Lepidoptera** | **Erebidae** |  |  | **0.999117** |
|  | **Insecta** | **Lepidoptera** | **Erebidae** | ***Lymantria*** |  | **0.984135** |
|  | **Insecta** | **Lepidoptera** | **Erebidae** | ***Lymantria*** | ***dispar*** | **0.946246** |

>11f

AATAGTGGGAACATCTCTAAGTTTACTAATTCGAGCTGAATTAGGGAATCCTGGATCATTAATCGGAAATGATCAAATTTATAATACTATTGTTACAGCTCATGCATTTATCATAATTTTTTTTATAGTTATACCTATTATAATT

BOLD output

| # | Class | Order | Family | Genus | Species | Match % |
| --- | --- | --- | --- | --- | --- | --- |
| 100 | **Insecta** | **Lepidoptera** | **Erebidae** | ***Lymantria*** | ***dispar*** | **99.31** |

FinPROTAX output

|  | Class | Order | Family | Genus | Species | Probability |
| --- | --- | --- | --- | --- | --- | --- |
|  | **Insecta** |  |  |  |  | **0.999989** |
|  | **Insecta** | **Lepidoptera** |  |  |  | **0.999791** |
|  | **Insecta** | **Lepidoptera** | **Erebidae** |  |  | **0.999584** |
|  | **Insecta** | **Lepidoptera** | **Erebidae** | ***Lymantria*** |  | **0.986837** |
|  | **Insecta** | **Lepidoptera** | **Erebidae** | ***Lymantria*** | ***dispar*** | **0.980138** |

>11g

AATAGTGGGAACATCTCTAAGTTTACTAATTCGAGCTGAATTAGGGAATCCTGGATCATTAATCGGAAATGATCAAATTTATAATACTATTGTTACAGCTCATGCGTTTATCATAATTTTTTTTATAGTTATACCAATTATAATT

BOLD output

| # | Class | Order | Family | Genus | Species | Match % |
| --- | --- | --- | --- | --- | --- | --- |
| 100 | **Insecta** | **Lepidoptera** | **Erebidae** | ***Lymantria*** | ***dispar*** | **99.31** |

FinPROTAX output

|  | Class | Order | Family | Genus | Species | Probability |
| --- | --- | --- | --- | --- | --- | --- |
|  | **Insecta** |  |  |  |  | **0.999989** |
|  | **Insecta** | **Lepidoptera** |  |  |  | **0.999690** |
|  | **Insecta** | **Lepidoptera** | **Erebidae** |  |  | **0.999439** |
|  | **Insecta** | **Lepidoptera** | **Erebidae** | ***Lymantria*** |  | **0.993909** |
|  | **Insecta** | **Lepidoptera** | **Erebidae** | ***Lymantria*** | ***dispar*** | **0.987163** |

>11h

GAATAGTGGGAACATCTCTAAGTTTACTAATTCGAGCTGAATTAGGGAATCCTGGATCATTAATCGGAAATGATAAAATTTATAATACTATTGTTACAGCTCATGCATTTATCATAATTTTTTTATAGTTATACCAATTATAATT

BOLD output

| # | Class | Order | Family | Genus | Species | Match % |
| --- | --- | --- | --- | --- | --- | --- |
| 100 | **Insecta** | **Lepidoptera** | **Erebidae** | ***Lymantria*** | ***dispar*** | **99.3** |

FinPROTAX output

|  | Class | Order | Family | Genus | Species | Probability |
| --- | --- | --- | --- | --- | --- | --- |
|  | **Insecta** |  |  |  |  | **0.999993** |
|  | **Insecta** | **Lepidoptera** |  |  |  | **0.999829** |
|  | **Insecta** | **Lepidoptera** | **Erebidae** |  |  | **0.999753** |
|  | **Insecta** | **Lepidoptera** | **Erebidae** | ***Lymantria*** |  | **0.993672** |
|  | **Insecta** | **Lepidoptera** | **Erebidae** | ***Lymantria*** | ***dispar*** | **0.987009** |

>11i

AATAGTGGGAACATCTCTAAGTTTACTAATTCGAGCTGAATTAGGAAATCCTGGATCATTAATCGGAAATGATCAAATTTATAATACTATTGTTACAGCTCATGCATTTATCATAATTTTTTTTATAGTTATACCAATTATAATT

BOLD output

| # | Class | Order | Family | Genus | Species | Match % |
| --- | --- | --- | --- | --- | --- | --- |
| 60 | **Insecta** | **Lepidoptera** | **Erebidae** | ***Lymantria*** | ***dispar*** | **100** |
| 1 | Insecta | Lepidoptera | Erebidae | *Lymantria* | *sp. AN-2017* | 100 |
| 2 | Insecta | Lepidoptera | Erebidae | *Lymantria* | *dispar* | 99.31 |
| 37 | Insecta | Lepidoptera | Erebidae | *Lymantria* | *umbrosa* | 99.31 |

FinPROTAX output

|  | Class | Order | Family | Genus | Species | Probability |
| --- | --- | --- | --- | --- | --- | --- |
|  | **Insecta** |  |  |  |  | **0.999989** |
|  | **Insecta** | **Lepidoptera** |  |  |  | **0.999586** |
|  | **Insecta** | **Lepidoptera** | **Erebidae** |  |  | **0.999116** |
|  | **Insecta** | **Lepidoptera** | **Erebidae** | ***Lymantria*** |  | **0.984669** |
|  | **Insecta** | **Lepidoptera** | **Erebidae** | ***Lymantria*** | ***dispar*** | **0.977985** |

>11j

GAATAGTGGGAACATCTCTAAGTTTACTAATTCGAGCTGAATTAGGAAATCCTGGATCATTAATCGGAAATGATCAAATTTATAATACTATTGTTACAGCTCATGCATTTATCATAATTTTTTTATAGTTATACCAATTATAATT

BOLD output

| # | Class | Order | Family | Genus | Species | Match % |
| --- | --- | --- | --- | --- | --- | --- |
| 60 | **Insecta** | **Lepidoptera** | **Erebidae** | ***Lymantria*** | ***dispar*** | **100** |
| 1 | Insecta | Lepidoptera | Erebidae | *Lymantria* | *sp. AN-2017* | 100 |
| 2 | Insecta | Lepidoptera | Erebidae | *Lymantria* | *dispar* | 99.3 |
| 37 | Insecta | Lepidoptera | Erebidae | *Lymantria* | *umbrosa* | 99.3 |

FinPROTAX output

|  | Class | Order | Family | Genus | Species | Probability |
| --- | --- | --- | --- | --- | --- | --- |
|  | **Insecta** |  |  |  |  | **0.999992** |
|  | **Insecta** | **Lepidoptera** |  |  |  | **0.999562** |
|  | **Insecta** | **Lepidoptera** | **Erebidae** |  |  | **0.999057** |
|  | **Insecta** | **Lepidoptera** | **Erebidae** | ***Lymantria*** |  | **0.982679** |
|  | **Insecta** | **Lepidoptera** | **Erebidae** | ***Lymantria*** | ***dispar*** | **0.976089** |

12. *Lymantria monacha*, Linnaeus 1758

>12a

TATAGTAGGAACTTCTCTTAGTTTATTAATCCGAGCTGAATTAGGAAATCCAGGATCATTAATTGGAAATGATCAAATTTATAATACGATTGTAACAGCCCATGCTTTTATTATAATTTTCTTTATAGTTATACCAATTATAATT

BOLD output

| # | Class | Order | Family | Genus | Species | Match % |
| --- | --- | --- | --- | --- | --- | --- |
| 91 | **Insecta** | **Lepidoptera** | **Erebidae** | ***Lymantria*** | ***monacha*** | **99.31** |
| 4 | Insecta | Lepidoptera | Erebidae | *Lymantria* | *monacha* | 99.3 |
| 1 | Insecta | Lepidoptera | Erebidae | *Lymantria* | *monacha* | 99.28 |
| 4 | Insecta | Lepidoptera | Erebidae | *Lymantria* | *monacha* | 98.61 |

FinPROTAX output

|  | Class | Order | Family | Genus | Species | Probability |
| --- | --- | --- | --- | --- | --- | --- |
|  | **Insecta** |  |  |  |  | **0.999997** |
|  | **Insecta** | **Lepidoptera** |  |  |  | **0.997517** |
|  | **Insecta** | **Lepidoptera** | **Erebidae** |  |  | **0.996947** |
|  | **Insecta** | **Lepidoptera** | **Erebidae** | ***Lymantria*** |  | **0.985414** |
|  | **Insecta** | **Lepidoptera** | **Erebidae** | ***Lymantria*** | ***monacha*** | **0.978725** |

>12b

TATAGTAGGAACTTCTCTTAGTTTATTAATCCGAGCTGAATTAGGAAATCCAGGATCATTAATTGGAAATGATCAAATTTATAATACGATTGTAACAGCTCATGCTTTTATTATAATTTTCTTTATAGTTATACCAATTATAATT

BOLD output

| # | Class | Order | Family | Genus | Species | Match % |
| --- | --- | --- | --- | --- | --- | --- |
| 96 | **Insecta** | **Lepidoptera** | **Erebidae** | ***Lymantria*** | ***monacha*** | **100** |
| 4 | Insecta | Lepidoptera | Erebidae | *Lymantria* | *monacha* | 99.31 |

FinPROTAX output

|  | Class | Order | Family | Genus | Species | Probability |
| --- | --- | --- | --- | --- | --- | --- |
|  | **Insecta** |  |  |  |  | **0.999998** |
|  | **Insecta** | **Lepidoptera** |  |  |  | **0.998205** |
|  | **Insecta** | **Lepidoptera** | **Erebidae** |  |  | **0.997651** |
|  | **Insecta** | **Lepidoptera** | **Erebidae** | ***Lymantria*** |  | **0.988649** |
|  | **Insecta** | **Lepidoptera** | **Erebidae** | ***Lymantria*** | ***monacha*** | **0.987497** |

>12c

TATAGTAAGAACTTCTCTTAGTTTATTAATCCGAGCTGAATTAGGAAATCCAGGATCATTAATTGGAAATGATCAAATTTATAATACGATTGTAACAGCTCATGCTTTTATTATAATTTTCTTTATAGTTATACCAATTATAATT

BOLD output

| # | Class | Order | Family | Genus | Species | Match % |
| --- | --- | --- | --- | --- | --- | --- |
| 91 | **Insecta** | **Lepidoptera** | **Erebidae** | ***Lymantria*** | ***monacha*** | **99.31** |
| 4 | Insecta | Lepidoptera | Erebidae | *Lymantria* | *monacha* | 99.3 |
| 1 | Insecta | Lepidoptera | Erebidae | *Lymantria* | *monacha* | 99.28 |
| 4 | Insecta | Lepidoptera | Erebidae | *Lymantria* | *monacha* | 98.61 |

FinPROTAX output

|  | Class | Order | Family | Genus | Species | Probability |
| --- | --- | --- | --- | --- | --- | --- |
|  | **Insecta** |  |  |  |  | **0.999997** |
|  | **Insecta** | **Lepidoptera** |  |  |  | **0.998205** |
|  | **Insecta** | **Lepidoptera** | **Erebidae** |  |  | **0.997651** |
|  | **Insecta** | **Lepidoptera** | **Erebidae** | ***Lymantria*** |  | **0.988439** |
|  | **Insecta** | **Lepidoptera** | **Erebidae** | ***Lymantria*** | ***monacha*** | **0.981729** |

>12d

TATAGTAGGAACTTCTCTTAGTTTATTAATCCGAGCTGAATTAGGAAATCCAGGATCATTAATTGGAAATGACCAAATTTATAATACGATTGTAACAGCTCATGCTTTTATTATAATTTTCTTTATAGTTATACCAATTATAATT

BOLD output

| # | Class | Order | Family | Genus | Species | Match % |
| --- | --- | --- | --- | --- | --- | --- |
| 91 | **Insecta** | **Lepidoptera** | **Erebidae** | ***Lymantria*** | ***monacha*** | **99.31** |
| 4 | Insecta | Lepidoptera | Erebidae | *Lymantria* | *monacha* | 99.3 |
| 1 | Insecta | Lepidoptera | Erebidae | *Lymantria* | *monacha* | 99.28 |
| 4 | Insecta | Lepidoptera | Erebidae | *Lymantria* | *monacha* | 98.61 |

FinPROTAX output

|  | Class | Order | Family | Genus | Species | Probability |
| --- | --- | --- | --- | --- | --- | --- |
|  | **Insecta** |  |  |  |  | **0.999997** |
|  | **Insecta** | **Lepidoptera** |  |  |  | **0.997567** |
|  | **Insecta** | **Lepidoptera** | **Erebidae** |  |  | **0.997017** |
|  | **Insecta** | **Lepidoptera** | **Erebidae** | ***Lymantria*** |  | **0.987997** |
|  | **Insecta** | **Lepidoptera** | **Erebidae** | ***Lymantria*** | ***monacha*** | **0.981291** |

13. *Malacosoma neustria*, Linnaeus 1758

>13

AATAGTTGGAACTTCACTAAGTTTATTAATTCGAGCAGAATTAGGTACTCCAGGGTCATTAATTGGAGATGACCAAATTTATAATACTATTGTAACAGCTCATGCTTTTATTATAATTTTTTTTATAGTTATACCAATTATAATT

BOLD output

| # | Class | Order | Family | Genus | Species | Match % |
| --- | --- | --- | --- | --- | --- | --- |
| 40 | **Insecta** | **Lepidoptera** | **Lasiocampidae** | ***Malacosoma*** | ***neustria*** | **100** |
| 13 | Insecta | Lepidoptera | Lasiocampidae | *Malacosoma* | *neustria* | 99.31 |
| 1 | Insecta | Lepidoptera | Nymphalidae | *Napeogenes* | *sylphis* | 98.89 |
| 13 | Insecta | Lepidoptera | Lasiocampidae | *Malacosoma* | *neustria* | 98.61 |
| 6 | Insecta | Lepidoptera | Lasiocampidae | *Malacosoma* | *neustria* | 97.92 |
| 3 | Insecta | Lepidoptera | Lasiocampidae | *Malacosoma* | *insignis* | 97.92 |
| 2 | Insecta | Lepidoptera | Nymphalidae | *Napeogenes* | *sylphis* | 97.85 |
| 22 | Insecta | Lepidoptera | Noctuidae | *Spodoptera* | *litura* | 97.85 |

FinPROTAX output

|  | Class | Order | Family | Genus | Species | Probability |
| --- | --- | --- | --- | --- | --- | --- |
|  | **Insecta** |  |  |  |  | **0.999998** |
|  | **Insecta** | **Lepidoptera** |  |  |  | **0.998241** |
|  | **Insecta** | **Lepidoptera** | **Lasiocampidae** |  |  | **0.992217** |
|  | **Insecta** | **Lepidoptera** | **Lasiocampidae** | ***Malacosoma*** |  | **0.980769** |
|  | **Insecta** | **Lepidoptera** | **Lasiocampidae** | ***Malacosoma*** | ***neustria*** | **0.979622** |

14. *Menophra abruptaria*, Thunberg 1792

>14

AATAGTGGGAACTTCTTTAAGATTATTAATTCGAGCTGAATTAGGTAACCCAGGATCACTAATTGGAGACGATCAAATTTATAATACTATTGTAACTGCCCATGCTTTTATTATAATTTTTTTTATGGTAATACCTATTATAATT

BOLD output

| # | Class | Order | Family | Genus | Species | Match % |
| --- | --- | --- | --- | --- | --- | --- |
| 5 | **Insecta** | **Lepidoptera** | **Geometridae** | ***Menophra*** | ***abruptaria*** | **100** |
| 23 | Insecta | Lepidoptera | Geometridae | *Menophra* | *abruptaria* | 99.31 |
| 6 | Insecta | Lepidoptera | Geometridae | *Menophra* | *abruptaria* | 98.61 |
| 1 | Insecta | Lepidoptera | Geometridae | *Argyrotome* | *subinquinataGM01* | 97.22 |
| 2 | Insecta | Lepidoptera | Geometridae | *Menophra* | *abruptaria* | 97.22 |
| 1 | Insecta | Lepidoptera | Geometridae | *Alcis* | *periphracta* | 96.53 |
| 1 | Insecta | Lepidoptera | Geometridae | *Phthonandria* | *indica* | 96.53 |
| 1 | Insecta | Lepidoptera | Erebidae | *Delphyre* | *orientalis* | 96.53 |
| 1 | Insecta | Lepidoptera | Geometridae | *Ophthalmoblysis* | *GM02* | 96.53 |
| 1 | Insecta | Lepidoptera | Geometridae | *Alcis* | *sp. ACA8528* | 96.53 |
| 2 | Insecta | Lepidoptera | Geometridae | *Menophra* | *abruptaria* | 96.53 |
| 2 | Insecta | Lepidoptera | Tortricidae | *Apotomis* | *capreana* | 96.53 |
| 1 | Insecta | Lepidoptera | Tortricidae | *Apotomis* | *infida* | 96.53 |
| 1 | Insecta | Lepidoptera | Geometridae | *Menophra* | *berenicidaria* | 96.53 |
| 6 | Insecta | Lepidoptera | Geometridae | *GeometridaeGEN* | *sp. ACL2840* | 96.53 |
| 1 | Insecta | Lepidoptera | Coleophoridae | *Coleophora* | *cornivorella* | 96.03 |
| 4 | Insecta | Lepidoptera | Geometridae | *Ourapteryx* | *contronivea* | 95.83 |
| 1 | Insecta | Lepidoptera | Geometridae | *Lomographa* | *sp.* | 95.83 |
| 5 | Insecta | Lepidoptera | Notodontidae | *Hemiceras* | *sabis* | 95.83 |
| 1 | Insecta | Lepidoptera | Crambidae | *Palpita* | *magniferalis* | 95.83 |
| 1 | Insecta | Lepidoptera | Geometridae | *Arichanna* | *AH01Vi* | 95.83 |
| 1 | Insecta | Lepidoptera | Tortricidae | *Phaneta* | *pauperana* | 95.83 |
| 3 | Insecta | Lepidoptera | Tortricidae | *Apotomis* | *algidana* | 95.83 |
| 1 | Insecta | Lepidoptera | Geometridae | *Pityeja* | *histrionariaGM04* | 95.83 |
| 1 | Insecta | Lepidoptera | Depressariidae | *Depressaria* | *pastukhovi* | 95.83 |
| 1 | Insecta | Lepidoptera | Erebidae | *Cuneisigna* | *cumamita* | 95.83 |
| 1 | Insecta | Lepidoptera | Erebidae | *Eugoa* | *nata* | 95.83 |
| 2 | Insecta | Lepidoptera | Geometridae | *Alcis* | *sp. ACA8528* | 95.83 |
| 2 | Insecta | Lepidoptera | Geometridae | *Menophra* | *abruptaria* | 95.83 |
| 12 | Insecta | Lepidoptera | Tortricidae | *Apotomis* | *capreana* | 95.83 |
| 9 | Insecta | Lepidoptera | Tortricidae | *Apotomis* | *infida* | 95.83 |

FinPROTAX output

|  | Class | Order | Family | Genus | Species | Probability |
| --- | --- | --- | --- | --- | --- | --- |
|  | **Insecta** |  |  |  |  | **0.999964** |
|  | Insecta | Coleoptera |  |  |  | 0.155666 |
|  | **Insecta** | **Lepidoptera** |  |  |  | **0.817593** |
|  | **Insecta** | **Lepidoptera** | **Geometridae** |  |  | **0.146577** |
|  | Insecta | Lepidoptera | Noctuidae |  |  | 0.183402 |
|  | Insecta | Lepidoptera | Tortricidae |  |  | 0.447365 |
|  | Insecta | Lepidoptera | Tortricidae | *Apotomis* |  | 0.162019 |
|  | Insecta | Lepidoptera | Tortricidae | *Apotomis* | unk | 0.158284 |

15. *Minucia lunaris*, Denis & Schiffermüller 1775

>15a

TATAGTAGGAACTTCTTTAAGTTTATTAATTCGAGCTGAATTAGGAAATCCAGGATCTTTAATCGGTGATGATCAAATTTATAACACTATTGTTACAGCTCATGCTTTTATTATAATTTTTTTTATAGTTATACCTATTATAATT

BOLD output

| # | Class | Order | Family | Genus | Species | Match % |
| --- | --- | --- | --- | --- | --- | --- |
| 1 | **Insecta** | **Lepidoptera** | **Erebidae** | ***Minucia*** | ***lunaris*** | **100** |
| 18 | Insecta | Lepidoptera | Erebidae | *Minucia* | *lunaris* | 99.31 |
| 1 | Insecta | Lepidoptera | Erebidae | *Minucia* | *lunaris* | 98.61 |
| 1 | Insecta | Lepidoptera | Nolidae | *Blenina* | *chloromelana* | 98.61 |
| 12 | Insecta | Lepidoptera | Noctuidae | *Hoplodrina* | *octogenaria* | 98.61 |
| 4 | Insecta | Lepidoptera | Geometridae | *Hammaptera* | *parinotata* | 98.61 |
| 5 | Insecta | Lepidoptera | Coleophoridae | *Coleophora* | *JFL275* | 97.92 |
| 13 | Insecta | Lepidoptera | Erebidae | *Composia* | *fidelissima* | 97.92 |
| 2 | Insecta | Lepidoptera | Noctuidae | *Parapamea* | *buffaloensis* | 97.92 |
| 5 | Insecta | Lepidoptera | Noctuidae | *Oxycnemis* | *grandimacula* | 97.92 |
| 1 | Insecta | Lepidoptera | Gelechiidae | *Trypanisma* | *prudens* | 97.92 |
| 1 | Insecta | Lepidoptera | Erebidae | *Ophiusa* | *legendrei* | 97.92 |
| 11 | Insecta | Lepidoptera | Notodontidae | *Lepasta* | *grammodes* | 97.92 |
| 1 | Insecta | Lepidoptera | Anthelidae | *Anthela* | *pudica* | 97.92 |
| 1 | Insecta | Lepidoptera | Erebidae | *Ophiusa* | *umbrilinea* | 97.92 |
| 1 | Insecta | Lepidoptera | Noctuidae | *Hemipachnobia* | *monochromatea* | 97.92 |
| 1 | Insecta | Lepidoptera | Noctuidae | *Lophoptera* | *conspicua* | 97.92 |
| 4 | Insecta | Lepidoptera | Noctuidae | *Hoplodrina* | *superstes* | 97.92 |
| 17 | Insecta | Lepidoptera | Noctuidae | *Hoplodrina* | *octogenaria* | 97.92 |

FinPROTAX output

|  | Class | Order | Family | Genus | Species | Probability |
| --- | --- | --- | --- | --- | --- | --- |
|  | **Insecta** |  |  |  |  | **0.999991** |
|  | **Insecta** | **Lepidoptera** |  |  |  | **0.989809** |
|  | **Insecta** | **Lepidoptera** | **Erebidae** |  |  | **0.607266** |
|  | **Insecta** | **Lepidoptera** | **Erebidae** | ***Minucia*** |  | **0.543668** |
|  | **Insecta** | **Lepidoptera** | **Erebidae** | ***Minucia*** | ***lunaris*** | **0.522737** |
|  | Insecta | Lepidoptera | Noctuidae |  |  | 0.342247 |

>15b

TATAGTAGGAACTTCTTTAAGTTTATTAATTCGAGCTGAATTAGGAAATCCAGGATCTTTAATCGGTGATGATCAAATTTATAATACTATTGTTACAGCTCATGCTTTTATTATAATTTTTTTTATAGTTATACCTATTATAATT

BOLD output

| # | Class | Order | Family | Genus | Species | Match % |
| --- | --- | --- | --- | --- | --- | --- |
| 4 | **Insecta** | **Lepidoptera** | **Erebidae** | ***Minucia*** | ***lunaris*** | **100** |
| 1 | Insecta | Lepidoptera | Erebidae | *Minucia* | *lunaris* | 99.31 |
| 1 | Insecta | Lepidoptera | Nymphalidae | *Melinaea* | *marsaeus* | 98.81 |
| 1 | Insecta | Lepidoptera | Nymphalidae | *Heliconius* | *ethilla* | 98.77 |
| 3 | Insecta | Lepidoptera | Nymphalidae | *Heliconius* | *melpomene* | 98.77 |
| 1 | Insecta | Lepidoptera | Nymphalidae | *Heliconius* | *ethilla x Heliconius melpomene* | 98.75 |
| 1 | Insecta | Lepidoptera | Nymphalidae | *Melinaea* | *menophilus* | 98.75 |
| 1 | Insecta | Lepidoptera | Nymphalidae | *Heliconius* | *ethilla* | 98.73 |
| 1 | Insecta | Lepidoptera | Nymphalidae | *Paralasa* | *styx* | 98.72 |
| 14 | Insecta | Lepidoptera | Erebidae | *Minucia* | *lunaris* | 98.61 |
| 1 | Insecta | Lepidoptera | Coleophoridae | *Coleophora* | *JFL275* | 98.61 |
| 13 | Insecta | Lepidoptera | Erebidae | *Composia* | *fidelissima* | 98.61 |
| 2 | Insecta | Lepidoptera | Noctuidae | *Parapamea* | *buffaloensis* | 98.61 |
| 5 | Insecta | Lepidoptera | Noctuidae | *Oxycnemis* | *grandimacula* | 98.61 |
| 1 | Insecta | Lepidoptera | Gelechiidae | *Trypanisma* | *prudens* | 98.61 |
| 1 | Insecta | Lepidoptera | Erebidae | *Ophiusa* | *legendrei* | 98.61 |
| 11 | Insecta | Lepidoptera | Notodontidae | *Lepasta* | *grammodes* | 98.61 |
| 1 | Insecta | Lepidoptera | Anthelidae | *Anthela* | *pudica* | 98.61 |
| 1 | Insecta | Lepidoptera | Erebidae | *Ophiusa* | *umbrilinea* | 98.61 |
| 1 | Insecta | Lepidoptera | Noctuidae | *Hemipachnobia* | *monochromatea* | 98.61 |
| 1 | Insecta | Lepidoptera | Noctuidae | *Lophoptera* | *conspicua* | 98.61 |
| 4 | Insecta | Lepidoptera | Noctuidae | *Hoplodrina* | *superstes* | 98.61 |
| 30 | Insecta | Lepidoptera | Noctuidae | *Hoplodrina* | *octogenaria* | 98.61 |

FinPROTAX output

|  | Class | Order | Family | Genus | Species | Probability |
| --- | --- | --- | --- | --- | --- | --- |
|  | **Insecta** |  |  |  |  | **0.999993** |
|  | **Insecta** | **Lepidoptera** |  |  |  | **0.975471** |
|  | Insecta | Lepidoptera | Noctuidae |  |  | 0.788032 |

16. *Ocneria rubea*, Denis & Schiffermüller 1775

>16a

TATAGTTGGAACATCATTAAGATTATTAATTCGAGCTGAATTAGGTAATCCCGGATCTTTAATTGGCAGCGATCAAATTTATAATACTATTGTTACAGCCCATGCTTTTATTATAATCTTTTTTATAGTAATACCTATTATAATT

BOLD output

| # | Class | Order | Family | Genus | Species | Match % |
| --- | --- | --- | --- | --- | --- | --- |
| 3 | **Insecta** | **Lepidoptera** | **Erebidae** | ***Ocneria*** | ***rubea*** | **99.31** |
| 1 | Insecta | Lepidoptera | Erebidae | *Ocneria* | *rubeaOE01Tu* | 98.61 |
| 1 | Insecta | Lepidoptera | Gelechiidae | *Gnorimoschema* | *nordlandicolella* | 95.14 |
| 1 | Insecta | Lepidoptera | Noctuidae | *Azenia* | *implora* | 95.12 |
| 13 | Insecta | Lepidoptera | Noctuidae | *Panolis* | *flammea* | 94.44 |
| 1 | Insecta | Lepidoptera | Gracillariidae | *Parornix* | *acuta* | 94.44 |
| 16 | Insecta | Lepidoptera | Noctuidae | *Lithophane* | *furcifera* | 94.44 |
| 3 | Insecta | Lepidoptera | Gelechiidae | *Gnorimoschema* | *saphirinella* | 94.44 |
| 2 | Insecta | Lepidoptera | Erebidae | *Peridrome* | *subfascia* | 94.44 |
| 4 | Insecta | Lepidoptera | Erebidae | *Ocneria* | *rubea* | 94.44 |
| 4 | Insecta | Lepidoptera | Depressariidae | *Agonopterix* | *rosaciliella* | 94.44 |
| 1 | Insecta | Lepidoptera | Erebidae | *Simplicia* | *robustalis* | 94.44 |
| 1 | Insecta | Lepidoptera | Thyrididae | *Aglaopus* | *centiginosa* | 94.44 |
| 28 | Insecta | Lepidoptera | Noctuidae | *Ammoconia* | *caecimacula* | 94.44 |
| 1 | Insecta | Lepidoptera | Noctuidae | *Procus* | *hypoxantha* | 94.44 |
| 1 | Insecta | Lepidoptera | Depressariidae | *Agonopterix* | *ciliella* | 94.44 |
| 1 | Insecta | Lepidoptera | Depressariidae | *Agonopterix* | *scopariella* | 94.44 |
| 10 | Insecta | Lepidoptera | Noctuidae | *Azenia* | *implora* | 94.44 |
| 1 | Insecta | Lepidoptera | Noctuidae | *Hypotrix* | *basistriga* | 94.44 |
| 7 | Insecta | Lepidoptera | Oecophoridae | *Artiastis* | *stenopolia* | 94.44 |

FinPROTAX output

|  | Class | Order | Family | Genus | Species | Probability |
| --- | --- | --- | --- | --- | --- | --- |
|  | **Insecta** |  |  |  |  | **0.999956** |
|  | **Insecta** | **Lepidoptera** |  |  |  | **0.970993** |
|  | Insecta | Lepidoptera | Gelechiidae |  |  | 0.534283 |
|  | Insecta | Lepidoptera | Gelechiidae | *Gnorimoschema* |  | 0.279705 |
|  | Insecta | Lepidoptera | Gelechiidae | *Gnorimoschema* | unk | 0.279220 |
|  | Insecta | Lepidoptera | Noctuidae |  |  | 0.306939 |

>16b

TATAGTTGGAACATCATTAAGATTATTAATTCGAGCTGAATTAGGTAATCCCGGATCTTTAATTGGCAACGATCAAATTTATAATACTATTGTTACAGCCCATGCTTTTATTATAATCTTTTTTATAGTAATACCTATTATAATT

BOLD output

| # | Class | Order | Family | Genus | Species | Match % |
| --- | --- | --- | --- | --- | --- | --- |
| 3 | **Insecta** | **Lepidoptera** | **Erebidae** | ***Ocneria*** | ***rubea*** | **100** |
| 1 | Insecta | Lepidoptera | Erebidae | *Ocneria* | *rubeaOE01Tu* | 99.31 |
| 1 | Insecta | Lepidoptera | Noctuidae | *Azenia* | *implora* | 95.93 |
| 1 | Insecta | Lepidoptera | Gelechiidae | *Gnorimoschema* | *nordlandicolella* | 95.83 |
| 13 | Insecta | Lepidoptera | Noctuidae | *Panolis* | *flammea* | 95.14 |
| 1 | Insecta | Lepidoptera | Gracillariidae | *Parornix* | *acuta* | 95.14 |
| 16 | Insecta | Lepidoptera | Noctuidae | *Lithophane* | *furcifera* | 95.14 |
| 3 | Insecta | Lepidoptera | Gelechiidae | *Gnorimoschema* | *saphirinella* | 95.14 |
| 2 | Insecta | Lepidoptera | Erebidae | *Peridrome* | *subfascia* | 95.14 |
| 4 | Insecta | Lepidoptera | Erebidae | *Ocneria* | *rubea* | 95.14 |
| 4 | Insecta | Lepidoptera | Depressariidae | *Agonopterix* | *rosaciliella* | 95.14 |
| 1 | Insecta | Lepidoptera | Erebidae | *Simplicia* | *robustalis* | 95.14 |
| 1 | Insecta | Lepidoptera | Thyrididae | *Aglaopus* | *centiginosa* | 95.14 |
| 28 | Insecta | Lepidoptera | Noctuidae | *Ammoconia* | *caecimacula* | 95.14 |
| 1 | Insecta | Lepidoptera | Noctuidae | *Procus* | *hypoxantha* | 95.14 |
| 1 | Insecta | Lepidoptera | Depressariidae | *Agonopterix* | *ciliella* | 95.14 |
| 1 | Insecta | Lepidoptera | Depressariidae | *Agonopterix* | *scopariella* | 95.14 |
| 10 | Insecta | Lepidoptera | Noctuidae | *Azenia* | *implora* | 95.14 |
| 1 | Insecta | Lepidoptera | Noctuidae | *Hypotrix* | *basistriga* | 95.14 |
| 7 | Insecta | Lepidoptera | Oecophoridae | *Artiastis* | *stenopolia* | 95.14 |

FinPROTAX output

|  | Class | Order | Family | Genus | Species | Probability |
| --- | --- | --- | --- | --- | --- | --- |
|  | **Insecta** |  |  |  |  | **0.999958** |
|  | **Insecta** | **Lepidoptera** |  |  |  | **0.971004** |
|  | Insecta | Lepidoptera | Gelechiidae |  |  | 0.534290 |
|  | Insecta | Lepidoptera | Gelechiidae | *Gnorimoschema* |  | 0.279790 |
|  | Insecta | Lepidoptera | Gelechiidae | *Gnorimoschema* | unk | 0.276974 |
|  | Insecta | Lepidoptera | Noctuidae |  |  | 0.306943 |

>16c

TATAGTTGGAACATCATTAAGATTATTAATTCGAGCTGAATTAGGTAATCCCGGATCTTTAATTGGCAACGATCAAATTTATAATACTATTGTTACAGCTCATGCTTTTATTATAATCTTTTTTATAGTAATACCTATTATAATT

BOLD output

| # | Class | Order | Family | Genus | Species | Match % |
| --- | --- | --- | --- | --- | --- | --- |
| 3 | **Insecta** | **Lepidoptera** | **Erebidae** | ***Ocneria*** | ***rubea*** | **99.31** |
| 1 | Insecta | Lepidoptera | Erebidae | *Ocneria* | *rubeaOE01Tu* | 98.61 |
| 1 | Insecta | Lepidoptera | Gracillariidae | *Parornix* | *acuta* | 95.83 |
| 16 | Insecta | Lepidoptera | Noctuidae | *Lithophane* | *furcifera* | 95.83 |
| 2 | Insecta | Lepidoptera | Erebidae | *Peridrome* | *subfascia* | 95.83 |
| 4 | Insecta | Lepidoptera | Erebidae | *Ocneria* | *rubea* | 95.83 |
| 1 | Insecta | Lepidoptera | Thyrididae | *Aglaopus* | *centiginosa* | 95.83 |
| 1 | Insecta | Lepidoptera | Noctuidae | *Procus* | *hypoxantha* | 95.83 |
| 14 | Insecta | Lepidoptera | Erebidae | *Peridrome* | *orbicularis* | 95.83 |
| 2 | Insecta | Lepidoptera | Thyrididae | *Aglaopus* | *centiginosa* | 95.77 |
| 1 | Insecta | Lepidoptera | Erebidae | *Catocala* | *andromache* | 95.54 |
| 1 | Insecta | Lepidoptera | Nymphalidae | *Calisto* | *pulchella* | 95.4 |
| 1 | Insecta | Lepidoptera | Depressariidae | *Agonopterix* | *sp.* | 95.14 |
| 6 | Insecta | Lepidoptera | Noctuidae | *Panolis* | *flammea* | 95.14 |
| 1 | Insecta | Lepidoptera | Geometridae | *Somatina* | *wiltshirei* | 95.14 |
| 5 | Insecta | Lepidoptera | Yponomeutidae | *Paraswammerdamia* | *nebulella* | 95.14 |
| 5 | Insecta | Lepidoptera | Erebidae | *Catocala* | *moltrechti* | 95.14 |
| 1 | Insecta | Lepidoptera | Geometridae | *Charissa* | *AH01Uz* | 95.14 |
| 4 | Insecta | Lepidoptera | Uraniidae | *Trotorhombia* | *metachromata* | 95.14 |
| 1 | Insecta | Lepidoptera | Gelechiidae | *Gnorimoschema* | *nordlandicolella* | 95.14 |
| 8 | Insecta | Lepidoptera | Gelechiidae | *Aristotelia* | *roseosuffusella* | 95.14 |
| 1 | Insecta | Lepidoptera | Geometridae | *Trimetopia* | *HS01SA* | 95.14 |
| 1 | Insecta | Lepidoptera | Gelechiidae | *Altenia* | *scriptella* | 95.14 |
| 1 | Insecta | Lepidoptera | Erebidae | *Asota* | *speciosa* | 95.14 |
| 1 | Insecta | Lepidoptera | Erebidae | *Nodaria* | *nodosalis* | 95.14 |
| 2 | Insecta | Lepidoptera | Erebidae | *Stenhypena* | *albopunctata* | 95.14 |
| 5 | Insecta | Lepidoptera | Noctuidae | *Neumichtis* | *signata* | 95.14 |
| 1 | Insecta | Lepidoptera | Noctuidae | *Neumichtis* | *adamantina* | 95.14 |
| 1 | Insecta | Lepidoptera | Limacodidae | *Avatara* | *basifusca* | 95.14 |
| 1 | Insecta | Lepidoptera | Erebidae | *Auchmophanes* | *sp. ANIC1* | 95.14 |
| 1 | Insecta | Lepidoptera | Erebidae | *Lacera* | *WS01Th* | 95.14 |
| 2 | Insecta | Lepidoptera | Geometridae | *Melinodes* | *fulvitincta group* | 95.14 |
| 4 | Insecta | Lepidoptera | Gelechiidae | *Gnorimoschema* | *crypticum* | 95.14 |

FinPROTAX output

|  | Class | Order | Family | Genus | Species | Probability |
| --- | --- | --- | --- | --- | --- | --- |
|  | **Insecta** |  |  |  |  | **0.999964** |
|  | **Insecta** | **Lepidoptera** |  |  |  | **0.962917** |
|  | Insecta | Lepidoptera | Noctuidae |  |  | 0.647424 |
|  | Insecta | Lepidoptera | Noctuidae | *Lithophane* |  | 0.203108 |
|  | Insecta | Lepidoptera | Noctuidae | *Lithophane* | unk | 0.200891 |
|  | Insecta | Lepidoptera | Tortricidae |  |  | 0.159468 |

>16d

TATAGTTGGAACATCATTAAGATTATTAATTCGAGCTGAATTAGGTAATCCCGGATCTTTAATTGGCAACGATCAAATTTATAATACTATTGTTACAGCCCATGCTTTTATTATAATCTTTTTTATAGTTATACCTATTATAATT

BOLD output

| # | Class | Order | Family | Genus | Species | Match % |
| --- | --- | --- | --- | --- | --- | --- |
| 3 | **Insecta** | **Lepidoptera** | **Erebidae** | ***Ocneria*** | ***rubea*** | **99.31** |
| 1 | Insecta | Lepidoptera | Erebidae | *Ocneria* | *rubeaOE01Tu* | 98.61 |
| 1 | Insecta | Lepidoptera | Gelechiidae | *Gnorimoschema* | *nordlandicolella* | 96.53 |
| 1 | Insecta | Lepidoptera | Gracillariidae | *Parornix* | *acuta* | 95.83 |
| 16 | Insecta | Lepidoptera | Noctuidae | *Lithophane* | *furcifera* | 95.83 |
| 4 | Insecta | Lepidoptera | Depressariidae | *Agonopterix* | *rosaciliella* | 95.83 |
| 1 | Insecta | Lepidoptera | Depressariidae | *Agonopterix* | *ciliella* | 95.83 |
| 1 | Insecta | Lepidoptera | Noctuidae | *Hypotrix* | *basistriga* | 95.83 |
| 2 | Insecta | Lepidoptera | Gelechiidae | *Gnorimoschema* | *altaica* | 95.83 |
| 1 | Insecta | Lepidoptera | Geometridae | *Scopula* | *adelpharia* | 95.77 |
| 1 | Insecta | Lepidoptera | Lycaenidae | *Phengaris* | *nausithous* | 95.6 |
| 66 | Insecta | Lepidoptera | Lycaenidae | *Phengaris* | *nausithous* | 95.56 |
| 2 | Insecta | Lepidoptera | Lycaenidae | *Phengaris* | *nausithous* | 95.51 |

FinPROTAX output

|  | Class | Order | Family | Genus | Species | Probability |
| --- | --- | --- | --- | --- | --- | --- |
|  | **Insecta** |  |  |  |  | **0.999980** |
|  | **Insecta** | **Lepidoptera** |  |  |  | **0.989039** |
|  | Insecta | Lepidoptera | Gelechiidae |  |  | 0.602172 |
|  | Insecta | Lepidoptera | Gelechiidae | *Caryocolum* |  | 0.126256 |
|  | Insecta | Lepidoptera | Gelechiidae | *Caryocolum* | unk | 0.126249 |
|  | Insecta | Lepidoptera | Gelechiidae | *Gnorimoschema* |  | 0.356918 |
|  | Insecta | Lepidoptera | Gelechiidae | *Gnorimoschema* | unk | 0.336857 |
|  | Insecta | Lepidoptera | Noctuidae |  |  | 0.345940 |
|  | Insecta | Lepidoptera | Noctuidae | *Lithophane* |  | 0.100281 |

17. *Peridea anceps*, Goeze 1781

>17

TATAGTAGGAACCTCTTTAAGTCTTTTAATTCGGGCTGAATTAGGAAATCCAGGTTCATTAATCGGAGATGATCAAATTTATAATACTATTGTTACAGCTCATGCTTTTATTATAATTTTTTTTATAGTTATACCTATTATAATT

BOLD output

| # | Class | Order | Family | Genus | Species | Match % |
| --- | --- | --- | --- | --- | --- | --- |
| 8 | **Insecta** | **Lepidoptera** | **Notodontidae** | ***Peridea*** | ***anceps*** | **100** |
| 1 | Insecta | Lepidoptera | Nymphalidae | *Heliconius* | *ethilla x Heliconius melpomene* | 100 |
| 2 | Insecta | Lepidoptera | Nymphalidae | *Heliconius* | *ethilla* | 100 |
| 3 | Insecta | Lepidoptera | Nymphalidae | *Heliconius* | *melpomene* | 100 |
| 1 | Insecta | Lepidoptera | Nymphalidae | *Melinaea* | *menophilus* | 100 |
| 1 | Insecta | Lepidoptera | Nymphalidae | *Melinaea* | *marsaeus* | 100 |
| 2 | Insecta | Lepidoptera | Nymphalidae | *Melinaea* | *menophilus* | 98.89 |
| 1 | Insecta | Lepidoptera | Nymphalidae | *Melinaea* | *menophilus* | 98.86 |
| 1 | Insecta | Lepidoptera | Nymphalidae | *Melinaea* | *satevis* | 98.86 |
| 1 | Insecta | Lepidoptera | Nymphalidae | *Oeneis* | *sculda* | 98.85 |
| 1 | Insecta | Lepidoptera | Nymphalidae | *Heliconius* | *elevatus* | 98.77 |
| 1 | Insecta | Lepidoptera | Nymphalidae | *Heliconius* | *ethilla* | 98.77 |
| 1 | Insecta | Lepidoptera | Nymphalidae | *Heliconius* | *hecale* | 98.77 |
| 1 | Insecta | Lepidoptera | Noctuidae | *Pseudobryomima* | *sp. KLKDNA0340* | 98.77 |
| 1 | Insecta | Lepidoptera | Nymphalidae | *Paralasa* | *styx* | 98.72 |
| 6 | Insecta | Lepidoptera | Notodontidae | *Peridea* | *anceps* | 97.92 |
| 19 | Insecta | Lepidoptera | Nymphalidae | *Melinaea* | *menophilus* | 97.85 |
| 15 | Insecta | Lepidoptera | Nymphalidae | *Heliconius* | *melpomene* | 97.85 |
| 4 | Insecta | Lepidoptera | Nymphalidae | *Heliconius* | *numata* | 97.85 |
| 27 | Insecta | Lepidoptera | Nymphalidae | *Melinaea* | *marsaeus* | 97.85 |
| 3 | Insecta | Lepidoptera | Nymphalidae | *Heliconius* | *timareta* | 97.85 |

FinPROTAX output

|  | Class | Order | Family | Genus | Species | Probability |
| --- | --- | --- | --- | --- | --- | --- |
|  | **Insecta** |  |  |  |  | **0.999969** |
|  | Insecta | Diptera |  |  |  | 0.122318 |
|  | **Insecta** | **Lepidoptera** |  |  |  | **0.837024** |
|  | Insecta | Lepidoptera | Gelechiidae |  |  | 0.175695 |
|  | Insecta | Lepidoptera | Noctuidae |  |  | 0.150545 |
|  | Insecta | Lepidoptera | Tortricidae |  |  | 0.367219 |
|  | Insecta | Lepidoptera | Tortricidae | *Aethes* |  | 0.164375 |
|  | Insecta | Lepidoptera | Tortricidae | *Aethes* | unk | 0.163009 |

18. *Rileyiana fovea*, Treitschke 1825

ASVs assigned to *Rileyiana fovea* have equally likely matches to *Dryobota labecula* (Esper 1788) in all cases. This is rarely the case for allogeneric sequences, but it was not possible to investigate this case further since most *R. fovea* and all *D. labecula* matches that were suggested belonged to non-public records. As full overlap with a single reference sequence for *R. fovea* could be confirmed, the relevant ASVs will be presented as assigned to *R. fovea*. Importantly, *R. fovea* and *D. labecula* are similarly non-local species; both have distant distributions in central Europe.

>18a

AATAGTAGGAACTTCCTTAAGATTATTAATTCGAGCCGAATTAGGAAATCCCGGATCTTTAATTGGAGATGATCAAATTTATAATACTATTGTTACAGCACATGCTTTTATTACAATTTTTTTTATAGTTATACCTATTATAATT

BOLD output

| # | Class | Order | Family | Genus | Species | Match % |
| --- | --- | --- | --- | --- | --- | --- |
| 3 | **Insecta** | **Lepidoptera** | **Noctuidae** | ***Rileyiana*** | ***fovea*** | **99.31** |
| 4 | **Insecta** | **Lepidoptera** | **Noctuidae** | ***Dryobota*** | ***labecula*** | **99.31** |
| 22 | Insecta | Lepidoptera | Noctuidae | *Parabagrotis* | *sulinaris* | 97.92 |
| 1 | Insecta | Lepidoptera | Noctuidae | *Eugnorisma* | *pontica* | 97.92 |
| 1 | Insecta | Diptera | Conopidae | *Stylogaster* | *inca* | 97.81 |
| 1 | Insecta | Lepidoptera | Nymphalidae | *Oeneis* | *sculda* | 97.7 |
| 1 | Insecta | Lepidoptera | Nymphalidae | *Heliconius* | *ethilla* | 97.53 |
| 3 | Insecta | Lepidoptera | Nymphalidae | *Heliconius* | *melpomene* | 97.53 |
| 1 | Insecta | Lepidoptera | Nymphalidae | *Heliconius* | *ethilla x Heliconius melpomene* | 97.5 |
| 1 | Insecta | Lepidoptera | Nymphalidae | *Melinaea* | *menophilus* | 97.5 |
| 1 | Insecta | Lepidoptera | Nymphalidae | *Heliconius* | *ethilla* | 97.47 |
| 7 | Insecta | Lepidoptera | Noctuidae | *Paradiarsia* | *punicea* | 97.22 |
| 51 | Insecta | Lepidoptera | Noctuidae | *Lacanobia* | *oleracea* | 97.22 |
| 3 | Insecta | Lepidoptera | Noctuidae | *Cerastis* | *leucographa* | 97.22 |

FinPROTAX output

|  | Class | Order | Family | Genus | Species | Probability |
| --- | --- | --- | --- | --- | --- | --- |
|  | **Insecta** |  |  |  |  | **0.999988** |
|  | **Insecta** | **Lepidoptera** |  |  |  | **0.956079** |
|  | Insecta | Lepidoptera | Depressariidae |  |  | 0.110277 |
|  | **Insecta** | **Lepidoptera** | **Noctuidae** |  |  | **0.681027** |

>18b

AATAGTAGGAACTTCCTTAAGATTATTAATTCGAGCCGAATTAGGAAATCCCGGATCTTTAATTGGAGATGATCAAATTTATAATACTATTGTTACAGCACATGCTTTTATTATAATTTTTTTTATAGTTATACCTATTATAATT

BOLD output

| # | Class | Order | Family | Genus | Species | Match % |
| --- | --- | --- | --- | --- | --- | --- |
| 3 | **Insecta** | **Lepidoptera** | **Noctuidae** | ***Rileyiana*** | ***fovea*** | **100** |
| 4 | **Insecta** | **Lepidoptera** | **Noctuidae** | ***Dryobota*** | ***labecula*** | **100** |
| 1 | Insecta | Lepidoptera | Nymphalidae | *Oeneis* | *sculda* | 98.85 |
| 1 | Insecta | Lepidoptera | Nymphalidae | *Heliconius* | *ethilla* | 98.77 |
| 3 | Insecta | Lepidoptera | Nymphalidae | *Heliconius* | *melpomene* | 98.77 |
| 1 | Insecta | Lepidoptera | Nymphalidae | *Heliconius* | *ethilla x Heliconius melpomene* | 98.75 |
| 1 | Insecta | Lepidoptera | Nymphalidae | *Melinaea* | *menophilus* | 98.75 |
| 1 | Insecta | Lepidoptera | Nymphalidae | *Heliconius* | *ethilla* | 98.73 |
| 22 | Insecta | Lepidoptera | Noctuidae | *Parabagrotis* | *sulinaris* | 98.61 |
| 1 | Insecta | Lepidoptera | Noctuidae | *Eugnorisma* | *pontica* | 98.61 |
| 1 | Insecta | Diptera | Conopidae | *Stylogaster* | *inca* | 98.54 |
| 1 | Insecta | Lepidoptera | Depressariidae | *Acria* | *sp.* | 98.15 |
| 1 | Insecta | Lepidoptera | Depressariidae | *Agonopterix* | *l-nigrum* | 98.15 |
| 7 | Insecta | Lepidoptera | Noctuidae | *Paradiarsia* | *punicea* | 97.92 |
| 51 | Insecta | Lepidoptera | Noctuidae | *Lacanobia* | *oleracea* | 97.92 |
| 1 | Insecta | Lepidoptera | Noctuidae | *Cerastis* | *leucographa* | 97.92 |

FinPROTAX output

|  | Class | Order | Family | Genus | Species | Probability |
| --- | --- | --- | --- | --- | --- | --- |
|  | **Insecta** |  |  |  |  | **0.999989** |
|  | **Insecta** | **Lepidoptera** |  |  |  | **0.956080** |
|  | Insecta | Lepidoptera | Depressariidae |  |  | 0.110277 |
|  | **Insecta** | **Lepidoptera** | **Noctuidae** |  |  | **0.681028** |

>18c

AATAGTAGGAACTTCCTTAAGATTATTAATTCGAGCCGAATTAGGAAATCCCGGATCTTTAATTGGAGATGATCAAATTTATAATACTATTGTTACAGCACATGCTTTTATTATAATTTTTTTTATAGTTATACCTATTATAATC

BOLD output

| # | Class | Order | Family | Genus | Species | Match % |
| --- | --- | --- | --- | --- | --- | --- |
| 3 | **Insecta** | **Lepidoptera** | **Noctuidae** | ***Rileyiana*** | ***fovea*** | **99.31** |
| 4 | **Insecta** | **Lepidoptera** | **Noctuidae** | ***Dryobota*** | ***labecula*** | **99.31** |
| 1 | Insecta | Lepidoptera | Nymphalidae | *Melitaea* | *arduinna* | 98.15 |
| 22 | Insecta | Lepidoptera | Noctuidae | *Parabagrotis* | *sulinaris* | 97.92 |
| 1 | Insecta | Lepidoptera | Noctuidae | *Eugnorisma* | *pontica* | 97.92 |
| 1 | Insecta | Lepidoptera | Noctuidae | *Copablepharon* | *nevada* | 97.92 |
| 1 | Insecta | Lepidoptera | Noctuidae | *Xestia* | *infimatis* | 97.92 |
| 10 | Insecta | Lepidoptera | Noctuidae | *Xestia* | *finatimis* | 97.92 |
| 6 | Insecta | Lepidoptera | Noctuidae | *Xestia* | *vernilis* | 97.92 |
| 1 | Insecta | Diptera | Conopidae | *Stylogaster* | *inca* | 97.81 |
| 1 | Insecta | Lepidoptera | Nymphalidae | *Cymothoe* | *egesta* | 97.7 |
| 1 | Insecta | Lepidoptera | Nymphalidae | *Oeneis* | *sculda* | 97.7 |
| 2 | Insecta | Lepidoptera | Nymphalidae | *Cymothoe* | *egesta* | 97.62 |
| 1 | Insecta | Lepidoptera | Nymphalidae | *Heliconius* | *ethilla* | 97.53 |
| 3 | Insecta | Lepidoptera | Nymphalidae | *Heliconius* | *melpomene* | 97.53 |
| 1 | Insecta | Lepidoptera | Nymphalidae | *Heliconius* | *ethilla x Heliconius melpomene* | 97.5 |
| 1 | Insecta | Lepidoptera | Nymphalidae | *Melinaea* | *menophilus* | 97.5 |
| 1 | Insecta | Lepidoptera | Nymphalidae | *Heliconius* | *ethilla* | 97.47 |
| 23 | Insecta | Lepidoptera | Noctuidae | *Xestia* | *sexstrigata* | 97.22 |
| 7 | Insecta | Lepidoptera | Noctuidae | *Paradiarsia* | *punicea* | 97.22 |
| 9 | Insecta | Lepidoptera | Noctuidae | *Lacanobia* | *oleracea* | 97.22 |

FinPROTAX output

|  | Class | Order | Family | Genus | Species | Probability |
| --- | --- | --- | --- | --- | --- | --- |
|  | **Insecta** |  |  |  |  | **0.999990** |
|  | **Insecta** | **Lepidoptera** |  |  |  | **0.957271** |
|  | Insecta | Lepidoptera | Depressariidae |  |  | 0.110889 |
|  | **Insecta** | **Lepidoptera** | **Noctuidae** |  |  | **0.683529** |

>18d

AATAGTAGGAACTTCCTTAAGATTATTAATTCGAGCCGAATTAGGAAATCCCGGATCTTTAATTGGAGATGATCAAATTTATAATACTATTGTTACAGCACATGCTTTTATTATAATTTTTTTTATAGTAATACCTATTATAATT

BOLD output

| # | Class | Order | Family | Genus | Species | Match % |
| --- | --- | --- | --- | --- | --- | --- |
| 3 | **Insecta** | **Lepidoptera** | **Noctuidae** | ***Rileyiana*** | ***fovea*** | **99.31** |
| 4 | **Insecta** | **Lepidoptera** | **Noctuidae** | ***Dryobota*** | ***labecula*** | **99.31** |
| 6 | Insecta | Lepidoptera | Noctuidae | *Panolis* | *flammea* | 97.92 |
| 1 | Insecta | Lepidoptera | Geometridae | *Gnophos* | *rjabovi* | 97.92 |
| 1 | Insecta | Lepidoptera | Euteliidae | *Eutelia* | *abscondens* | 97.92 |
| 4 | Insecta | Lepidoptera | Noctuidae | *Agrotis* | *interjectionis* | 97.92 |
| 4 | Insecta | Lepidoptera | Tortricidae | *Adoxophyes* | *thoracica* | 97.92 |
| 22 | Insecta | Lepidoptera | Noctuidae | *Parabagrotis* | *sulinaris* | 97.92 |
| 2 | Insecta | Lepidoptera | Noctuidae | *Abagrotis* | *striata* | 97.92 |
| 1 | Insecta | Lepidoptera | Noctuidae | *Agrotis* | *obesa* | 97.92 |
| 4 | Insecta | Lepidoptera | Noctuidae | *Xestia* | *ashworthii* | 97.92 |
| 4 | Insecta | Lepidoptera | Geometridae | *Charissa* | *ali* | 97.92 |
| 1 | Insecta | Lepidoptera | Erebidae | *Hyphoraia* | *testudinaria* | 97.92 |
| 1 | Insecta | Lepidoptera | Noctuidae | *Eugnorisma* | *pontica* | 97.92 |
| 34 | Insecta | Lepidoptera | Noctuidae | *Xestia* | *badicollis* | 97.92 |
| 8 | Insecta | Lepidoptera | Noctuidae | *Xestia* | *praevia* | 97.92 |

FinPROTAX output

|  | Class | Order | Family | Genus | Species | Probability |
| --- | --- | --- | --- | --- | --- | --- |
|  | **Insecta** |  |  |  |  | **0.999984** |
|  | **Insecta** | **Lepidoptera** |  |  |  | **0.904087** |
|  | **Insecta** | **Lepidoptera** | **Noctuidae** |  |  | **0.723052** |
|  | Insecta | Lepidoptera | Noctuidae | *Xestia* |  | 0.198348 |
|  | Insecta | Lepidoptera | Noctuidae | *Xestia* | *ashworthii* | 0.113183 |

>18e

GAATAGTAGGAACTTCCTTAAGATTATTAATTCGAGCCGAATTAGGAAATCCCGGATCTTTAATTGGAGATGATCAAATTTATAATACTATTGTTACAGCACATGCTTTTATTATAATTTTTTTATAGTTATACCTATTATAATT

BOLD output

| # | Class | Order | Family | Genus | Species | Match % |
| --- | --- | --- | --- | --- | --- | --- |
| 3 | **Insecta** | **Lepidoptera** | **Noctuidae** | ***Rileyiana*** | ***fovea*** | **100** |
| 4 | **Insecta** | **Lepidoptera** | **Noctuidae** | ***Dryobota*** | ***labecula*** | **100** |
| 1 | Insecta | Lepidoptera | Nymphalidae | *Heteropsis* | *ankoma* | 98.91 |
| 1 | Insecta | Lepidoptera | Nymphalidae | *Oeneis* | *sculda* | 98.84 |
| 1 | Insecta | Lepidoptera | Nymphalidae | *Heliconius* | *ethilla* | 98.75 |
| 3 | Insecta | Lepidoptera | Nymphalidae | *Heliconius* | *melpomene* | 98.75 |
| 1 | Insecta | Lepidoptera | Nymphalidae | *Heliconius* | *ethilla x Heliconius melpomene* | 98.73 |
| 1 | Insecta | Lepidoptera | Nymphalidae | *Melinaea* | *menophilus* | 98.73 |
| 1 | Insecta | Lepidoptera | Nymphalidae | *Heliconius* | *ethilla* | 98.72 |
| 22 | Insecta | Lepidoptera | Noctuidae | *Parabagrotis* | *sulinaris* | 98.6 |
| 1 | Insecta | Lepidoptera | Noctuidae | *Eugnorisma* | *pontica* | 98.6 |
| 1 | Insecta | Diptera | Conopidae | *Stylogaster* | *inca* | 98.53 |
| 1 | Insecta | Lepidoptera | Depressariidae | *Acria* | *sp.* | 98.13 |
| 1 | Insecta | Lepidoptera | Depressariidae | *Agonopterix* | *l-nigrum* | 98.13 |
| 7 | Insecta | Lepidoptera | Noctuidae | *Paradiarsia* | *punicea* | 97.9 |
| 51 | Insecta | Lepidoptera | Noctuidae | *Lacanobia* | *oleracea* | 97.9 |

FinPROTAX output

|  | Class | Order | Family | Genus | Species | Probability |
| --- | --- | --- | --- | --- | --- | --- |
|  | **Insecta** |  |  |  |  | **0.999992** |
|  | **Insecta** | **Lepidoptera** |  |  |  | **0.927339** |
|  | **Insecta** | **Lepidoptera** | **Noctuidae** |  |  | **0.668829** |
|  | Insecta | Lepidoptera | Noctuidae | *Xestia* |  | 0.103236 |

>18f

AATAGTGGGAACTTCCTTAAGATTATTAATTCGAGCCGAATTAGGAAATCCCGGATCTTTAATTGGAGATGATCAAATTTATAATACTATTGTTACAGCACATGCTTTTATTATAATTTTTTTTATAGTTATACCTATTATAATT

BOLD output

| # | Class | Order | Family | Genus | Species | Match % |
| --- | --- | --- | --- | --- | --- | --- |
| 3 | **Insecta** | **Lepidoptera** | **Noctuidae** | ***Rileyiana*** | ***fovea*** | **99.31** |
| 4 | **Insecta** | **Lepidoptera** | **Noctuidae** | ***Dryobota*** | ***labecula*** | **99.31** |
| 1 | Insecta | Lepidoptera | Nymphalidae | *Oeneis* | *sculda* | 98.85 |
| 1 | Insecta | Lepidoptera | Nymphalidae | *Heliconius* | *ethilla* | 98.77 |
| 3 | Insecta | Lepidoptera | Nymphalidae | *Heliconius* | *melpomene* | 98.77 |
| 1 | Insecta | Lepidoptera | Nymphalidae | *Heliconius* | *ethilla x Heliconius melpomene* | 98.75 |
| 1 | Insecta | Lepidoptera | Nymphalidae | *Melinaea* | *menophilus* | 98.75 |
| 1 | Insecta | Lepidoptera | Nymphalidae | *Heliconius* | *ethilla* | 98.73 |
| 1 | Insecta | Lepidoptera | Depressariidae | *Acria* | *sp.* | 98.15 |
| 1 | Insecta | Lepidoptera | Depressariidae | *Agonopterix* | *l-nigrum* | 98.15 |
| 22 | Insecta | Lepidoptera | Noctuidae | *Parabagrotis* | *sulinaris* | 97.92 |
| 4 | Insecta | Lepidoptera | Noctuidae | *Anaplectoides* | *pressus* | 97.92 |
| 1 | Insecta | Lepidoptera | Noctuidae | *Cerastis* | *robertsoni* | 97.92 |
| 3 | Insecta | Lepidoptera | Noctuidae | *Ochropleura* | *sidamoGB01* | 97.92 |
| 1 | Insecta | Lepidoptera | Erebidae | *Cyana* | *flavalba* | 97.92 |
| 1 | Insecta | Lepidoptera | Noctuidae | *Eugnorisma* | *pontica* | 97.92 |
| 9 | Insecta | Lepidoptera | Noctuidae | *Orthosia* | *garmani* | 97.92 |
| 1 | Insecta | Lepidoptera | Geometridae | *Eudrepanulatrix* | *rectifascia* | 97.92 |
| 2 | Insecta | Lepidoptera | Noctuidae | *Eugraphe* | *sigma* | 97.92 |
| 1 | Insecta | Lepidoptera | Noctuidae | *Parabagrotis* | *cupidissima* | 97.92 |
| 1 | Insecta | Lepidoptera | Riodinidae | *Stalachtis* | *euterpe* | 97.89 |
| 1 | Insecta | Lepidoptera | Noctuidae | *Helicoverpa* | *gelotopoeon* | 97.85 |
| 4 | Insecta | Lepidoptera | Noctuidae | *Spodoptera* | *dolichos* | 97.85 |
| 15 | Insecta | Lepidoptera | Nymphalidae | *Heliconius* | *melpomene* | 97.85 |
| 4 | Insecta | Lepidoptera | Nymphalidae | *Heliconius* | *numata* | 97.85 |
| 2 | Insecta | Lepidoptera | Noctuidae | *Spodoptera* | *pulchella* | 97.85 |
| 11 | Insecta | Lepidoptera | Nymphalidae | *Heliconius* | *timareta* | 97.85 |

FinPROTAX output

|  | Class | Order | Family | Genus | Species | Probability |
| --- | --- | --- | --- | --- | --- | --- |
|  | **Insecta** |  |  |  |  | **0.999984** |
|  | **Insecta** | **Lepidoptera** |  |  |  | **0.948791** |
|  | **Insecta** | **Lepidoptera** | **Noctuidae** |  |  | **0.677965** |

>18g

AATAGTAGGAACTTCCTTAAGATTATTAATTCGAGCCGAATTAGGAAATCACGGATCTTTAATTGGAGATGATCAAATTTATAATACTATTGTTACAGCACATGCTTTTATTATAATTTTTTTTATAGTTATACCTATTATAATT

BOLD output

| # | Class | Order | Family | Genus | Species | Match % |
| --- | --- | --- | --- | --- | --- | --- |
| 3 | **Insecta** | **Lepidoptera** | **Noctuidae** | ***Rileyiana*** | ***fovea*** | **99.31** |
| 4 | **Insecta** | **Lepidoptera** | **Noctuidae** | ***Dryobota*** | ***labecula*** | **99.31** |
| 1 | Insecta | Lepidoptera | Nymphalidae | *Oeneis* | *sculda* | 98.85 |
| 1 | Insecta | Lepidoptera | Nymphalidae | *Heliconius* | *ethilla* | 98.77 |
| 3 | Insecta | Lepidoptera | Nymphalidae | *Heliconius* | *melpomene* | 98.77 |
| 1 | Insecta | Lepidoptera | Nymphalidae | *Heliconius* | *ethilla x Heliconius melpomene* | 98.75 |
| 1 | Insecta | Lepidoptera | Nymphalidae | *Melinaea* | *menophilus* | 98.75 |
| 1 | Insecta | Lepidoptera | Nymphalidae | *Heliconius* | *ethilla* | 98.73 |
| 22 | Insecta | Lepidoptera | Noctuidae | *Parabagrotis* | *sulinaris* | 97.92 |
| 1 | Insecta | Lepidoptera | Noctuidae | *Eugnorisma* | *pontica* | 97.92 |
| 1 | Insecta | Lepidoptera | Noctuidae | *Helicoverpa* | *gelotopoeon* | 97.85 |
| 4 | Insecta | Lepidoptera | Noctuidae | *Spodoptera* | *dolichos* | 97.85 |
| 15 | Insecta | Lepidoptera | Nymphalidae | *Heliconius* | *melpomene* | 97.85 |
| 8 | Insecta | Lepidoptera | Nymphalidae | *Heliconius* | *numata* | 97.85 |
| 2 | Insecta | Lepidoptera | Noctuidae | *Spodoptera* | *pulchella* | 97.85 |
| 11 | Insecta | Lepidoptera | Nymphalidae | *Heliconius* | *timareta* | 97.85 |
| 1 | Insecta | Lepidoptera | Nymphalidae | *Brevioleria* | *arzalia* | 97.85 |
| 2 | Insecta | Lepidoptera | Nymphalidae | *Hyposcada* | *illinissa* | 97.85 |
| 1 | Insecta | Lepidoptera | Nymphalidae | *Heteropsis* | *ankoma* | 97.85 |
| 1 | Insecta | Lepidoptera | Nymphalidae | *Hyposcada* | *illinissa* | 97.83 |
| 1 | Insecta | Diptera | Conopidae | *Stylogaster* | *inca* | 97.81 |
| 1 | Insecta | Lepidoptera | Nymphalidae | *Hyposcada* | *illinissa* | 97.73 |
| 1 | Insecta | Lepidoptera | Nymphalidae | *Calisto* | *pulchella* | 97.7 |
| 1 | Insecta | Lepidoptera | Papilionidae | *Pachliopta* | *aristolochiae* | 97.7 |
| 1 | Insecta | Lepidoptera | Nymphalidae | *Heliconius* | *numata* | 97.7 |
| 1 | Insecta | Lepidoptera | Noctuidae | *Spodoptera* | *frugiperda* | 97.7 |
| 1 | Insecta | Lepidoptera | Nymphalidae | *Bicyclus* | *rhacotis* | 97.7 |
| 1 | Insecta | Lepidoptera | Nymphalidae | *Melinaea* | *marsaeus* | 97.62 |
| 1 | Insecta | Lepidoptera | Geometridae | *Racotis* | *boarmiaria* | 97.62 |
| 1 | Insecta | Lepidoptera | Nymphalidae | *Heliconius* | *elevatus* | 97.53 |
| 1 | Insecta | Lepidoptera | Nymphalidae | *Heliconius* | *ethilla* | 97.53 |
| 1 | Insecta | Lepidoptera | Lycaenidae | *Polyommatus* | *dizinensis* | 97.53 |
| 1 | Insecta | Lepidoptera | Nymphalidae | *Heliconius* | *hecale* | 97.53 |
| 1 | Insecta | Lepidoptera | Noctuidae | *Pseudobryomima* | *sp. KLKDNA0340* | 97.53 |
| 1 | Insecta | Lepidoptera | Nymphalidae | *Paralasa* | *styx* | 97.44 |
| 1 | Insecta | Lepidoptera | Nymphalidae | *Anthanassa* | *drusilla* | 97.44 |

FinPROTAX output

|  | Class | Order | Family | Genus | Species | Probability |
| --- | --- | --- | --- | --- | --- | --- |
|  | **Insecta** |  |  |  |  | **0.999988** |
|  | **Insecta** | **Lepidoptera** |  |  |  | **0.956077** |
|  | Insecta | Lepidoptera | Depressariidae |  |  | 0.110277 |
|  | **Insecta** | **Lepidoptera** | **Noctuidae** |  |  | **0.681025** |

>18h

AATAGTAGGAACTTCCTTAAGATTATTAATTCGAGCCGAATTAGGAAATCCCGGATCTTTAATTGGAGGTGATCAAATTTATAATACTATTGTTACAGCACATGCTTTTATTATAATTTTTTTTATAGTTATACCTATTATAATT

BOLD output

| # | Class | Order | Family | Genus | Species | Match % |
| --- | --- | --- | --- | --- | --- | --- |
| 3 | **Insecta** | **Lepidoptera** | **Noctuidae** | ***Rileyiana*** | ***fovea*** | **99.31** |
| 4 | **Insecta** | **Lepidoptera** | **Noctuidae** | ***Dryobota*** | ***labecula*** | **99.31** |
| 22 | Insecta | Lepidoptera | Noctuidae | *Parabagrotis* | *sulinaris* | 97.92 |
| 1 | Insecta | Lepidoptera | Noctuidae | *Eugnorisma* | *pontica* | 97.92 |
| 1 | Insecta | Diptera | Conopidae | *Stylogaster* | *inca* | 97.81 |
| 1 | Insecta | Lepidoptera | Nymphalidae | *Oeneis* | *sculda* | 97.7 |
| 1 | Insecta | Lepidoptera | Nymphalidae | *Heliconius* | *ethilla* | 97.53 |
| 3 | Insecta | Lepidoptera | Nymphalidae | *Heliconius* | *melpomene* | 97.53 |
| 1 | Insecta | Lepidoptera | Nymphalidae | *Heliconius* | *ethilla x Heliconius melpomene* | 97.5 |
| 1 | Insecta | Lepidoptera | Nymphalidae | *Melinaea* | *menophilus* | 97.5 |
| 1 | Insecta | Lepidoptera | Nymphalidae | *Heliconius* | *ethilla* | 97.47 |
| 7 | Insecta | Lepidoptera | Noctuidae | *Paradiarsia* | *punicea* | 97.22 |
| 51 | Insecta | Lepidoptera | Noctuidae | *Lacanobia* | *oleracea* | 97.22 |
| 3 | Insecta | Lepidoptera | Noctuidae | *Cerastis* | *leucographa* | 97.22 |

FinPROTAX output

|  | Class | Order | Family | Genus | Species | Probability |
| --- | --- | --- | --- | --- | --- | --- |
|  | **Insecta** |  |  |  |  | **0.999988** |
|  | **Insecta** | **Lepidoptera** |  |  |  | **0.956079** |
|  | Insecta | Lepidoptera | Depressariidae |  |  | 0.110277 |
|  | **Insecta** | **Lepidoptera** | **Noctuidae** |  |  | **0.681025** |

>18i

AATAGTAGGAACTTCCTTAAGATTATTAATTCGAGCCGAATTAGGAAATCCCGGATCCTTAATTGGAGATGATCAAATTTATAATACTATTGTTACAGCACATGCTTTTATTATAATTTTTTTTATAGTTATACCTATTATAATT

BOLD output

| # | Class | Order | Family | Genus | Species | Match % |
| --- | --- | --- | --- | --- | --- | --- |
| 3 | **Insecta** | **Lepidoptera** | **Noctuidae** | ***Rileyiana*** | ***fovea*** | **99.31** |
| 4 | **Insecta** | **Lepidoptera** | **Noctuidae** | ***Dryobota*** | ***labecula*** | **99.31** |
| 1 | Insecta | Lepidoptera | Nymphalidae | *Oeneis* | *sculda* | 98.85 |
| 1 | Insecta | Lepidoptera | Nymphalidae | *Heliconius* | *ethilla* | 98.77 |
| 3 | Insecta | Lepidoptera | Nymphalidae | *Heliconius* | *melpomene* | 98.77 |
| 1 | Insecta | Lepidoptera | Nymphalidae | *Heliconius* | *ethilla x Heliconius melpomene* | 98.75 |
| 1 | Insecta | Lepidoptera | Nymphalidae | *Melinaea* | *menophilus* | 98.75 |
| 1 | Insecta | Lepidoptera | Nymphalidae | *Heliconius* | *ethilla* | 98.73 |
| 1 | Insecta | Lepidoptera | Noctuidae | *Copablepharon* | *mustelini* | 98.61 |
| 22 | Insecta | Lepidoptera | Noctuidae | *Parabagrotis* | *sulinaris* | 97.92 |
| 1 | Insecta | Lepidoptera | Noctuidae | *Eugnorisma* | *pontica* | 97.92 |
| 34 | Insecta | Lepidoptera | Noctuidae | *Xestia* | *ursae* | 97.92 |
| 4 | Insecta | Lepidoptera | Noctuidae | *Copablepharon* | *mustelini* | 97.92 |
| 1 | Insecta | Lepidoptera | Noctuidae | *Pseudohermonassa* | *bicarnea* | 97.92 |
| 1 | Insecta | Lepidoptera | Noctuidae | *Pronoctua* | *pyrophiloides* | 97.92 |
| 19 | Insecta | Lepidoptera | Noctuidae | *Xestia* | *atrata* | 97.92 |
| 1 | Insecta | Lepidoptera | Noctuidae | *Xestia* | *gelida* | 97.86 |
| 1 | Insecta | Lepidoptera | Nymphalidae | *Heliconius* | *melpomene* | 97.85 |

FinPROTAX output

|  | Class | Order | Family | Genus | Species | Probability |
| --- | --- | --- | --- | --- | --- | --- |
|  | **Insecta** |  |  |  |  | **0.999989** |
|  | **Insecta** | **Lepidoptera** |  |  |  | **0.968700** |
|  | **Insecta** | **Lepidoptera** | **Noctuidae** |  |  | **0.863646** |
|  | Insecta | Lepidoptera | Noctuidae | *Xestia* |  | 0.288186 |
|  | Insecta | Lepidoptera | Noctuidae | *Xestia* | *atrata* | 0.145443 |

>18j

AATAGTAGGAACTTCCTTAAGATTATTAATTCGAGCCGAATTAGGAAATCCCAGATCTTTAATTGGAGATGATCAAATTTATAATACTATTGTTACAGCACATGCTTTTATTATAATTTTTTTTATAGTTATACCTATTATAATT

BOLD output

| # | Class | Order | Family | Genus | Species | Match % |
| --- | --- | --- | --- | --- | --- | --- |
| 3 | **Insecta** | **Lepidoptera** | **Noctuidae** | ***Rileyiana*** | ***fovea*** | **99.31** |
| 4 | **Insecta** | **Lepidoptera** | **Noctuidae** | ***Dryobota*** | ***labecula*** | **99.31** |
| 1 | Insecta | Lepidoptera | Nymphalidae | *Oeneis* | *sculda* | 98.85 |
| 1 | Insecta | Lepidoptera | Nymphalidae | *Heliconius* | *ethilla* | 98.77 |
| 3 | Insecta | Lepidoptera | Nymphalidae | *Heliconius* | *melpomene* | 98.77 |
| 1 | Insecta | Lepidoptera | Nymphalidae | *Heliconius* | *ethilla x Heliconius melpomene* | 98.75 |
| 1 | Insecta | Lepidoptera | Nymphalidae | *Melinaea* | *menophilus* | 98.75 |
| 1 | Insecta | Lepidoptera | Nymphalidae | *Heliconius* | *ethilla* | 98.73 |
| 22 | Insecta | Lepidoptera | Noctuidae | *Parabagrotis* | *sulinaris* | 97.92 |
| 1 | Insecta | Lepidoptera | Noctuidae | *Eugnorisma* | *pontica* | 97.92 |
| 1 | Insecta | Diptera | Conopidae | *Stylogaster* | *inca* | 97.81 |
| 1 | Insecta | Lepidoptera | Nymphalidae | *Calisto* | *pulchella* | 97.7 |
| 1 | Insecta | Lepidoptera | Papilionidae | *Pachliopta* | *aristolochiae* | 97.7 |
| 1 | Insecta | Lepidoptera | Nymphalidae | *Heliconius* | *numata* | 97.7 |
| 1 | Insecta | Lepidoptera | Noctuidae | *Spodoptera* | *frugiperda* | 97.7 |
| 1 | Insecta | Lepidoptera | Nymphalidae | *Bicyclus* | *rhacotis* | 97.7 |
| 1 | Insecta | Lepidoptera | Nymphalidae | *Melinaea* | *marsaeus* | 97.62 |
| 1 | Insecta | Lepidoptera | Geometridae | *Racotis* | *boarmiaria* | 97.62 |
| 1 | Insecta | Lepidoptera | Nymphalidae | *Heliconius* | *elevatus* | 97.53 |
| 1 | Insecta | Lepidoptera | Nymphalidae | *Heliconius* | *ethilla* | 97.53 |
| 1 | Insecta | Lepidoptera | Lycaenidae | *Polyommatus* | *dizinensis* | 97.53 |
| 1 | Insecta | Lepidoptera | Nymphalidae | *Heliconius* | *hecale* | 97.53 |
| 1 | Insecta | Lepidoptera | Noctuidae | *Pseudobryomima* | *sp. KLKDNA0340* | 97.53 |
| 1 | Insecta | Lepidoptera | Nymphalidae | *Paralasa* | *styx* | 97.44 |
| 1 | Insecta | Lepidoptera | Nymphalidae | *Anthanassa* | *drusilla* | 97.44 |
| 7 | Insecta | Lepidoptera | Noctuidae | *Paradiarsia* | *punicea* | 97.22 |
| 40 | Insecta | Lepidoptera | Noctuidae | *Lacanobia* | *oleracea* | 97.22 |

FinPROTAX output

|  | Class | Order | Family | Genus | Species | Probability |
| --- | --- | --- | --- | --- | --- | --- |
|  | **Insecta** |  |  |  |  | **0.999988** |
|  | **Insecta** | **Lepidoptera** |  |  |  | **0.956077** |
|  | Insecta | Lepidoptera | Depressariidae |  |  | 0.110267 |
|  | **Insecta** | **Lepidoptera** | **Noctuidae** |  |  | **0.680963** |

>18k

AATAGTAGGAACTTCCTTAAGATTATTAACTCGAGCCGAATTAGGAAATCCCGGATCTTTAATTGGAGATGATCAAATTTATAATACTATTGTTACAGCACATGCTTTTATTATAATTTTTTTTATAGTTATACCTATTATAATT

BOLD output

| # | Class | Order | Family | Genus | Species | Match % |
| --- | --- | --- | --- | --- | --- | --- |
| 3 | **Insecta** | **Lepidoptera** | **Noctuidae** | ***Rileyiana*** | ***fovea*** | **99.31** |
| 4 | **Insecta** | **Lepidoptera** | **Noctuidae** | ***Dryobota*** | ***labecula*** | **99.31** |
| 1 | Insecta | Lepidoptera | Nymphalidae | *Oeneis* | *sculda* | 98.85 |
| 1 | Insecta | Lepidoptera | Nymphalidae | *Heliconius* | *ethilla* | 98.77 |
| 3 | Insecta | Lepidoptera | Nymphalidae | *Heliconius* | *melpomene* | 98.77 |
| 1 | Insecta | Lepidoptera | Nymphalidae | *Heliconius* | *ethilla x Heliconius melpomene* | 98.75 |
| 1 | Insecta | Lepidoptera | Nymphalidae | *Melinaea* | *menophilus* | 98.75 |
| 1 | Insecta | Lepidoptera | Nymphalidae | *Heliconius* | *ethilla* | 98.73 |
| 1 | Insecta | Lepidoptera | Depressariidae | *Acria* | *sp.* | 98.15 |
| 1 | Insecta | Lepidoptera | Depressariidae | *Agonopterix* | *l-nigrum* | 98.15 |
| 22 | Insecta | Lepidoptera | Noctuidae | *Parabagrotis* | *sulinaris* | 97.92 |
| 1 | Insecta | Lepidoptera | Noctuidae | *Eugnorisma* | *pontica* | 97.92 |
| 1 | Insecta | Lepidoptera | Riodinidae | *Stalachtis* | *euterpe* | 97.89 |
| 1 | Insecta | Lepidoptera | Noctuidae | *Helicoverpa* | *gelotopoeon* | 97.85 |
| 4 | Insecta | Lepidoptera | Noctuidae | *Spodoptera* | *dolichos* | 97.85 |
| 15 | Insecta | Lepidoptera | Nymphalidae | *Heliconius* | *melpomene* | 97.85 |
| 8 | Insecta | Lepidoptera | Nymphalidae | *Heliconius* | *numata* | 97.85 |
| 2 | Insecta | Lepidoptera | Noctuidae | *Spodoptera* | *pulchella* | 97.85 |
| 11 | Insecta | Lepidoptera | Nymphalidae | *Heliconius* | *timareta* | 97.85 |
| 1 | Insecta | Lepidoptera | Nymphalidae | *Brevioleria* | *arzalia* | 97.85 |
| 2 | Insecta | Lepidoptera | Nymphalidae | *Hyposcada* | *illinissa* | 97.85 |
| 1 | Insecta | Lepidoptera | Nymphalidae | *Heteropsis* | *ankoma* | 97.85 |
| 1 | Insecta | Lepidoptera | Nymphalidae | *Hyposcada* | *illinissa* | 97.83 |
| 1 | Insecta | Diptera | Conopidae | *Stylogaster* | *inca* | 97.81 |
| 1 | Insecta | Lepidoptera | Nymphalidae | *Hyposcada* | *illinissa* | 97.73 |
| 1 | Insecta | Lepidoptera | Nymphalidae | *Calisto* | *pulchella* | 97.7 |
| 1 | Insecta | Lepidoptera | Papilionidae | *Pachliopta* | *aristolochiae* | 97.7 |
| 1 | Insecta | Lepidoptera | Nymphalidae | *Heliconius* | *numata* | 97.7 |
| 1 | Insecta | Lepidoptera | Noctuidae | *Spodoptera* | *frugiperda* | 97.7 |
| 1 | Insecta | Lepidoptera | Nymphalidae | *Bicyclus* | *rhacotis* | 97.7 |
| 1 | Insecta | Lepidoptera | Nymphalidae | *Melinaea* | *marsaeus* | 97.62 |
| 1 | Insecta | Lepidoptera | Geometridae | *Racotis* | *boarmiaria* | 97.62 |
| 1 | Insecta | Lepidoptera | Nymphalidae | *Heliconius* | *elevatus* | 97.53 |
| 1 | Insecta | Lepidoptera | Nymphalidae | *Heliconius* | *ethilla* | 97.53 |
| 1 | Insecta | Lepidoptera | Lycaenidae | *Polyommatus* | *dizinensis* | 97.53 |
| 1 | Insecta | Lepidoptera | Nymphalidae | *Heliconius* | *hecale* | 97.53 |

FinPROTAX output

|  | Class | Order | Family | Genus | Species | Probability |
| --- | --- | --- | --- | --- | --- | --- |
|  | **Insecta** |  |  |  |  | **0.999988** |
|  | **Insecta** | **Lepidoptera** |  |  |  | **0.956079** |
|  | Insecta | Lepidoptera | Depressariidae |  |  | 0.110277 |
|  | **Insecta** | **Lepidoptera** | **Noctuidae** |  |  | **0.681027** |

>18l

AATAGTAGGAACTTCCTTAAGATTATTAATTCGAGCCGAATTAGGAAATCCCGGATCTTTAATTGGAGATGATCAAATTTATAATGCTATTGTTACAGCACATGCTTTTATTATAATTTTTTTTATAGTTATACCTATTATAATT

BOLD output

| # | Class | Order | Family | Genus | Species | Match % |
| --- | --- | --- | --- | --- | --- | --- |
| 3 | **Insecta** | **Lepidoptera** | **Noctuidae** | ***Rileyiana*** | ***fovea*** | **99.31** |
| 4 | **Insecta** | **Lepidoptera** | **Noctuidae** | ***Dryobota*** | ***labecula*** | **99.31** |
| 22 | Insecta | Lepidoptera | Noctuidae | *Parabagrotis* | *sulinaris* | 97.92 |
| 1 | Insecta | Lepidoptera | Noctuidae | *Eugnorisma* | *pontica* | 97.92 |
| 1 | Insecta | Diptera | Conopidae | *Stylogaster* | *inca* | 97.81 |
| 1 | Insecta | Lepidoptera | Nymphalidae | *Oeneis* | *sculda* | 97.7 |
| 1 | Insecta | Lepidoptera | Nymphalidae | *Heliconius* | *ethilla* | 97.53 |
| 3 | Insecta | Lepidoptera | Nymphalidae | *Heliconius* | *melpomene* | 97.53 |
| 1 | Insecta | Lepidoptera | Nymphalidae | *Heliconius* | *ethilla x Heliconius melpomene* | 97.5 |
| 1 | Insecta | Lepidoptera | Nymphalidae | *Melinaea* | *menophilus* | 97.5 |
| 1 | Insecta | Lepidoptera | Nymphalidae | *Heliconius* | *ethilla* | 97.47 |
| 7 | Insecta | Lepidoptera | Noctuidae | *Paradiarsia* | *punicea* | 97.22 |
| 51 | Insecta | Lepidoptera | Noctuidae | *Lacanobia* | *oleracea* | 97.22 |
| 3 | Insecta | Lepidoptera | Noctuidae | *Cerastis* | *leucographa* | 97.22 |

FinPROTAX output

|  | Class | Order | Family | Genus | Species | Probability |
| --- | --- | --- | --- | --- | --- | --- |
|  | **Insecta** |  |  |  |  | **0.999977** |
|  | Insecta | Coleoptera |  |  |  | 0.106609 |
|  | **Insecta** | **Lepidoptera** |  |  |  | **0.864351** |
|  | **Insecta** | **Lepidoptera** | **Noctuidae** |  |  | **0.615682** |

>18m

AATAGTAGGAACTTCCTTAAGACTATTAATTCGAGCCGAATTAGGAAATCCCGGATCTTTAATTGGAGATGATCAAATTTATAATACTATTGTTACAGCACATGCTTTTATTATAATTTTTTTTATAGTTATACCTATTATAATT

BOLD output

| # | Class | Order | Family | Genus | Species | Match % |
| --- | --- | --- | --- | --- | --- | --- |
| 3 | **Insecta** | **Lepidoptera** | **Noctuidae** | ***Rileyiana*** | ***fovea*** | **99.31** |
| 4 | **Insecta** | **Lepidoptera** | **Noctuidae** | ***Dryobota*** | ***labecula*** | **99.31** |
| 1 | Insecta | Lepidoptera | Nymphalidae | *Oeneis* | *sculda* | 98.85 |
| 1 | Insecta | Lepidoptera | Nymphalidae | *Heliconius* | *ethilla* | 98.77 |
| 3 | Insecta | Lepidoptera | Nymphalidae | *Heliconius* | *melpomene* | 98.77 |
| 1 | Insecta | Lepidoptera | Nymphalidae | *Heliconius* | *ethilla x Heliconius melpomene* | 98.75 |
| 1 | Insecta | Lepidoptera | Nymphalidae | *Melinaea* | *menophilus* | 98.75 |
| 1 | Insecta | Lepidoptera | Nymphalidae | *Heliconius* | *ethilla* | 98.73 |
| 8 | Insecta | Lepidoptera | Noctuidae | *Parabagrotis* | *insularis* | 98.61 |
| 19 | Insecta | Lepidoptera | Noctuidae | *Parabagrotis* | *formalis* | 98.61 |
| 1 | Insecta | Lepidoptera | Noctuidae | *Parabagrotis* | *cupidissima* | 98.61 |
| 1 | Insecta | Lepidoptera | Depressariidae | *Acria* | *sp.* | 98.15 |
| 1 | Insecta | Lepidoptera | Depressariidae | *Agonopterix* | *l-nigrum* | 98.15 |
| 22 | Insecta | Lepidoptera | Noctuidae | *Parabagrotis* | *sulinaris* | 97.92 |
| 1 | Insecta | Lepidoptera | Noctuidae | *Eugnorisma* | *pontica* | 97.92 |
| 25 | Insecta | Lepidoptera | Noctuidae | *Parabagrotis* | *insularis* | 97.92 |
| 7 | Insecta | Lepidoptera | Noctuidae | *Parabagrotis* | *cupidissima* | 97.92 |

FinPROTAX output

|  | Class | Order | Family | Genus | Species | Probability |
| --- | --- | --- | --- | --- | --- | --- |
|  | **Insecta** |  |  |  |  | **0.999984** |
|  | **Insecta** | **Lepidoptera** |  |  |  | **0.903845** |
|  | Insecta | Lepidoptera | Depressariidae |  |  | 0.104343 |
|  | **Insecta** | **Lepidoptera** | **Noctuidae** |  |  | **0.644383** |

>18n

AATAGTAGGAACTTCCTTAAGATTATTAATTCGAGCCGAATTAGGAAATCCCGGATCTTTAATTGGCGATGATCAAATTTATAATACTATTGTTACAGCACATGCTTTTATTATAATTTTTTTTATAGTTATACCTATTATAATT

BOLD output

| # | Class | Order | Family | Genus | Species | Match % |
| --- | --- | --- | --- | --- | --- | --- |
| 3 | **Insecta** | **Lepidoptera** | **Noctuidae** | ***Rileyiana*** | ***fovea*** | **99.31** |
| 4 | **Insecta** | **Lepidoptera** | **Noctuidae** | ***Dryobota*** | ***labecula*** | **99.31** |
| 22 | Insecta | Lepidoptera | Noctuidae | *Parabagrotis* | *sulinaris* | 97.92 |
| 1 | Insecta | Lepidoptera | Noctuidae | *Eugnorisma* | *pontica* | 97.92 |
| 1 | Insecta | Diptera | Conopidae | *Stylogaster* | *inca* | 97.81 |
| 1 | Insecta | Lepidoptera | Nymphalidae | *Oeneis* | *sculda* | 97.7 |
| 1 | Insecta | Lepidoptera | Nymphalidae | *Heliconius* | *ethilla* | 97.53 |
| 3 | Insecta | Lepidoptera | Nymphalidae | *Heliconius* | *melpomene* | 97.53 |
| 1 | Insecta | Lepidoptera | Nymphalidae | *Heliconius* | *ethilla x Heliconius melpomene* | 97.5 |
| 1 | Insecta | Lepidoptera | Nymphalidae | *Melinaea* | *menophilus* | 97.5 |
| 1 | Insecta | Lepidoptera | Nymphalidae | *Heliconius* | *ethilla* | 97.47 |
| 1 | Insecta | Lepidoptera | Nymphalidae | *Paralasa* | *styx* | 97.44 |
| 1 | Insecta | Lepidoptera | Nymphalidae | *Anthanassa* | *drusilla* | 97.44 |
| 7 | Insecta | Lepidoptera | Noctuidae | *Paradiarsia* | *punicea* | 97.22 |
| 51 | Insecta | Lepidoptera | Noctuidae | *Lacanobia* | *oleracea* | 97.22 |
| 1 | Insecta | Lepidoptera | Noctuidae | *Cerastis* | *leucographa* | 97.22 |

FinPROTAX output

|  | Class | Order | Family | Genus | Species | Probability |
| --- | --- | --- | --- | --- | --- | --- |
|  | **Insecta** |  |  |  |  | **0.999984** |
|  | **Insecta** | **Lepidoptera** |  |  |  | **0.922024** |
|  | Insecta | Lepidoptera | Depressariidae |  |  | 0.106565 |
|  | **Insecta** | **Lepidoptera** | **Noctuidae** |  |  | **0.658103** |

19. *Satyrium esculi*, Hübner 1804

ASVs assigned to *Satyrium esculi* also have a single, equally good match to *Satyrium spini* (Denis & Schiffermüller 1775). The latter could not be verified since it was a private record, but based on the several dozen matches to *S. esculi* and the lack of any further *S. spini* matches (despite >100 reference sequences for it being available in BOLD), these ASVs were plausibly assigned to *S. esculi*. Note that *S. spini* would still have been a non-local match as well.

>19a

AATACTAGGAACATCTTTAAGAATTCTAATTCGAATAGAATTAGGAACACCAGGATCTTTAATTGGAGATGATCAAATTTATAATACCATCGTAACAGCTCATGCTTTTATCATAATTTTTTTTATAGTAATACCTATTATAATT

BOLD output

| # | Class | Order | Family | Genus | Species | Match % |
| --- | --- | --- | --- | --- | --- | --- |
| 38 | **Insecta** | **Lepidoptera** | **Lycaenidae** | ***Satyrium*** | ***esculi*** | **100** |
| 1 | **Insecta** | **Lepidoptera** | **Lycaenidae** | ***Satyrium*** | ***spini*** | **100** |
| 7 | Insecta | Lepidoptera | Lycaenidae | *Satyrium* | *esculi* | 99.31 |
| 6 | Insecta | Lepidoptera | Lycaenidae | *Satyrium* | *esculi* | 98.61 |
| 1 | Insecta | Lepidoptera | Nymphalidae | *Melitaea* | *didyma* | 97.22 |
| 47 | Insecta | Lepidoptera | Lycaenidae | *Satyrium* | *ilicis* | 97.22 |

FinPROTAX output

|  | Class | Order | Family | Genus | Species | Probability |
| --- | --- | --- | --- | --- | --- | --- |
|  | **Insecta** |  |  |  |  | **0.999886** |
|  | **Insecta** | **Lepidoptera** |  |  |  | **0.938490** |
|  | **Insecta** | **Lepidoptera** | **Lycaenidae** |  |  | **0.700636** |
|  | Insecta | Lepidoptera | Lycaenidae | *Scolitantides* |  | 0.313302 |
|  | Insecta | Lepidoptera | Lycaenidae | *Scolitantides* | unk | 0.313109 |

>19b

AATACTAGGAACATCTTTAAGAATTCTAATTCGAATAGAATTAGGAACGCCAGGATCTTTAATTGGAGATGATCAAATTTATAATACCATCGTAACAGCTCATGCTTTTATCATAATTTTTTTTATAGTAATACCTATTATAATT

BOLD output

| # | Class | Order | Family | Genus | Species | Match % |
| --- | --- | --- | --- | --- | --- | --- |
| 38 | **Insecta** | **Lepidoptera** | **Lycaenidae** | ***Satyrium*** | ***esculi*** | **99.31** |
| 1 | **Insecta** | **Lepidoptera** | **Lycaenidae** | ***Satyrium*** | ***spini*** | **99.31** |
| 7 | Insecta | Lepidoptera | Lycaenidae | *Satyrium* | *esculi* | 98.61 |
| 6 | Insecta | Lepidoptera | Lycaenidae | *Satyrium* | *esculi* | 97.92 |
| 1 | Insecta | Lepidoptera | Nymphalidae | *Melitaea* | *didyma* | 96.53 |
| 47 | Insecta | Lepidoptera | Lycaenidae | *Satyrium* | *ilicis* | 96.53 |

FinPROTAX output

|  | Class | Order | Family | Genus | Species | Probability |
| --- | --- | --- | --- | --- | --- | --- |
|  | **Insecta** |  |  |  |  | **0.999921** |
|  | **Insecta** | **Lepidoptera** |  |  |  | **0.929578** |
|  | **Insecta** | **Lepidoptera** | **Lycaenidae** |  |  | **0.693998** |
|  | Insecta | Lepidoptera | Lycaenidae | *Scolitantides* |  | 0.209406 |
|  | Insecta | Lepidoptera | Lycaenidae | *Scolitantides* | unk | 0.209384 |

>19c

AATACTAGGAACATCTTTAAGAATTCTAATTCGAATAGAATTAGGAACACCAGGATCTTTAATTGGAGATGATCAAATTTATAATACCATCGTAACAGCCCATGCTTTTATCATAATTTTTTTTATAGTAATACCTATTATAATT

BOLD output

| # | Class | Order | Family | Genus | Species | Match % |
| --- | --- | --- | --- | --- | --- | --- |
| 38 | **Insecta** | **Lepidoptera** | **Lycaenidae** | ***Satyrium*** | ***esculi*** | **99.31** |
| 1 | **Insecta** | **Lepidoptera** | **Lycaenidae** | ***Satyrium*** | ***spini*** | **99.31** |
| 7 | Insecta | Lepidoptera | Lycaenidae | *Satyrium* | *esculi* | 98.61 |
| 6 | Insecta | Lepidoptera | Lycaenidae | *Satyrium* | *esculi* | 97.92 |
| 1 | Insecta | Lepidoptera | Nymphalidae | *Melitaea* | *didyma* | 96.53 |
| 47 | Insecta | Lepidoptera | Lycaenidae | *Satyrium* | *ilicis* | 96.53 |

FinPROTAX output

|  | Class | Order | Family | Genus | Species | Probability |
| --- | --- | --- | --- | --- | --- | --- |
|  | **Insecta** |  |  |  |  | **0.999886** |
|  | **Insecta** | **Lepidoptera** |  |  |  | **0.936973** |
|  | **Insecta** | **Lepidoptera** | **Lycaenidae** |  |  | **0.838034** |
|  | **Insecta** | **Lepidoptera** | **Lycaenidae** | ***Satyrium*** |  | **0.151910** |
|  | **Insecta** | **Lepidoptera** | **Lycaenidae** | ***Satyrium*** | **unk** | **0.151816** |
|  | Insecta | Lepidoptera | Lycaenidae | *Scolitantides* |  | 0.302637 |
|  | Insecta | Lepidoptera | Lycaenidae | *Scolitantides* | unk | 0.302605 |

>19d

AATACTAGGAACATCTTTAAGAATTCTAATTCGAATAGAATTAGGAACACCAGGATCTTTAATTGGATATGATCAAATTTATAATACCATCGTAACAGCTCATGCTTTTATCATAATTTTTTTTATAGTAATACCTATTATAATT

BOLD output

| # | Class | Order | Family | Genus | Species | Match % |
| --- | --- | --- | --- | --- | --- | --- |
| 38 | **Insecta** | **Lepidoptera** | **Lycaenidae** | ***Satyrium*** | ***esculi*** | **99.31** |
| 1 | **Insecta** | **Lepidoptera** | **Lycaenidae** | ***Satyrium*** | ***spini*** | **99.31** |
| 7 | Insecta | Lepidoptera | Lycaenidae | *Satyrium* | *esculi* | 98.61 |
| 6 | Insecta | Lepidoptera | Lycaenidae | *Satyrium* | *esculi* | 97.92 |
| 1 | Insecta | Lepidoptera | Nymphalidae | *Melitaea* | *didyma* | 96.53 |
| 47 | Insecta | Lepidoptera | Lycaenidae | *Satyrium* | *ilicis* | 96.53 |

FinPROTAX output

|  | Class | Order | Family | Genus | Species | Probability |
| --- | --- | --- | --- | --- | --- | --- |
|  | **Insecta** |  |  |  |  | **0.999831** |
|  | **Insecta** | **Lepidoptera** |  |  |  | **0.914721** |
|  | **Insecta** | **Lepidoptera** | **Lycaenidae** |  |  | **0.682196** |
|  | Insecta | Lepidoptera | Lycaenidae | *Scolitantides* |  | 0.289773 |
|  | Insecta | Lepidoptera | Lycaenidae | *Scolitantides* | unk | 0.289742 |

>19e

AATACTAGGAACATCCTTAAGAATTCTAATTCGAATAGAATTAGGAACACCAGGATCTTTAATTGGAGATGATCAAATTTATAATACCATCGTAACAGCTCATGCTTTTATCATAATTTTTTTTATAGTAATACCTATTATAATT

BOLD output

| # | Class | Order | Family | Genus | Species | Match % |
| --- | --- | --- | --- | --- | --- | --- |
| 38 | **Insecta** | **Lepidoptera** | **Lycaenidae** | ***Satyrium*** | ***esculi*** | **99.31** |
| 1 | **Insecta** | **Lepidoptera** | **Lycaenidae** | ***Satyrium*** | ***spini*** | **99.31** |
| 7 | Insecta | Lepidoptera | Lycaenidae | *Satyrium* | *esculi* | 98.61 |
| 6 | Insecta | Lepidoptera | Lycaenidae | *Satyrium* | *esculi* | 97.92 |
| 1 | Insecta | Lepidoptera | Nymphalidae | *Melitaea* | *didyma* | 96.53 |
| 47 | Insecta | Lepidoptera | Lycaenidae | *Satyrium* | *ilicis* | 96.53 |

FinPROTAX output

|  | Class | Order | Family | Genus | Species | Probability |
| --- | --- | --- | --- | --- | --- | --- |
|  | **Insecta** |  |  |  |  | **0.999756** |
|  | **Insecta** | **Lepidoptera** |  |  |  | **0.905403** |
|  | **Insecta** | **Lepidoptera** | **Lycaenidae** |  |  | **0.653620** |
|  | Insecta | Lepidoptera | Lycaenidae | *Scolitantides* |  | 0.263903 |
|  | Insecta | Lepidoptera | Lycaenidae | *Scolitantides* | unk | 0.263875 |

20. *Xestia agathina*, Duponchel 1827

>20

GATAGTAGGAACTTCTTTAAGATTATTAATTCGAGCTGAATTAGGAAATCCTGGATCTTTAATTGGAGATGATCAAATTTATAATACTATTGTTACAGCACATGCTTTCATTATAATTTTTTTTATGGTAATACCTATTATAATT

BOLD output

| # | Class | Order | Family | Genus | Species | Match % |
| --- | --- | --- | --- | --- | --- | --- |
| 9 | **Insecta** | **Lepidoptera** | **Noctuidae** | ***Xestia*** | ***agathina*** | **100** |
| 3 | Insecta | Lepidoptera | Noctuidae | *Xestia* | *agathina* | 99.31 |
| 1 | Insecta | Lepidoptera | Noctuidae | *Luperina* | *testacea* | 98.61 |
| 1 | Insecta | Lepidoptera | Geometridae | *Chaetolopha* | *emporias* | 98.61 |
| 35 | Insecta | Lepidoptera | Noctuidae | *Xestia* | *ashworthii* | 98.61 |
| 1 | Insecta | Lepidoptera | Noctuidae | *Athetis* | *carayoniRF337-2016* | 98.61 |
| 1 | Insecta | Lepidoptera | Tortricidae | *Adoxophyes* | *sp.* | 98.61 |
| 1 | Insecta | Lepidoptera | Noctuidae | *Chersotis* | *cyrnea* | 98.61 |
| 1 | Insecta | Lepidoptera | Noctuidae | *Xestia* | *c-nigrum* | 98.6 |
| 1 | Insecta | Lepidoptera | Noctuidae | *Noctua* | *tertia* | 97.92 |
| 43 | Insecta | Lepidoptera | Noctuidae | *Luperina* | *testacea* | 97.92 |
| 1 | Insecta | Lepidoptera | Noctuidae | *Resapamea* | *testacea* | 97.92 |
| 1 | Insecta | Lepidoptera | Euteliidae | *Eutelia* | *abscondens* | 97.92 |
| 1 | Insecta | Lepidoptera | Geometridae | *Chaetolopha* | *emporias* | 97.92 |

FinPROTAX output

|  | Class | Order | Family | Genus | Species | Probability |
| --- | --- | --- | --- | --- | --- | --- |
|  | **Insecta** |  |  |  |  | **0.999985** |
|  | **Insecta** | **Lepidoptera** |  |  |  | **0.971673** |
|  | **Insecta** | **Lepidoptera** | **Noctuidae** |  |  | **0.899370** |
|  | **Insecta** | **Lepidoptera** | **Noctuidae** | ***Xestia*** |  | **0.308741** |
|  | Insecta | Lepidoptera | Noctuidae | *Xestia* | *ashworthii* | 0.233590 |

21. *Xylocampa areola*, Esper 1789

>21

AATAGTAGGAACTTCATTAAGATTGTTAATTCGAGCTGAATTAGGAAACCCTGGATCTTTAATTGGAGATGATCAAATTTATAATACTATTGTCACAGCTCATGCTTTTATTATAATTTTTTTTATAGTAATACCAATTATAATT

BOLD output

| # | Class | Order | Family | Genus | Species | Match % |
| --- | --- | --- | --- | --- | --- | --- |
| 9 | **Insecta** | **Lepidoptera** | **Noctuidae** | ***Xylocampa*** | ***areola*** | **100** |
| 1 | Insecta | Lepidoptera | Noctuidae | *Xylocampa* | *areola* | 99.31 |
| 1 | Insecta | Lepidoptera | Noctuidae | *Xylocampa* | *mustapha* | 99.31 |
| 1 | Insecta | Lepidoptera | Nymphalidae | *Brevioleria* | *aelia* | 98.92 |
| 1 | Insecta | Lepidoptera | Nymphalidae | *Brevioleria* | *arzalia* | 98.92 |
| 1 | Insecta | Lepidoptera | Nymphalidae | *Brevioleria* | *arzalia* | 98.89 |
| 1 | Insecta | Lepidoptera | Nymphalidae | *Brevioleria* | *arzalia* | 98.85 |
| 1 | Insecta | Lepidoptera | Erebidae | *Simplicia* | *moorei* | 98.25 |
| 1 | Insecta | Lepidoptera | Erebidae | *Simplicia* | *moorei* | 98.15 |
| 5 | Insecta | Lepidoptera | Noctuidae | *Xylocampa* | *areola* | 97.92 |
| 21 | Insecta | Lepidoptera | Erebidae | *Eublemma* | *purpurina* | 97.92 |
| 38 | Insecta | Lepidoptera | Noctuidae | *Helicoverpa* | *armigera* | 97.92 |
| 1 | Insecta | Lepidoptera | Erebidae | *Lophocampa* | *sp. 2-ML6* | 97.92 |
| 1 | Insecta | Lepidoptera | Geometridae | *Chiasmia* | *parallacta* | 97.92 |
| 1 | Insecta | Lepidoptera | Erebidae | *Lophocampa* | *sp. 17* | 97.92 |
| 16 | Insecta | Lepidoptera | Erebidae | *Lophocampa* | *pectina* | 97.92 |

FinPROTAX output

|  | Class | Order | Family | Genus | Species | Probability |
| --- | --- | --- | --- | --- | --- | --- |
|  | **Insecta** |  |  |  |  | **0.999977** |
|  | **Insecta** | **Lepidoptera** |  |  |  | **0.949856** |
|  | **Insecta** | **Lepidoptera** | **Noctuidae** |  |  | **0.786240** |
|  | Insecta | Lepidoptera | Noctuidae | *Xestia* |  | 0.108696 |
|  | Insecta | Lepidoptera | Noctuidae | *Xestia* | unk | 0.101170 |

22. *Aethes seriatana*, Zeller 1875

The match to *Aethes seriatana* has a single similarly good match to *Aethes baloghi* (Sabourin & Metzler 2002), but overlap to the latter could not be checked, while *A. seriatana* had multiple public matching records. For convenience, the identity is set to *A. seriatana*, but it has limited influence on the presented results since both have similar distributions in North America.

>22

AATAGTTGGAACATCATTAAGTTTATTAATTCGAGCAGAACTAGGTAATCCAGGATCACTAATTGGAGATGATCAAATTTATAATACTATTGTCACAGCTCATGCTTTTATTATAATTTTTTTTATAGTTATACCTATTATAATT

BOLD output

| # | Class | Order | Family | Genus | Species | Match % |
| --- | --- | --- | --- | --- | --- | --- |
| 6 | **Insecta** | **Lepidoptera** | **Tortricidae** | ***Aethes*** | ***seriatana*** | **100** |
| 1 | **Insecta** | **Lepidoptera** | **Tortricidae** | ***Aethes*** | ***baloghi*** | **100** |
| 1 | Insecta | Lepidoptera | Tortricidae | *Aethes* | *baloghi* | 99.31 |
| 1 | Insecta | Lepidoptera | Tortricidae | *Aethes* | *seriatana* | 99.31 |
| 1 | Insecta | Lepidoptera | Nymphalidae | *Heliconius* | *ethilla* | 98.77 |
| 3 | Insecta | Lepidoptera | Nymphalidae | *Heliconius* | *melpomene* | 98.77 |
| 1 | Insecta | Lepidoptera | Nymphalidae | *Heliconius* | *ethilla x Heliconius melpomene* | 98.75 |
| 1 | Insecta | Lepidoptera | Nymphalidae | *Melinaea* | *menophilus* | 98.75 |
| 1 | Insecta | Lepidoptera | Nymphalidae | *Heliconius* | *ethilla* | 98.73 |
| 1 | Insecta | Lepidoptera | Tortricidae | *Aethes* | *baloghi* | 98.61 |
| 1 | Insecta | Lepidoptera | Noctuidae | *Spodoptera* | *littoralis* | 97.85 |
| 15 | Insecta | Lepidoptera | Nymphalidae | *Heliconius* | *melpomene* | 97.85 |
| 8 | Insecta | Lepidoptera | Nymphalidae | *Heliconius* | *numata* | 97.85 |
| 11 | Insecta | Lepidoptera | Nymphalidae | *Heliconius* | *timareta* | 97.85 |
| 1 | Insecta | Lepidoptera | Nymphalidae | *Heliconius* | *pardalinus* | 97.8 |
| 1 | Insecta | Lepidoptera | Nymphalidae | *Heliconius* | *pardalinus* | 97.78 |
| 1 | Insecta | Lepidoptera | Nymphalidae | *Heliconius* | *numata* | 97.7 |
| 1 | Insecta | Lepidoptera | Noctuidae | *Spodoptera* | *frugiperda* | 97.7 |
| 1 | Insecta | Lepidoptera | Nymphalidae | *Oeneis* | *sculda* | 97.7 |
| 2 | Insecta | Lepidoptera | Nymphalidae | *Cymothoe* | *egesta* | 97.62 |
| 1 | Insecta | Lepidoptera | Nymphalidae | *Melinaea* | *marsaeus* | 97.62 |
| 1 | Insecta | Lepidoptera | Nymphalidae | *Heliconius* | *elevatus* | 97.53 |
| 1 | Insecta | Lepidoptera | Nymphalidae | *Heliconius* | *ethilla* | 97.53 |
| 1 | Insecta | Lepidoptera | Nymphalidae | *Mechanitis* | *mazaeus* | 97.53 |
| 1 | Insecta | Lepidoptera | Nymphalidae | *Heliconius* | *hecale* | 97.53 |
| 1 | Insecta | Lepidoptera | Noctuidae | *Pseudobryomima* | *sp. KLKDNA0340* | 97.53 |
| 1 | Insecta | Lepidoptera | Nymphalidae | *Paralasa* | *styx* | 97.44 |
| 1 | Insecta | Lepidoptera | Hesperiidae | *Halotus* | *jonaveriorum* | 97.14 |
| 6 | Insecta | Lepidoptera | Noctuidae | *Spodoptera* | *frugiperda sp. 2* | 96.88 |
| 1 | Insecta | Lepidoptera | Noctuidae | *Spodoptera* | *frugiperda* | 96.88 |
| 1 | Insecta | Lepidoptera | Pieridae | *Colias* | *erate* | 96.77 |
| 4 | Insecta | Lepidoptera | Noctuidae | *Spodoptera* | *dolichos* | 96.77 |
| 1 | Insecta | Lepidoptera | Nymphalidae | *Brevioleria* | *aelia* | 96.77 |
| 1 | Insecta | Lepidoptera | Nymphalidae | *Brevioleria* | *arzalia* | 96.77 |
| 2 | Insecta | Lepidoptera | Nymphalidae | *Melinaea* | *satevis* | 96.77 |
| 1 | Insecta | Lepidoptera | Nymphalidae | *Napeogenes* | *sylphis* | 96.77 |
| 2 | Insecta | Lepidoptera | Noctuidae | *Spodoptera* | *pulchella* | 96.77 |
| 14 | Insecta | Lepidoptera | Noctuidae | *Spodoptera* | *litura* | 96.77 |

FinPROTAX output

|  | Class | Order | Family | Genus | Species | Probability |
| --- | --- | --- | --- | --- | --- | --- |
|  | **Insecta** |  |  |  |  | **0.999956** |
|  | **Insecta** | **Lepidoptera** |  |  |  | **0.870781** |
|  | Insecta | Lepidoptera | Gelechiidae |  |  | 0.122653 |
|  | Insecta | Lepidoptera | Noctuidae |  |  | 0.309410 |

23. *Henricus cognatus* (*Henricus cognata*), Walsingham 1914

>23a

AATAGTTGGAACTTCTTTAAGATTATTAATTCGAGCTGAATTAGGAAGTCCAGGTTCATTAATTGGTGACGATCAAATTTACAATACAATTGTCACAGCTCATGCATTTATTATAATTTTTTTCATAGTTATACCCATTATAATT

BOLD output

| # | Class | Order | Family | Genus | Species | Match % |
| --- | --- | --- | --- | --- | --- | --- |
| 3 | **Insecta** | **Lepidoptera** | **Tortricidae** | ***Henricus*** | ***cognatus*** | **100** |
| 1 | Insecta | Lepidoptera | Tortricidae | *Henricus* | *cognatus* | 99.31 |
| 1 | Insecta | Lepidoptera | Tortricidae | *Henricus* | *cognatus* | 96.53 |
| 1 | Insecta | Lepidoptera | Tortricidae | *tortBioLep01* | *BioLep47* | 95.83 |
| 1 | Insecta | Lepidoptera | Tortricidae | *Henricus* | *umbrabasanus* | 95.83 |
| 2 | Insecta | Lepidoptera | Tortricidae | *Henricus* | *edwardsiana* | 95.83 |
| 1 | Insecta | Lepidoptera | Tortricidae | *tortBioLep01* | *BioLep47* | 95.14 |
| 4 | Insecta | Lepidoptera | Tortricidae | *Henricus* | *edwardsiana* | 95.14 |
| 7 | Insecta | Lepidoptera | Geometridae | *Lithostege* | *fissurata* | 94.44 |
| 1 | Insecta | Lepidoptera | Bucculatricidae | *Bucculatrix* | *sp.* | 94.44 |
| 1 | Insecta | Lepidoptera | Geometridae | *Laciniodes* | *plurilinearia* | 94.44 |
| 1 | Insecta | Lepidoptera | Geometridae | *Problepsis* | *flavistigma* | 94.44 |
| 5 | Insecta | Lepidoptera | Geometridae | *Somatina* | *centrophora* | 94.44 |
| 1 | Insecta | Lepidoptera | Bucculatricidae | *Bucculatrix* | *diffusella* | 94.44 |
| 22 | Insecta | Lepidoptera | Sphingidae | *Phyllosphingia* | *dissimilis* | 94.44 |
| 2 | Insecta | Lepidoptera | Erebidae | *Metalectra* | *sp. nr. edilis* | 94.44 |
| 1 | Insecta | Lepidoptera | Crambidae | *Carectocultus* | *dominicki* | 94.44 |
| 5 | Insecta | Lepidoptera | Tortricidae | *Henricus* | *umbrabasanus* | 94.44 |
| 5 | Insecta | Lepidoptera | Tortricidae | *Henricus* | *edwardsiana* | 94.44 |
| 10 | Insecta | Lepidoptera | Crambidae | *Chrysothyridia* | *invertalis* | 94.44 |
| 2 | Insecta | Lepidoptera | Gelechiidae | *Ardozyga* | *polioxysta* | 94.44 |
| 3 | Insecta | Lepidoptera | Crambidae | *Diaphania* | *BioLep345* | 94.44 |
| 1 | Insecta | Lepidoptera | Noctuidae | *Anadevidia* | *peponis* | 94.44 |
| 1 | Insecta | Lepidoptera | Crambidae | *Syllepis* | *marialis* | 94.44 |
| 1 | Insecta | Lepidoptera | Geometridae | *Problepsis* | *centrophora DS01* | 94.44 |
| 14 | Insecta | Lepidoptera | Erebidae | *Metalectra* | *dixoni* | 94.44 |
| 1 | Insecta | Lepidoptera | Tortricidae | *tortBioLep01* | *BioLep1462* | 94.41 |
| 1 | Insecta | Lepidoptera | Sphingidae | *Phyllosphingia* | *dissimilis* | 94.41 |
| 1 | Insecta | Lepidoptera | Erebidae | *Metalectra* | *dixoni* | 94.41 |

FinPROTAX output

|  | Class | Order | Family | Genus | Species | Probability |
| --- | --- | --- | --- | --- | --- | --- |
|  | **Insecta** |  |  |  |  | **0.999917** |
|  | Insecta | Diptera |  |  |  | 0.114813 |
|  | **Insecta** | **Lepidoptera** |  |  |  | **0.785670** |
|  | Insecta | Lepidoptera | Geometridae |  |  | 0.102800 |
|  | Insecta | Lepidoptera | Noctuidae |  |  | 0.128627 |
|  | **Insecta** | **Lepidoptera** | **Tortricidae** |  |  | **0.409584** |
|  | Insecta | Lepidoptera | Tortricidae | *Aethes* |  | 0.145034 |
|  | Insecta | Lepidoptera | Tortricidae | *Aethes* | unk | 0.145031 |

>23b

AATAGTTGGAACTTCTTTAAGATTATTAATTCGAGCTGAATTAGGAAGTCCAGGTTCGTTAATTGGTGACGATCAAATTTACAATACAATTGTCACAGCTCATGCATTTATTATAATTTTTTTCATAGTTATACCCATTATAATT

BOLD output

| # | Class | Order | Family | Genus | Species | Match % |
| --- | --- | --- | --- | --- | --- | --- |
| 3 | **Insecta** | **Lepidoptera** | **Tortricidae** | ***Henricus*** | ***cognatus*** | **99.31** |
| 1 | Insecta | Lepidoptera | Tortricidae | *Henricus* | *cognatus* | 98.61 |
| 1 | Insecta | Lepidoptera | Tortricidae | *Henricus* | *cognatus* | 95.83 |
| 1 | Insecta | Lepidoptera | Tortricidae | *tortBioLep01* | *BioLep47* | 95.14 |
| 1 | Insecta | Lepidoptera | Tortricidae | *Henricus* | *umbrabasanus* | 95.14 |
| 2 | Insecta | Lepidoptera | Tortricidae | *Henricus* | *edwardsiana* | 95.14 |
| 1 | Insecta | Lepidoptera | Bucculatricidae | *Bucculatrix* | *sp.* | 94.44 |
| 1 | Insecta | Lepidoptera | Bucculatricidae | *Bucculatrix* | *diffusella* | 94.44 |
| 1 | Insecta | Lepidoptera | Tortricidae | *tortBioLep01* | *BioLep47* | 94.44 |
| 2 | Insecta | Lepidoptera | Erebidae | *Metalectra* | *sp. nr. edilis* | 94.44 |
| 4 | Insecta | Lepidoptera | Tortricidae | *Henricus* | *edwardsiana* | 94.44 |
| 1 | Insecta | Lepidoptera | Noctuidae | *Anadevidia* | *peponis* | 94.44 |
| 14 | Insecta | Lepidoptera | Erebidae | *Metalectra* | *dixoni* | 94.44 |
| 1 | Insecta | Lepidoptera | Erebidae | *Metalectra* | *dixoni* | 94.41 |
| 7 | Insecta | Lepidoptera | Geometridae | *Lithostege* | *fissurata* | 93.75 |
| 1 | Insecta | Lepidoptera | Noctuidae | *Odontestra* | *sp.* | 93.75 |
| 4 | Insecta | Lepidoptera | Coleophoridae | *Coleophora* | *maritimella* | 93.75 |
| 1 | Insecta | Lepidoptera | Lasiocampidae | *Sena* | *prompta* | 93.75 |
| 1 | Insecta | Lepidoptera | Geometridae | *Hypobapta* | *barnardi* | 93.75 |
| 1 | Insecta | Lepidoptera | Coleophoridae | *Coleophora* | *acutiphaga* | 93.75 |
| 1 | Insecta | Lepidoptera | Noctuidae | *Euxoa* | *vernalis* | 93.75 |
| 1 | Insecta | Lepidoptera | Geometridae | *Laciniodes* | *plurilinearia* | 93.75 |
| 1 | Insecta | Lepidoptera | Geometridae | *Problepsis* | *flavistigma* | 93.75 |
| 5 | Insecta | Lepidoptera | Geometridae | *Somatina* | *centrophora* | 93.75 |
| 2 | Insecta | Lepidoptera | Erebidae | *Amata* | *cf. cyanura* | 93.75 |
| 4 | Insecta | Lepidoptera | Crambidae | *Omiodes* | *poeonalis* | 93.75 |
| 5 | Insecta | Lepidoptera | Depressariidae | *elachBioLep01* | *BioLep816* | 93.75 |
| 1 | Insecta | Lepidoptera | Sphingidae | *Phyllosphingia* | *dissimilis* | 93.75 |
| 1 | Insecta | Lepidoptera | Erebidae | *Metalectra* | *praecisalis* | 93.75 |
| 3 | Insecta | Lepidoptera | Coleophoridae | *Coleophora* | *tanitella* | 93.75 |
| 1 | Insecta | Lepidoptera | Erebidae | *Hyalurga* | *leucophlebia* | 93.75 |
| 6 | Insecta | Lepidoptera | Coleophoridae | *Coleophora* | *soffneriella* | 93.75 |
| 1 | Insecta | Lepidoptera | Crambidae | *Carectocultus* | *dominicki* | 93.75 |
| 1 | Insecta | Lepidoptera | Noctuidae | *Drobeta* | *sp. JB1092* | 93.75 |
| 18 | Insecta | Lepidoptera | Noctuidae | *Euxoa* | *auxiliaris* | 93.75 |

FinPROTAX output

|  | Class | Order | Family | Genus | Species | Probability |
| --- | --- | --- | --- | --- | --- | --- |
|  | **Insecta** |  |  |  |  | **0.999877** |
|  | Insecta | Diptera |  |  |  | 0.114431 |
|  | **Insecta** | **Lepidoptera** |  |  |  | **0.783055** |
|  | Insecta | Lepidoptera | Geometridae |  |  | 0.198132 |
|  | Insecta | Lepidoptera | Noctuidae |  |  | 0.247910 |
|  | **Insecta** | **Lepidoptera** | **Tortricidae** |  |  | **0.234682** |

24. *Henricus umbrabasanus*, Kearfott 1908

>24

AATAGTTGGAACTTCATTAAGATTATTAATTCGAGCTGAATTAGGAAGTCCTGGCTCATTAATTGGTGATGATCAAATTTATAATACAATTGTTACAGCTCACGCATTTATTATAATTTTTTTTATAGTTATACCCATTATAATT

BOLD output

| # | Class | Order | Family | Genus | Species | Match % |
| --- | --- | --- | --- | --- | --- | --- |
| 4 | **Insecta** | **Lepidoptera** | **Tortricidae** | ***Henricus*** | ***umbrabasanus*** | **100** |
| 12 | Insecta | Lepidoptera | Tortricidae | *Henricus* | *umbrabasanus* | 99.31 |
| 7 | Insecta | Lepidoptera | Tortricidae | *Henricus* | *umbrabasanus* | 98.61 |
| 1 | Insecta | Lepidoptera | Tortricidae | *Henricus* | *umbrabasanus* | 97.92 |
| 1 | Insecta | Lepidoptera | Tortricidae | *tortBioLep01* | *BioLep1462* | 96.5 |
| 1 | Insecta | Lepidoptera | Coleophoridae | *Coleophora* | *JFL002* | 96.24 |
| 2 | Insecta | Lepidoptera | Tortricidae | *Henricus* | *edwardsiana* | 95.83 |
| 27 | Insecta | Lepidoptera | Geometridae | *geoMalaise01* | *Malaise5260* | 95.83 |
| 2 | Insecta | Lepidoptera | Crambidae | *Sylepta* | *solilucis* | 95.83 |
| 23 | Insecta | Lepidoptera | Crambidae | *Hyalobathra* | *brevialis* | 95.83 |
| 2 | Insecta | Lepidoptera | Tortricidae | *Henricus* | *BioLep52* | 95.83 |
| 1 | Insecta | Lepidoptera | Nepticulidae | *Zimmermannia* | *longicaudella* | 95.77 |
| 1 | Insecta | Lepidoptera | Nepticulidae | *Stigmella* | *arbusculae* | 95.42 |
| 1 | Insecta | Lepidoptera | Papilionidae | *Pachliopta* | *aristolochiae* | 95.4 |
| 1 | Insecta | Lepidoptera | Nymphalidae | *Brevioleria* | *aelia* | 95.29 |
| 3 | Insecta | Lepidoptera | Geometridae | *Rhodostrophia* | *tumulosa* | 95.14 |
| 2 | Insecta | Lepidoptera | Erebidae | *Saurita* | *afflicta* | 95.14 |
| 9 | Insecta | Lepidoptera | Crambidae | *Pilemia* | *periusalis* | 95.14 |

FinPROTAX output

|  | Class | Order | Family | Genus | Species | Probability |
| --- | --- | --- | --- | --- | --- | --- |
|  | **Insecta** |  |  |  |  | **0.999962** |
|  | Insecta | Coleoptera |  |  |  | 0.221035 |
|  | Insecta | Coleoptera | Staphylinidae |  |  | 0.217889 |
|  | Insecta | Coleoptera | Staphylinidae | *Atheta* |  | 0.192618 |
|  | Insecta | Coleoptera | Staphylinidae | *Atheta* | unk | 0.192618 |
|  | **Insecta** | **Lepidoptera** |  |  |  | **0.732691** |
|  | Insecta | Lepidoptera | Coleophoridae |  |  | 0.162075 |
|  | Insecta | Lepidoptera | Coleophoridae | *Coleophora* |  | 0.162068 |
|  | Insecta | Lepidoptera | Coleophoridae | *Coleophora* | unk | 0.161985 |
|  | Insecta | Lepidoptera | Geometridae |  |  | 0.195112 |
|  | Insecta | Lepidoptera | Noctuidae |  |  | 0.244131 |

25. *Platphalonidia felix*, Walsingham 1895

While a good match at 100% for *Spodoptera frugiperda* (Smith & Abbot 1797) appears to exist for the ASV identified as *Platphalonidia felix*, the former is based on only 87 (out of 145) bp overlap between queried and reference sequence, and as such it was not taken into account.

>25

AATAGTAGGAACTTCTTTAAGATTATTAATTCGAGCTGAATTAGGTAATCCTGGTTCTTTAATTGGAGATGATCAAATTTATAATACTATTGTAACAGCTCATGCTTTTATTATAATTTTTTTTATAGTTATACCTATTATAATT

BOLD output

| # | Class | Order | Family | Genus | Species | Match % |
| --- | --- | --- | --- | --- | --- | --- |
| 4 | **Insecta** | **Lepidoptera** | **Tortricidae** | ***Platphalonidia*** | ***felix*** | **100** |
| 1 | Insecta | Lepidoptera | Noctuidae | *Spodoptera* | *frugiperda* | 100 |
| 1 | Insecta | Lepidoptera | Geometridae | *Scopula* | *risa* | 99.31 |
| 1 | Insecta | Lepidoptera | Erebidae | *Oraesia* | *wintgensi* | 99.31 |
| 1 | Insecta | Lepidoptera | Geometridae | *Scopula* | *iterata* | 99.31 |
| 1 | Insecta | Lepidoptera | Erebidae | *Arctia* | *thibetica* | 99.31 |
| 1 | Insecta | Lepidoptera | Noctuidae | *Ecpatia* | *spiculivalva* | 99.31 |
| 23 | Insecta | Lepidoptera | Erebidae | *Tetanolita* | *floridana* | 99.31 |
| 67 | Insecta | Lepidoptera | Depressariidae | *Ethmia* | *hammella* | 99.31 |

FinPROTAX output

|  | Class | Order | Family | Genus | Species | Probability |
| --- | --- | --- | --- | --- | --- | --- |
|  | **Insecta** |  |  |  |  | **0.999987** |
|  | **Insecta** | **Lepidoptera** |  |  |  | **0.948688** |
|  | Insecta | Lepidoptera | Erebidae |  |  | 0.100919 |
|  | Insecta | Lepidoptera | Noctuidae |  |  | 0.372512 |
|  | **Insecta** | **Lepidoptera** | **Tortricidae** |  |  | **0.308638** |
