# Supplementary material for: Exploring and validating observations of non‐local species in eDNA samples
Source: Ecol Evol. 2023 Oct 14;13(10):e10612. doi: 10.1002/ece3.10612 (PMC10576249; doi:10.1002/ece3.10612)
Supplement: Supplementary file 3 — Appendix S3 [file ECE3-13-e10612-s005.docx]

**Exploring and validating observations of non-local species in eDNA samples**

Coen Westerduin, Marko Suokas, Tuukka Petäjä, Ulla Saarela, Seppo Vainio, Marko Mutanen

**Supporting Information 3.** Maps indicating approximate distributions based on observational data for all 25 non-local species found in the original 2017 dataset. Occurrences are indicated by yellow-orange markers (darker colours indicate higher numbers), while the location of the field area near Oulu is indicated by a light blue star. GBIF data is licenced under CC BY-NC 4.0, base map data is © OpenStreetMap contributors and © OpenMapTiles.

1 *Agriopis marginaria*


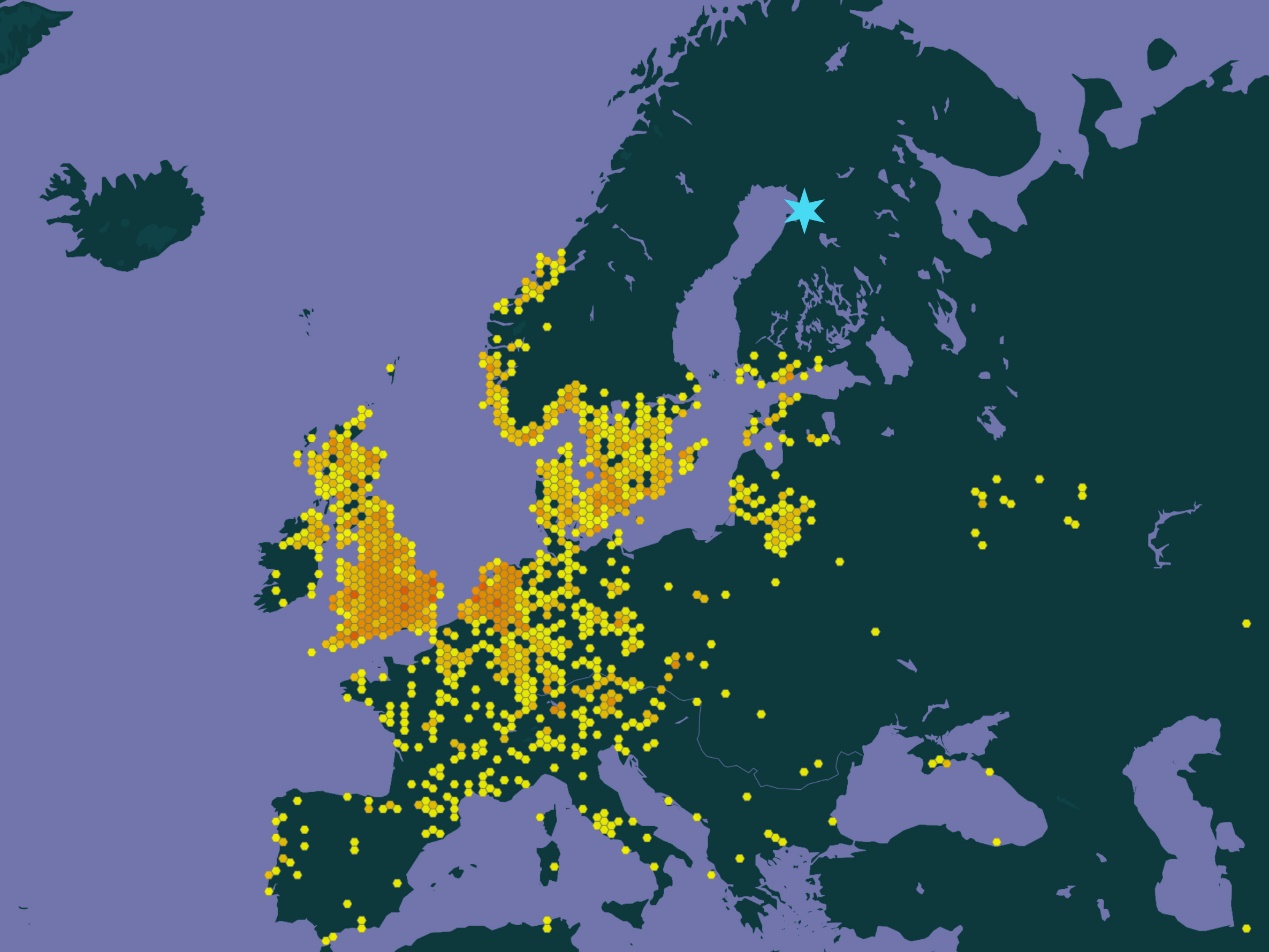


Source: GBIF.org (18 May 2022) GBIF Occurrence Download https://doi.org/10.15468/dl.89959f

2 *Bena bicolorana*


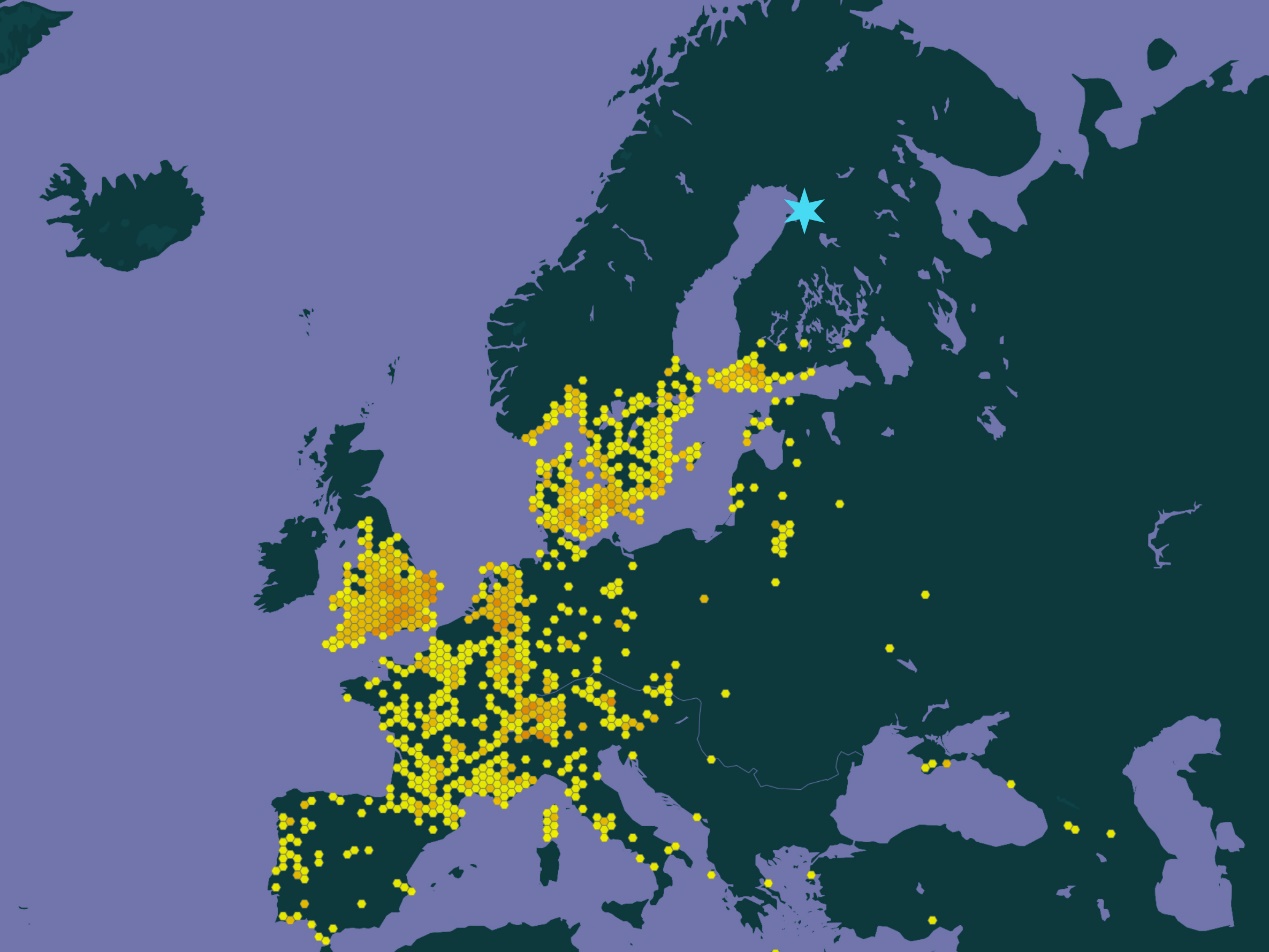


Source: GBIF.org (18 May 2022) GBIF Occurrence Download https://doi.org/10.15468/dl.rc8bna

3 *Biston stratarius* (as *Biston strataria*)


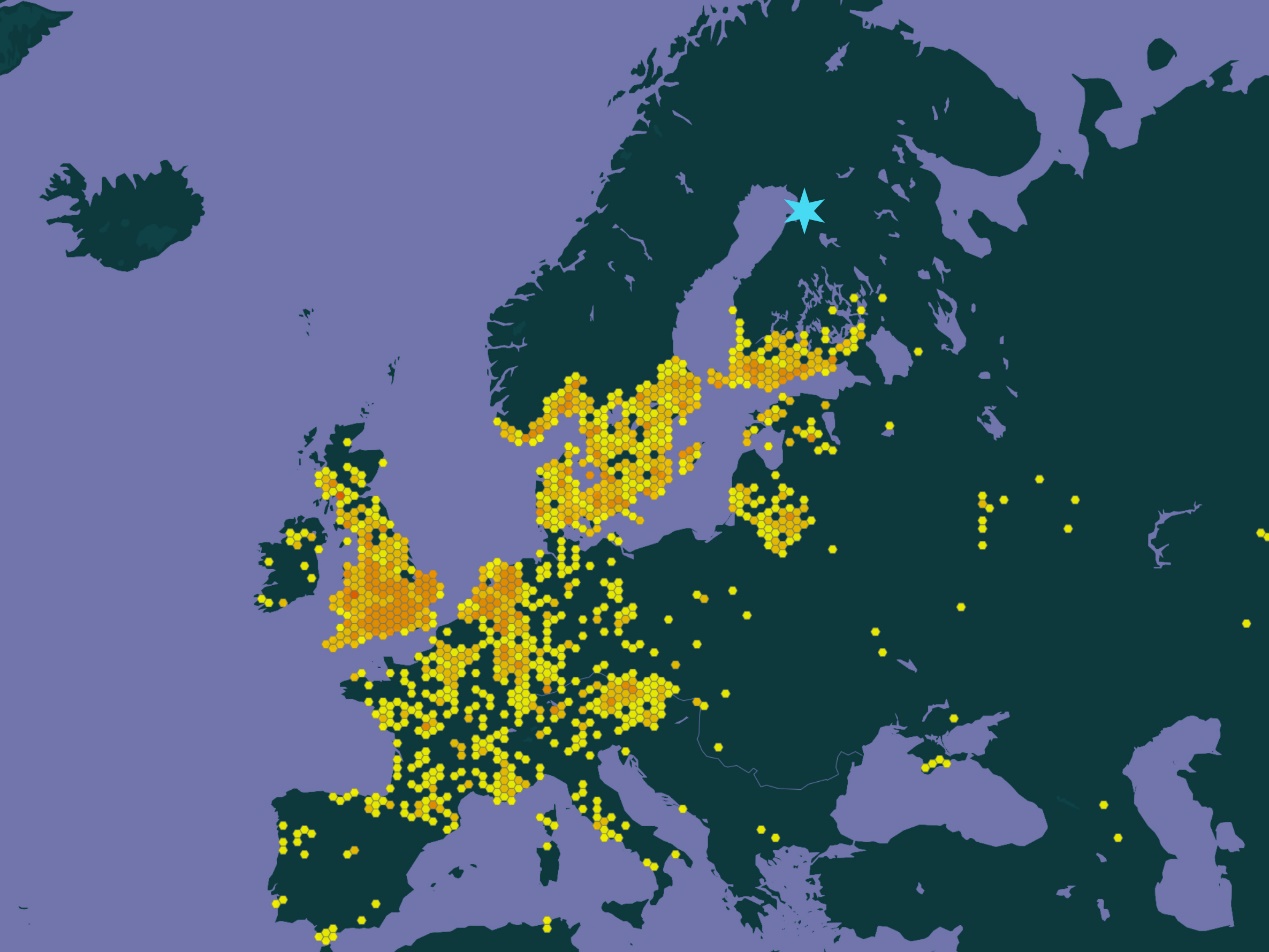


Source: GBIF.org (18 May 2022) GBIF Occurrence Download https://doi.org/10.15468/dl.53efzd

4 *Campaea honoraria*


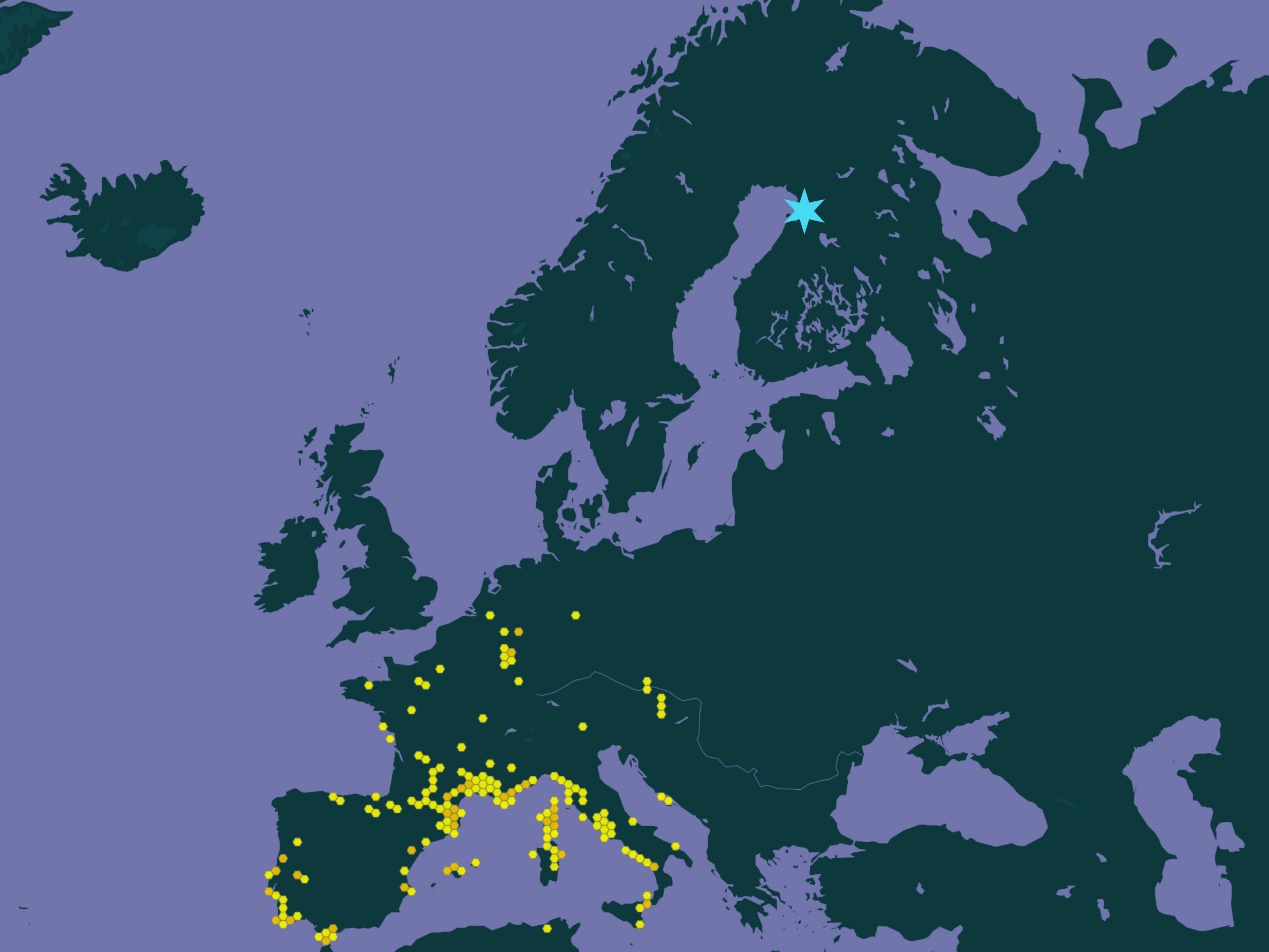


Source: GBIF.org (18 May 2022) GBIF Occurrence Download https://doi.org/10.15468/dl.5uy5xe

5 *Catephia alchymista*


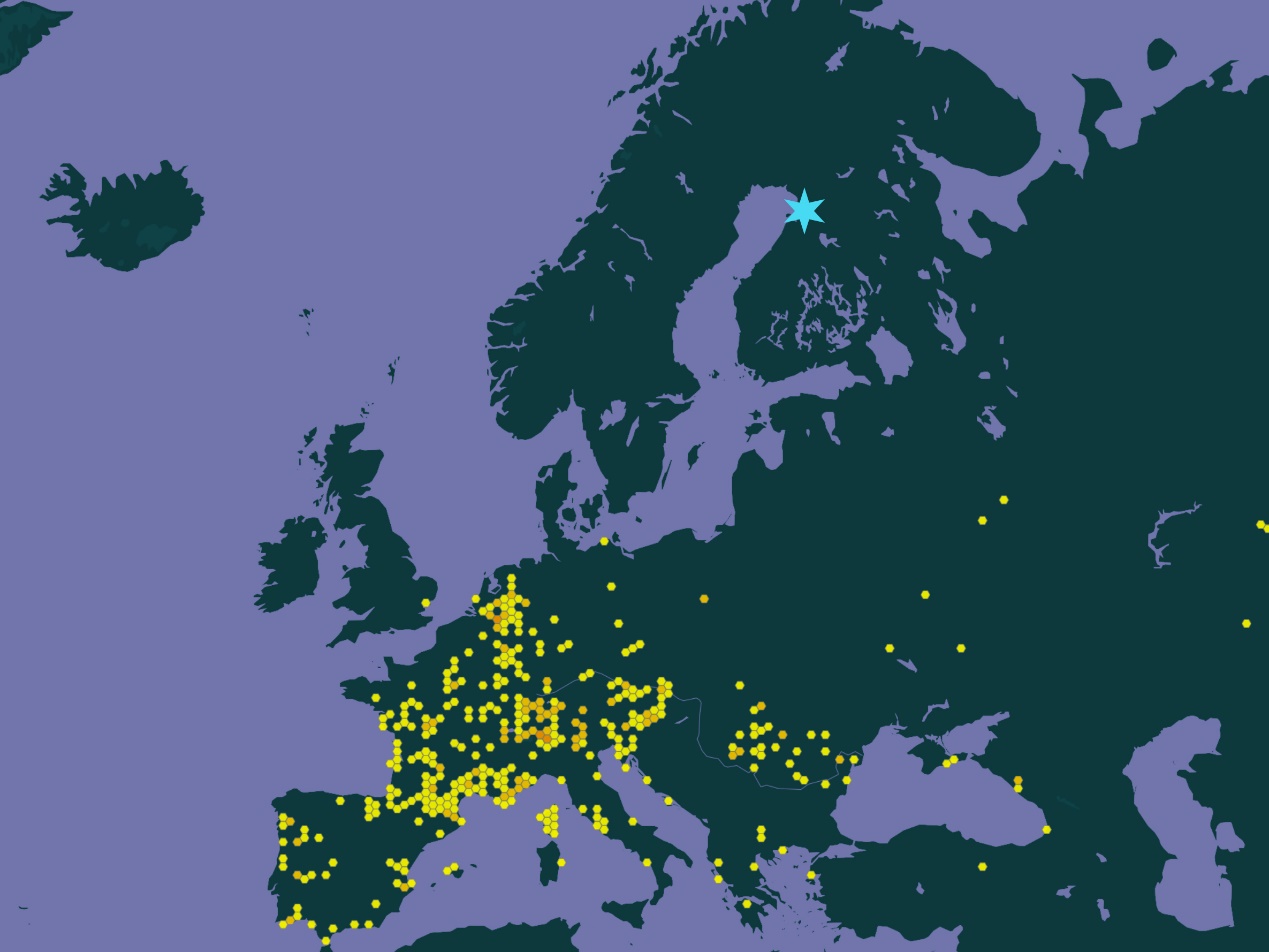


Source: GBIF.org (18 May 2022) GBIF Occurrence Download https://doi.org/10.15468/dl.9m8cfw

6 *Catocala conjuncta* (as *Catocala coniuncta*)


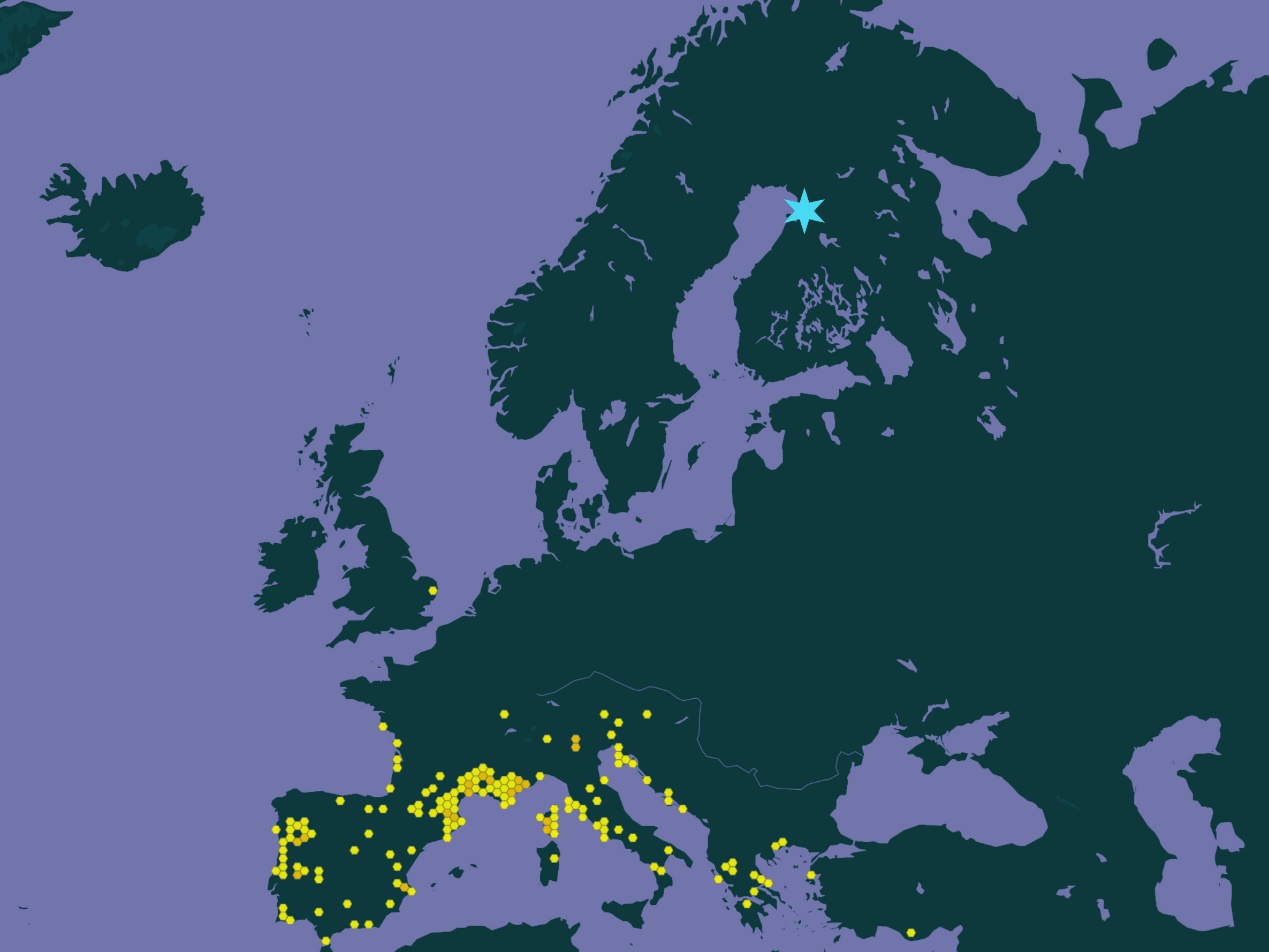


Source: GBIF.org (18 May 2022) GBIF Occurrence Download https://doi.org/10.15468/dl.vd3qgf

7 *Catocala nymphagoga*


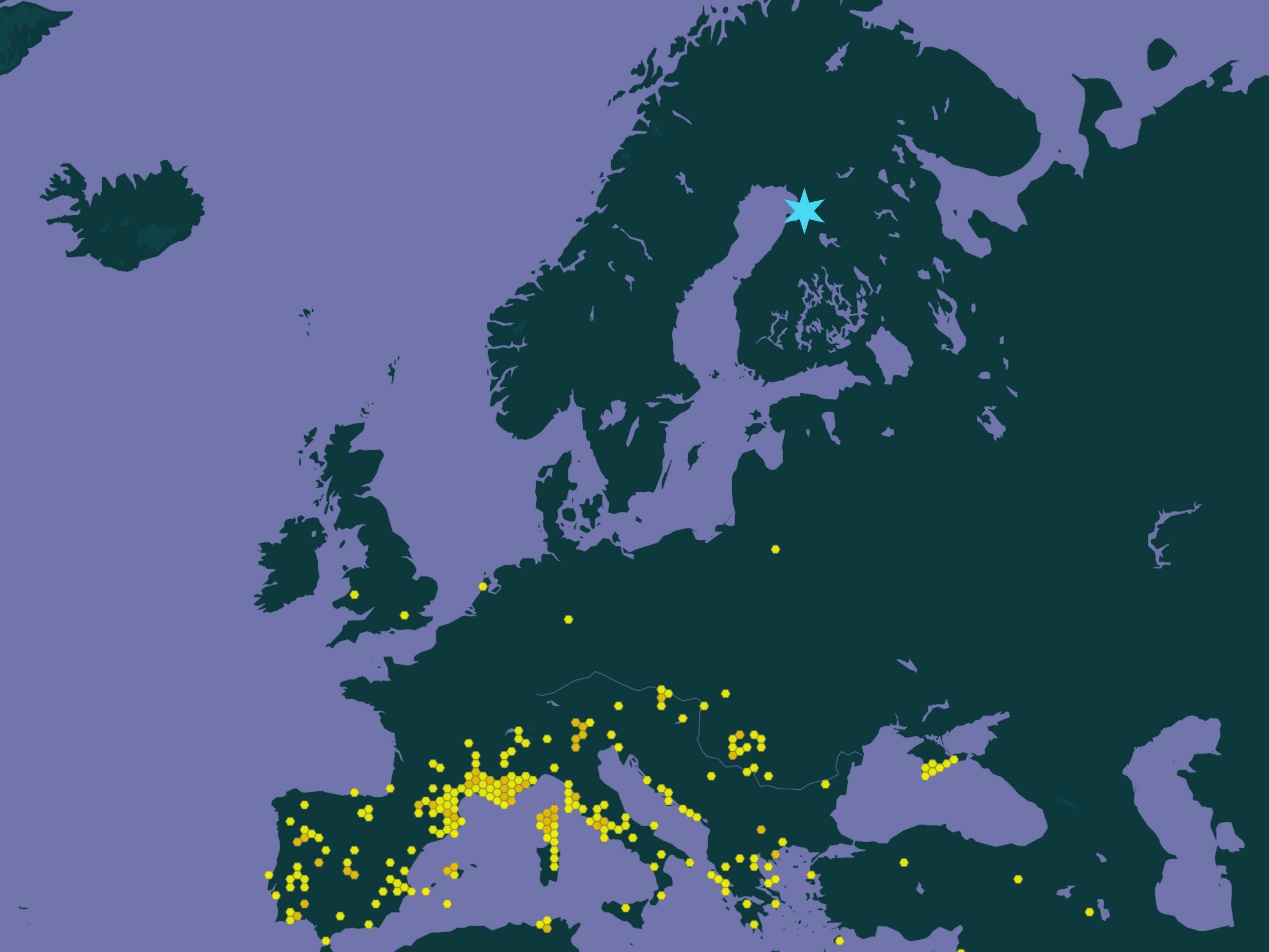


Source: GBIF.org (18 May 2022) GBIF Occurrence Download https://doi.org/10.15468/dl.bftpeb

8 *Dryobotodes tenebrosa*


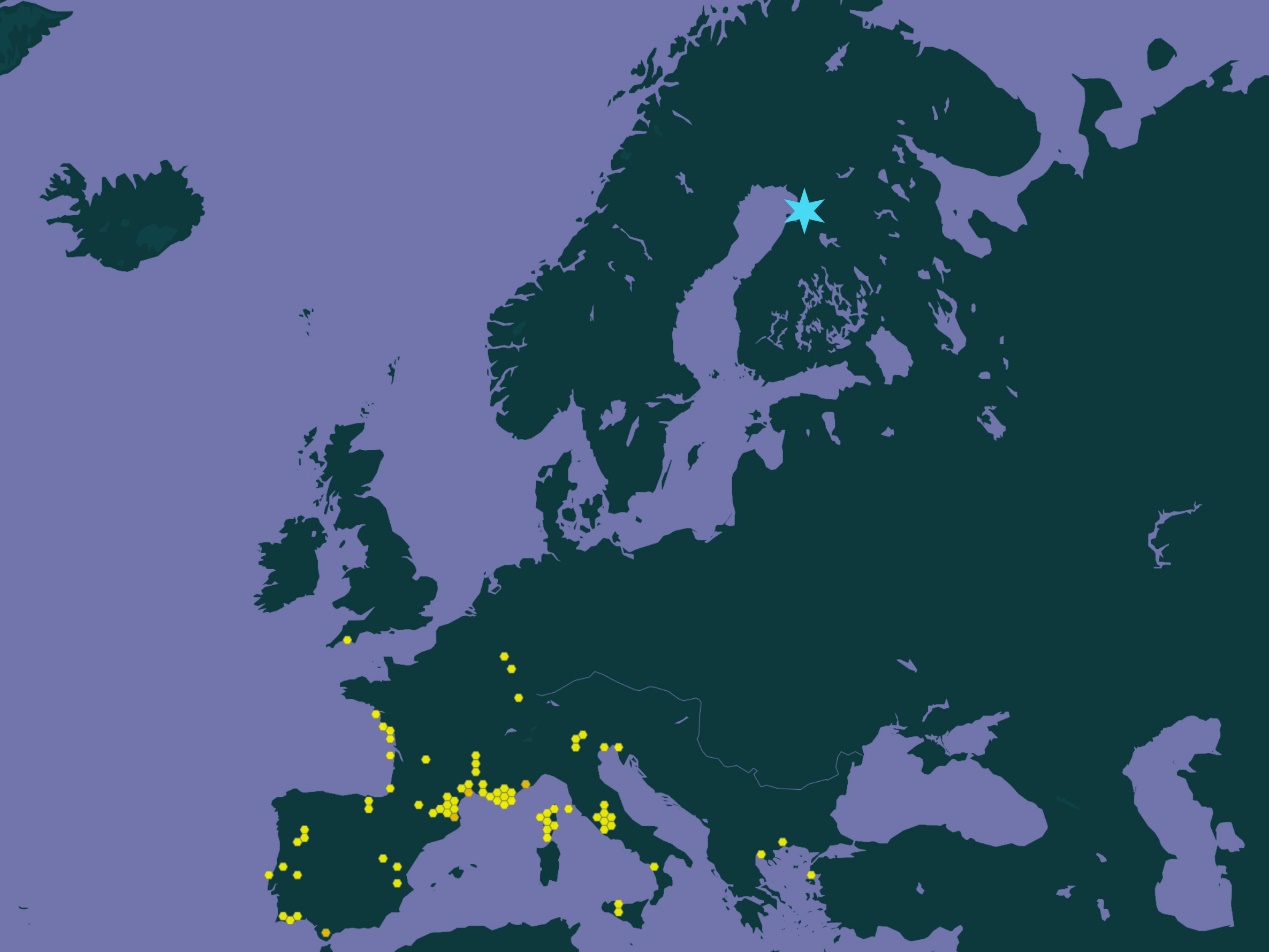


Source: GBIF.org (18 May 2022) GBIF Occurrence Download https://doi.org/10.15468/dl.u96ayg

9 *Gonepteryx cleopatra*


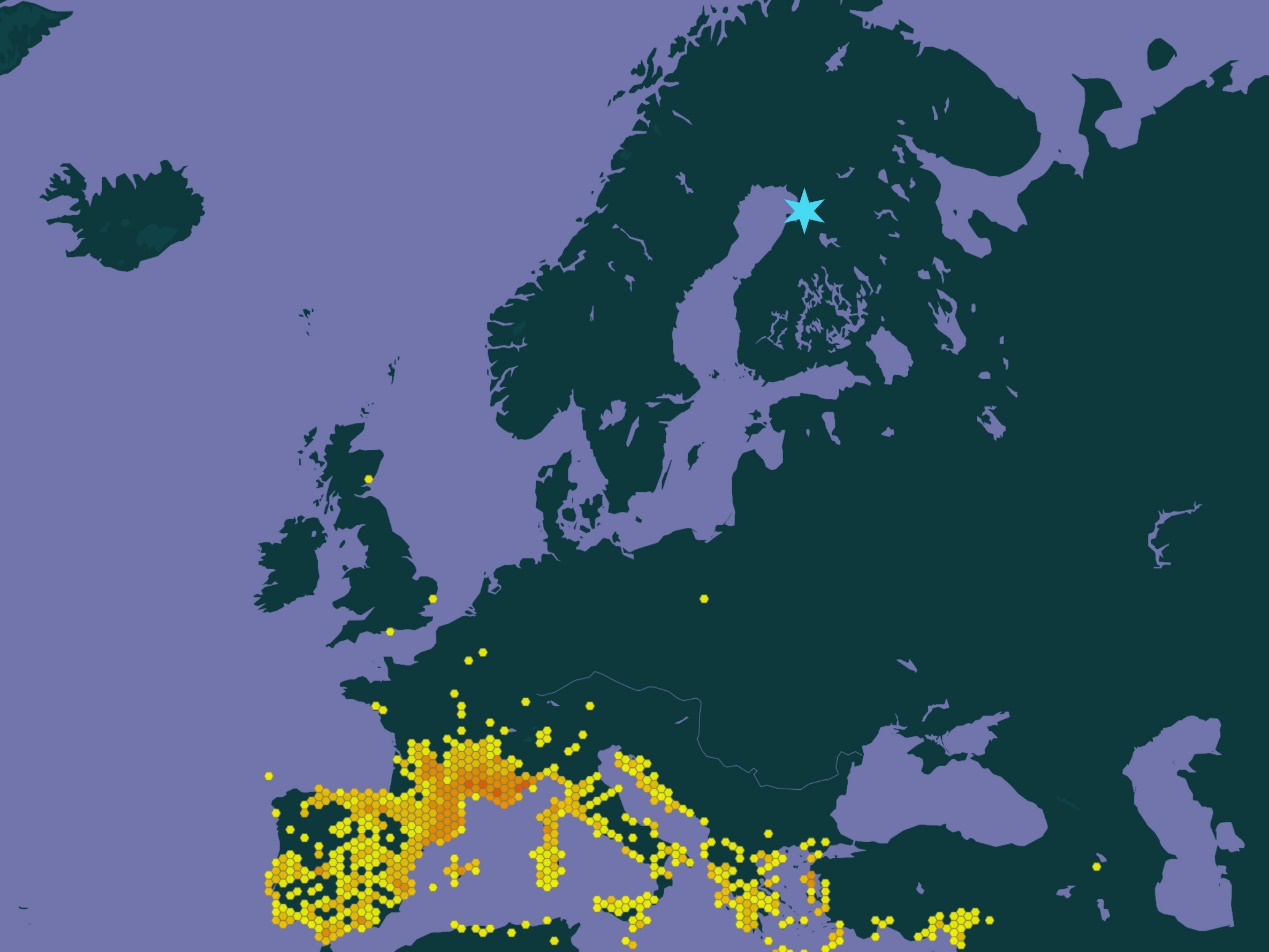


Source: GBIF.org (18 May 2022) GBIF Occurrence Download https://doi.org/10.15468/dl.uaycxp

10 *Lithosia quadra*


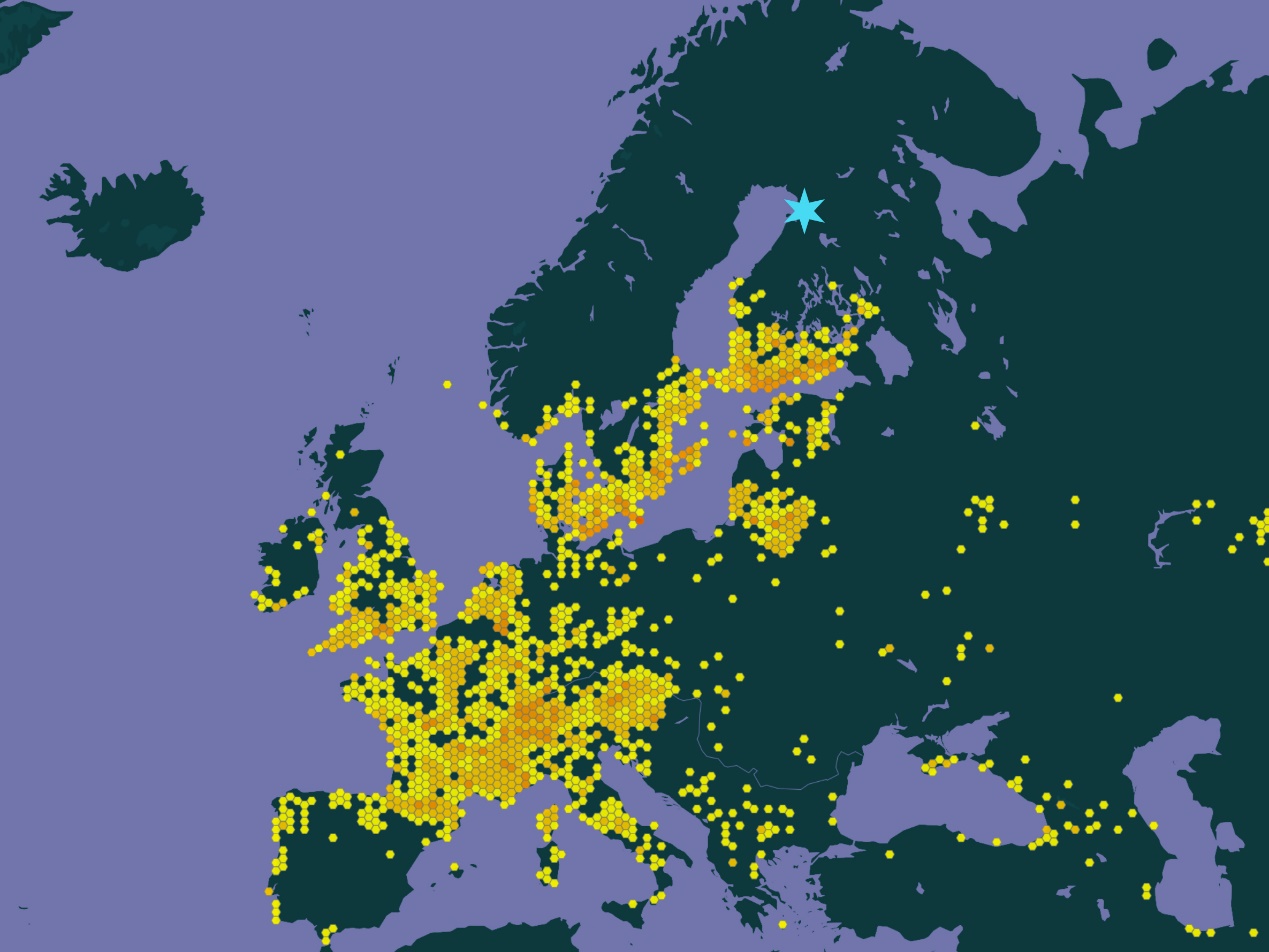


Source: GBIF.org (18 May 2022) GBIF Occurrence Download https://doi.org/10.15468/dl.qfxdzb

11 *Lymantria dispar*


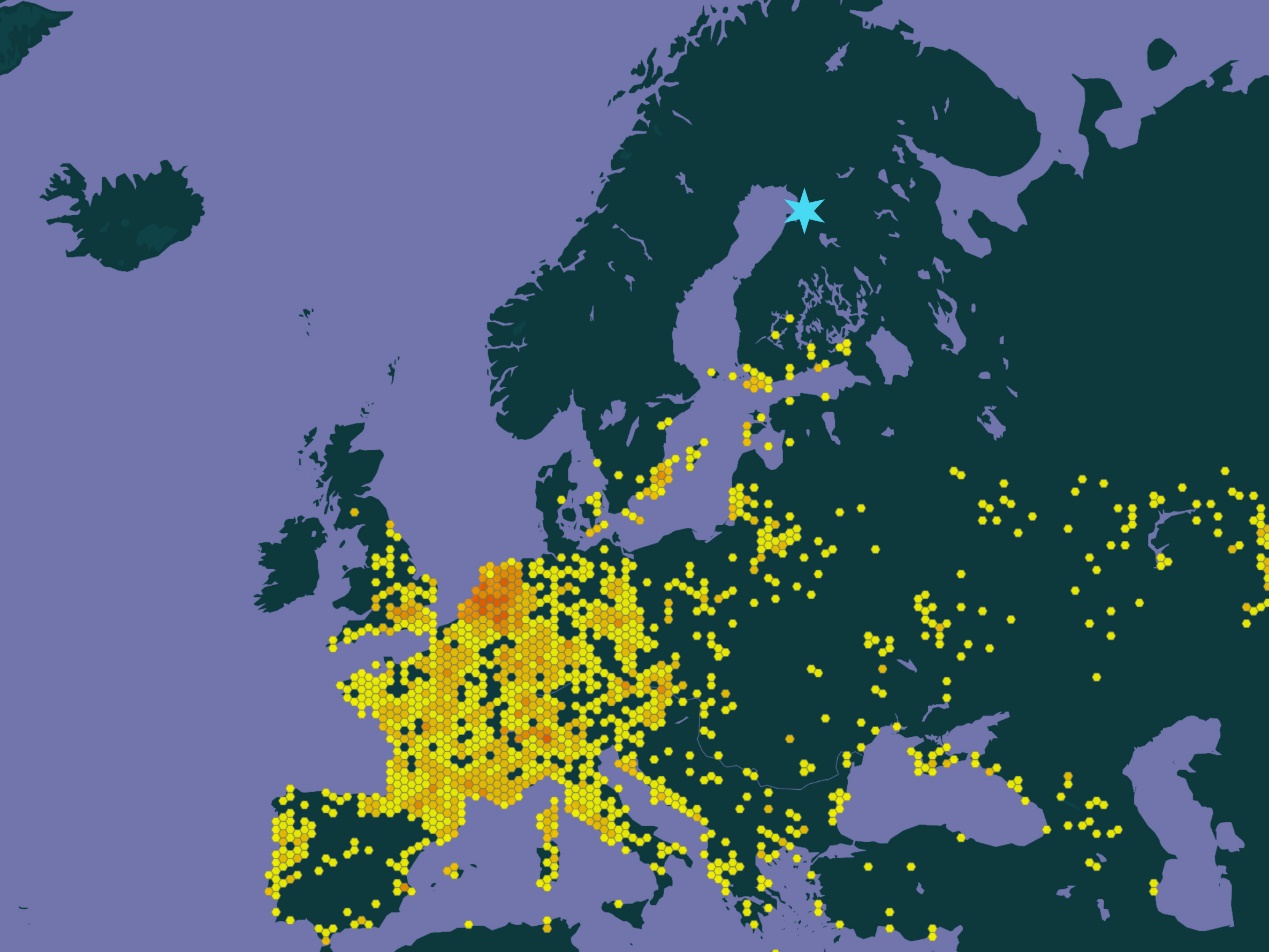


Source: GBIF.org (18 May 2022) GBIF Occurrence Download https://doi.org/10.15468/dl.dzbb4v

12 *Lymantria monacha*


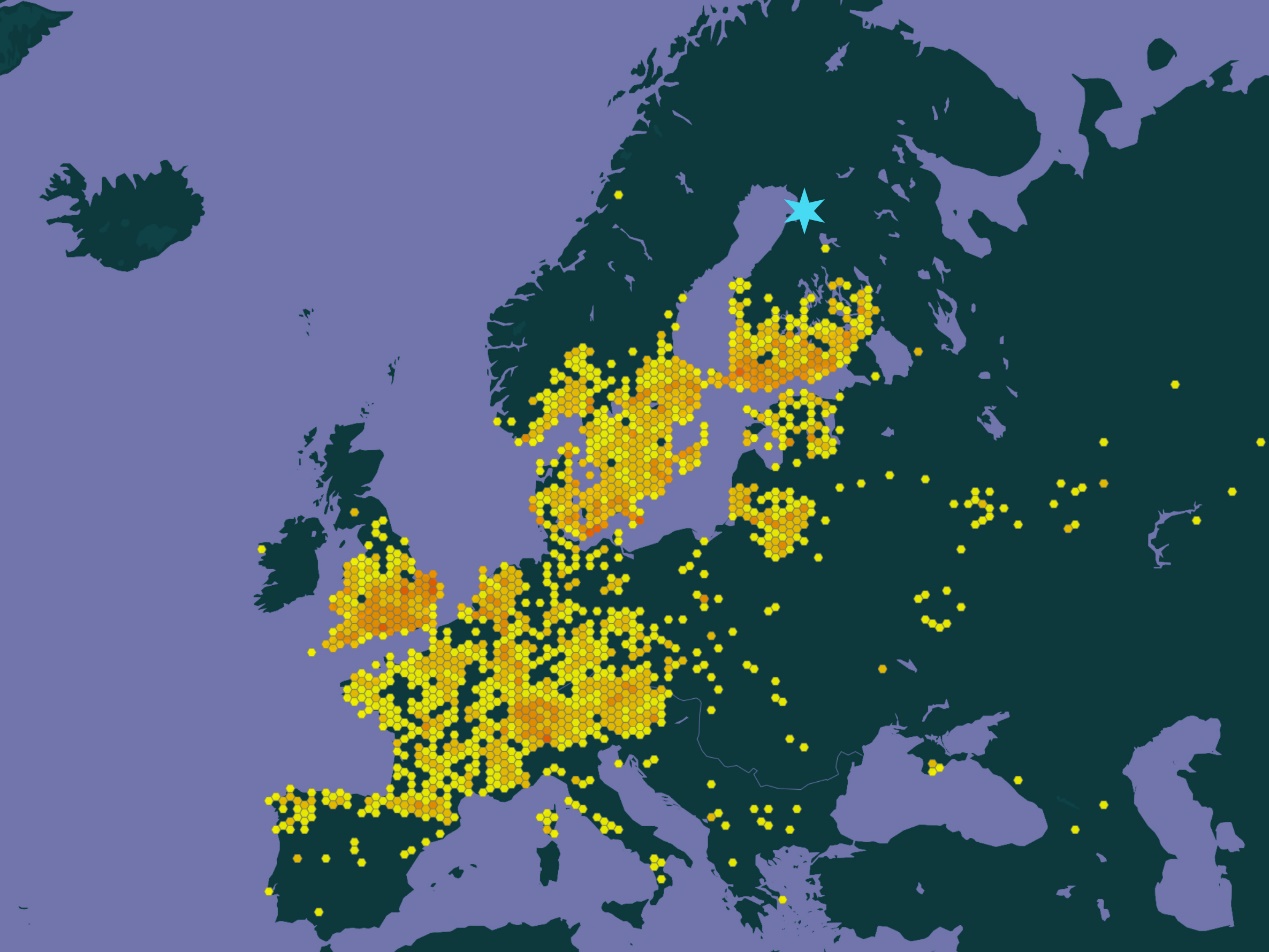


Source: GBIF.org (18 May 2022) GBIF Occurrence Download https://doi.org/10.15468/dl.4nr6n8

13 *Malacosoma neustria*


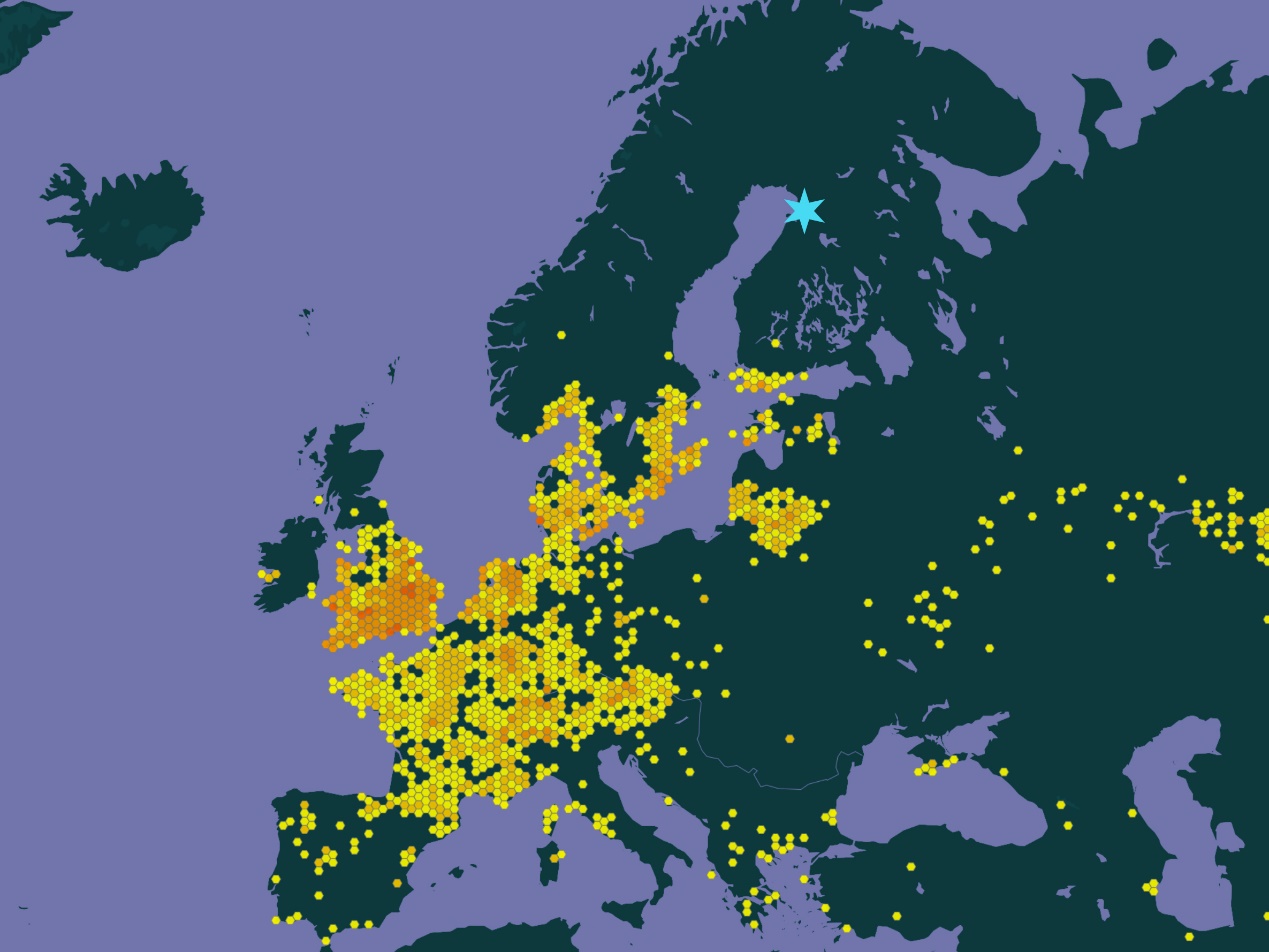


Source: GBIF.org (18 May 2022) GBIF Occurrence Download https://doi.org/10.15468/dl.jf9bnw

14 *Menophra abruptaria*


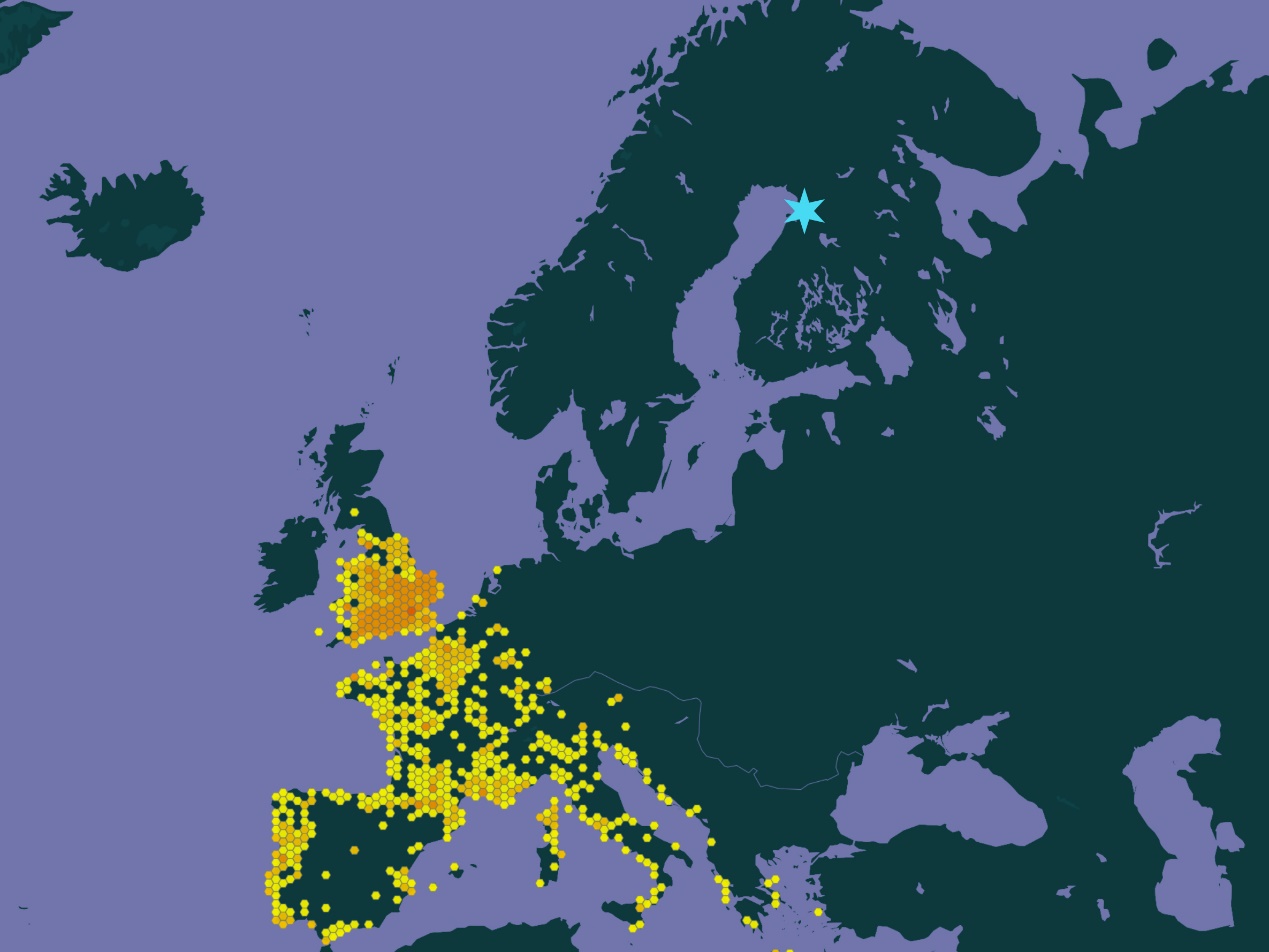


Source: GBIF.org (18 May 2022) GBIF Occurrence Download https://doi.org/10.15468/dl.9etqxq

15 *Minucia lunaris*


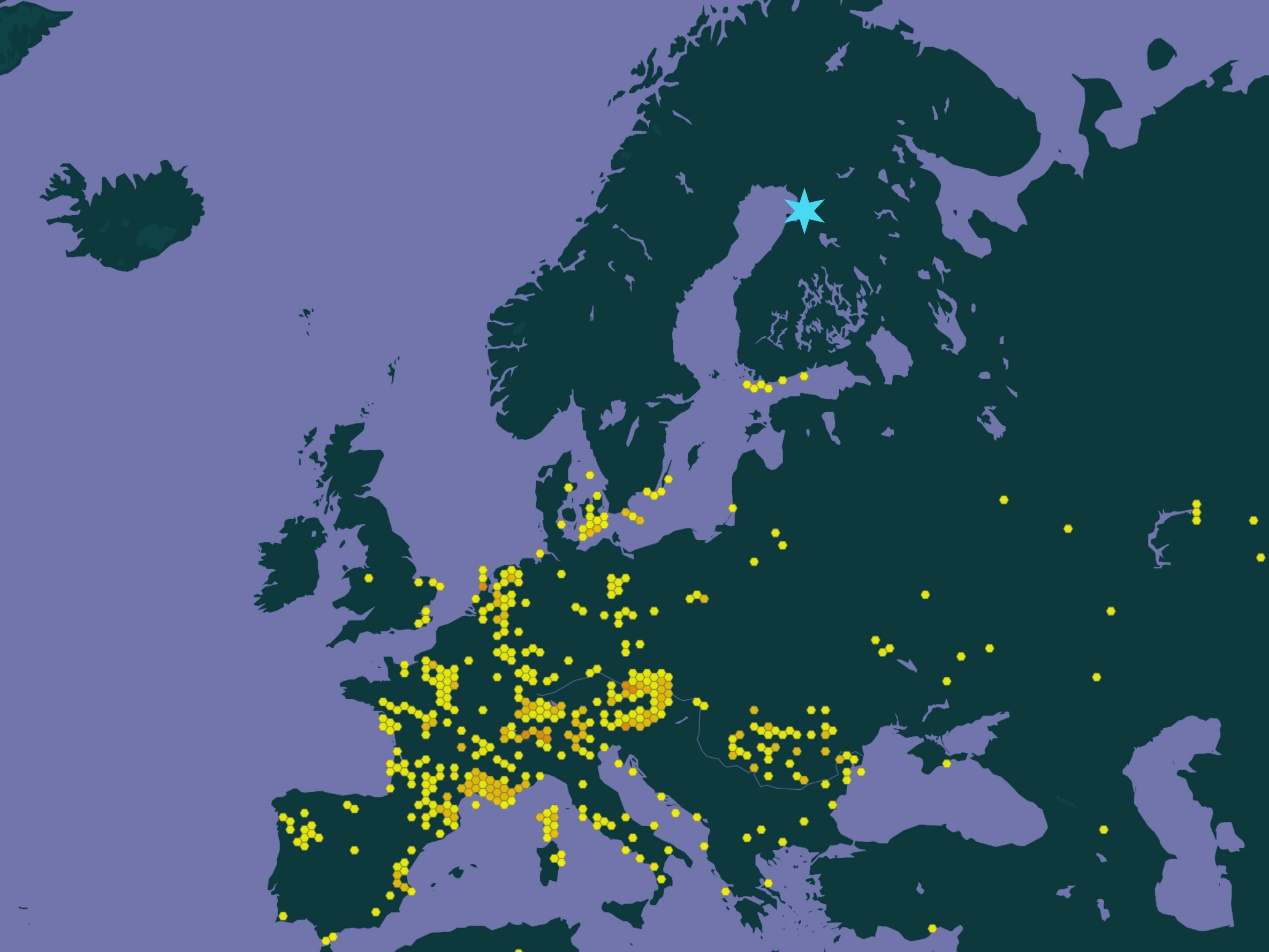


Source: GBIF.org (18 May 2022) GBIF Occurrence Download https://doi.org/10.15468/dl.ye2xkd

16 *Ocneria rubea*


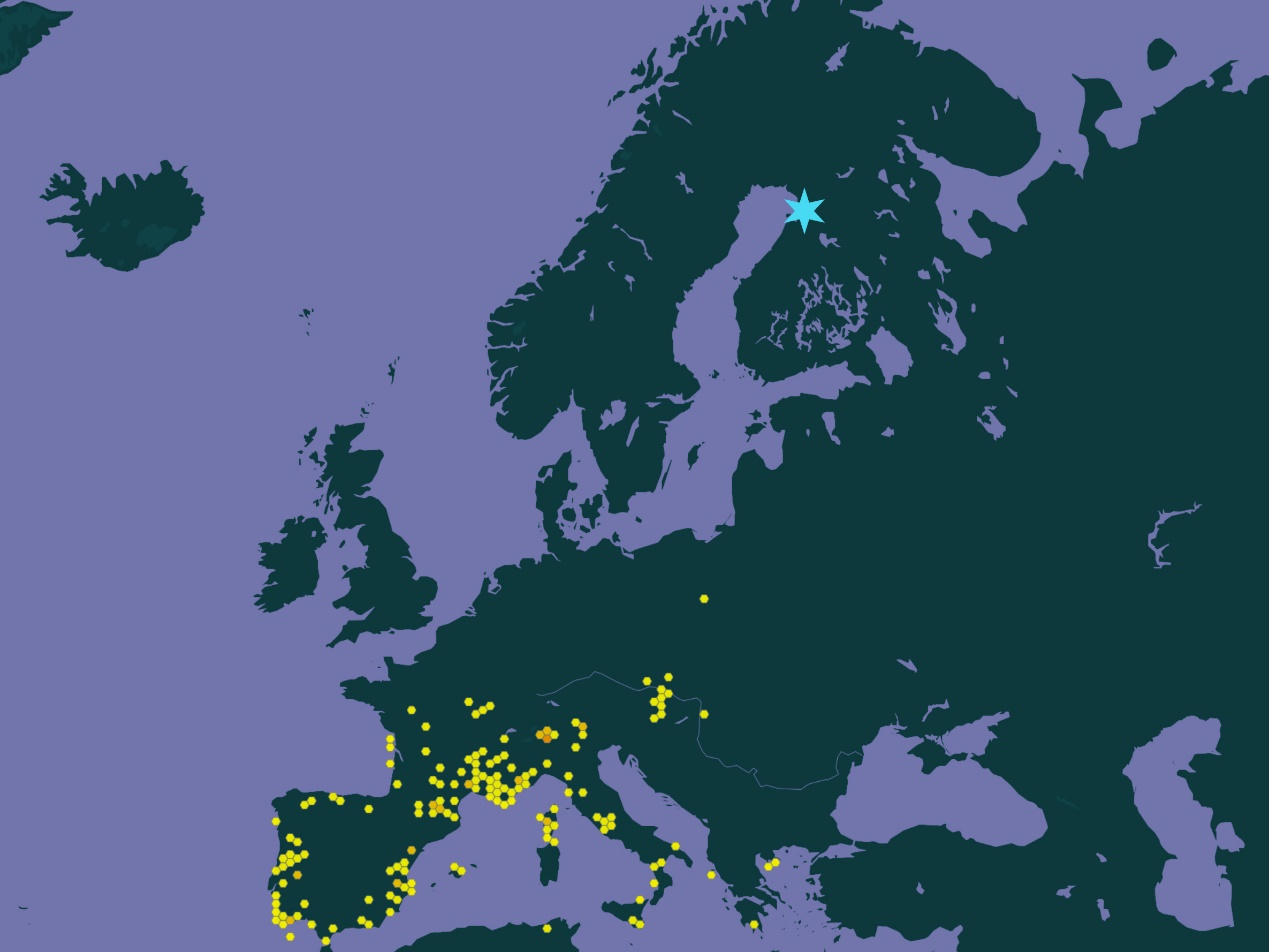


Source: GBIF.org (18 May 2022) GBIF Occurrence Download https://doi.org/10.15468/dl.geupjq

17 *Peridea anceps*


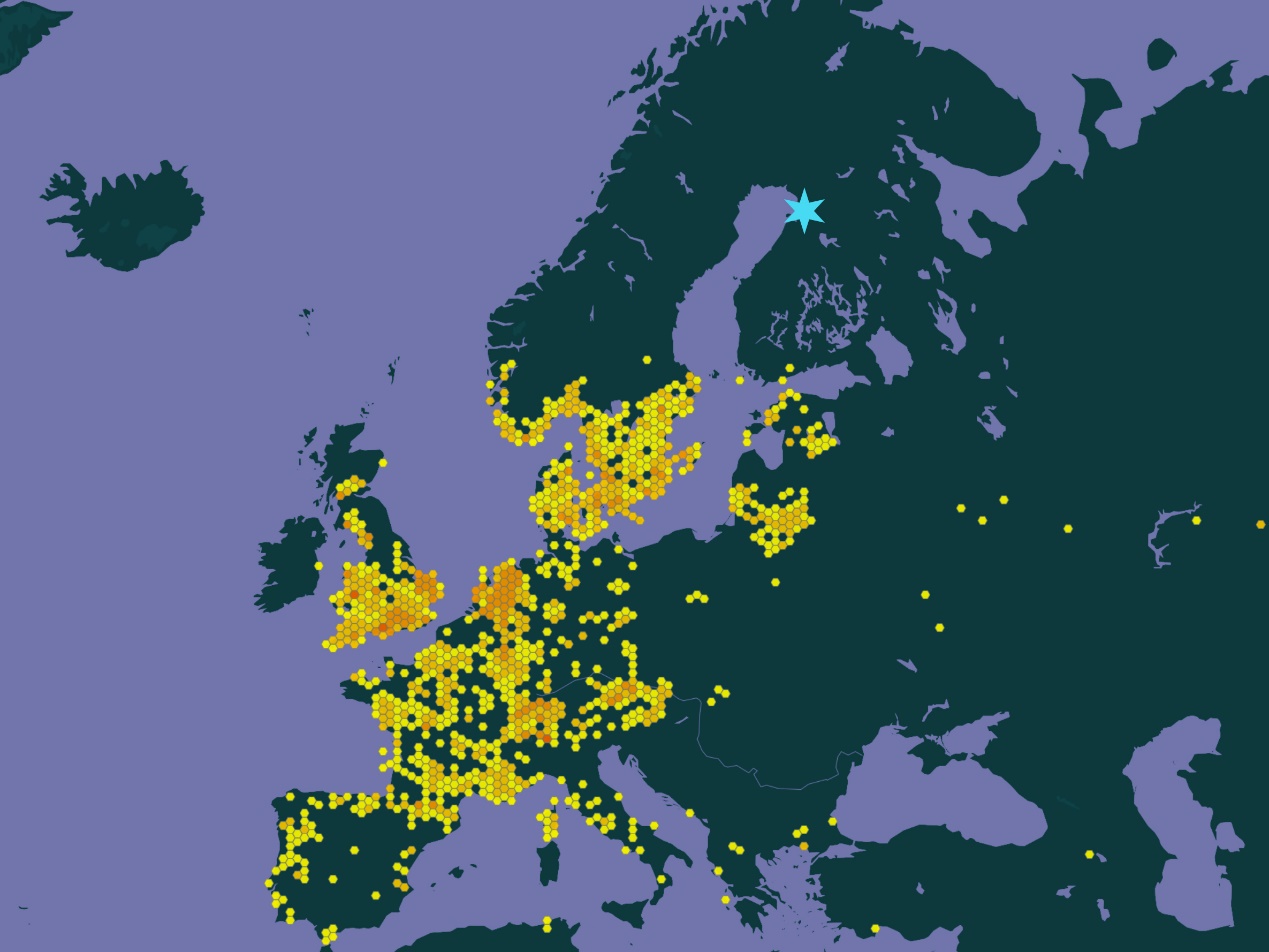


Source: GBIF.org (18 May 2022) GBIF Occurrence Download https://doi.org/10.15468/dl.g3u8he

18 *Rileyiana fovea*


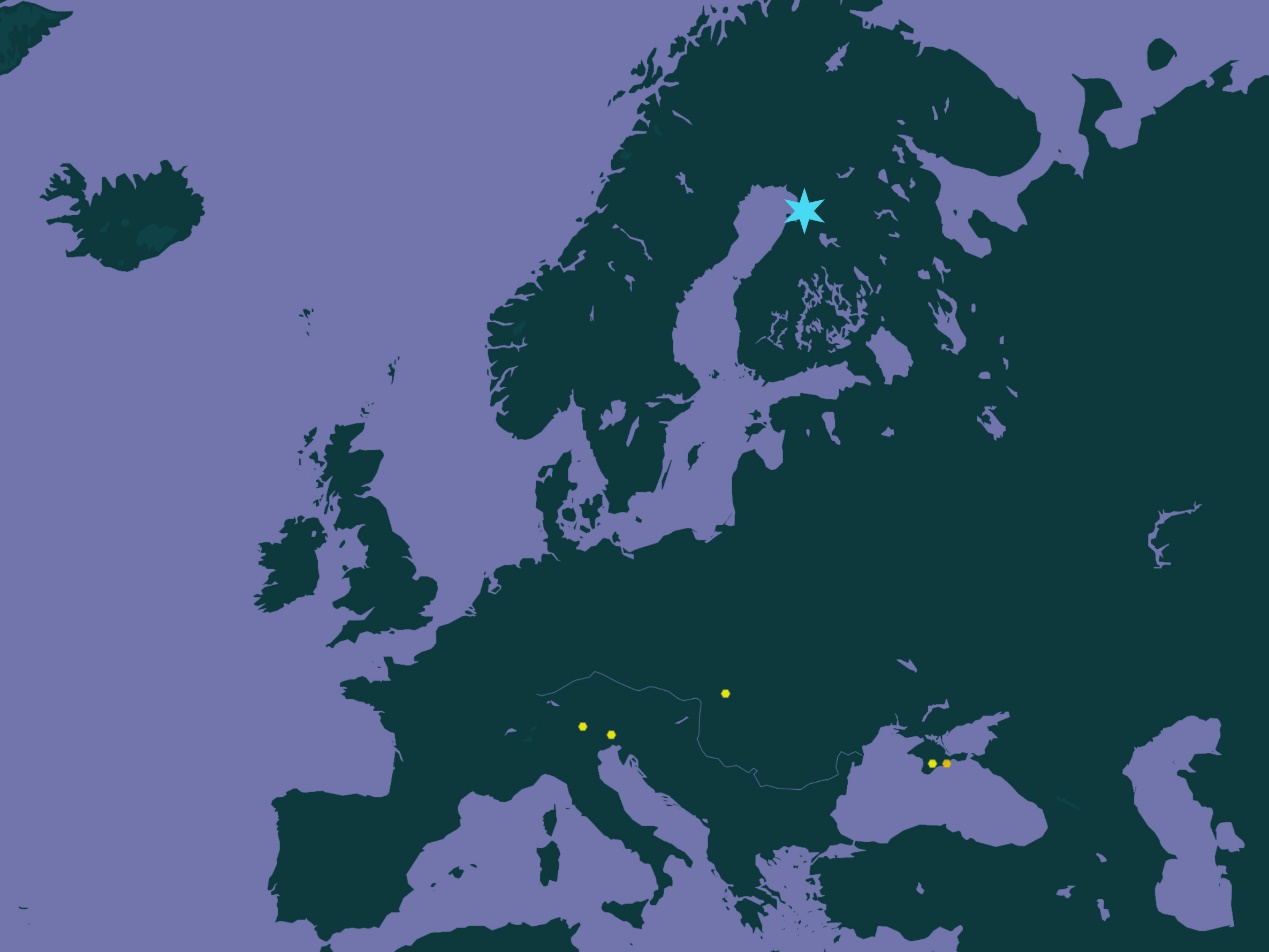


Source: GBIF.org (18 May 2022) GBIF Occurrence Download https://doi.org/10.15468/dl.jtdgw3

19 *Satyrium esculi*


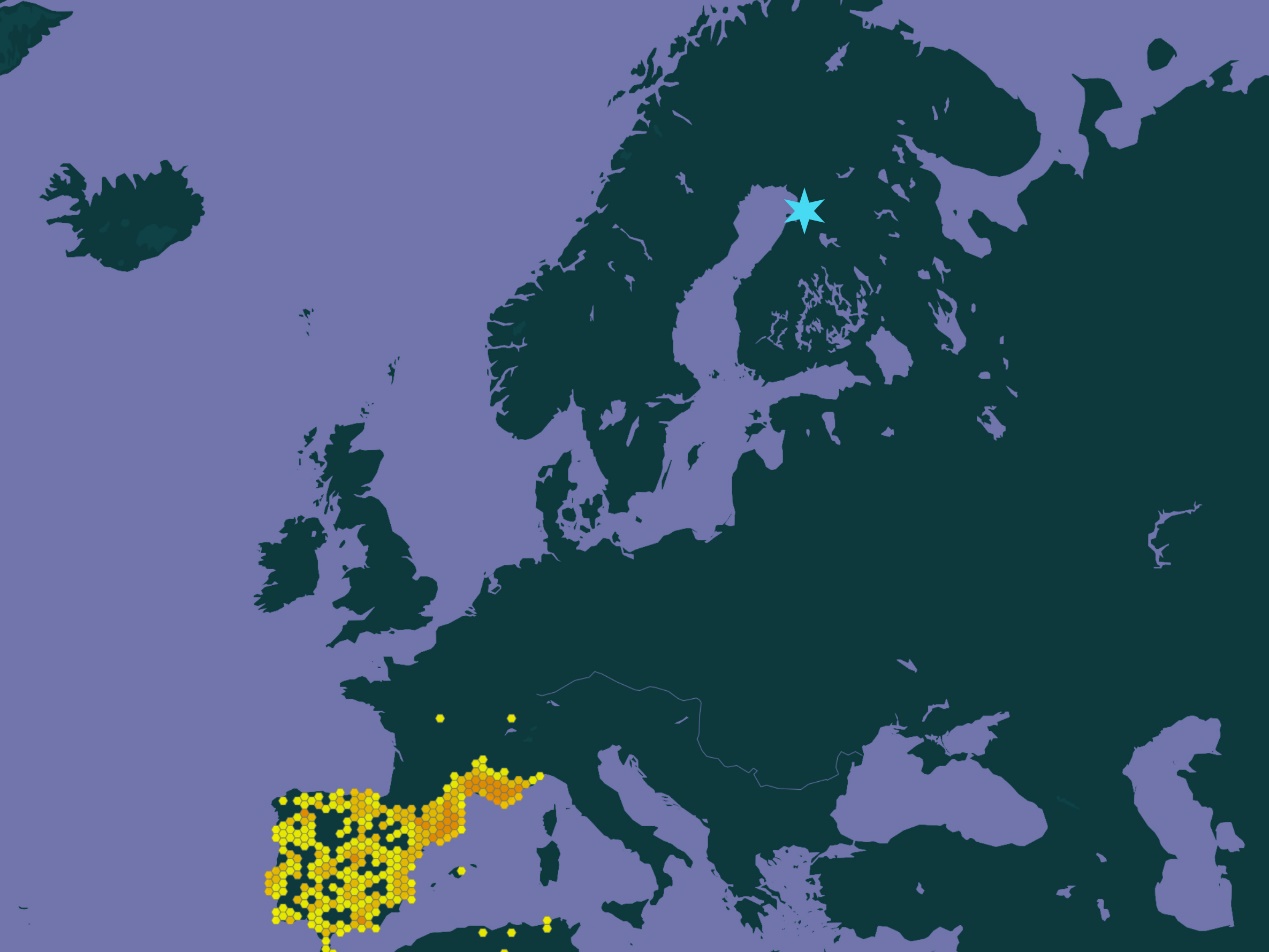


Source: GBIF.org (18 May 2022) GBIF Occurrence Download https://doi.org/10.15468/dl.y9d4yk

20 *Xestia agathina*


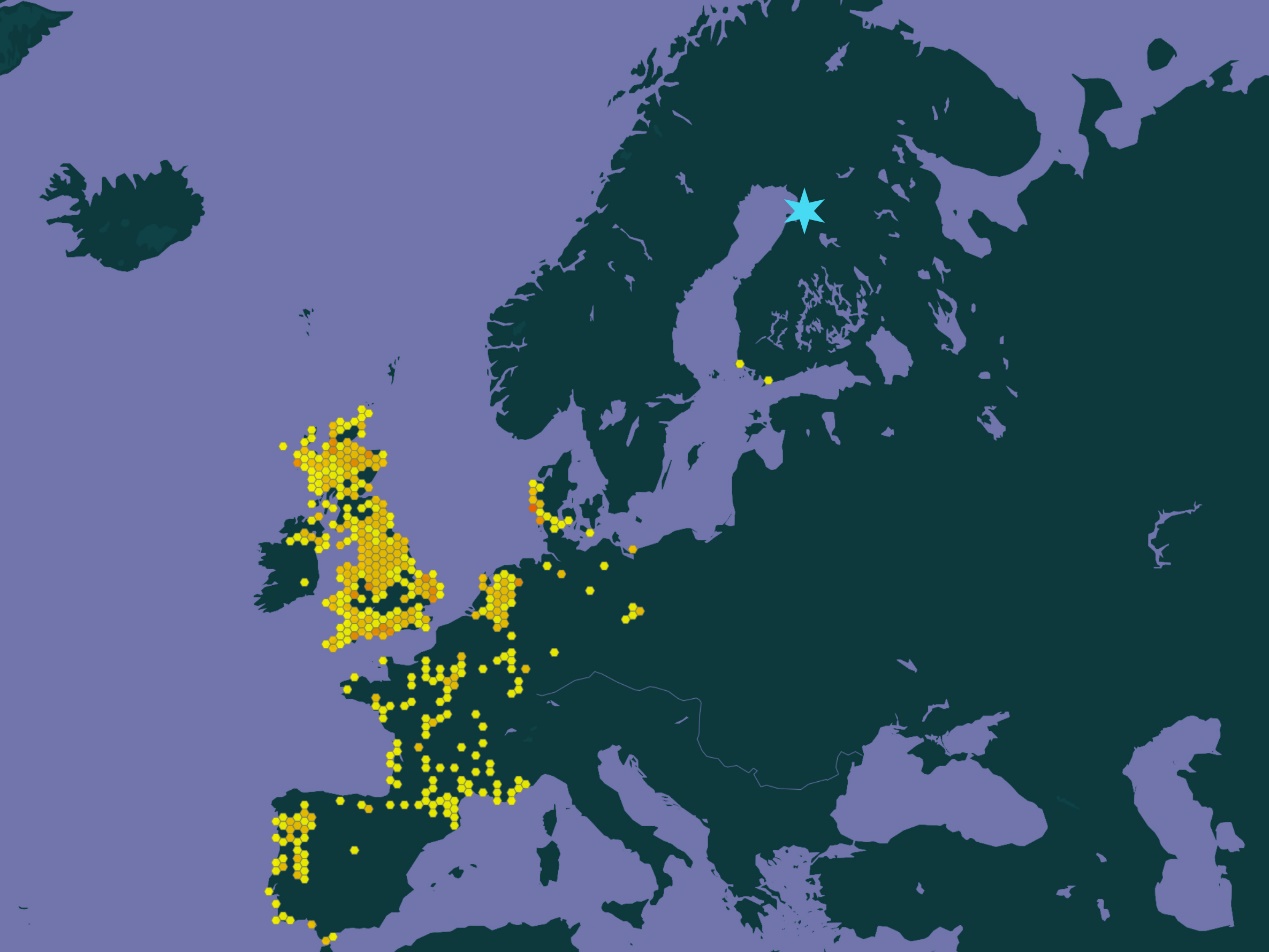


Source: GBIF.org (18 May 2022) GBIF Occurrence Download https://doi.org/10.15468/dl.7jfnz8

21 *Xylocampa areola*


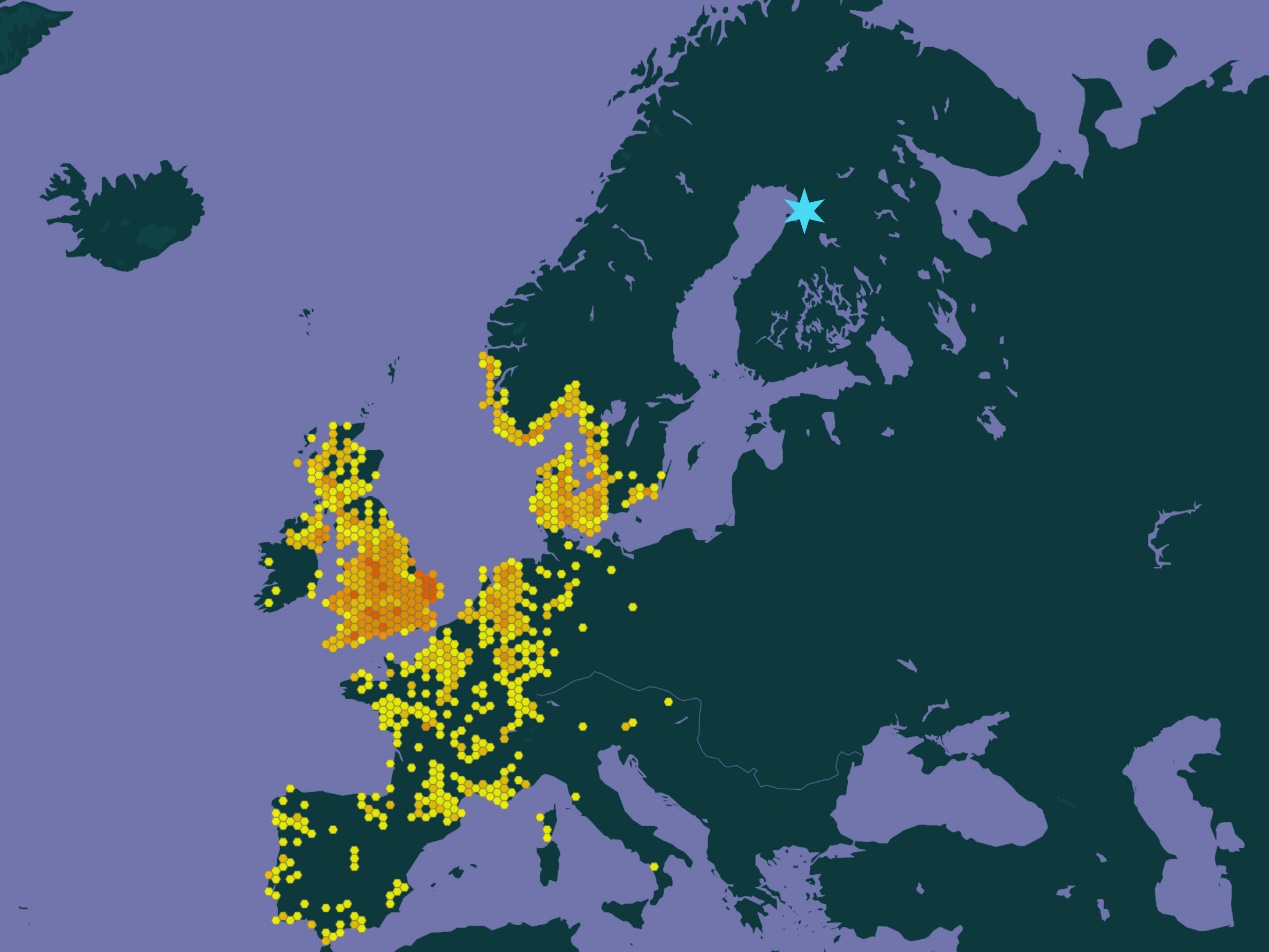


Source: GBIF.org (18 May 2022) GBIF Occurrence Download https://doi.org/10.15468/dl.2nx4wk

22 *Aethes seriatana*


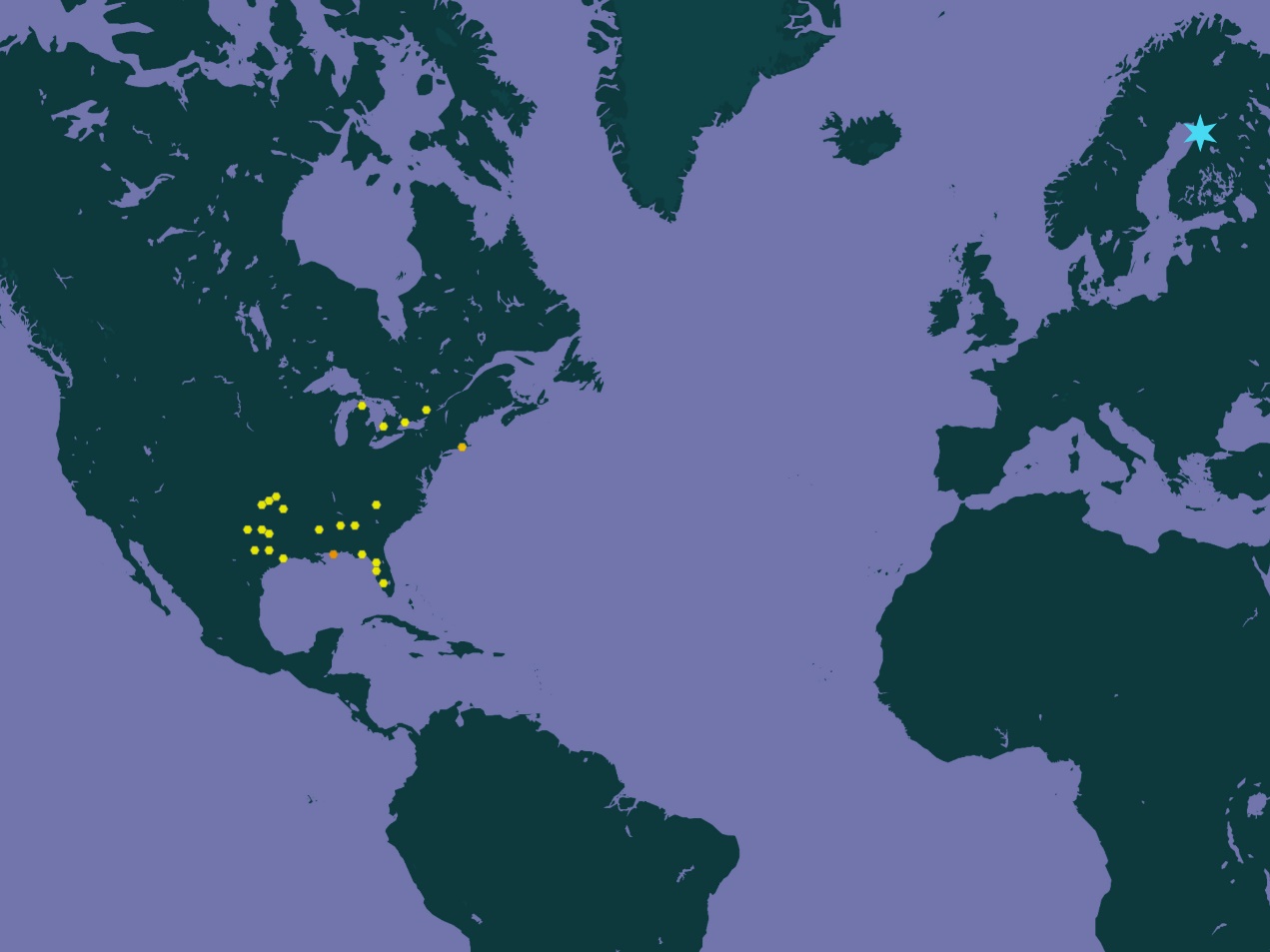


Source: GBIF.org (18 May 2022) GBIF Occurrence Download https://doi.org/10.15468/dl.fj5986

23 *Henricus cognatus* (as *Henricus cognata*)


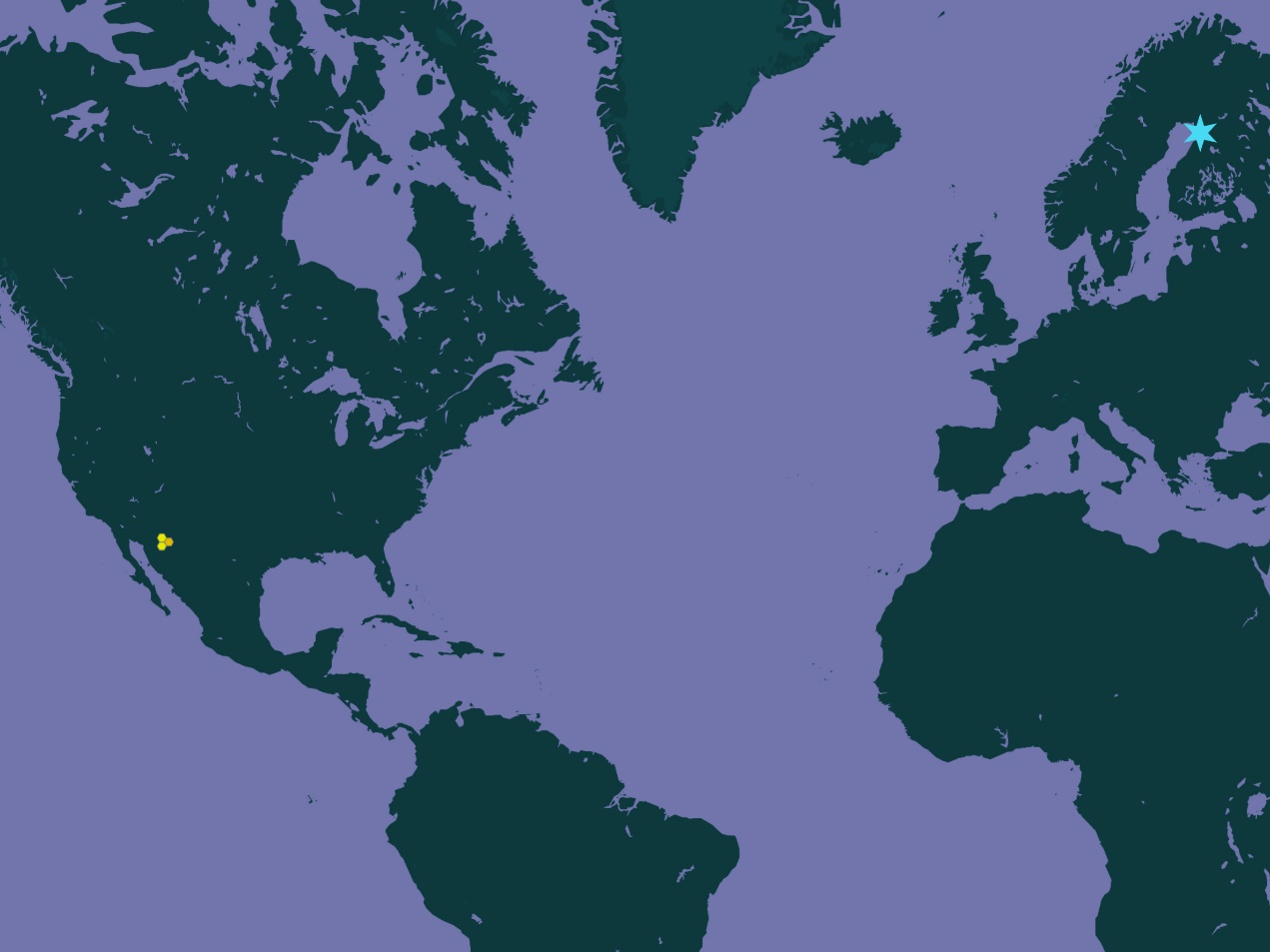


Source: GBIF.org (18 May 2022) GBIF Occurrence Download https://doi.org/10.15468/dl.82cjnx

24 *Henricus umbrabasanus*


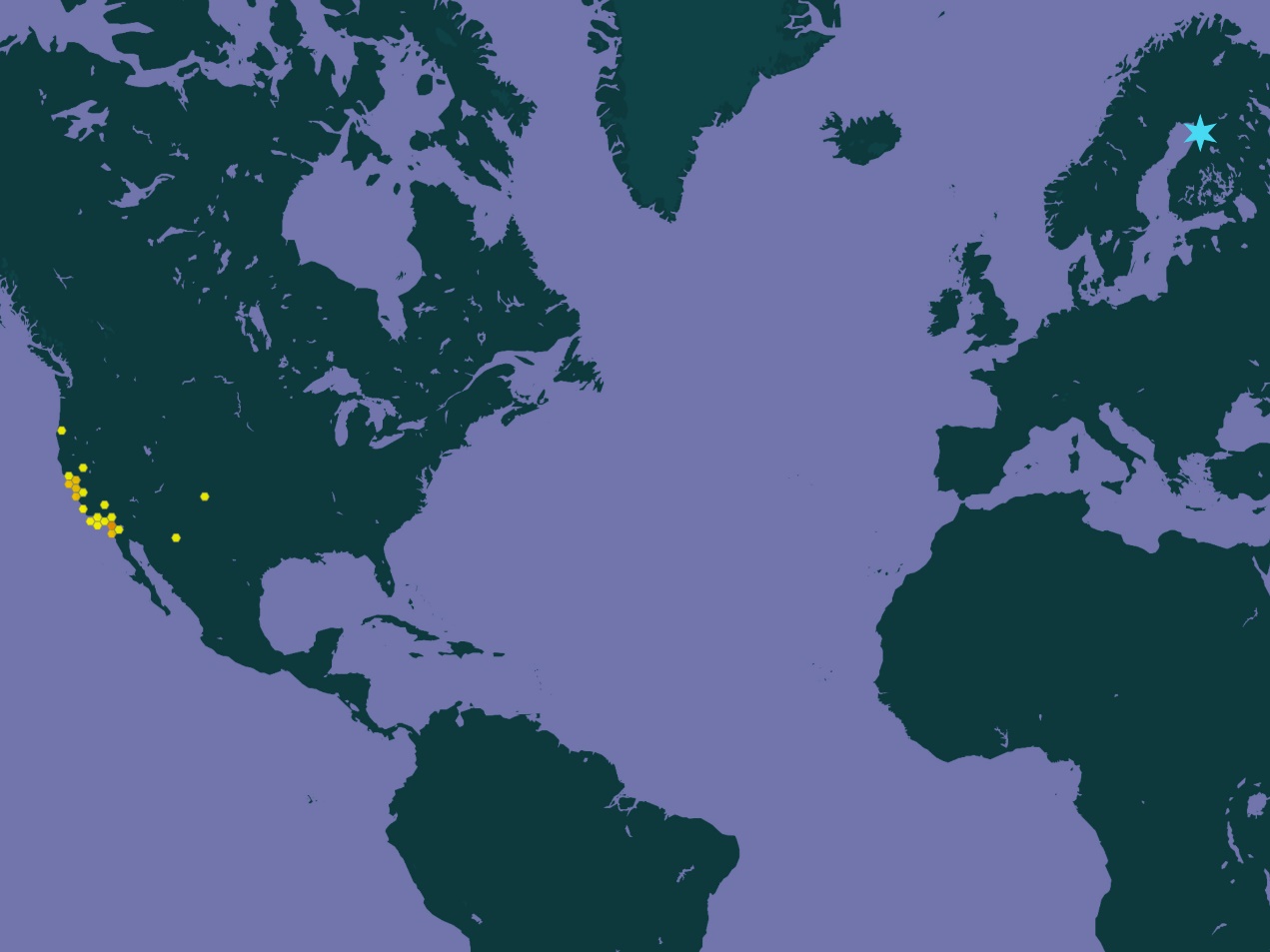


Source: GBIF.org (18 May 2022) GBIF Occurrence Download https://doi.org/10.15468/dl.cshdku

25 *Platphalonidia felix*


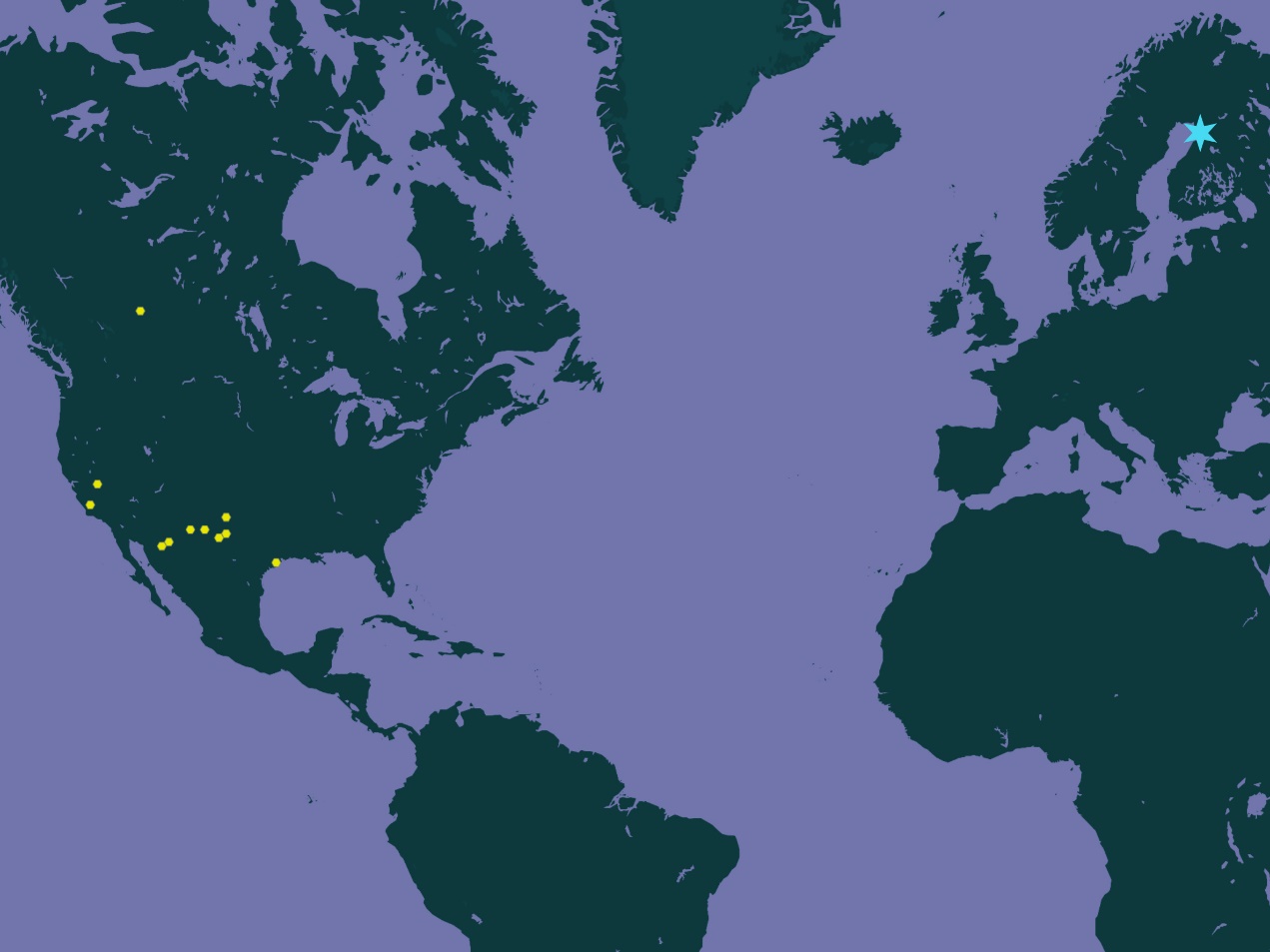


Source: GBIF.org (18 May 2022) GBIF Occurrence Download https://doi.org/10.15468/dl.zrp8zh
