# Supplementary material for: Exploring and validating observations of non‐local species in eDNA samples
Source: Ecol Evol. 2023 Oct 14;13(10):e10612. doi: 10.1002/ece3.10612 (PMC10576249; doi:10.1002/ece3.10612)
Supplement: Supplementary file 4 — Appendix S4 [file ECE3-13-e10612-s004.docx]

**Exploring and validating observations of non-local species in eDNA samples**

Coen Westerduin, Marko Suokas, Tuukka Petäjä, Ulla Saarela, Seppo Vainio, Marko Mutanen

**Supporting Information 4.** Distributions of ASVs assigned to non-local species across samples.

Figure S4A. Distribution of ASVs matching non-local species across the samples. Samples shown as columns, in order of field collection date. Different sequences are displayed as rows, with species clustered and indicated by numbers, while letters indicate variant ASVs (if any). Coloured cells indicate occurrence of the ASV, with the shade indicating the abundance. The species are: 1 *Agriopis marginaria*, 2 *Bena bicolorana*, 3 *Biston stratarius*, 4 *Campaea honoraria*, 5 *Catephia alchymista*, 6 *Catocala conjuncta*, 7 *Catocala nymphagoga*, 8 *Dryobotodes tenebrosa*, 9 *Gonepteryx cleopatra*, 10 *Lithosia quadra*, 11 *Lymantria dispar*, 12 *Lymantria monacha*, 13 *Malacosoma neustria*, 14 *Menophra abruptaria*, 15 *Minucia lunaris*, 16 *Ocneria rubea*, 17 *Peridea anceps*, 18 *Rileyiana fovea*, 19 *Satyrium esculi*, 20 *Xestia agathina*, 21 *Xylocampa areola*, 22 *Aethes seriatana*, 23 *Henricus cognatus*, 24 *Henricus umbrabasanus*, 25 *Platphalonidia felix*. Sample numbers in the top row correspond to those listed in Supporting Information 1.

 Figure S4B. Distribution of ASVs matching non-local species across the samples. Samples shown as columns, in order of processing in the lab. Different sequences are displayed as rows, with species clustered and indicated by numbers, while letters indicate variant ASVs (if any). Coloured cells indicate occurrence of the ASV, with the shade indicating the abundance. The species are: 1 *Agriopis marginaria*, 2 *Bena bicolorana*, 3 *Biston stratarius*, 4 *Campaea honoraria*, 5 *Catephia alchymista*, 6 *Catocala conjuncta*, 7 *Catocala nymphagoga*, 8 *Dryobotodes tenebrosa*, 9 *Gonepteryx cleopatra*, 10 *Lithosia quadra*, 11 *Lymantria dispar*, 12 *Lymantria monacha*, 13 *Malacosoma neustria*, 14 *Menophra abruptaria*, 15 *Minucia lunaris*, 16 *Ocneria rubea*, 17 *Peridea anceps*, 18 *Rileyiana fovea*, 19 *Satyrium esculi*, 20 *Xestia agathina*, 21 *Xylocampa areola*, 22 *Aethes seriatana*, 23 *Henricus cognatus*, 24 *Henricus umbrabasanus*, 25 *Platphalonidia felix*. Sample numbers in the top row correspond to those listed in Supporting Information 1.
