# Supplementary material for: Exploring and validating observations of non‐local species in eDNA samples
Source: Ecol Evol. 2023 Oct 14;13(10):e10612. doi: 10.1002/ece3.10612 (PMC10576249; doi:10.1002/ece3.10612)
Supplement: Supplementary file 5 — Appendix S5 [file ECE3-13-e10612-s002.pdf]

Exploring and validating observations of non-local species in eDNA samples

Coen Westerduin, Marko Suokas, Tuukka Petäjä, Ulla Saarela,

Seppo Vainio, Marko Mutanen

Supporting Information 5. Neighbor-Joining tree for all 952 ASVs from the original 2017 dataset. Total branch length is 31.83958653. Distances are calculated as per Maximum Composite Likelihood. The assigned identities are given, followed by a number indicating the BOLD matching percentage. Note that complete overlap between query and output has not been verified for all sequences that were assigned local species. Numbers in brackets differentiate otherwise identical names (i.e., same taxon and same percentage). The ASVs belonging to non-local species are highlighted in yellow, and include the identifying codes (1–25, and any letters) used elswhere in the study. Some ASVs assigned to non-local species will lack these markings as they represent the rare sequences filtered out due to low occurrences, and these were left out of the main analyses presented in the study as a result.

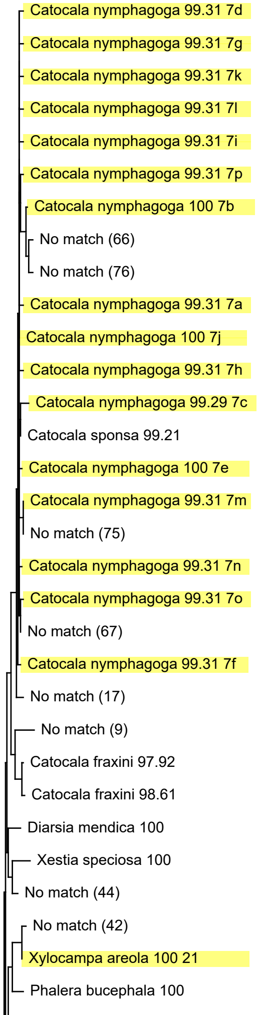

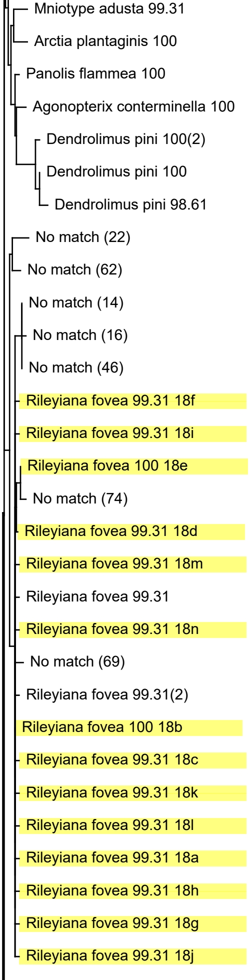

Mniotype adusta 99.31
Arctia plantaginis 100
Panolis flammea 100
Agonopterix conterminella 100
Dendrolimus pini 100(2)
Dendrolimus pini 100
Dendrolimus pini 98.61
No match (22)
No match (62)
No match (14)
No match (16)
No match (46)
Rileyiana fovea 99.31 18f
Rileyiana fovea 99.31 18i
Rileyiana fovea 100 18e
No match (74)
Rileyiana fovea 99.31 18d
Rileyiana fovea 99.31 18m
Rileyiana fovea 99.31
Rileyiana fovea 99.31 18n
No match (69)
Rileyiana fovea 99.31(2)
Rileyiana fovea 100 18b
Rileyiana fovea 99.31 18c
Rileyiana fovea 99.31 18k
Rileyiana fovea 99.31 18l
Rileyiana fovea 99.31 18a
Rileyiana fovea 99.31 18h
Rileyiana fovea 99.31 18g
Rileyiana fovea 99.31 18j

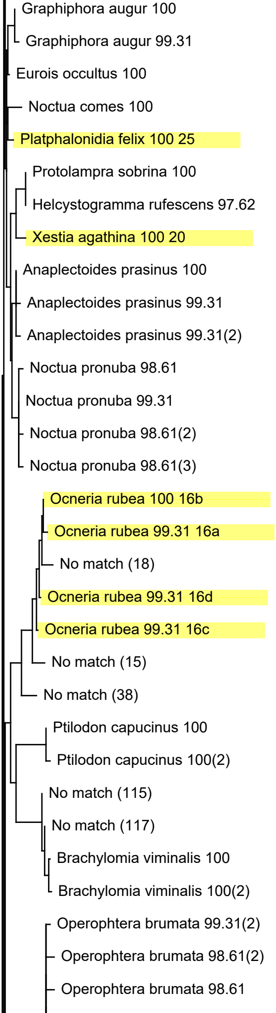
Graphiphora augur 100
Graphiphora augur 99.31
Eurois occultus 100
Noctua comes 100
Platphalonidia felix 100 25
Protolampra sobrina 100
Helcystogramma rufescens 97.62
Xestia agathina 100 20
Anaplectoides prasinus 100
Anaplectoides prasinus 99.31
Anaplectoides prasinus 99.31(2)
Noctua pronuba 98.61
Noctua pronuba 99.31
Noctua pronuba 98.61(2)
Noctua pronuba 98.61(3)
Ocneria rubea 100 16b
Ocneria rubea 99.31 16a
No match (18)
Ocneria rubea 99.31 16d
Ocneria rubea 99.31 16c
No match (15)
No match (38)
Ptilodon capucinus 100
Ptilodon capucinus 100(2)
No match (115)
No match (117)
Brachylomia viminalis 100
Brachylomia viminalis 100(2)
Operophtera brumata 99.31(2)
Operophtera brumata 98.61(2)
Operophtera brumata 98.61

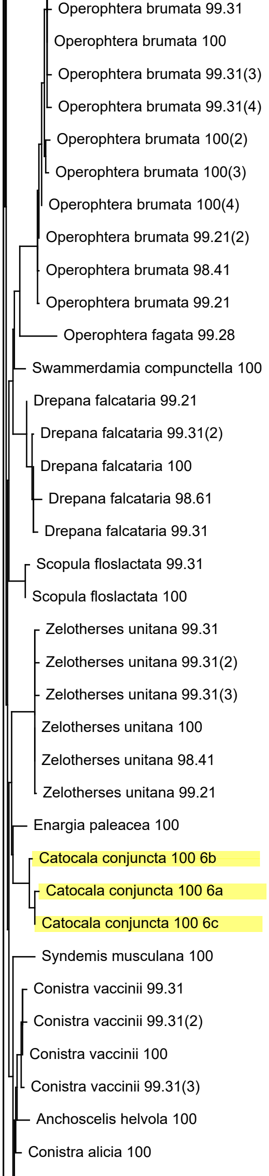
Operophtera brumata 99.31
Operophtera brumata 100
Operophtera brumata 99.31(3)
Operophtera brumata 99.31(4)
Operophtera brumata 100(2)
Operophtera brumata 100(3)
Operophtera brumata 100(4)
Operophtera brumata 99.21(2)
Operophtera brumata 98.41
Operophtera brumata 99.21
Operophtera fagata 99.28
Swammerdamia compunctella 100
Drepana falcataria 99.21
Drepana falcataria 99.31(2)
Drepana falcataria 100
Drepana falcataria 98.61
Drepana falcataria 99.31
Scopula floslactata 99.31
Scopula floslactata 100
Zelotherses unitana 99.31
Zelotherses unitana 99.31(2)
Zelotherses unitana 99.31(3)
Zelotherses unitana 100
Zelotherses unitana 98.41
Zelotherses unitana 99.21
Enargia paleacea 100
Catocala conjuncta 100 6b
Catocala conjuncta 100 6a
Catocala conjuncta 100 6c
Syndemis musculana 100
Conistra vaccinii 99.31
Conistra vaccinii 99.31(2)
Conistra vaccinii 100
Conistra vaccinii 99.31(3)
Anchoscelis helvola 100
Conistra alicia 100

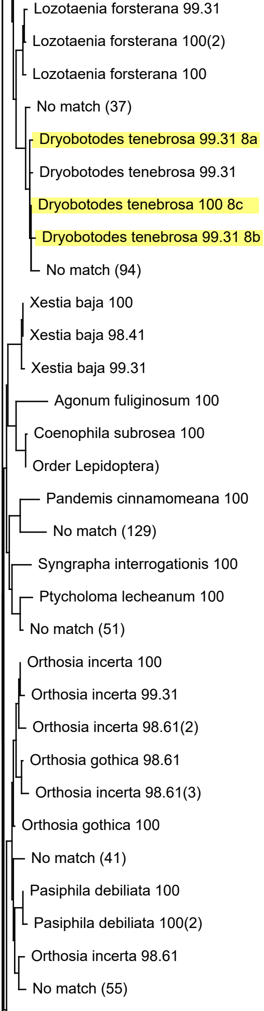
Lozotaenia forsterana 99.31
Lozotaenia forsterana 100(2)
Lozotaenia forsterana 100
No match (37)
Dryobotodes tenebrosa 99.31 8a
Dryobotodes tenebrosa 99.31
Dryobotodes tenebrosa 100 8c
Dryobotodes tenebrosa 99.31 8b
No match (94)
Xestia baja 100
Xestia baja 98.41
Xestia baja 99.31
Agonum fuliginosum 100
Coenophila subrosea 100
Order Lepidoptera)
Pandemis cinnamomeana 100
No match (129)
Syngrapha interrogationis 100
Ptycholoma lecheanum 100
No match (51)
Orthosia incerta 100
Orthosia incerta 99.31
Orthosia incerta 98.61(2)
Orthosia gothica 98.61
Orthosia incerta 98.61(3)
Orthosia gothica 100
No match (41)
Pasiphila debiliata 100
Pasiphila debiliata 100(2)
Orthosia incerta 98.61
No match (55)

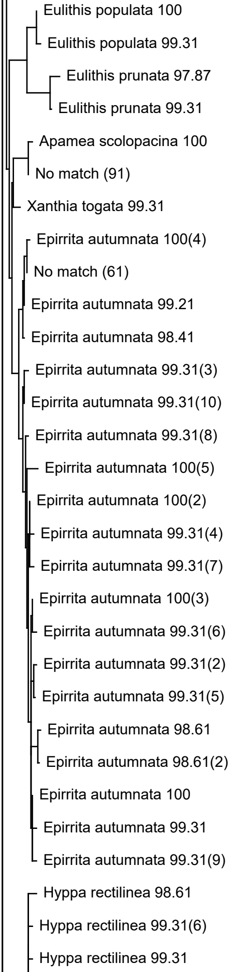
Eulithis populata 100
Eulithis populata 99.31
Eulithis prunata 97.87
Eulithis prunata 99.31
Apamea scolopacina 100
No match (91)
Xanthia togata 99.31
Epirrita autumnata 100(4)
No match (61)
Epirrita autumnata 99.21
Epirrita autumnata 98.41
Epirrita autumnata 99.31(3)
Epirrita autumnata 99.31(10)
Epirrita autumnata 99.31(8)
Epirrita autumnata 100(5)
Epirrita autumnata 100(2)
Epirrita autumnata 99.31(4)
Epirrita autumnata 99.31(7)
Epirrita autumnata 100(3)
Epirrita autumnata 99.31(6)
Epirrita autumnata 99.31(2)
Epirrita autumnata 99.31(5)
Epirrita autumnata 98.61
Epirrita autumnata 98.61(2)
Epirrita autumnata 100
Epirrita autumnata 99.31
Epirrita autumnata 99.31(9)
Hyppa rectilinea 98.61
Hyppa rectilinea 99.31(6)
Hyppa rectilinea 99.31

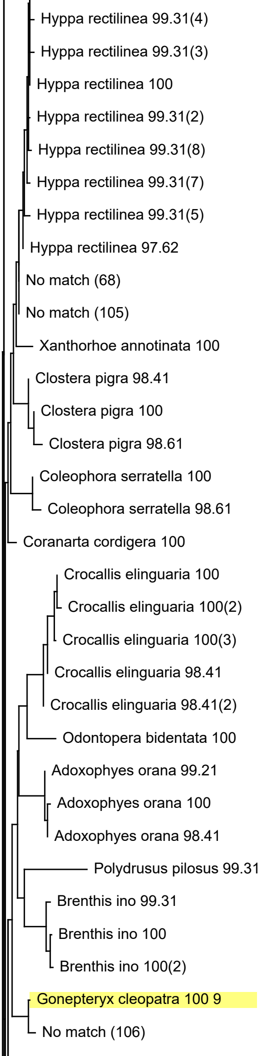
Hyppa rectilinea 99.31(4)
Hyppa rectilinea 99.31(3)
Hyppa rectilinea 100
Hyppa rectilinea 99.31(2)
Hyppa rectilinea 99.31(8)
Hyppa rectilinea 99.31(7)
Hyppa rectilinea 99.31(5)
Hyppa rectilinea 97.62
No match (68)
No match (105)
Xanthorhoe annotinata 100
Clostera pigra 98.41
Clostera pigra 100
Clostera pigra 98.61
Coleophora serratella 100
Coleophora serratella 98.61
Coranarta cordigera 100
Crocallis elinguaria 100
Crocallis elinguaria 100(2)
Crocallis elinguaria 100(3)
Crocallis elinguaria 98.41
Crocallis elinguaria 98.41(2)
Odontopera bidentata 100
Adoxophyes orana 99.21
Adoxophyes orana 100
Adoxophyes orana 98.41
Polydrusus pilosus 99.31
Brenthis ino 99.31
Brenthis ino 100
Brenthis ino 100(2)
Gonepteryx cleopatra 100 9
No match (106)

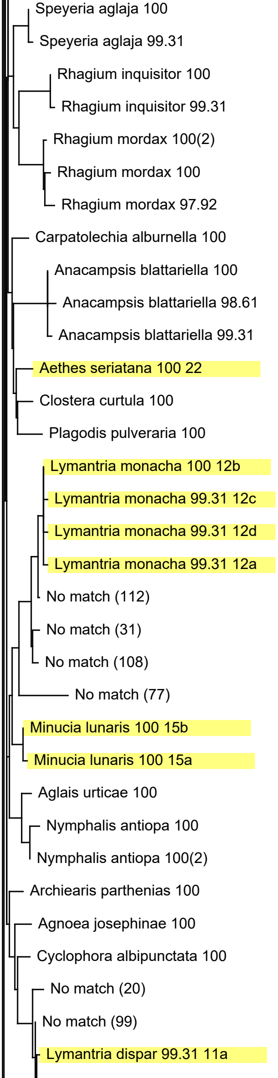
Speyeria aglaja 100
Speyeria aglaja 99.31
Rhagium inquisitor 100
Rhagium inquisitor 99.31
Rhagium mordax 100(2)
Rhagium mordax 100
Rhagium mordax 97.92
Carpatolechia alburnella 100
Anacampsis blattariella 100
Anacampsis blattariella 98.61
Anacampsis blattariella 99.31
Aethes seriatana 100 22
Clostera curtula 100
Plagodis pulveraria 100
Lymantria monacha 100 12b
Lymantria monacha 99.31 12c
Lymantria monacha 99.31 12d
Lymantria monacha 99.31 12a
No match (112)
No match (31)
No match (108)
No match (77)
Minucia lunaris 100 15b
Minucia lunaris 100 15a
Aglais urticae 100
Nymphalis antiopa 100
Nymphalis antiopa 100(2)
Archiearis parthenias 100
Agnoea josephinae 100
Cyclophora albipunctata 100
No match (20)
No match (99)
Lymantria dispar 99.31 11a

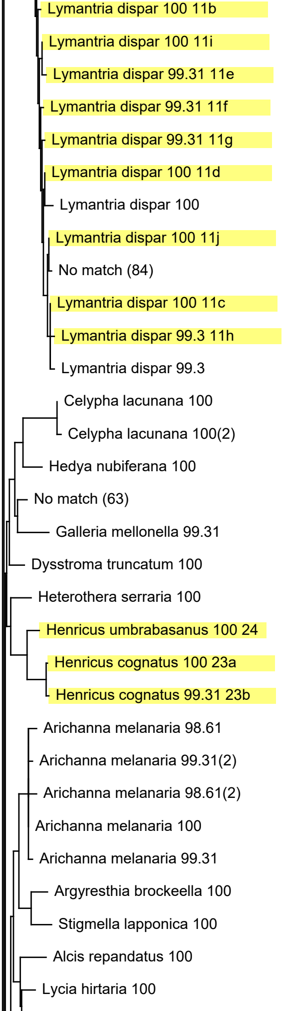

Lymantria dispar 100 11b
Lymantria dispar 100 11i
Lymantria dispar 99.31 11e
Lymantria dispar 99.31 11f
Lymantria dispar 99.31 11g
Lymantria dispar 100 11d
Lymantria dispar 100
Lymantria dispar 100 11j
No match (84)
Lymantria dispar 100 11c
Lymantria dispar 99.3 11h
Lymantria dispar 99.3
Celypha lacunana 100
Celypha lacunana 100(2)
Hedya nubiferana 100
No match (63)
Galleria mellonella 99.31
Dysstroma truncatum 100
Heterothera serraria 100
Henricus umbrabasanus 100 24
Henricus cognatus 100 23a
Henricus cognatus 99.31 23b
Arichanna melanaria 98.61
Arichanna melanaria 99.31(2)
Arichanna melanaria 98.61(2)
Arichanna melanaria 100
Arichanna melanaria 99.31
Argyresthia brockeella 100
Stigmella lapponica 100
Alcis repandatus 100
Lycia hirtaria 100

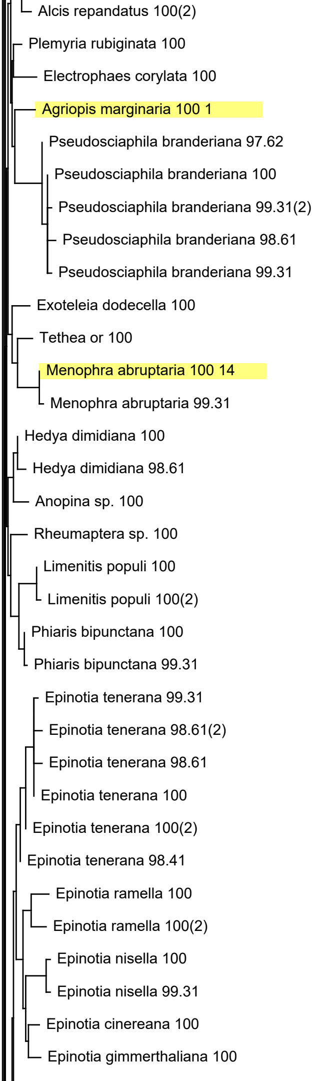
Alcis repandatus 100(2)
Plemyria rubiginata 100
Electrophaes corylata 100
Agriopis marginaria 100 1
Pseudosciaphila branderiana 97.62
Pseudosciaphila branderiana 100
Pseudosciaphila branderiana 99.31(2)
Pseudosciaphila branderiana 98.61
Pseudosciaphila branderiana 99.31
Exoteleia dodecella 100
Tethea or 100
Menophra abruptaria 100 14
Menophra abruptaria 99.31
Hedya dimidiana 100
Hedya dimidiana 98.61
Anopina sp. 100
Rheumaptera sp. 100
Limenitis populi 100
Limenitis populi 100(2)
Phiaris bipunctana 100
Phiaris bipunctana 99.31
Epinotia tenerana 99.31
Epinotia tenerana 98.61(2)
Epinotia tenerana 98.61
Epinotia tenerana 100
Epinotia tenerana 100(2)
Epinotia tenerana 98.41
Epinotia ramella 100
Epinotia ramella 100(2)
Epinotia nisella 100
Epinotia nisella 99.31
Epinotia cinereana 100
Epinotia gimmerthaliana 100

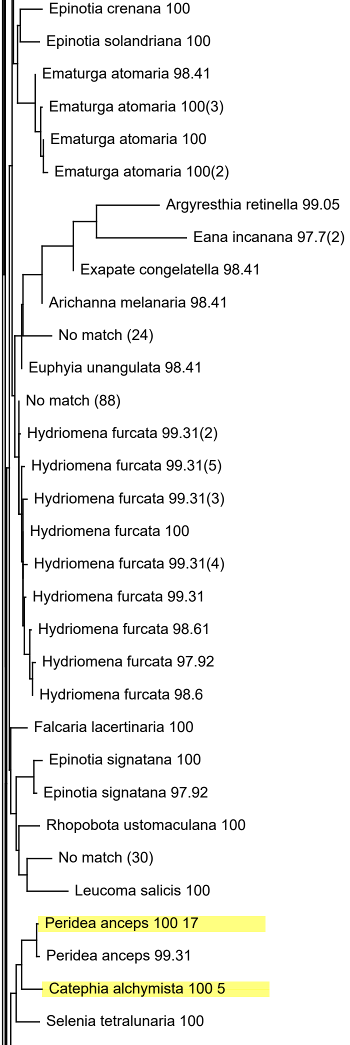
Epinotia crenana 100
Epinotia solandriana 100
Ematurga atomaria 98.41
Ematurga atomaria 100(3)
Ematurga atomaria 100
Ematurga atomaria 100(2)
Argyresthia retinella 99.05
Eana incanana 97.7(2)
Exapate congelatella 98.41
Arichanna melanaria 98.41
No match (24)
Euphyia unangulata 98.41
No match (88)
Hydriomena furcata 99.31(2)
Hydriomena furcata 99.31(5)
Hydriomena furcata 99.31(3)
Hydriomena furcata 100
Hydriomena furcata 99.31(4)
Hydriomena furcata 99.31
Hydriomena furcata 98.61
Hydriomena furcata 97.92
Hydriomena furcata 98.6
Falcaria lacertinaria 100
Epinotia signatana 100
Epinotia signatana 97.92
Rhopobota ustomaculana 100
No match (30)
Leucoma salicis 100
Peridea anceps 100 17
Peridea anceps 99.31
Catephia alchymista 100 5
Selenia tetralunaria 100

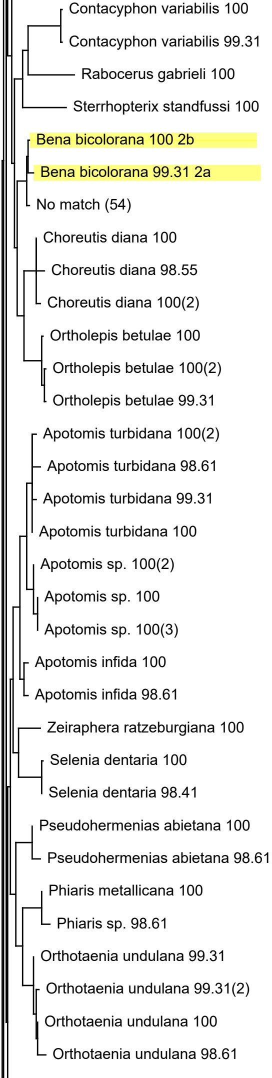
Contacyphon variabilis 100
Contacyphon variabilis 99.31
Rabocerus gabrieli 100
Sterrhopterix standfussi 100
Bena bicolorana 100 2b
Bena bicolorana 99.31 2a
No match (54)
Choreutis diana 100
Choreutis diana 98.55
Choreutis diana 100(2)
Ortholepis betulae 100
Ortholepis betulae 100(2)
Ortholepis betulae 99.31
Apotomis turbidana 100(2)
Apotomis turbidana 98.61
Apotomis turbidana 99.31
Apotomis turbidana 100
Apotomis sp. 100(2)
Apotomis sp. 100
Apotomis sp. 100(3)
Apotomis infida 100
Apotomis infida 98.61
Zeiraphera ratzeburgiana 100
Selenia dentaria 100
Selenia dentaria 98.41
Pseudohermenias abietana 100
Pseudohermenias abietana 98.61
Phiaris metallicana 100
Phiaris sp. 98.61
Orthotaenia undulana 99.31
Orthotaenia undulana 99.31(2)
Orthotaenia undulana 100
Orthotaenia undulana 98.61

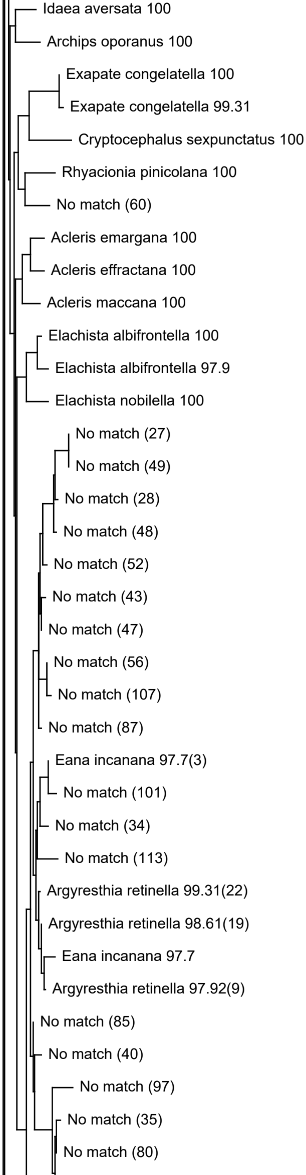
Idaea aversata 100
Archips oporanus 100
Exapate congelatella 100
Exapate congelatella 99.31
Cryptocephalus sexpunctatus 100
Rhyacionia pinicolana 100
No match (60)
Acleris emargana 100
Acleris effractana 100
Acleris maccana 100
Elachista albifrontella 100
Elachista albifrontella 97.9
Elachista nobilella 100
No match (27)
No match (49)
No match (28)
No match (48)
No match (52)
No match (43)
No match (47)
No match (56)
No match (107)
No match (87)
Eana incanana 97.7(3)
No match (101)
No match (34)
No match (113)
Argyresthia retinella 99.31(22)
Argyresthia retinella 98.61(19)
Eana incanana 97.7
Argyresthia retinella 97.92(9)
No match (85)
No match (40)
No match (97)
No match (35)
No match (80)

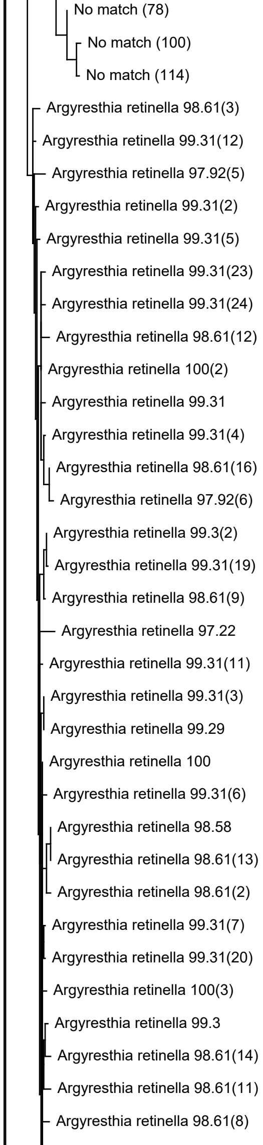
No match (78)
No match (100)
No match (114)
Argyresthia retinella 98.61(3)
Argyresthia retinella 99.31(12)
Argyresthia retinella 97.92(5)
Argyresthia retinella 99.31(2)
Argyresthia retinella 99.31(5)
Argyresthia retinella 99.31(23)
Argyresthia retinella 99.31(24)
Argyresthia retinella 98.61(12)
Argyresthia retinella 100(2)
Argyresthia retinella 99.31
Argyresthia retinella 99.31(4)
Argyresthia retinella 98.61(16)
Argyresthia retinella 97.92(6)
Argyresthia retinella 99.3(2)
Argyresthia retinella 99.31(19)
Argyresthia retinella 98.61(9)
Argyresthia retinella 97.22
Argyresthia retinella 99.31(11)
Argyresthia retinella 99.31(3)
Argyresthia retinella 99.29
Argyresthia retinella 100
Argyresthia retinella 99.31(6)
Argyresthia retinella 98.58
Argyresthia retinella 98.61(13)
Argyresthia retinella 98.61(2)
Argyresthia retinella 99.31(7)
Argyresthia retinella 99.31(20)
Argyresthia retinella 100(3)
Argyresthia retinella 99.3
Argyresthia retinella 98.61(14)
Argyresthia retinella 98.61(11)
Argyresthia retinella 98.61(8)

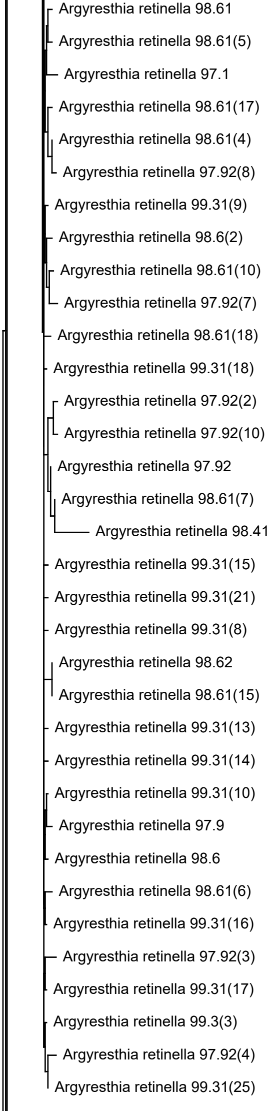
Argyresthia retinella 98.61
Argyresthia retinella 98.61(5)
Argyresthia retinella 97.1
Argyresthia retinella 98.61(17)
Argyresthia retinella 98.61(4)
Argyresthia retinella 97.92(8)
Argyresthia retinella 99.31(9)
Argyresthia retinella 98.6(2)
Argyresthia retinella 98.61(10)
Argyresthia retinella 97.92(7)
Argyresthia retinella 98.61(18)
Argyresthia retinella 99.31(18)
Argyresthia retinella 97.92(2)
Argyresthia retinella 97.92(10)
Argyresthia retinella 97.92
Argyresthia retinella 98.61(7)
Argyresthia retinella 98.41
Argyresthia retinella 99.31(15)
Argyresthia retinella 99.31(21)
Argyresthia retinella 99.31(8)
Argyresthia retinella 98.62
Argyresthia retinella 98.61(15)
Argyresthia retinella 99.31(13)
Argyresthia retinella 99.31(14)
Argyresthia retinella 99.31(10)
Argyresthia retinella 97.9
Argyresthia retinella 98.6
Argyresthia retinella 98.61(6)
Argyresthia retinella 99.31(16)
Argyresthia retinella 97.92(3)
Argyresthia retinella 99.31(17)
Argyresthia retinella 99.3(3)
Argyresthia retinella 97.92(4)
Argyresthia retinella 99.31(25)

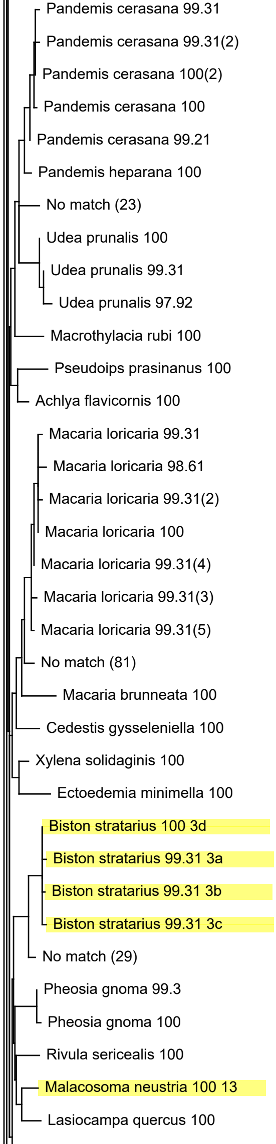
Pandemis cerasana 99.31
Pandemis cerasana 99.31(2)
Pandemis cerasana 100(2)
Pandemis cerasana 100
Pandemis cerasana 99.21
Pandemis heparana 100
No match (23)
Udea prunalis 100
Udea prunalis 99.31
Udea prunalis 97.92
Macrothylacia rubi 100
Pseudoips prasinanus 100
Achlya flavicornis 100
Macaria loricaria 99.31
Macaria loricaria 98.61
Macaria loricaria 99.31(2)
Macaria loricaria 100
Macaria loricaria 99.31(4)
Macaria loricaria 99.31(3)
Macaria loricaria 99.31(5)
No match (81)
Macaria brunneata 100
Cedestis gysseleniella 100
Xylena solidaginis 100
Ectoedemia minimella 100
Biston stratarius 100 3d
Biston stratarius 99.31 3a
Biston stratarius 99.31 3b
Biston stratarius 99.31 3c
No match (29)
Pheosia gnoma 99.3
Pheosia gnoma 100
Rivula sericealis 100
Malacosoma neustria 100 13
Lasiocampa quercus 100

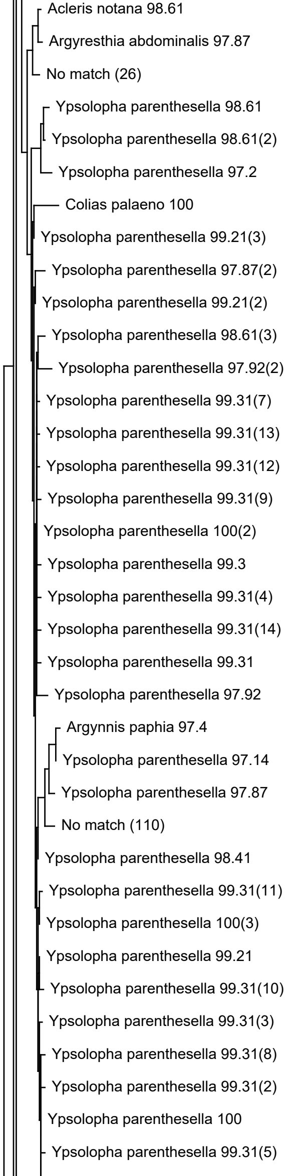
Acleris notana 98.61
Argyresthia abdominalis 97.87
No match (26)
Ypsolopha parenthesella 98.61
Ypsolopha parenthesella 98.61(2)
Ypsolopha parenthesella 97.2
Colias palaeno 100
Ypsolopha parenthesella 99.21(3)
Ypsolopha parenthesella 97.87(2)
Ypsolopha parenthesella 99.21(2)
Ypsolopha parenthesella 98.61(3)
Ypsolopha parenthesella 97.92(2)
Ypsolopha parenthesella 99.31(7)
Ypsolopha parenthesella 99.31(13)
Ypsolopha parenthesella 99.31(12)
Ypsolopha parenthesella 99.31(9)
Ypsolopha parenthesella 100(2)
Ypsolopha parenthesella 99.3
Ypsolopha parenthesella 99.31(4)
Ypsolopha parenthesella 99.31(14)
Ypsolopha parenthesella 99.31
Ypsolopha parenthesella 97.92
Argynnis paphia 97.4
Ypsolopha parenthesella 97.14
Ypsolopha parenthesella 97.87
No match (110)
Ypsolopha parenthesella 98.41
Ypsolopha parenthesella 99.31(11)
Ypsolopha parenthesella 100(3)
Ypsolopha parenthesella 99.21
Ypsolopha parenthesella 99.31(10)
Ypsolopha parenthesella 99.31(3)
Ypsolopha parenthesella 99.31(8)
Ypsolopha parenthesella 99.31(2)
Ypsolopha parenthesella 100
Ypsolopha parenthesella 99.31(5)

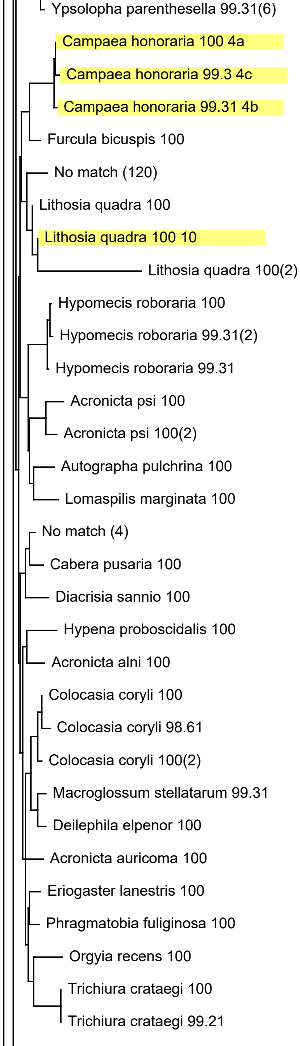
Ypsolopha parenthesella 99.31(6)
Campaea honoraria 100 4a
Campaea honoraria 99.3 4c
Campaea honoraria 99.31 4b
Furcula bicuspis 100
No match (120)
Lithosia quadra 100
Lithosia quadra 100 10
Lithosia quadra 100(2)
Hypomecis roboraria 100
Hypomecis roboraria 99.31(2)
Hypomecis roboraria 99.31
Acronicta psi 100
Acronicta psi 100(2)
Autographa pulchrina 100
Lomaspilis marginata 100
No match (4)
Cabera pusaria 100
Diacrisia sannio 100
Hypena proboscidalis 100
Acronicta alni 100
Colocasia coryli 100
Colocasia coryli 98.61
Colocasia coryli 100(2)
Macroglossum stellatarum 99.31
Deilephila elpenor 100
Acronicta auricoma 100
Eriogaster lanestris 100
Phragmatobia fuliginosa 100
Orgyia recens 100
Trichiura crataegi 100
Trichiura crataegi 99.21

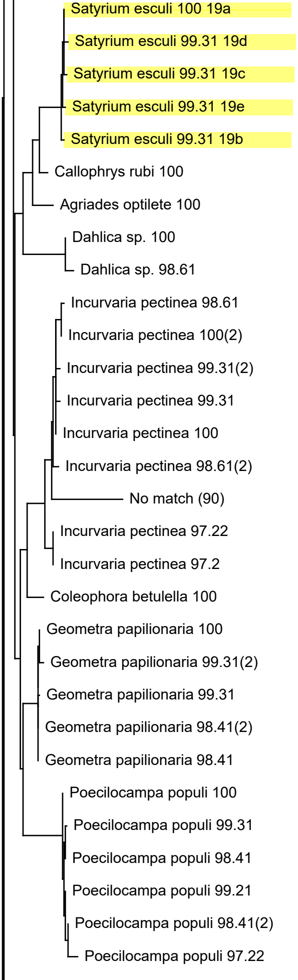
Satyrium esculi 100 19a
Satyrium esculi 99.31 19d
Satyrium esculi 99.31 19c
Satyrium esculi 99.31 19e
Satyrium esculi 99.31 19b
Callophrys rubi 100
Agriades optilete 100
Dahlica sp. 100
Dahlica sp. 98.61
Incurvaria pectinea 98.61
Incurvaria pectinea 100(2)
Incurvaria pectinea 99.31(2)
Incurvaria pectinea 99.31
Incurvaria pectinea 100
Incurvaria pectinea 98.61(2)
No match (90)
Incurvaria pectinea 97.22
Incurvaria pectinea 97.2
Coleophora betulella 100
Geometra papilionaria 100
Geometra papilionaria 99.31(2)
Geometra papilionaria 99.31
Geometra papilionaria 98.41(2)
Geometra papilionaria 98.41
Poecilocampa populi 100
Poecilocampa populi 99.31
Poecilocampa populi 98.41
Poecilocampa populi 99.21
Poecilocampa populi 98.41(2)
Poecilocampa populi 97.22

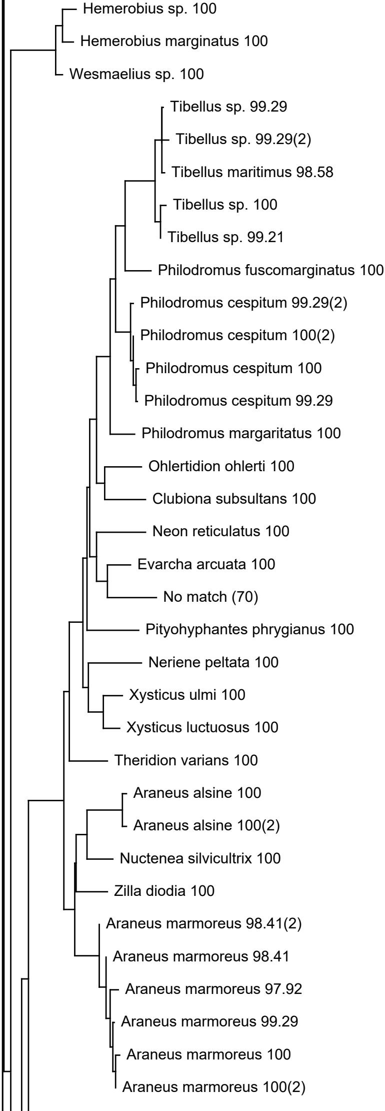
Hemerobius sp. 100
Hemerobius marginatus 100
Wesmaelius sp. 100
Tibellus sp. 99.29
Tibellus sp. 99.29(2)
Tibellus maritimus 98.58
Tibellus sp. 100
Tibellus sp. 99.21
Philodromus fuscomarginatus 100
Philodromus cespitum 99.29(2)
Philodromus cespitum 100(2)
Philodromus cespitum 100
Philodromus cespitum 99.29
Philodromus margaritatus 100
Ohlertidion ohlerti 100
Clubiona subsultans 100
Neon reticulatus 100
Evarcha arcuata 100
No match (70)
Pityohyphantes phrygianus 100
Neriene peltata 100
Xysticus ulmi 100
Xysticus luctuosus 100
Theridion varians 100
Araneus alsine 100
Araneus alsine 100(2)
Nuctenea silvicultrix 100
Zilla diodia 100
Araneus marmoreus 98.41(2)
Araneus marmoreus 98.41
Araneus marmoreus 97.92
Araneus marmoreus 99.29
Araneus marmoreus 100
Araneus marmoreus 100(2)

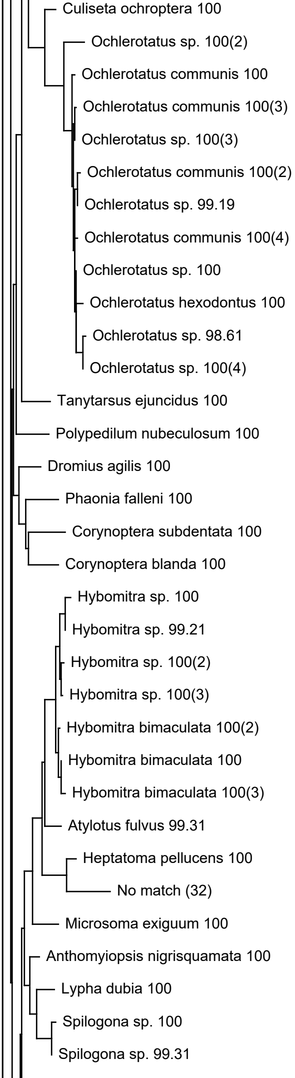
Culiseta ochroptera 100
Ochlerotatus sp. 100(2)
Ochlerotatus communis 100
Ochlerotatus communis 100(3)
Ochlerotatus sp. 100(3)
Ochlerotatus communis 100(2)
Ochlerotatus sp. 99.19
Ochlerotatus communis 100(4)
Ochlerotatus sp. 100
Ochlerotatus hexodontus 100
Ochlerotatus sp. 98.61
Ochlerotatus sp. 100(4)
Tanytarsus ejuncidus 100
Polypedilum nubeculosum 100
Dromius agilis 100
Phaonia falleni 100
Corynoptera subdentata 100
Corynoptera blanda 100
Hybomitra sp. 100
Hybomitra sp. 99.21
Hybomitra sp. 100(2)
Hybomitra sp. 100(3)
Hybomitra bimaculata 100(2)
Hybomitra bimaculata 100
Hybomitra bimaculata 100(3)
Atylotus fulvus 99.31
Heptatoma pellucens 100
No match (32)
Microsoma exiguum 100
Anthomyiopsis nigrisquamata 100
Lypha dubia 100
Spilogona sp. 100
Spilogona sp. 99.31

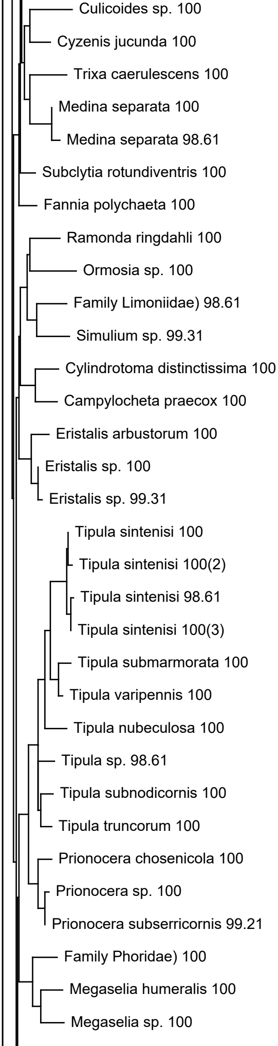
Culicoides sp. 100
Cyzenis jucunda 100
Trixa caerulescens 100
Medina separata 100
Medina separata 98.61
Subclytia rotundiventris 100
Fannia polychaeta 100
Ramonda ringdahli 100
Ormosia sp. 100
Family Limoniidae) 98.61
Simulium sp. 99.31
Cylindrotoma distinctissima 100
Campylocheta praecox 100
Eristalis arbustorum 100
Eristalis sp. 100
Eristalis sp. 99.31
Tipula sintenisi 100
Tipula sintenisi 100(2)
Tipula sintenisi 98.61
Tipula sintenisi 100(3)
Tipula submarmorata 100
Tipula varipennis 100
Tipula nubeculosa 100
Tipula sp. 98.61
Tipula subnodicornis 100
Tipula truncorum 100
Prionocera chosenicola 100
Prionocera sp. 100
Prionocera subserricornis 99.21
Family Phoridae) 100
Megaselia humeralis 100
Megaselia sp. 100

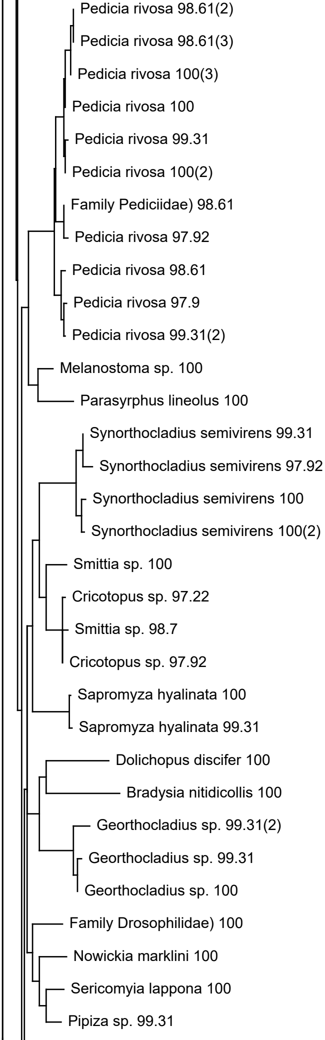

Pedicia rivosa 98.61(2)
Pedicia rivosa 98.61(3)
Pedicia rivosa 100(3)
Pedicia rivosa 100
Pedicia rivosa 99.31
Pedicia rivosa 100(2)
Family Pediciidae) 98.61
Pedicia rivosa 97.92
Pedicia rivosa 98.61
Pedicia rivosa 97.9
Pedicia rivosa 99.31(2)
Melanostoma sp. 100
Parasyrphus lineolus 100
Synorthocladius semivirens 99.31
Synorthocladius semivirens 97.92
Synorthocladius semivirens 100
Synorthocladius semivirens 100(2)
Smittia sp. 100
Cricotopus sp. 97.22
Smittia sp. 98.7
Cricotopus sp. 97.92
Sapromyza hyalinata 100
Sapromyza hyalinata 99.31
Dolichopus discifer 100
Bradysia nitidicollis 100
Georthocladius sp. 99.31(2)
Georthocladius sp. 99.31
Georthocladius sp. 100
Family Drosophilidae) 100
Nowickia marklini 100
Sericomyia lappona 100
Pipiza sp. 99.31

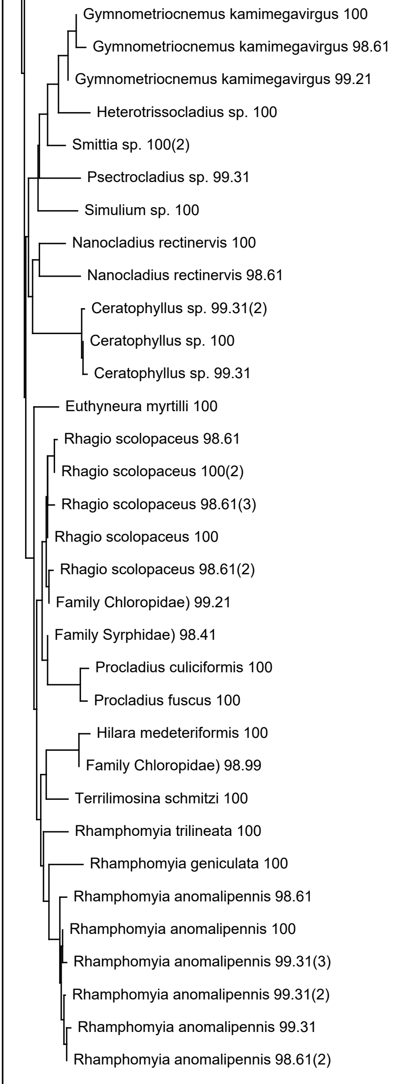
Gymnometriocnemus kamimegavirgus 100
Gymnometriocnemus kamimegavirgus 98.61
Gymnometriocnemus kamimegavirgus 99.21
Heterotrissocladius sp. 100
Smittia sp. 100(2)
Psectrocladius sp. 99.31
Simulium sp. 100
Nanocladius rectinervis 100
Nanocladius rectinervis 98.61
Ceratophyllus sp. 99.31(2)
Ceratophyllus sp. 100
Ceratophyllus sp. 99.31
Euthyneura myrtilli 100
Rhagio scolopaceus 98.61
Rhagio scolopaceus 100(2)
Rhagio scolopaceus 98.61(3)
Rhagio scolopaceus 100
Rhagio scolopaceus 98.61(2)
Family Chloropidae) 99.21
Family Syrphidae) 98.41
Procladius culiciformis 100
Procladius fuscus 100
Hilara medeteriformis 100
Family Chloropidae) 98.99
Terrilimosina schmitzi 100
Rhamphomyia trilineata 100
Rhamphomyia geniculata 100
Rhamphomyia anomalipennis 98.61
Rhamphomyia anomalipennis 100
Rhamphomyia anomalipennis 99.31(3)
Rhamphomyia anomalipennis 99.31(2)
Rhamphomyia anomalipennis 99.31
Rhamphomyia anomalipennis 98.61(2)

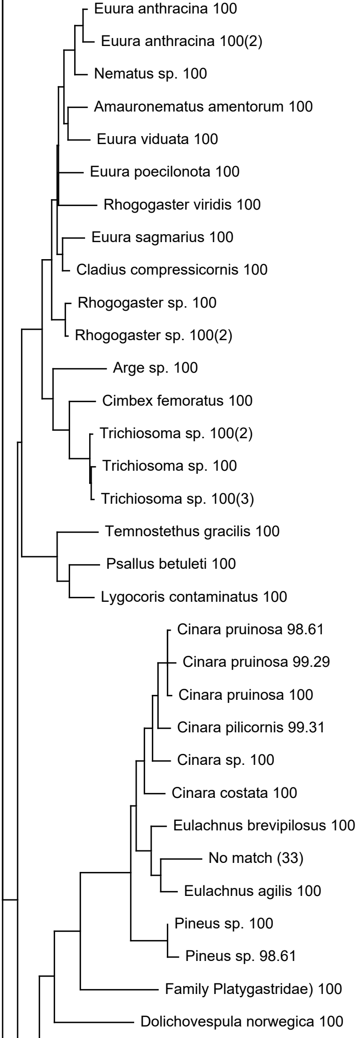
Euura anthracina 100
Euura anthracina 100(2)
Nematus sp. 100
Amauronematus amentorum 100
Euura viduata 100
Euura poecilonota 100
Rhogogaster viridis 100
Euura sagmarius 100
Cladius compressicornis 100
Rhogogaster sp. 100
Rhogogaster sp. 100(2)
Arge sp. 100
Cimbex femoratus 100
Trichiosoma sp. 100(2)
Trichiosoma sp. 100
Trichiosoma sp. 100(3)
Temnostethus gracilis 100
Psallus betuleti 100
Lygocoris contaminatus 100
Cinara pruinosa 98.61
Cinara pruinosa 99.29
Cinara pruinosa 100
Cinara pilicornis 99.31
Cinara sp. 100
Cinara costata 100
Eulachnus brevipilosus 100
No match (33)
Eulachnus agilis 100
Pineus sp. 100
Pineus sp. 98.61
Family Platygastridae) 100
Dolichovespula norwegica 100

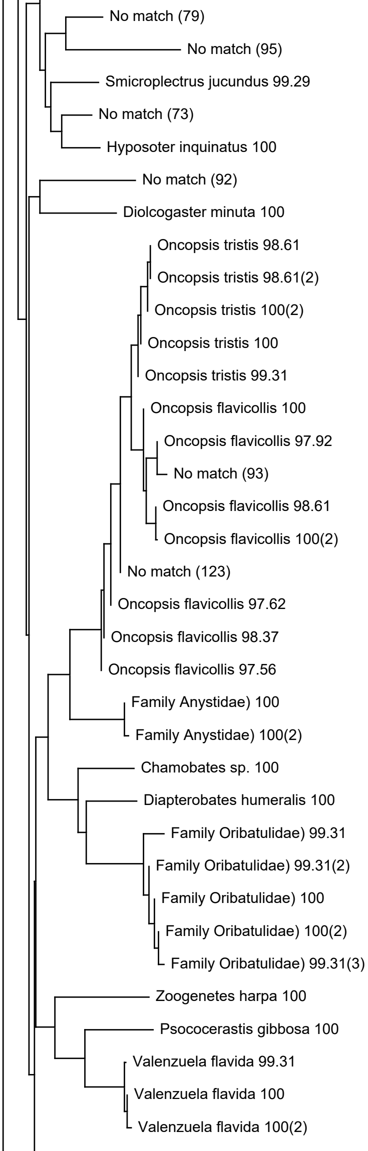
No match (79)
No match (95)
Smicroplectrus jucundus 99.29
No match (73)
Hyposoter inquinatus 100
No match (92)
Diolcogaster minuta 100
Oncopsis tristis 98.61
Oncopsis tristis 98.61(2)
Oncopsis tristis 100(2)
Oncopsis tristis 100
Oncopsis tristis 99.31
Oncopsis flavicollis 100
Oncopsis flavicollis 97.92
No match (93)
Oncopsis flavicollis 98.61
Oncopsis flavicollis 100(2)
No match (123)
Oncopsis flavicollis 97.62
Oncopsis flavicollis 98.37
Oncopsis flavicollis 97.56
Family Anystidae) 100
Family Anystidae) 100(2)
Chamobates sp. 100
Diapterobates humeralis 100
Family Oribatulidae) 99.31
Family Oribatulidae) 99.31(2)
Family Oribatulidae) 100
Family Oribatulidae) 100(2)
Family Oribatulidae) 99.31(3)
Zoogenetes harpa 100
Psococerastis gibbosa 100
Valenzuela flavida 99.31
Valenzuela flavida 100
Valenzuela flavida 100(2)

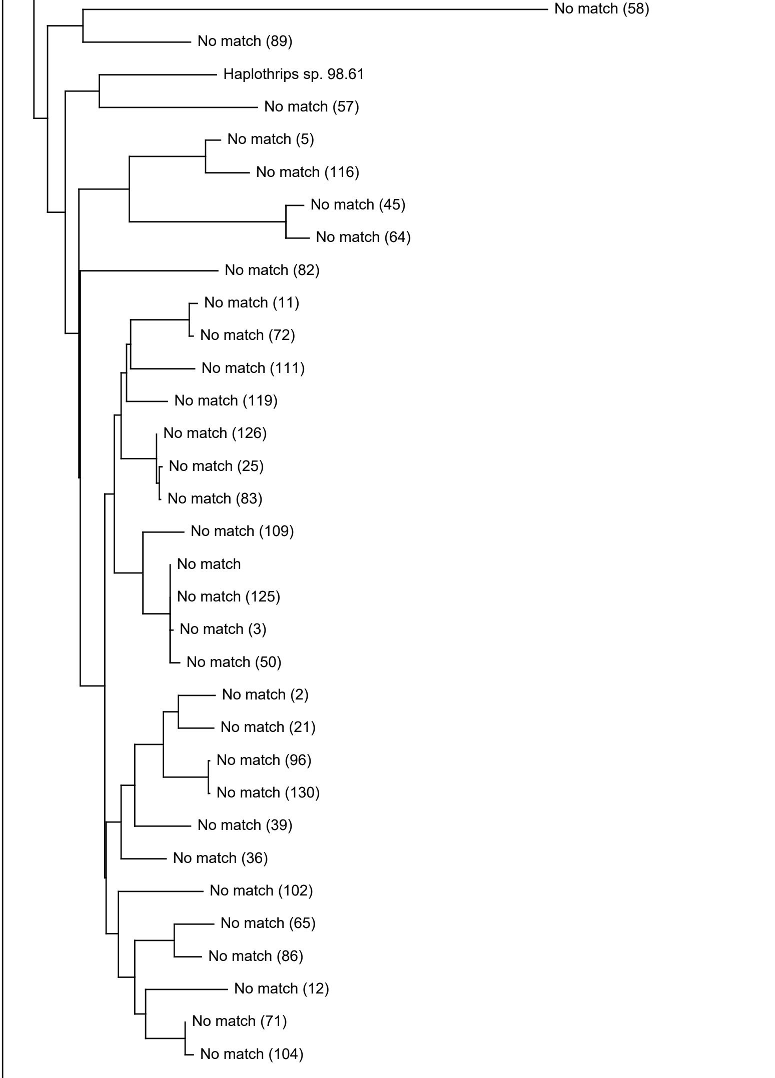
No match (58)
No match (89)
Haplothrips sp. 98.61
No match (57)
No match (5)
No match (116)
No match (45)
No match (64)
No match (82)
No match (11)
No match (72)
No match (111)
No match (119)
No match (126)
No match (25)
No match (83)
No match (109)
No match
No match (125)
No match (3)
No match (50)
No match (2)
No match (21)
No match (96)
No match (130)
No match (39)
No match (36)
No match (102)
No match (65)
No match (86)
No match (12)
No match (71)
No match (104)

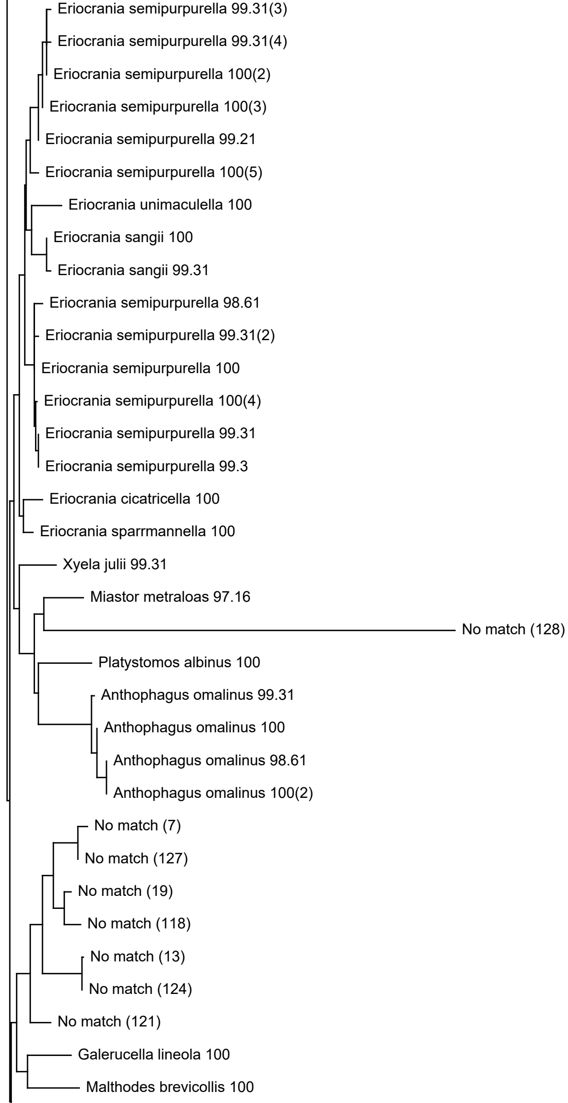

Eriocrania semipurpurella 99.31(3)
Eriocrania semipurpurella 99.31(4)
Eriocrania semipurpurella 100(2)
Eriocrania semipurpurella 100(3)
Eriocrania semipurpurella 99.21
Eriocrania semipurpurella 100(5)
Eriocrania unimaculella 100
Eriocrania sangii 100
Eriocrania sangii 99.31
Eriocrania semipurpurella 98.61
Eriocrania semipurpurella 99.31(2)
Eriocrania semipurpurella 100
Eriocrania semipurpurella 100(4)
Eriocrania semipurpurella 99.31
Eriocrania semipurpurella 99.3
Eriocrania cicatricella 100
Eriocrania sparrmannella 100
Xyela julii 99.31
Miastor metraloas 97.16
No match (128)
Platystomos albinus 100
Anthophagus omalinus 99.31
Anthophagus omalinus 100
Anthophagus omalinus 98.61
Anthophagus omalinus 100(2)
No match (7)
No match (127)
No match (19)
No match (118)
No match (13)
No match (124)
No match (121)
Galerucella lineola 100
Malthodes brevicollis 100

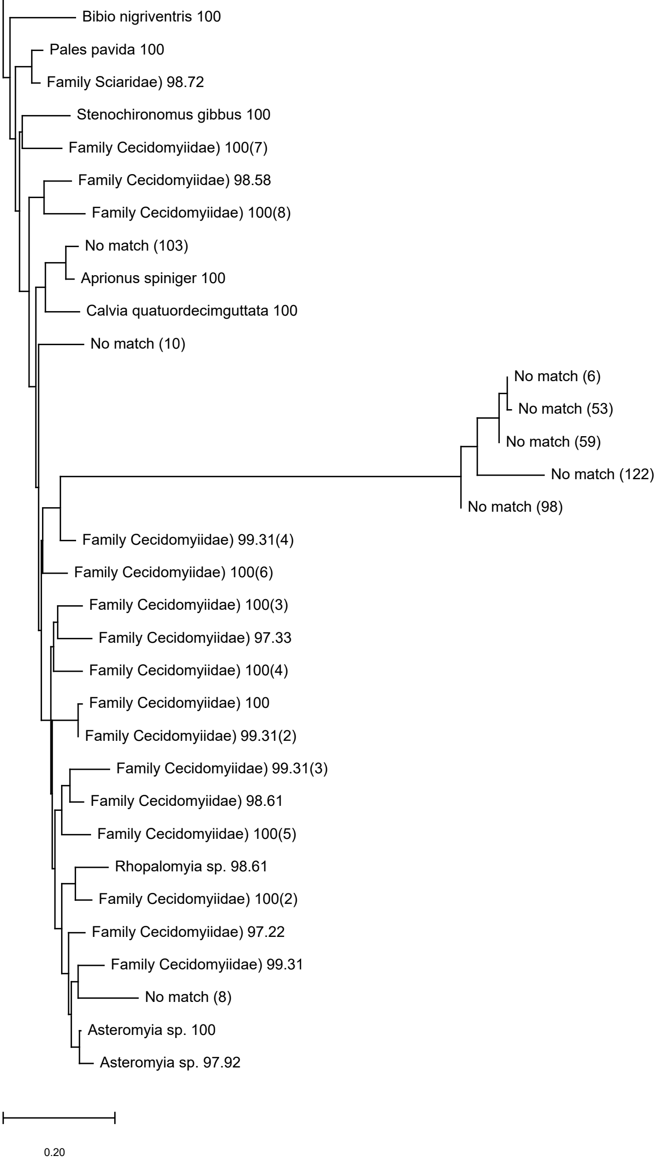
Bibio nigriventris 100
Pales pavida 100
Family Sciaridae) 98.72
Stenochironomus gibbus 100
Family Cecidomyiidae) 100(7)
Family Cecidomyiidae) 98.58
Family Cecidomyiidae) 100(8)
No match (103)
Aprionus spiniger 100
Calvia quatuordecimguttata 100
No match (10)
No match (6)
No match (53)
No match (59)
No match (122)
No match (98)
Family Cecidomyiidae) 99.31(4)
Family Cecidomyiidae) 100(6)
Family Cecidomyiidae) 100(3)
Family Cecidomyiidae) 97.33
Family Cecidomyiidae) 100(4)
Family Cecidomyiidae) 100
Family Cecidomyiidae) 99.31(2)
Family Cecidomyiidae) 99.31(3)
Family Cecidomyiidae) 98.61
Family Cecidomyiidae) 100(5)
Rhopalomyia sp. 98.61
Family Cecidomyiidae) 100(2)
Family Cecidomyiidae) 97.22
Family Cecidomyiidae) 99.31
No match (8)
Asteromyia sp. 100
Asteromyia sp. 97.92
0.20
